# Dose-escalation studies of mesenchymal stromal cell therapy for decompensated liver cirrhosis: phase Ia/Ib results and immune modulation insights

**DOI:** 10.1038/s41392-025-02318-4

**Published:** 2025-07-29

**Authors:** Lei Shi, Ziying Zhang, Song Mei, Zerui Wang, Zhe Xu, Weiqi Yao, Limin Liu, Mengqi Yuan, Yuefei Pan, Kaidi Zhu, Kai Liu, Fanglin Meng, Jiao Sun, Wenying Liu, Xiaohui Xie, Tengyun Dong, Lei Huang, Fanping Meng, Jun-Liang Fu, Yuanyuan Li, Chao Zhang, Xing Fan, Ming Shi, Yu Zhang, Yonggang Li, Wei-Fen Xie, Peng Zhang, Fu-Sheng Wang

**Affiliations:** 1 Senior Department of Infectious Diseases, The Fifth Medical Center of Chinese PLA General Hospital, National Clinical Research Center for Infectious Diseases, Beijing, China; 2 Beijing Key Laboratory for Genetics of Birth Defects, Beijing Pediatric Research Institute, MOE Key Laboratory of Major Diseases in Children; Rare Disease Center, Beijing Children’s Hospital, Capital Medical University, National Center for Children’s Health, Beijing, China; 3 Shanghai Institute of Immunology, Department of Immunology and Microbiology, Shanghai Jiao Tong University School of Medicine, Shanghai, China; 4 Senior Department of Gastroenterology, the First Medical Center of Chinese PLA General Hospital, Beijing, China; 5 School of Life and Health Sciences, Hubei University of Technology, Wuhan, China; 6 Wuhan Optics Valley Zhongyuan Pharmaceutical Co., Ltd., Hubei, China; 7 Department of Gastroenterology, Changzheng Hospital, Second Military Medical University, Shanghai, China

**Keywords:** Stem-cell research, Mesenchymal stem cells, Clinical trials

## Abstract

Decompensated liver cirrhosis (DLC) is characterized by severe liver dysfunction and immune dysregulation, posing significant treatment challenges. Mesenchymal stromal cell (MSC) therapy has shown promise in DLC treatment, but the optimal dosing strategies and dose-dependent therapeutic mechanisms in humans remain unclear, limiting its clinical application. We conducted sequential Phase Ia/Ib trials using a single-arm, dose-escalation design to evaluate the safety and tolerability of MSC therapy in DLC patients while also exploring its immunomodulatory effects and gathering preliminary therapeutic signals. In Phase Ia, four dose cohorts received a single dose of MSCs: 5.0 × 10⁷, 1.0 × 10⁸, 1.5 × 10⁸, and 2.0 × 10⁸ cells. Patients were followed up on Days 3, 7, 14, and 28. Multiomics analyses, including single-cell RNA sequencing and cytometry by time of flight, were conducted to perform exploratory mechanistic analyses investigating immune cell dynamics and dose-dependent responses. Building on these findings, Phase Ib included two dose cohorts, each of which received three doses of MSCs administered one week apart: 1.0 × 10⁸ and 2.0 × 10⁸ cells per dose. Patients were followed up on Days 7, 14, 21, and 28 to further evaluate the safety and feasibility of multiple-dose regimens. The trials were registered at ClinicalTrials.gov (NCT05227846 and NCT05984303). MSC therapy demonstrated good safety and tolerability in both Phase Ia and Phase Ib trials, with no severe adverse events, dose-limiting toxicities, or serious unexpected adverse reactions observed up to Day 28. Multi-omics analyses revealed that higher MSC doses elicited stronger immunomodulatory effects, particularly by modulating monocyte subsets. In particular, myxovirus resistance 1 positive (MX1^+^) monocytes, a key monocyte population, exhibited dose-dependent changes and were identified as a mediator of MSC-induced immunomodulation. These effects were sustained for up to seven days post-treatment but diminished by Day 14. Preliminary clinical signals included improvements in Child–Pugh scores, Model for End-Stage Liver Disease scores, liver function markers, and quality-of-life metrics, particularly in the higher-dose and multiple-dose groups. This study demonstrates the safety and tolerability of MSC therapy in patients with DLC and provides the first human-based evidence on the dose‒effect relationship and optimal administration regimens. The identification of MX1^+^ monocytes as a critical mediator highlights the potential of MSC therapy to modulate immune dysfunction in DLC. These findings offer valuable insights for optimizing MSC therapy and informing the design of future efficacy-focused clinical trials.

## Introduction

Decompensated liver cirrhosis (DLC) represents the advanced stage of liver cirrhosis, characterized by severe liver dysfunction and complications such as ascites, variceal bleeding, hepatic encephalopathy, and increased infection risk, resulting in substantial global morbidity and mortality.^1,2^ The diminished capacity of the liver for regeneration and metabolic function in DLC impacts various organs and systems, including the immune system.^3,4^ Cirrhosis-associated immune dysfunction (CAID), defined by a paradoxical combination of systemic inflammation and immune deficiency, is a critical contributor to DLC progression.^5^ Elevated levels of proinflammatory cytokines, endotoxins, and damage- and pathogen-associated molecular patterns (DAMPs and PAMPs) drive systemic inflammation, leading to tissue injury.^6–8^ Simultaneously, immune deficiency arises from impaired immune cell function and is characterized by a reduction in pathogen clearance. This immune dysfunction predisposes patients to frequent and severe infections and promotes bacterial translocation, thereby exacerbating liver damage, contributing to multiorgan failure, and ultimately leading to poor prognosis.^4,9^

Liver transplantation remains the only curative option for DLC but is limited by donor shortages, rejection risks, and high costs. Addressing CAID is challenging, as targeting systemic inflammation can increase infection risk, whereas stimulating the immune response may worsen immunopathology.^10,11^ Thus, effective treatment of CAID necessitates strategies that modulate the immune response rather than inhibiting or excessively stimulating it.^4^

Mesenchymal stromal cells (MSCs) have gained recognition as promising cell therapy candidates due to their ease of access, low immunogenicity, differentiation potential, and potent immunomodulatory capabilities.^12^ Characterized by immune dysregulation and progressive tissue injury, DLC has emerged as a favorable target for MSC therapy, given the potential of MSCs to restore immune homeostasis.^13–15^ Clinical studies, including work from our group, have documented improvements in metrics such as model for end-stage liver disease (MELD) scores, prothrombin time, and liver function following MSC infusions.^16–18^ However, the clinical results remain mixed, with some studies reporting limited or no significant benefits.^19,20^ These variations may stem from differences in MSC dosing, administration intervals, inclusion criteria, and outcome definitions, which pose challenges to standardizing MSC therapy in patients with cirrhosis.^21^

While the immunomodulatory potential of MSCs is recognized as a key therapeutic benefit,^17,21^ most mechanistic insights have been derived from preclinical studies,^17,22–24^ with limited evidence available from human trials.^25^ A clearer understanding of the mechanisms underlying MSC persistence and activity in humans is critical for optimizing dosing strategies and treatment intervals.^21^ Notably, the dose‒dependent effects of MSCs in human DLC have rarely been systematically investigated, and the dose‒response relationship of MSC-mediated immunomodulation remains poorly understood. This knowledge gap represents a major obstacle to the effective clinical application of MSCs in DLC, highlighting the urgent need for targeted research to address these challenges and facilitate precise clinical translation.

To address these gaps, we conducted sequential, single-arm, dose-escalation phase I clinical trials to evaluate the safety and tolerability of MSC therapy in DLC patients while exploring its immunomodulatory effects and gathering preliminary signals to guide future efficacy-focused studies. The first trial (Phase Ia) assessed the safety of single-dose MSC administration and utilized multiomics analyses to elucidate dose-dependent immune responses, providing the first human-based insights into MSC-induced immunomodulation in DLC. On the basis of these findings, we designed and implemented a multiple-dose dose-escalation trial (Phase Ib) to refine the dosing regimen and optimize the treatment intervals. By integrating clinical observations with advanced multiomics analyses, our work aims to address the critical gap in understanding the therapeutic and immunological mechanisms of MSCs in DLC, thereby laying the groundwork for advancing MSC therapy in this challenging condition.

## Results

### Patient characteristics

**Fig. 1:**
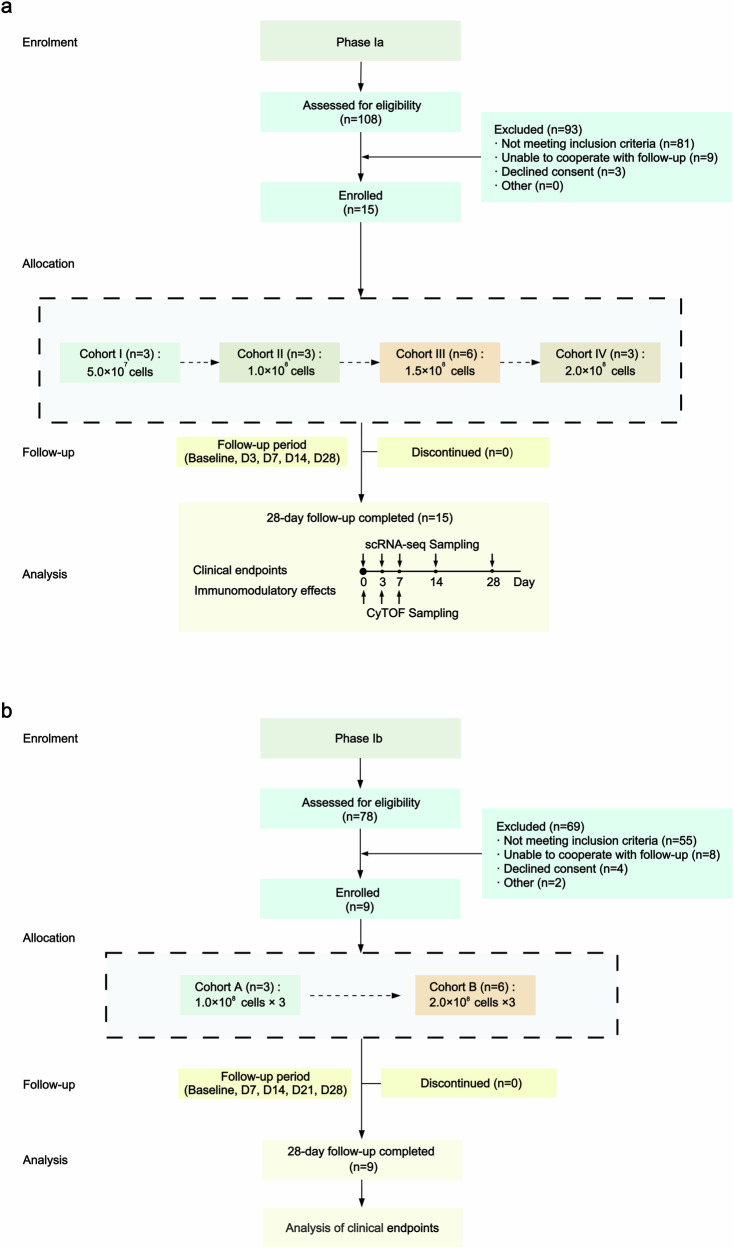
Study design. **a** Phase Ia; **b** Phase Ib. scRNA-seq (single-cell RNA sequencing) profiles transcriptomes across thousands of individual cells, whereas CyTOF (cytometry by time of flight) uses metal-tagged antibodies to simultaneously quantify >40 protein markers at single-cell resolution. Integrated analysis enables cross-validation between immune cell surface protein expression and transcriptional states

A total of 186 patients with DLC were screened from March 2022 to March 2024. The study comprised two stages (Phases Ia and Ib), as shown in Fig. 1. Phase Ia enrolled 15 patients who received MSC infusions following a “3 + 3” dose-escalation protocol. Three patients were enrolled in Cohorts I, II, and IV, while Cohort III included six patients. As per the study protocol, the enrollment of additional patients in Cohort III was necessitated by an adverse event (rash, level 1, possibly related) observed in one patient on the second day post-infusion. After the 28-day follow-up, MSC dosages and the interval of Phase Ib were determined based on Phase Ia results. Phase Ib enrolled nine patients. Cohort A (1.0 × 10⁸ cells per dose, three doses) included three patients, while Cohort B (2.0 × 10⁸ cells per dose, three doses) included six patients to comprehensively evaluate safety.

The median age of the participants was 51.0 ± 10.1 years; 15 (62.5%) were males, and 9 (37.5%) were females. The median MELD score was 12.38 ± 3.42, and the median Child‒Pugh score was 8.12 ± 1.36. The leading etiologies were HBV infection (9/24, 37.5%) and alcohol use (9/24, 37.5%), with fewer cases attributed to primary biliary cirrhosis (5/24, 20.8%) or HCV infection (1/24, 4.2%). Patients exhibited liver dysfunction (median MELD score: 12.38 ± 3.42; Child‒Pugh score: 8.12 ± 1.36) and typical complications: ascites (Phase Ia: 13/15, 86.7%; Phase Ib: 6/9, 66.7%), esophageal/gastric varices (Phase Ia: 11/15, 73.3%; Phase Ib: 8/9, 88.9%), and hepatic encephalopathy (Phase Ia: 3/15, 20%; Phase Ib: 4/9, 44.4%). The comorbidities included gastrointestinal diseases (15/24, 62.5%), diabetes (4/24, 16.7%), and hypertension (1/24, 4.2%). The baseline characteristics of all patients are presented in Table 1. All patients completed the 28-day follow-up without death, withdrawal, or loss to follow-up.

**Table 1:** Clinical and demographic features

	Phase Ia				Phase Ib		Total (*n* = 24)
	Cohort I (*n* = 3)	Cohort II (*n* = 3)	Cohort III (*n* = 6)	Cohort IV (*n* = 3)	Cohort A (*n* = 3)	Cohort B (*n* = 6)	
**Age (years)**	46.00 ± 11.14	49.33 ± 8.62	60.17 ± 6.65	42.00 ± 12.53	56.33 ± 12.06	47.00 ± 4.69	51.00 ± 10.10
**Sex,** ***n*** **(%)**							
male	3 (100.00)	2 (66.67)	1 (16.67)	2 (66.67)	3 (100.00)	4 (66.67)	15 (62.50)
female	0 (0.00)	1 (33.33)	5 (83.33)	1 (33.33)	0 (0.00)	2 (33.33)	9 (37.50)
**BMI(kg/m**^2^**)**	24.86 ± 1.97	24.89 ± 1.72	20.45 ± 2.83	24.00 ± 2.92	24.55 ± 1.98	25.58 ± 5.72	23.79 ± 3.83
**Pathogenesis,** ***n*** **(%)**							
Hepatitis B	2 (66.67)	1 (33.33)	1 (16.67)	3 (100.00)	0 (0.00)	2 (33.33)	9 (37.50)
Hepatitis C	0 (0.00)	0 (0.00)	1 (16.67)	0 (0.00)	0 (0.00)	0 (0.00)	1 (4.17)
Alcohol	1 (33.33)	1 (33.33)	1 (16.67)	0 (0.00)	3 (100.00)	3 (50.00)	9 (37.50)
Primary biliary cirrhosis	0 (0.00)	1 (33.33)	3 (50.00)	0 (0.00)	0 (0.00)	1 (16.67)	5 (20.83)
**MELD Score**	12.67 ± 3.21	16.67 ± 1.15	11.67 ± 4.27	12.33 ± 3.51	12.00 ± 3.46	11.00 ± 2.68	12.38 ± 3.42
**Child–Pugh Score**	7.33 ± 0.58	9.00 ± 2.00	8.50 ± 1.87	7.67 ± 1.15	8.33 ± 1.15	7.83 ± 0.98	8.12 ± 1.36
**EQ-5D VAS**	76.67 ± 15.28	89.67 ± 8.08	74.17 ± 18.00	65.00 ± 13.23	69.67 ± 9.50	74.17 ± 10.21	74.71 ± 13.70
**CLQD**, M (Q₁, Q₃)	181.00 (166.00,183.50)	201.00 (195.50,202.00)	177.50 (167.50,182.25)	116.00 (110.50,132.00)	158.00 (128.50,166.00)	132.50 (104.75,144.50)	154.50 (120.50, 181.50)
**Comorbidities,** ***n*** **(%)**							
Gastrointestinal diseases	1 (33.33)	1 (33.33)	3 (50.00)	1 (33.33)	3 (100.00)	6 (100.00)	15 (62.50)
Diabetes	0 (0.00)	0 (0.00)	0 (0.00)	1 (33.33)	2 (66.67)	1 (16.67)	4 (16.67)
Hypertension	0 (0.00)	0 (0.00)	1 (16.67)	0 (0.00)	0 (0.00)	0 (0.00)	1 (4.17)
**Complications of liver cirrhosis,** ***n*** **(%)**							
Ascites	3 (100.00)	2 (66.67)	5 (83.33)	3 (100.00)	3 (100.00)	3 (50.00)	19 (79.17)
Esophageal and gastric varices	2 (66.67)	3 (100.00)	5 (83.33)	1 (33.33)	3 (100.00)	5 (83.33)	19 (79.17)
Hepatic encephalopathy	0 (0.00)	0 (0.00)	2 (33.33)	1 (33.33)	1 (33.33)	3 (50.00)	7 (29.17)
**laboratory data**							
WBC, (10^9^/L)	1.95 ± 0.83	2.53 ± 1.04	2.72 ± 1.03	3.23 ± 0.73	3.47 ± 1.89	3.32 ± 1.27	2.91 ± 1.16
Hemoglobin, (g/L)	122.33 ± 26.50	97.00 ± 13.00	104.17 ± 16.46	113.33 ± 24.66	92.67 ± 21.36	105.33 ± 16.79	105.54 ± 19.10
Prothrombin time activity,(%)	62.97 ± 23.59	43.57 ± 6.46	72.52 ± 32.10	66.17 ± 16.55	64.40 ± 18.89	65.18 ± 9.32	64.06 ± 20.60
Total bilirubin,(umol/L)	35.07 ± 18.75	52.80 ± 13.76	39.87 ± 19.43	39.80 ± 23.76	32.23 ± 10.75	35.22 ± 31.34	38.76 ± 20.94
Albumin,(g/L)	41.67 ± 11.59	31.67 ± 6.11	32.17 ± 4.45	32.67 ± 5.03	33.67 ± 4.73	30.17 ± 5.64	33.04 ± 6.53
Prealbumin,(mg/L)	85.58 ± 37.93	104.67 ± 16.26	58.33 ± 25.17	73.17 ± 30.52	107.00 ± 64.37	118.33 ± 50.52	75.00 ± 27.31
Cholinesterase,(U/L)	3333.00 ± 762.04	1524.00 ± 340.99	2937.50 ± 1391.54	4757.00 ± 3896.82	3599.33 ± 1836.31	2800.83 ± 715.59	3086.25 ± 1719.97

*BMI* Weight(kg)/Height(m)^2^

### Safety

The MSC infusion was well tolerated, with no adverse hemodynamic or respiratory changes observed during or up to 30-min post-infusion. Adverse events (AEs) up to Day 28 are summarized in Table 2. In total, ten AEs were reported in phases Ia and Ib. Four AEs occurred in Phase Ia: one rash (level 2, unlikely to be related) in Cohort II; one case each of rash (level 1, possibly related) and renal stone (level 2, unlikely to be related) in Cohort III; and one case of influenza-like symptoms (level 1, unlikely to be related) in Cohort IV. The rash in Patient 2 of Cohort III was deemed possibly related to MSC infusion, prompting the inclusion of three additional patients in this cohort; none developed a new rash. Six AEs occurred in Phase Ib: one case of diarrhea (level 2, unlikely to be related), one case of bleeding gums (level 2, unlikely to be related), one upper respiratory tract infection (level 3, possibly related) in Cohort A, and two cases of fever (level 1, possibly related) and one case of hemorrhoids (level 2, unlikely to be related) in Cohort B. The three above-reported AEs were judged as possibly related to the MSC infusion due to the close time relationship. Nevertheless, they may also be attributable to DLC or other concomitant drugs and may be unrelated to MSC treatment.

**Table 2:** Adverse events up to Day 28

	Phase Ia				Phase Ib		Total, *n*
	Cohort I (*n* = 3)	Cohort II (*n* = 3)	Cohort III (*n* = 6)	Cohort IV (*n* = 3)	Cohort A (*n* = 3)	Cohort B (*n* = 6)	
**Adverse event**	0	1	2	1	3	3	10
**Relationship to MSCs treatment**							
Definitely related	0	0	0	0	0	0	0
Probably related	0	0	0	0	0	0	0
Possibly related	0	0	1	0	1	2	4
Unlikely to be related	0	1	1	1	2	1	6
Not related	0	0	0	0	0	0	0
**Severity level**							
Level 1	0	0	1	1	0	2	4
Level 2	0	1	1	0	2	1	5
Level 3	0	0	0	0	1	0	1
Level 4	0	0	0	0	0	0	0
Level 5	0	0	0	0	0	0	0
**Serious adverse event**	0	0	0	0	0	0	0
**Adverse events resulted in death**	0	0	0	0	0	0	0
**Categories**							
Rash	0	1	1	0	0	0	2
Fever	0	0	0	0	0	2	2
Upper respiratory tract infection	0	0	0	0	1	0	1
Influenza-like symptoms	0	0	0	1	0	0	1
Kidney calculi	0	0	1	0	0	0	1
Diarrhea	0	0	0	0	1	0	1
Hemorrhoids	0	0	0	0	0	1	1
Bleeding gums	0	0	0	0	1	0	1
**Adverse events outcome**							
Recovered	0	0	0	1	3	0	4
Improvement	0	1	2	0	0	3	6
Persistence	0	0	0	0	0	0	0
Aggravation	0	0	0	0	0	0	0
Sequelae	0	0	0	0	0	0	0
Death	0	0	0	0	0	0	0

Throughout the 28-day observation period, there were no dose-limiting toxicities (DLTs), AEs leading to drug discontinuation, withdrawals due to AEs, serious adverse events (SAEs), or suspected unexpected serious adverse reactions (SUSARs).

### Preliminary exploratory endpoints

**Fig. 2:**
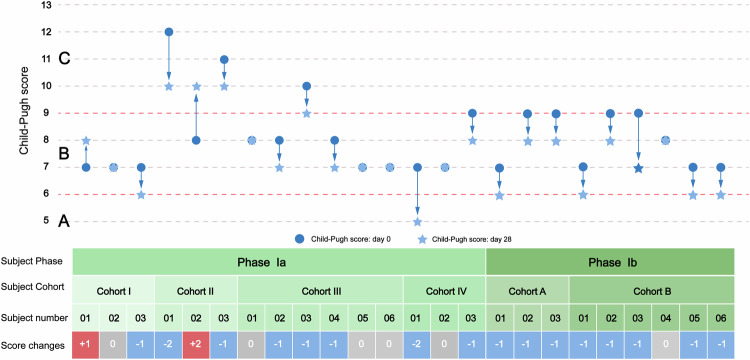
Changes in individual Child‒Pugh scores from baseline to Day 28. **A** child class A; **B** child class B; **C** child class C

On day 28, the Child‒Pugh score decreased in 53.3% (*n* = 8) of the participants in Phase Ia, while increases were observed in 13.3% (*n* = 2), limited to Cohorts 1 and 2, with no increases recorded in Cohorts 3 and 4. In Phase Ib, the Child‒Pugh score decreased in 88.9% (*n* = 8) of the participants, with 3 patients in Cohort A and 5 in Cohort B, and no increases were reported in either cohort. Overall, the higher-dose groups were more likely to have lower Child‒Pugh scores across both phases. (Fig. 2)

From baseline to day 28 in Phase Ia, 33.3% (*n* = 5) of the participants had decreased MELD scores, with reductions in Cohorts I, II, III, and IV by 1, 0, 1, and 3 cases, respectively. In contrast, the MELD scores increased in 2, 2, and 3 cases in Cohorts I, II, and III, while Cohort IV had no increases. In Phase Ib, 44.4% (*n* = 4) of participants had decreased MELD scores, with decreases of 1 case in Cohort A and 3 cases in Cohort B; no increases were observed in Cohort B (Table 3). Data at additional time points are provided in Supplementary Table 1,2.

**Table 3:** Clinical outcome indicators at Day 28

	Phase Ia				Phase Ib		Total *n* (%)
	Cohort I (*n* = 3)	Cohort II (*n* = 3)	Cohort III (*n* = 6)	Cohort IV (*n* = 3)	Cohort A (*n* = 3)	Cohort B (*n* = 6)	
**MELD score changes**							
Up	2	2	3	0	2	0	9
Down	1	0	1	3	1	3	9
Unchanged	0	1	2	0	0	3	6
Missing	0	0	0	0	0	0	0
**Child–Pugh score changes**							0
Up	1	1	1	0	0	0	3
Down	1	2	3	2	3	5	16
Unchanged	1	0	2	1	0	1	5
Missing	0	0	0	0	0	0	0
**EQ-5D-5L scale utility index changes**							0
Up	1	0	2	0	1	4	8
Down	0	1	1	2	0	1	5
Unchanged	3	1	2	1	2	1	10
Missing	0	1	1	0	0	0	2
**EQ-5D-5L scale VAS Score**							0
Up	1	0	2	1	0	3	7
Down	1	1	3	2	2	2	11
Unchanged	1	1	0	0	1	1	4
Missing	0	1	1	0	0	0	2
**Chronic Liver Disease Questionnaire** **Score**							0
Up	2	0	1	2	2	6	13
Down	1	2	4	1	1	0	9
Unchanged	0	0	0	0	0	0	0
Missing	0	1	1	0	0	0	2
**Incidence of complications in DLC,** ***n***	0	0	1	0	0	0	1
**Survival without liver transplantation,** ***n***	3	3	6	3	3	6	24
**Occurrence of liver failure,** *n*	0	0	0	0	0	0	0
**Incidence of liver cancer,** ***n***	0	0	0	0	0	0	0
**Albumin (g/L)**							0
Up	1	2	4	1	3	5	16
Down	2	1	2	2	0	0	7
Unchanged	0	0	1	0	0	1	2
Missing	0	0	0	0	0	0	0
**Prealbumin (g/L)**							0
Up	2	1	3	2	1	5	14
Down	1	1	2	1	2	1	8
Unchanged	0	0	0	0	0	0	0
Missing	0	1	1	0	0	0	2
**Total bilirubin (μmol/L)**							0
Up	2	2	4	0	2	3	13
Down	1	1	2	3	1	3	11
Unchanged	0	0	0	0	0	0	0
Missing	0	0	0	0	0	0	0
**Lactic dehydrogenase (U/L)**							0
Up	1	2	2	1	3	5	14
Down	1	0	3	2	0	1	7
Unchanged	0	0	0	0	0	0	0
Missing	1	1	1	0	0	0	3
**Cholinesterase (U/L)**							0
Up	2	1	5	3	3	4	18
Down	1	1	0	0	0	2	4
Unchanged	0	0	0	0	0	0	0
Missing	0	1	1	0	0	0	2
**Total bile acid (μmol/L)**							0
Up	3	1	2	2	2	3	13
Down	0	1	3	1	1	3	9
Unchanged	0	0	0	0	0	0	0
Missing	0	1	1	0	0	0	2
**Prothrombin activity (%)**							0
Up	1	2	2	3	1	5	14
Down	2	1	4	0	2	1	10
Unchanged	0	0	0	0	0	0	0
Missing	0	0	0	0	0	0	0

To assess the impact of MSC therapy on patients’ nutritional status and liver synthetic function, we analyzed changes in albumin, prealbumin, and cholinesterase levels and in prothrombin activity. Albumin levels improved in 53.3% (*n* = 8) of Phase Ia participants and 88.9% (*n* = 8) of Phase Ib participants. Prealbumin levels increased in 53.3% (*n* = 8) of the Phase Ia participants and 66.7% (*n* = 6) of the Phase Ib participants. Cholinesterase levels increased in 73.3% (*n* = 11) of Phase Ia and 77.8% (*n* = 7) of Phase Ib participants, whereas prothrombin activity improved in 53.3% (*n* = 8) of Phase Ia and 66.7% (*n* = 6) of Phase Ib participants.

For quality-of-life assessments, chronic liver disease questionnaire (CLDQ) scores improved in 55.6% (*n* = 5) of Phase Ia participants and 88.9% (*n* = 8) of Phase Ib participants, with all Cohort B participants showing improvements. The EuroQol 5-dimension self-report questionnaire (EQ-5D) scores improved in 20% (*n* = 3) of Phase Ia participants and 55.6% (*n* = 5) of Phase Ib participants, with Cohort B accounting for 66.7% (*n* = 4) of these improvements. These results suggest that higher or multiple doses may improve quality of life. (Table 3)

Regarding cirrhosis complications within 28 days after MSC treatment, no cases of liver failure, hepatocellular carcinoma, or death occurred in any cohort.

### Dynamic single-cell landscape of PBMCs following MSC treatment

**Fig. 3:**
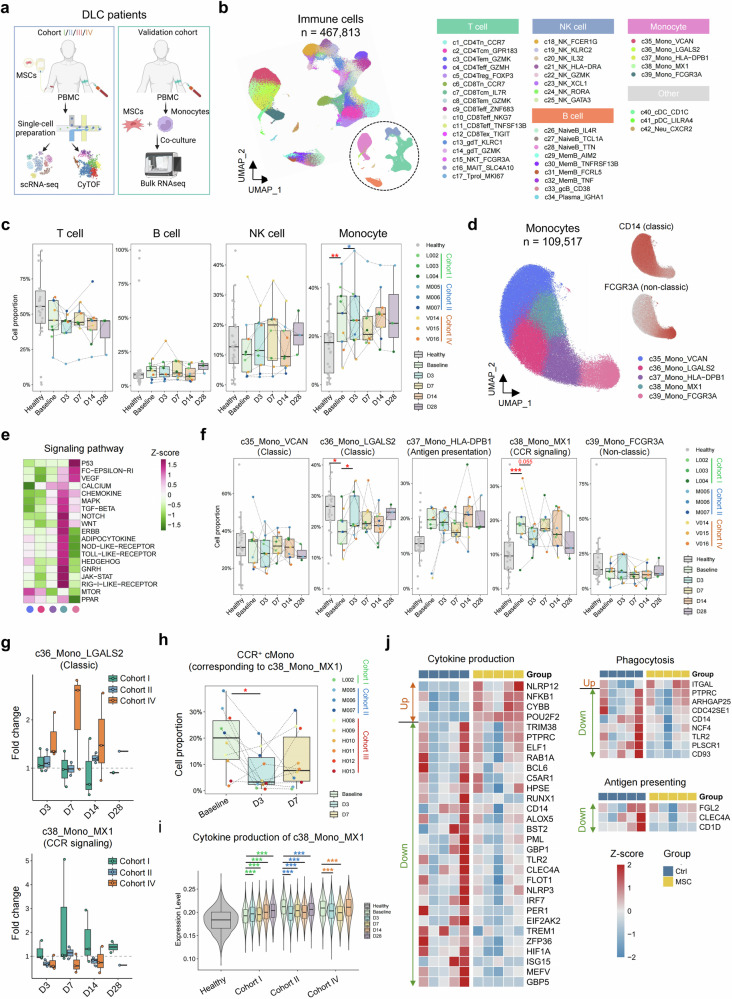
The dynamic single-cell landscape of DLC patients under MSC treatment. **a** Schematic diagram of the multiomics analysis of cohorts in the clinical trial and the validation cohort. **b** Uniform manifold approximation and projection (UMAP) plots of the annotated cell lineages (left) and subpopulations (right) at a relatively high resolution. The cell clusters were annotated via canonical markers. **c** Boxplot showing the dynamics of the four major immune cell lineages. Lineage proportions were calculated as the percentage of total immune cells. **d** UMAP plot of the annotated monocyte subpopulations (left) and the expression of *CD14* and *FCGR3A* (right). **e** Heatmap showing the scaled signature scores of the KEGG signaling pathways across monocyte subpopulations. The signature scores were calculated via the “AddModuleScore” function in Seurat. **f** Boxplot showing the dynamics of the five monocyte subpopulations. Cell proportions were calculated as the percentage of total monocytes. **g** Boxplot showing the fold change in cell proportions of c36_Mono_LGALS2 and c38_mono_MX1 at different MSC treatment time points compared with baseline across the three cohorts of DLC patients. **h** Boxplot showing the dynamics of the CCR^+^ cMono proportion in CyTOF. **i** Violin plot showing the signature score of the cytokine production process in c38_mono_MX1. Related genes were derived from the Gene Ontology database. **j** Heatmap of bulk RNA-seq data showing the expression of MX1^+^ monocyte-specific genes before and after coculture with MSCs. The Wilcoxon rank-sum test was used to test the difference in cell proportion between groups (*p*-values highlighted in red) and between posttreatment and baseline in each cohort (*p*-values highlighted in the corresponding cohort color). ***p < 0.001. **p < 0.01. *p < 0.05

We performed single-cell RNA sequencing (scRNA-seq) on peripheral blood mononuclear cell (PBMC) samples from three patients in Cohorts I, II, and IV at five time points (baseline, Day 3 [D3], Day 7 [D7], Day 14 [D14], and Day 28 [D28], Fig. 3a). Moreover, we included 28 PBMC samples from healthy donors in the public dataset to identify alterations in CAID patients at baseline. After rigorous quality control and batch effect removal, we obtained 467,831 single cells (Fig. 3b). Following dimensionality reduction and annotation on the basis of canonical markers, we identified four main immune cell lineages: T cells, B cells, NK cells, monocytes, and other myeloid immune cells (Fig. 3b, Supplementary Table 3). These lineages were further subdivided at higher resolution. We subsequently calculated the proportion of cell lineage alterations at baseline and their dynamics following MSC treatment. At baseline, the proportions of monocytes and B cells were elevated. In contrast, the proportions of T cells and NK cells were reduced (Fig. 3c). After MSC treatment, the proportion of these cell lineages gradually shifted toward the healthy state compared with the baseline state, with the most notable recovery observed on D7. To validate the cellular dynamics of immune cell lineages under MSC treatment, we also performed cytometry by time-of-flight (CyTOF) staining on PBMC samples from 10 patients (one patient in Cohort I, three patients in Cohort II, and six patients in Cohort III) at three time points (baseline, D3, and D7). The cells were categorized into seven clusters corresponding to the scRNA-seq data (Supplementary Fig. 1a). T cells and NK cells also tended to increase with MSC treatment, while B cells tended to decrease (Supplementary Fig. 1b). Interestingly, monocytes tended to increase on D3 in most patients but decreased on D7. Considering that monocytes are diverse among PBMCs, we hypothesized that various monocyte subpopulations underwent distinct changes during MSC treatment. These results indicate that monocytes had the most significant changes in DLC patients compared with healthy individuals among all the immune cell subsets. Moreover, MSC treatment had a relatively minor effect on the proportions of immune cells other than monocytes. This, to some extent, indicates that changes in monocytes may be the primary mechanism through which MSCs exert their immunomodulatory functions. Furthermore, the impaired proportion and function of monocytes are closely associated with cirrhosis progression.^4,26^ The important role of monocytes and the varying trends observed in different cohorts prompted us to analyze monocyte subpopulations.

### Alterations in myxovirus resistance 1-positive (MX1^+^) monocytes in response to MSC treatment

To elucidate the changes in monocyte subpopulations during MSC treatment, we analyzed five monocyte subpopulations (Fig. 3d). Among them, versican^+^ (VCAN^+^, c35) monocytes, galectin 2^+^ (LGALS2^+^, c36) monocytes, and myxovirus resistance 1-positive (MX1^+^, c38) monocytes expressed more CD14 and fewer Fc gamma receptor IIIa (FCGR3A, CD16), corresponding to classical monocytes. Moreover, MX1^+^ monocytes highly expressed IFN-stimulated genes (Supplementary Table 4). MHC class II molecules DP beta^+^ (HLA-DPB^+^, c37) monocytes expressed both FCGR3A and CD14 (transitional monocytes), whereas FCGR3A^+^ (c39) monocytes expressed high levels of FCGR3A (CD16) and low levels of CD14, corresponding to nonclassical monocytes. KEGG pathway analysis revealed that the MX1^+^ monocytes were more immunologically active, with high expression of genes related to the chemokine receptor (CCR), mitogen-activated protein kinase (MAPK), and transforming growth factor-beta (TGF-β) pathways (Fig. 3e). Further analysis of cell proportions revealed that the number of MX1^+^ monocytes was significantly greater at baseline than it was in healthy controls, whereas the number of LGALS2^+^ monocytes was notably lower (Fig. 3f). After undergoing MSC treatment, both subpopulations tended toward healthy controls; however, these changes varied across cohorts: MSCs effectively reduced the number of MX1^+^ monocytes and increased the number of LGALS2^+^ monocytes, with the most pronounced effects on D7 in Cohort IV. In contrast, MSCs demonstrated moderate efficacy in Cohort II but failed to significantly influence these changes in Cohort I (Fig. 3g). In the CyTOF data, we also identified a subpopulation with high expression of CCR4 and CCR6, corresponding to MX1^+^ monocytes (Supplementary Fig. 1c). The proportion of this subpopulation also decreased on D3 and D7 (Fig. 3h).

Pseudotime analysis via Monocle3 and validation via Slingshot indicated that VCAN^+^ monocytes represent the root of differentiation, with FCGR3A^+^ monocytes serving as the terminal state (Supplementary Fig. 1d). As differentiation progresses, cells increasingly express MHC class II molecules, including HLA-DRA, HLA-DRB1, HLA-DQA1, HLA-DPA1, and HLA-DMA (Supplementary Fig. 1e). At baseline, the FCGR3A^+^ monocytes exhibited an earlier phenotype compared to the healthy state (Supplementary Fig. 1f). MSC treatment could trigger MSC differentiation toward the terminal phenotype, especially in Cohort IV.

To further explore the functional changes in monocyte subpopulations, we analyzed the potential differentiation process of monocyte subpopulations. Cytokine production decreased in MX1^+^ monocytes following MSC treatment, and this downward trend was more pronounced in Cohort IV, with the strongest effect observed on D7 (Fig. 3i). Next, we analyzed which cytokines were influenced by MSC treatment. The production of proinflammatory cytokines, interleukin-15 (IL15), and macrophage stimulating 1 (MST1) in MX1^+^ monocytes, which were elevated in DLC patients at baseline, were decreased by MSC treatment. However, MSCs further increased the expression of tumor necrosis factor (TNF) superfamily members (TNFSF10, TNFSF13, and TNFSF14), which are known to stimulate T-cell activation (Supplementary Fig. 1g).

To validate the function of MX1^+^ monocytes, we isolated CD14⁺ monocytes from the peripheral blood of an independent cohort of five DLC patients and cocultured them with MSCs, followed by bulk RNA-seq analysis. Using scRNA-seq as a reference, we examined the functional changes in MX1⁺ monocytes before and after coculture. The results revealed that, after coculture with MSCs, the expression of genes related to cytokine production, phagocytosis, and antigen presentation in MX1⁺ monocytes were reduced, which confirms the immunomodulatory effect of MSCs on MX1⁺ monocytes (Fig. 3j). To assess whether MX1⁺ monocytes are associated with clinical outcomes, we categorized patients from Cohorts I, II, and IV into four groups based on changes in their Child‒Pugh scores (i.e., +1/+2, 0, −1, and −2) and analyzed the changes in the proportion of MX1⁺ monocytes (Supplementary Fig. 1h). The results showed that in the “+1/+2” group, the proportion of MX1⁺ monocytes increased to above baseline by D7, while in the “0” group, the proportion increased above baseline by D14. In contrast, patients in the “−1” group showed a decrease below baseline on D3, while the proportions on D7 and D14 were comparable to those at baseline. In contrast, the “−1” group showed little difference between D7 and D14 compared to baseline. More importantly, in the “−2” group, the proportion of MX1⁺ monocytes remained consistently below baseline at all four posttreatment time points. These findings suggest that the proportion of MX1⁺ monocytes is associated with clinical outcomes.

These results suggest that MSC treatment has varying effects on different monocyte subpopulations. The modulatory effect of MSC treatment on the highly immune-active c38 subpopulation is dose-dependent, indicating that MX1^+^ monocytes may be a key monocyte subpopulation that responds to MSC therapy. Moreover, changes in the cytokine secretion function of MX1^+^ monocytes suggest that it may influence the function of other immune cells.

### MX1^+^ monocytes mediated the immunomodulatory function of MSCs

**Fig. 4:**
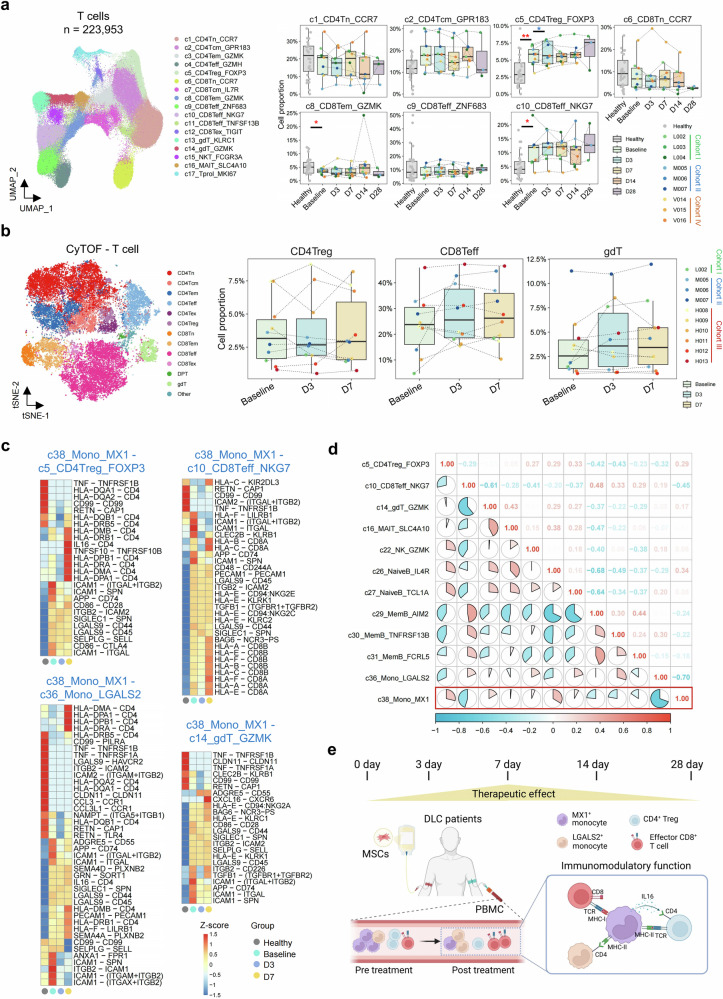
The variation in other immune cells and their interactions with monocytes in DLC patients under MSC treatment. **a** UMAP plot of the annotated T-cell subpopulations (left) and boxplots showing the dynamics of the major T-cell subpopulations (right). Cell proportions were calculated as the percentage of total T cells. Subpopulations with a maximum median proportion across groups over 5% are displayed. **b** t-SNE plot of the annotated T-cell subpopulations in CyTOF (left) and boxplot showing the dynamics of the three T-cell subpopulations in CyTOF (right). **c** Heatmaps showing the strength of ligand‒receptor pairs between c38_Mono_MX1 and four other immune cell subpopulations in four groups: healthy, baseline, D3, and D7. **d** Pie chart illustrating the correlations among cell subpopulations and indicating the differences between DLC patients at baseline and healthy individuals. The corresponding Spearman correlation coefficients are shown in the top-right corner. **e** Scheme showing the optimal efficacy timing of MSC treatment, its impact on immune cell subpopulations, and the key regulatory role of MX1^+^ monocytes. The Wilcoxon rank-sum test was used to test the difference in cell proportion between groups (*p*-values highlighted in red) and between posttreatment and baseline in each cohort (*p*-values highlighted in the corresponding cohort color). ***p < 0.001. **p < 0.01. *p < 0.05

The observed variations in cytokine production indicate the immunomodulatory function of MX1^+^ monocytes. To identify how MX1^+^ monocytes influence other immune cells, we first analyzed other immune cell subpopulations with relatively higher proportions in DLC patients, which differed from those in healthy individuals, and detailed their changes following MSC treatment. All 223,953T cells were clustered into 17 subpopulations (Fig. 4a, Supplementary Table 5). Both regulatory CD4^+^ T cells (c5_CD4Treg_FOXP3) and effector CD8^+^ T cells (c10_CD8Teff_NKG7) were elevated at baseline. Following MSC treatment, CD4Treg exhibited a gradual decrease trend on D3 and D7. For CD8Teff cells, MSC treatment did not directly inhibit the further increase in the proportions of CD8Teff cells. In CyTOF, the corresponding subpopulations of CD4Treg and CD8Teff cells were also identified (Fig. 4b). Most of the patients whose CD4Treg proportions increased on D3 were in the high-dose group. CD8Teff cells remained stable or slightly increased in most patients, which is consistent with the trend observed via scRNA-seq. Among the eight indicated NK cell subpopulations, c22_NK_GZMK was identified as a CD56^bright^ NK cell subset (Supplementary Fig. 2a, Supplementary Table 6). Compared with healthy individuals, this subpopulation presented an increased proportion at baseline. Interestingly, the proportion of this subpopulation varied across different patients: a decreasing trend was observed in Cohort I, whereas an increasing trend was observed in some patients from Cohorts II, III, and IV (Supplementary Fig. 2b). For 9 B-cell subpopulations, two naïve subpopulations (c26_NaiveB_IL4R and c27_NaiveB_TCL1A) and germinal center B cells (c33_gcB_CD38) increased at baseline, whereas three memory subpopulations (c29_MemB_AIM2, c30_MemB_TNFRSF13B, and c31_MemB_FCRL5) decreased (Supplementary Fig. 2c, Supplementary Table 7). Following MSC treatment, the number of naïve B cells remained stable and gradually decreased on D3 or D7 (Supplementary Fig. 2c, d).

We then explored cell–cell communications between altered immune cell subpopulations under MSC treatment. Both the outgoing and incoming signals of each subpopulation were elevated at baseline compared with those of healthy controls and decreased following MSC treatment (Supplementary Fig. 3a). MX1^+^ monocytes predominantly act as signal emitters, while CD8Teff receives the most signals. Among the signals emitted by MX1^+^ monocytes, two pathways changed with MSC treatment: galectin was elevated in DLC patients but decreased on D7 in Cohorts II and IV. On the other hand, resistin was reduced in patients but increased on D3 and D7 in Cohort IV (Supplementary Fig. 3b, c). We also analyzed the variation in ligand‒receptor pairs between altered subpopulations (Fig. 4c, Supplementary Fig. 3d). At baseline, MX1^+^ monocytes promote the transendothelial migration of CD4Treg, CD8Teff, γδ T, and LGALS2^+^ monocytes via intercellular cell adhesion molecule-1 (ICAM1). Additionally, MX1^+^ monocytes interacted with other subpopulations through the amyloid protein precursor (APP)-CD74 axis. The inhibitory function of CD4Treg cells was strengthened by MX1^+^ monocytes via the CD86-cytotoxic T-lymphocyte-associated protein 4 (CTLA4) interaction, while the chemotaxis and phagocytosis of LGALS2^+^ monocytes were enhanced by Annexin A1 (ANXA1) produced by MX1^+^ monocytes. Following MSC treatment at D3 and D7, the chemotactic activity of MX1^+^ monocytes was reduced, whereas their antigen-presentation capacity was enhanced. Furthermore, MX1^+^ monocytes can modulate CD4Treg function via the IL16-CD4 interaction. To further investigate the immunomodulatory role of MX1^+^ monocytes, we analyzed their correlations with other altered subpopulations (Fig. 4d). We found that MX1^+^ monocytes were positively correlated with CD4Treg, γδ T, and naïve B cells but negatively correlated with CD8Teff, LGALS2^+^ monocytes, and memory B cells. Based on the identified cell proportion changes and interactions among cell subpopulations, the regulatory effects of MSCs on immune cells can be illustrated with a schematic diagram (Fig. 4e).

## Discussion

This Phase I clinical trial represents a pioneering effort to evaluate the safety, tolerability, and immunological effects of varying MSC doses in DLC while exploring preliminary signals to guide future efficacy-focused studies. By addressing the dose‒effect relationship of MSCss and their immunomodulatory potential, this study highlights how MSCs regulate CAID, a key factor influencing DLC outcomes.^5^ Using multiomics approaches, we analyzed Phase Ia data and demonstrated that higher MSC doses elicited stronger immunomodulatory effects, reflected in the significant modulation of immune cell subsets critical to CAID pathogenesis. These effects were sustained for 7 days posttreatment but diminished by Day 14, emphasizing the importance of optimizing the dose and treatment intervals. Our findings provide the first human-based evidence of the dose‒response relationship of MSC therapy, identify MX1⁺ monocytes as key immune mediators, and offer critical mechanistic insights that support the design of Phase II–III clinical trials in DLC patients.

Owing to the complex clinical profile of DLC, precise optimization of MSC dosing, timing, and duration is necessary to maximize therapeutic efficacy. Clinical studies have demonstrated the therapeutic potential of MSCs in cirrhosis, with documented improvements in fibrosis scores, mortality reduction, MELD score enhancement, and survival prolongation.^27–32^ However, the existing evidence remains contradictory, as some trials report no significant clinical benefits and even indicate possible profibrotic effects.^20,33,34^ The variability in clinical outcomes across studies likely reflects differences in dosing regimens and intervals.^13,17,18,21^ These discrepancies underscore the critical need for evidence-based dose optimization in MSC therapy. Currently, MSC dosing for DLC relies more on clinical experience than on standardized, evidence-based protocols. Few trials have used a dose-escalation approach to evaluate MSC dose‒response in DLC.^21^ Thus, a deeper understanding of the mechanisms by which MSCs treat DLC and CAID in the human body is necessary to enable effective clinical protocols. To address these gaps, we employed a “3 + 3” dose-escalation design, which allowed for systematic dose increases while ensuring safety. Previous studies reported positive outcomes in inflammatory diseases with MSC doses ranging from 1 to 2 × 10^6^ cells/kg.^12,35–37^ For liver disease, doses generally range from 5 × 10^5^ to 1 × 10^6^ cells/kg.^18^ On the basis of this evidence, we selected doses of 5.0 × 10^7^ to 2.0 × 10^8^ cells for Phase Ia, corresponding to 7 × 10^5^ to 3 × 10^6^ cells/kg for a 70 kg patient (estimated average weight). This approach enables the precise assessment of the dose‒effect relationship and informed dosing for subsequent trials.

Our study confirms that MSC therapy is well tolerated across a range of doses, with no SAEs, DLTs, or SUSARs observed in any cohort. The majority of AEs were mild (Grade 1) or moderate (Grade 2), with only one Grade 3 upper respiratory tract infection reported, likely due to the underlying immune dysfunction of DLC patients rather than MSC treatment.^38^ Importantly, even at the highest dose tested (2.0 × 10^8^ cells, three doses), MSC therapy demonstrated a safe profile, supporting its feasibility for use in patients with severe liver dysfunction. This provides a strong foundation for advancing larger trials with more robust designs to validate these findings.

Previous studies have demonstrated the immunomodulatory effects of MSCs on both innate and adaptive immunity.^4,9,39^ For example, MSCs can induce CSF-1R⁺ monocytes that produce IL-10^40^ and modulate monocyte-driven inflammation via the Pinch–Cxcl12–Mbl2 axis^41^ in mouse models. Additionally, MSCs promote early Ly6C⁺ monocyte recruitment and later macrophage remodeling through CD14/TLR4/NF-κB signaling.^42^ These findings underscore the central role of monocytes in mediating MSC-driven immunomodulation and repair. However, immune cell responses may vary across different stages of CAID. Notably, monocytes exhibit elevated HLA-DR expression in early-stage DLC but reduced expression during liver failure.^43,44^ This difference suggests that immune cells play various roles in CAID.^45,46^ Our study provides the first comprehensive single-cell landscape of immune cell alterations in DLC patients treated with MSC, utilizing scRNA-seq and CyTOF. This detailed analysis highlights the broad immunomodulatory potential of MSCs, which influence multiple immune cell subsets, especially monocytes, in the circulatory system of DLC patients. Monocytes constitute a crucial immune cell type whose varied proportions and impaired functions are responsible for infection susceptibility and adverse outcomes.^4,26^ According to our data and previous studies, the number of monocytes with high expression of HLA-DR and CD14 (HLA-DPB1^+^ monocytes) is elevated in DLC patients,^43^ and MSC treatment can reduce the proportion of this monocyte subset. Monocytes in blood are first activated by DAMPs, PAMPs, and cytokines and highly express HLA-DR to induce excessive systemic inflammation. After receiving anti-inflammatory mediators from the liver, monocytes in blood undergo functional reprogramming, exhibit decreased HLA-DR, and exhibit immunosuppressive functions.^47^ Therefore, both proinflammatory (HLA-DR^high^) and anti-inflammatory (HLA-DR^low^) monocytes may coexist in the blood of DLC patients. Moreover, a previous study revealed a decrease in HLA-DR in the classical monocytes of DLC and ACLF patients.^48^ However, another study revealed that the antigen presentation process was more common in classical monocytes from nonrecovered ACLF patients than in those from recovered ACLF patients.^49^ The two different outcomes may be associated with distinct subsets of monocytes. Our single-cell-level study can further elucidate these issues. MSCs reduce inflammation by suppressing HLA-DR^high^ monocytes.^50^ Conversely, they increase HLA-DR in nonclassical monocytes to support T-cell immunity.^9^

For other immune cells, while cytotoxic CD8^+^ T cells and NK cells were already elevated at baseline, MSC treatment further increased their levels. Previous studies have shown that patients with DLC exhibit impaired T-cell responses, with high expression of exhausted molecules programmed cell death protein-1 (PD-1) and T-cell immunoglobulin and mucin domain-containing protein 3 (TIM-3),^46^ as well as reduced cytolytic activity for circulating NK cells.^51^ Therefore, the MSC-induced increase in CD8^+^ T-cell and NK cell cytotoxicity could enhance the clearance of aberrant cells, suggesting improved immune homeostasis.

A key contribution of this study is the establishment of a dose‒response relationship for MSC therapy. Higher doses of MSCs elicited stronger and more sustained immunomodulatory effects, as evidenced by the modulation of monocyte subsets. MX1^+^ monocytes, a monocyte population characterized by high expression of interferon-stimulated genes and CCRs, emerged as a dose-dependent mediator of MSC-induced immunomodulation. Notably, changes in MX1^+^ monocyte proportions and functional activity were most pronounced at higher doses, particularly at D7 posttreatment, but diminished at D14. This highlights the importance of dose optimization and interval refinement to sustain therapeutic effects. These findings offer valuable insights into how MSC therapy restores immune homeostasis by modulating innate immunity. Previous studies have demonstrated that peripheral blood monocytes can serve as biomarkers for various diseases, such as fibrotic diseases,^52^ multiple myeloma,^53^ and autoimmune encephalitis.^54^ In our studies, the change in the proportion of MX1⁺ monocytes was consistent with the change in the Child‒Pugh score—patients who showed a greater decrease in score after treatment maintained a lower level of MX1⁺ monocytes. These findings indicate that MX1^+^ monocytes not only represent a key therapeutic target but also serve as potential biomarkers for monitoring MSC treatment response. The identification of this monocyte subset advances our understanding of the mechanistic basis of MSC therapy and underscores its translational potential for DLC and other immune-dysregulated conditions.

This study has several limitations. The small sample size, inherent to phase I trials, limits the statistical power and generalizability of the results. While clinical efficacy remained exploratory, multiomics analyses (scRNA-seq and CyTOF) applied rigorous statistics. To validate immune cell dynamics in small cohorts, we compared changes in cell proportions across time points and groups via one-way ANOVA, paired *t*-tests, and linear mixed models. Cross-modal consistency minimized platform bias and supported MX1⁺ monocytes as a key MSC-responsive subset. Furthermore, long-term outcomes remain to be evaluated. As outlined in the study protocol, we will extend follow-up to 2 years to assess the durability of the clinical and immunological responses comprehensively. Despite these limitations, the integration of high-dimensional multiomics has provided robust mechanistic insights and laid the foundation for future trials.

In conclusion, this Phase 1 clinical trial demonstrated the safety, tolerability, and potential therapeutic signals of MSC therapy in DLC treatment while offering insights into its immunomodulatory effects. The conducted multiomics analyses revealed, for the first time, the dose‒effect relationship and optimal administration intervals for MSC therapy in DLC. The identification of MX1^+^ monocytes as a critical subset mediating MSC-induced immunomodulation offers novel mechanistic insights into MSC therapy in CAID. Notably, MX1⁺ monocytes may serve as a predictive biomarker for assessing MSC therapeutic response in future trials, potentially guiding patient stratification and treatment optimization. These results not only deepen our understanding of the immunological mechanisms underlying MSC therapy but also provide a critical foundation for designing future trials to optimize MSC-based treatment strategies for DLC patients. Well-designed phase II clinical trials with larger sample sizes are warranted to validate the safety and efficacy of MSC therapy, ultimately advancing its clinical translation.

## Materials and methods

### Ethical statement

The study was approved by the Ethics Review Board of the Chinese PLA General Hospital (Beijing, China) and conducted in accordance with the principles of the Declaration of Helsinki and Good Clinical Practice guidelines. Written informed consent was obtained from all participating patients. The trials were registered on ClinicalTrials.gov (NCT05227846 and NCT05984303). The clinical protocol and statistical analysis plan are available in Supplementary Files 1 and 2.

### Study design

This open-label, sequential single-arm, dose-escalation Phase Ia/Ib clinical trial involved both single and multiple administrations of MSCs according to the “3 + 3” rule. The sample size was based on a Phase I dose-escalation design, which prioritizes safety evaluation while minimizing patient risk in accordance with regulatory guidelines. In Phase Ia, patients were assigned to one of four cohorts, each receiving a single dose of MSCs: Cohort I received 5.0 × 10^7^ cells, Cohort II received 1.0 × 10^8^ cells, Cohort III received 1.5 × 10^8^ cells, and Cohort IV received 2.0 × 10^8^ cells. Based on the data from Phase Ia, which assessed safety, efficacy, and the immunomodulatory effects and duration of different MSC doses, patients in Phase Ib received three doses of MSCs, administered one week apart. In Phase Ib, Cohort A received 1.0 × 10^8^ cells per dose, while Cohort B received 2.0 × 10^8^ cells per dose. Dose escalation proceeded cautiously from the lower to the higher dose, contingent on the absence of safety concerns within each cohort. Follow-up assessments were conducted at baseline; on Days 3 (D3), 7 (D7), 14 (D14), and 28 (D28) in Phase Ia; and on Days 7 (D7), 14 (D14), 21 (D21), and 28 (D28) in Phase Ib.

### Participants

Phase Ia patients were recruited at the Fifth Medical Center of the Chinese PLA General Hospital between March 22, 2022, and July 5, 2023. After the data from Phase Ia were evaluated, recruitment for Phase Ib continued from August 22, 2023, to March 22, 2024. The details of the inclusion and exclusion criteria are outlined in the protocol. In brief, patients aged 18–75 years with a Child‒Pugh score of 7–12 and a diagnosis of DLC were included. The diagnoses were based on clinical presentation, laboratory tests, imaging findings, and/or representative pathological results. Additionally, patients must have presented with at least one severe complication, such as hepatic encephalopathy, upper gastrointestinal bleeding from esophageal or gastric varices, spontaneous bacterial peritonitis, or ascites. To prevent interference from potential recompensation, the study excluded patients who were receiving antiviral therapy for HBV infection for less than 12 months, had undergone TIPS insertion within the past 6 months, or were receiving corticosteroid therapy for autoimmune cirrhosis for less than 6 months.

To validate the immunological mechanisms identified in the above-mentioned cohorts, we recruited five DLC patients who met the inclusion and exclusion criteria as an independent validation cohort. These patients did not receive MSC treatment. Instead, peripheral blood was collected, and monocytes were isolated and cocultured with MSCs in vitro. The phenotypic and functional changes in monocytes after coculture were subsequently analyzed.

### Procedures

The first three patients were assigned to receive low-dose MSCs. Each patient was monitored for three days to determine if they exhibited signs of DLT before the next patient in the same cohort received the same treatment. The last patient in each cohort was observed for seven days before the subsequent cohort, which received a higher dose, could be treated. Once the first three patients in a cohort were observed, if one of the three patients showed signs of DLT, the next three patients in that cohort received the same dose. If two or more patients in any cohort exhibited signs of DLT, the study would have been terminated. The study progressed to the next cohort only if none of the first three patients showed signs of DLT after the specified observation periods or if only one out of six patients in a cohort showed signs of DLT. The maximum tolerated dose (MTD) was defined as the highest dose that induced DLT in no more than one patient among the extended cohort of six patients.

Human umbilical cord MSCs (VUM02, 5 × 10^7^ cells/10 mL/bag) were prepared by Wuhan Optics Valley Vcanbiopharma Co., Ltd., China, following the methods described in our previous study. These cells were required to meet the identification standards for MSCs established by the International Society for Cell Therapy (ISCT) in 2006. The final cell preparation, supplemented with a cryoprotective agent, was stored in liquid nitrogen. The cryopreserved MSCs were then shipped frozen to the hospital using a validated liquid nitrogen shipper equipped with a continuous temperature monitoring device. Upon receipt, the cellular product was inspected and stored in a controlled, continuously monitored liquid nitrogen tank. Prior to infusion, trypan blue staining and flow cytometry were used to assess cell viability and perform phenotypic characterization of the MSCs (Supplementary Fig. 4). Cultures for bacteria, fungi, and Mycoplasma were conducted to confirm the absence of pathogens.

Before infusion, frozen MSC injections were rapidly thawed in a 37 °C water bath within one minute to achieve a light-yellow, ice-free solution. The vials were disinfected with 75% medical alcohol, and one to two bags of cells were processed at a time. For patients requiring multiple bags, subsequent bags were prepared immediately after the saline flush of the previous infusion. After electrocardiographic monitoring, patients received a slow intravenous infusion of MSCs via a peripheral vein, followed by a five-minute saline flush to ensure complete delivery. The patients were monitored for 30 min after infusion and placed under electrocardiographic surveillance. Their vital signs were recorded before, during, and after the procedure.

### Clinical endpoints

The primary safety outcome was the incidence of adverse events. Preliminary therapeutic signals included changes in the Child–Pugh score and MELD score, as well as indicators of liver function, such as albumin, prealbumin, bilirubin, cholinesterase, and prothrombin time. Additionally, quality of life was assessed via the EQ-5D score and the CLDQ score. Other outcomes included the incidence of complications associated with DLC, progression to liver failure, development of liver cancer, and liver transplant–free survival.

### Sample collection and processing

Peripheral blood samples for scRNA-seq were collected at baseline and at D3, D7, D14, and D28, whereas samples for CyTOF staining were collected at baseline and at D3 and D7. These samples were transported to the laboratory for processing within 12 h at room temperature or within 48 h if they were kept at approximately 4 °C. All the samples were analyzed fresh without cryopreservation. Peripheral blood mononuclear cells (PBMCs) were isolated using Ficoll density gradient centrifugation. The cell pellets were resuspended in 5 mL of precooled fluorescence-activated cell sorting (FACS) buffer (1× phosphate-buffered saline (PBS) supplemented with 0.5% bovine serum albumin), followed by centrifugation at 400 × *g* for 5 min at 4 °C. After the supernatant was discarded, the pellets were resuspended in FACS buffer. Cell counts were performed, and samples for subsequent analysis were required to meet the following criteria: a cell count of at least 3 × 10^6^ and a viability rate above 85%.

### Single-cell transcriptome preprocessing

Raw FASTQ files were mapped to the GRCh38 genome via Cell Ranger software (version 4.0.0) with default parameters. Following alignment, digital gene expression (DGE) matrices were generated. For each sample, a barcode file, a gene annotation file, and a raw count matrix file were generated. These data were then imported into R (version 4.2.1) with the Seurat package (version 4.2.0).^55^ Genes expressed in <10 cells and cells with <200 gene features or 1000 read counts were excluded from the count matrix. Cells with <10% mitochondrial genes were kept for further analysis. Then, the count matrix was normalized using the log-transformed method, and the top 2000 variable features were chosen for scaling and dimensionality reduction. To eliminate batch effects arising from samples collected at different time points and from different patients, we applied the FastMNN method to integrate all Seurat objects. To remove doublets, we applied the DoubletFinder package (version 2.0.3).^56^ For every increase of 1000 cells in the sample, the double-positive cell proportion increases by 0.8%. Doublets with high confidence were subsequently removed.

Dimensionality reduction was performed using principal component analysis (PCA). The first 30 principal components were selected for clustering, and the cell clusters were visualized using uniform manifold approximation and projection (UMAP). The functions FindNeighbors and FindCluster in Seurat were used to obtain the cell subtypes. Finally, subtypes were annotated based on marker genes to obtain immune cell subtypes.

Signaling pathways in the Kyoto Encyclopedia of Genes and Genomes (KEGG) were downloaded using the msigdbr package (version 7.5.1) to facilitate the signature score calculation of single cells. Then, the gene lists of these pathways were provided to calculate signature scores by the “AddModuleScore” function in Seurat with default parameters. The average signaling pathway score of each cell subset was calculated using the “AverageExpression” function in Seurat.

### Cell–cell interaction network analysis

CellChat (version 1.5.0) is a publicly available repository of curated receptors, ligands, and their interactions that can be used to search for cell–cell interactions and receptor–ligand pairs among cell types.^57^ Potential interactions between two cell types were inferred via the CellChat method on the basis of the expression levels of each receptor and ligand gene pair. Receptors and ligands expressed in more than 20% of the cells in the corresponding subclusters were included in the analysis. Various built-in functions of the CellChat package (with default parameters) were used for visualization.

### Pseudotime analysis

Pseudotime analysis was conducted with Monocle3 (version: 1.2.9) for developmental trajectory inference.^58^ The raw count matrix and metadata from the integrated Seurat object were passed to the monocle3 workflow to form a monocle3 cell dataset (cds) object. First, the cds object was preprocessed to prepare for trajectory inference via the “preprocess_cds” function. Then, the UMAP embedding calculated by the “reduce_dimension” function was replaced by the corresponding values stored in the Seurat object. The cells were subsequently clustered via the “cluster_cells” function, and the principal graph was learned from the reduced dimension space via the “learn_graph” function with default parameters. Genes whose expression changed over pseudotime were judged by genes with *q*_value = 0 and morans_I > 0.5, which was calculated via the graph_test function. The top 50 genes ordered by morans_I were used for Euclidean clustering and visualization via ComplexHeatmap (version 2.15.2). To confirm the trajectory inference results, we used Slingshot (version 0.3.3) to weigh the differentiation potential of monocyte subtypes.^59^ The raw count matrix from the Seurat object was input into the Slingshot workflow with default parameters. c35_Mono_VCAN was identified as the root cluster.

### CyTOF staining and data acquisition

The antibodies used in this study, along with their providers, clone numbers, and mass tags, are listed in Supplementary Table 8. Antibody labeling was performed via the Maxpar Antibody Conjugation Kit (Fluidigm), and the concentration of the mass-tagged antibodies was determined via a NanoDrop. The concentration of labeled antibodies was adjusted to 200 mg/mL via an antibody stabilizer buffer, and titration was performed to determine the optimal concentration. The obtained cells were washed with PBS and stained with 100 μL of 250 nM cisplatin (Fluidigm, South San Francisco, CA, USA) on ice for five minutes to exclude dead cells. The cells were then incubated in Fc receptor blocking solution before being stained with a cocktail of surface antibodies on ice for 30 min. After the samples were washed with PBS, unique barcode isotope combinations were used to label the individual samples for 30 min. The cells were washed twice with FACS buffer and fixed overnight in 200 μL of intercalation solution (Maxpar Fix and Perm Buffer containing 250 nM 191/193Ir, Fluidigm). The cells were subsequently washed with FACS buffer and Perm Buffer (eBioscience, San Diego, CA, USA) and stained with an intracellular antibody cocktail on ice for 30 min. After staining, the cells were washed and resuspended in deionized water, mixed with 20% EQ beads (Fluidigm), and analyzed via a Helios mass cytometer (Fluidigm). The CyTOF experiments were conducted by PLTTECH (Hangzhou, China). Signal strength adjustments were made for each channel on the basis of the same bead signals (140Ce, 151Eu, 153Eu, 165Ho, and 175Lu) before loading each batch. All samples were standardized to prevent batch effects before analysis.

### CyTOF data analysis

The raw data from each sample were debarcoded via a double-filtering scheme with unique mass-tagged barcodes. The .fcs files from different batches were normalized via the bead normalization method. FlowJo software (FlowJo, Ashland, OR, USA) was used for manual gating to exclude debris, dead cells, and doublets, leaving only live single immune cells. The X-shift clustering algorithm was applied to partition cells into distinct phenotypes on the basis of marker expression levels. Each cluster was then annotated according to its marker expression pattern and visualized on a heatmap. The t-distributed stochastic neighbor embedding (t-SNE) algorithm was used to analyze the high-dimensional data by reducing dimensionality and visualizing cluster distributions, marker expression, and differences between groups or sample types.

### Coculture of monocytes and MSCs followed by bulk RNA sequencing

Peripheral blood (10 mL) was collected from independent validation cohort patients, and plasma and peripheral blood mononuclear cells (PBMCs) were isolated. CD14⁺ monocytes were then purified from PBMCs via magnetic-activated cell sorting (MACS). The isolated monocytes were seeded into 0.4 μm Transwell inserts, which were placed into 12-well plates preseeded with MSCs at a 1:1 cell ratio. The coculture was maintained at 37 °C for 24 h. After incubation, monocytes were harvested, and total RNA was extracted for bulk RNA sequencing. Clean data were obtained by removing adapters and filtering out low-quality reads. The paired-end reads were aligned to the human genome (UCSC hg38) via HISAT2 (version 2.1.0). Gene annotation and read counting were performed with HTSeq (version 0.11.2). Protein-coding genes were selected, and normalized expression values were calculated via the DESeq2 package (version 1.32.0) on the R platform (version 4.1.0). MX1⁺ monocyte-specific genes were defined on the basis of two criteria: (1) expression in more than 30% of MX1⁺ monocytes and (2) an average log₂-fold change in expression between MX1⁺ monocytes and other cells greater than 0.2. Gene Ontology (GO) biological process terms were used to annotate the functions of the MX1⁺ monocyte-specific genes. Finally, genes that were differentially expressed after MSC coculture were analyzed within three functional categories: (1) cytokine production, (2) phagocytosis, and (3) antigen processing and presentation.

### Statistical analysis

Safety was assessed by analyzing adverse events within each cohort and noting their category and severity. Efficacy was evaluated according to the intention-to-treat (ITT) analysis principle. Continuous variables were summarized by mean ± standard deviation (SD), while categorical variables were summarized by frequency counts and percentages. The χ^2^ test or Fisher’s exact test was applied to compare categorical variables between different dose cohorts. These tests were also used to compare the occurrence rates of adverse events between cohorts for safety assessment. Changes in patients’ laboratory values within each cohort after treatment were analyzed to explore potential correlations with adverse events. Continuous variables were compared using the *t*-test or the Wilcoxon signed-rank test. The corresponding 95% confidence interval (95% CI) was provided. A detailed statistical analysis plan was formulated and finalized before the database lock, which dictated the specific methods and content of the study’s statistical analysis.

To analyze the changes in cell proportions across multiple cohorts and time points, we applied three statistical strategies: (1) comparing the changes in cell proportions between different time points for all patients, using one-way ANOVA followed by paired *t*-tests; (2) comparing the changes in cell proportions between different time points within each group of patients, conducting one-way ANOVA followed by paired *t*-tests for each group; (3) comparing the differences in cell proportions at each time point between different patient groups, using linear mixed models. *P*-values of cell proportion variation were provided in Supplementary Tables 9 and 10. A *p*-value of <0.05 was considered statistically significant. All clinical statistical analyses were performed using SAS 9.4 (SAS Institute, Cary, North Carolina, USA). Statistical analysis of the frequency of annotated cell populations was performed using Student’s *t*-test via the R platform (version 4.2.2).

## Supplementary information

Supplementary File 1: Clinical Study Protocol

Supplementary File 2: Statistical Analysis Plan (SAP)

Supplementary Materials

## Data Availability

After approval from the Human Genetic Resources Administration of China, these trial data can be shared with qualifying researchers who submit a proposal with a valuable research question. A contract should be signed. scRNA-seq, CyTOF, and Bulk RNA-seq data were deposited in the China National Center for Bioinformation under accession (https://www.cncb.ac.cn/) under the accession number PRJCA041746. The R scripts used for bioinformatics analysis are available at GitHub (https://github.com/SongMei233/DLC_patients).
