# Supplementary material for: Dose-escalation studies of mesenchymal stromal cell therapy for decompensated liver cirrhosis: phase Ia/Ib results and immune modulation insights
Source: Signal Transduct Target Ther. 2025 Jul 29;10:238. doi: 10.1038/s41392-025-02318-4 (PMC12304480; doi:10.1038/s41392-025-02318-4)
Supplement: Supplementary file 1 — Clinical Study Protocol [file 41392_2025_2318_MOESM1_ESM.pdf]

Supplementary File 1

Clinical Study Protocol

This appendix contains the following two clinical study protocols:

Clinical study	Page
An exploratory clinical study of the tolerability, safety and efficacy of human umbilical cord-derived mesenchymal stem cells for dose-escalation in patients with decompensated cirrhosis (MSC-DLC-1a)	2 ~ 23
A clinical study on the safety and efficacy of human umbilical cord-derived mesenchymal stem cells for treating cirrhosis in the decompensated stage (MSC-DLC-1b)	24 ~ 46

An exploratory clinical study of the tolerability, safety and efficacy of human umbilical cord-derived mesenchymal stem cells for dose-escalation in patients with decompensated cirrhosis (MSC-DLC-1a)

Protocol

Version 2.3 – March 22, 2023

Sponsor	The Fifth Medical Center of PLA General Hospital
Principal Investigator	Fu-Sheng Wang
Ethics Reference Number	2018-107-D-4
ClinicalTrials.gov	NCT05227846

Table of Content

1 TRIAL SUMMARY 5
2 INTRODUCTION 8
2.1 Background and rationale 8
2.2 Objectives 9
2.3 Trial design 9
3 METHODS 9
3.1 Study setting 9
3.2 Eligibility criteria 9
3.2.1 Inclusion criteria 9
3.2.2 Exclusion criteria 9
3.3 Interventions 10
3.3.1 Investigational medicinal product 11
3.3.2 Standard of care(SOC) 11
3.3.3 Intervention assignment 11
3.4 Outcomes 11
3.4.1 Primary outcomes 11
3.4.2 Secondary outcomes 12
3.5 Participant timeline 12
3.6 Sample size 15
3.7 Recruitment 15
3.8 Allocation and product management 15
3.8.1 Allocation 15
3.8.2 Dose escalation and dose-limiting toxicity 15
3.8.3 Assessment of DLT and MTD 16
3.8.4 Product management 17
3.9 Data collection and data management 17
3.10 Statistical considerations 17
3.10.1 Statistical methods 17
3.10.2 Analysis population 18
3.11 Safety/harms 18
3.12 Auditing 18
4 ETHICS AND DISSEMINATION 19

4.1 Research ethics approval ... 19
4.2 Protocol amendments ... 19
4.3 Informed consent process ... 19
4.4 Confidentiality ... 19
4.5 Declaration of interests ... 19
4.6 Access to data ... 20
4.7 Ancillary and post-trial care ... 20
4.8 Dissemination policy ... 20
5 STUDY ADMINISTRATION ... 20
5.1 Key contracts ... 20
5.2 Roles and responsibilities ... 20
5.2.1 Protocol contributors ... 20
5.2.2 Sponsor and funding and collaborators ... 21
6 REFERENCES ... 21

1 TRIAL SUMMARY

Title	An exploratory clinical study of the tolerability, safety and efficacy of human umbilical cord-derived mesenchymal stem cells for dose-escalation in patients with decompensated cirrhosis (MSC-DLC-1)
Primary registry and trial identifying number	ClinicalTrials.gov NCT05227846
Secondary identifying numbers	2018-107-D-4
Sources of monetary or material support	The Fifth Medical Center of PLA General Hospital
Primary sponsor	The Fifth Medical Center of PLA General Hospital
Central contract	Principal Investigator: Fu-Sheng Wang, MD, Ph.D.<br>86-10-66933332 fswang302@163.com<br>Investigator and coordinator: Lei Shi, MD, Ph.D.<br>86-10-66933333 shilei302@126.com<br>Study site:<br>The Fifth Medical Center of PLA General Hospital, Beijng, China., 100039
Study officials/investigators	Study Principal Investigator<br>Fu-Sheng Wang, MD, Ph.D.<br>The Fifth Medical Center of PLA General Hospital
Brief title	Human Umbilical Cord-derived Mesenchymal Stem Cells for Decompensated Cirrhosis (MSC-DLC-1)
Countries of recruitment	P. R. China
Condition(s) or focus of study	Decompensated cirrhosis
Interventions	Human umbilical cord-derived mesenchymal stem cells(UC-MSCs)
Key eligibility criteria	Inclusion criteria<br>1. Signed the informed consent form

	2. Aged 18–75 years old<br>3. Diagnosed as having decompensated cirrhosis based on clinical presentation, laboratory tests, imaging and/or representative pathological findings<br>4. Child–Pugh score of 7–12<br>Exclusion criteria<br>1. Had serious complications, such as hepatic encephalopathy, refractory ascites, hepatorenal syndrome or bleeding from esophageal varices within the last month<br>2. Had at least one uncontrolled severe infection within the past 2weeks<br>3. Had a hepatitis B virus DNA test result above the testing threshold<br>4. Has hepatitis B/C cirrhosis and has either not received antiviral treatment for more than 12 months or may stop antiviral treatment during the research period<br>5. Has autoimmune cirrhosis and has not been treated with glucocorticoids for more than 6months<br>6. Pregnant or currently breastfeeding<br>7. Currently drinks alcohol and refuses to stop drinking during the research period<br>8. Uses illegal psychoactive substances, has a history of substance abuse or has been diagnosed with other psychological disorders<br>9. Has participated in other clinical studies within the last 3months<br>10. Has had transjugular intrahepatic portosystemic shunt surgery less than 6months ago<br>11. Has severe jaundice (total serum bilirubin level $\geq$170μmol/L), has obvious deficiencies in kidney function (serum creatinine $\geq$1.2 times normal levels), has severe electrolyte abnormalities (serum sodium level $<$125mmol/L) and/or severe leucopenia (white cell count $<1\times10^9$/L)<br>12. Has combined biliary obstruction, hepatic, or portal, or splenic vein thrombosis, or portal vein cavernous lesions<br>13. Has had certain surgery, such as splenectomy or portal shunt, in

	the past<br>14. Has a confirmed or suspected malignant tumour(s)<br>15. Has a history of any major organ transplant or any severe systemic disease involving the major organs, such as the heart, kidneys, liver or the blood<br>16. Has received a blood transfusion within the last month<br>17. Has other circumstances or complications that the researchers deem unsuitable for the study<br>18. Has a positive HIV antibody test result<br>19. Has allergies or a history of severe allergies
Study design	Open-label, dose-escalation, single-armed phase I trial
Date of enrollment	March 22, 2023 (The first subject signed the informed consent form)
Target sample size	12~24
Recruitment status	Participants in all dose groups have completed 28 days of observation after the dose, and 2-year safety follow-up is ongoing. The trial finishing date is estimated as March 1, 2025
Primary outcomes	The primary outcomes are the incidence of adverse events and the change in the Model for End-stage Liver Disease (MELD) score from baseline to the 28th day after the intervention
Secondary outcomes	The secondary outcomes include:<br>1. Change in the MELD score from baseline to the score at 3days, 7days, 14 days, 3 months, 6 months, 9 months, 12 months, 15 months, 18 months, 21 months and 24 months after the intervention<br>2. Incidence of each complication associated with decompensated cirrhosis<br>3. Liver transplant-free survival rate<br>4. Incidence of liver failure<br>5. Plasma albumin (ALB) level<br>6. Plasma prealbumin (PALB) level

	7. Total bilirubin (TBIL)<br>8. Serum cholinesterase (CHE)<br>9. Prothrombin time (PT)<br>10. Child-Turcotte-Pugh(CTP) score<br>11. EuroQol Group 5-Dimension Self-Report Questionnaire (EQ-5D) scale score<br>12. Incidence of liver cancer<br>13. Chronic Liver Disease Questionnaire (CLDQ) score

2 INTRODUCTION

2.1 Background and rationale

Decompensated liver cirrhosis (DLC) is characterized by severely impaired liver function and other associated complications, such as portal hypertension, ascites, spontaneous peritonitis, coagulation dysfunction, gastrointestinal bleeding, hepatic encephalopathy, and hepatorenal syndrome [1]. Current available treatment strategies can help improve the patients' quality of life and partially reduce the complications of DLC, and include taking diuretics and following a low-sodium diet, but there are no efficient therapeutic regimens [2, 3]. Furthermore, DLC indicates that the patient is nearing the end stage of liver failure, and liver transplantation then becomes the best option [4]. However, liver transplantation carries considerable risks, since liver failure is frequently associated with liver immune disorders that may contribute to rejection of the new organ and a decrease in the patient's quality of life [2]. In the absence of a liver transplant, an alternative therapy that can effectively relieve the symptoms and promote recovery would be very helpful for patients.

Mesenchymal stem cell (MSC) therapies have emerged as a novel alternative for the treatment of end-stage liver diseases [5, 6]. MSCs have a variety of useful properties, such as the secretion of cytokines, growth factors, and hepatocytes, which can help improve liver functionality and regress fibrosis. Studies have also shown that MSCs can suppress the proliferation of hepatic stellate cells and induce their apoptosis [7-10]. Multiple studies on MSCs have demonstrated their potential for treating end-stage liver disease [11-17], but the appropriate dose and related mechanisms are still

unclear. In addition, no clinical research reports have yet explored escalating the dose of MSCs for treating DLC.

2.2 Objectives

We have designed an investigator-initiated, single-arm, dose-escalation clinical trial to evaluate the safety and identify the suitable dose of UC-MSCs as an infusion in patients with decompensated liver cirrhosis. This trial aims to provide evidence for evaluating the safety and efficacy of the UC-MSC infusion in future studies.

2.3 Trial design

This study was an open-label, dose-escalation, single-armed phase I trial (MSC-DLC-1) to evaluate the safety and efficacy of UC-MSCs for the treatment of patients with DLC. The study comprises three phases: screening + evaluation, 3 + 3 escalation, and follow up. Patients with DLC will be enrolled in the study. Each patient will be injected with one dose of a solution containing human UC-MSCs. After treatment, the patients will be followed up for 24 months.

3 METHODS

3.1 Study setting

The trial was conducted in The Fifth Medical Center of PLA General Hospital.

3.2 Eligibility criteria

3.2.1 Inclusion criteria

1. Signed the informed consent form
2. Aged 18–75 years old
3. Diagnosed as having decompensated cirrhosis based on clinical presentation, laboratory tests, imaging and/or representative pathological findings
4. Child–Pugh score of 7–12

3.2.2 Exclusion criteria

1. Had serious complications, such as hepatic encephalopathy, refractory ascites, hepatorenal syndrome or bleeding from esophageal varices within the last month
2. Had at least one uncontrolled severe infection within the past 2weeks
3. Had a hepatitis B virus DNA test result above the testing threshold
4. Has hepatitis B/C cirrhosis and has either not received antiviral treatment for more than 12 months or may stop antiviral treatment during the research period
5. Has autoimmune cirrhosis and has not been treated with glucocorticoids for more than 6months
6. Pregnant or currently breastfeeding
7. Currently drinks alcohol and refuses to stop drinking during the research period
8. Uses illegal psychoactive substances, has a history of substance abuse or has been diagnosed with other psychological disorders
9. Has participated in other clinical studies within the last 3months
10. Has had transjugular intrahepatic portosystemic shunt surgery less than 6months ago
11. Has severe jaundice (total serum bilirubin level ⩾170μmol/L), has obvious deficiencies in kidney function (serum creatinine ⩾1.2 times normal levels), has severe electrolyte abnormalities (serum sodium level <125mmol/L) and/or severe leucopenia (white cell count <1×109/L)
12. Has combined biliary obstruction, hepatic, or portal, or splenic vein thrombosis, or portal vein cavernous lesions
13. Has had certain surgery, such as splenectomy or portal shunt, in the past
14. Has a confirmed or suspected malignant tumour(s)
15. Has a history of any major organ transplant or any severe systemic disease involving the major organs, such as the heart, kidneys, liver or the blood
16. Has received a blood transfusion within the last month
17. Has other circumstances or complications that the researchers deem unsuitable for the study
18. Has a positive HIV antibody test result
19. Has allergies or a history of severe allergies

3.3 Interventions

3.3.1 Investigational medicinal product

The UC-MSCs (VUM02, 5×10^7 cells/10 mL/bag) will be prepared by Wuhan Optics Valley Vcanbiopharma Co., Ltd., in China. Briefly, mesenchymal stem cells (MSCs) will be obtained according to the method described in our previous study [18]. Umbilical cord tissue for sampling will be selected and collected after the mothers have signed the informed consent form. After the blood vessels are removed from the umbilical cord tissue, it will be cut into small pieces of about 1–2 mm^3, uniformly inoculated in cell culture flasks for isolation of the MSCs, and the primary cells will be digested with TrypLE digestive enzyme for 10–13 days. The primary cells will be inoculated in cell culture flasks at a density of about $2\times10^4/cm^2$ and cultured in an incubator at 37 °C with a volume fraction of 5% CO_2 and saturated humidity. After being passaged for P4 generation, the cells will be cryopreserved for use as the working cell bank. Subsequently, safety and biological performance tests will be conducted on the working cell bank. Following successful testing, the cells will undergo a resuscitation culture for 48 to 72 h to prepare P5 generation cell formulations. The cells must meet strict criteria to be used in our study. Specifically, the expression of cell immunophenotypes CD90, CD105, CD73 must be greater than 95%; the expression of CD45, CD34, CD14, CD19, and HLA-DR must be lower than 2%; the cells must test negative for sterility and mycoplasma; and the intracellular toxins must be less than 1 EU /mL. Only UC-MSCs that pass these tests will be used for the clinical trials.

3.3.2 Standard of care(SOC)

Provide optimal support and medication in accordance with relevant latest clinical guidelines.

3.3.3 Intervention assignment

This study is an open-label, dose-escalation, single-armed phase I trial. In this study, a total four dose groups (5.0×10^7 cells, 1.0×10^8 cells, 1.5×10^8 cells, 2.0×10^8 cells) are set up, dose escalation will be performed according to the "3+3" rule, each subject will be only entered into one corresponding dose group. See Section 3.8 for details.

3.4 Outcomes

3.4.1 Primary outcomes

The primary outcomes are the incidence of adverse events and the change in the Model for End-stage Liver Disease (MELD) score from baseline to the 28th day after the intervention.

3.4.2 Secondary outcomes

The secondary outcomes include:

1. Change in the MELD score from baseline to the score at 3 days, 7 days, 14 days, 3 months, 6 months, 9 months, 12 months, 5months, 18 months and 24 months after the intervention
2. Incidence of each complication associated with decompensated cirrhosis
3. Liver transplant-free survival
4. Incidence of liver failure
5. Plasma albumin (ALB) level
6. Plasma prealbumin (PALB) level
7. Total bilirubin (TBIL)
8. Serum cholinesterase (CHE)
9. Prothrombin time (PT)
10. Child-Turcotte-Pugh (CTP) score
11. EuroQol Group 5-Dimension Self-Report Questionnaire (EQ-5D) scale score
12. Incidence of liver cancer
13. Chronic Liver Disease Questionnaire (CLDQ) score

3.5 Participant timeline

Assessment schedule for the patients with decompensated cirrhosis

	Screening	Period of treatment and observation period					Long-term follow-up							
**Timepoint**	Day -21 to 0	Day 1 ±0 day	Day 3 ±0 day	Day 7 ± 0 day	Day 14 ± 1 day	Day 28 ± 3 days	Month 3	Month 6	Month 9	Month 12	Month 15	Month 18	Month 21	Month 24
							±7 days	±7 days	±7 days	±7 days	±7 days	±7 days	±7 days	±7 days
Enrollment and informed consent	×													
Screening table	×													
Demographic data	×													
Allergies and family history	×													
Past medical history and treatment history	×													
Existing disease and concomitant treatment	×													
Vital signs	×	×	×	×	×	×	×	×	×	×	×	×	×	×
Physical examination	×	×	×	×	×	×	×	×	×	×	×	×	×	×
Hepatitis B blood test/ HCV antibody	×							×		×		×		×
HIV/TP antibody	×							×		×		×		×
Blood routine test	×	×	×	×	×	×	×	×	×	×	×	×	×	×
Urine routine test	×	×			×	×	×	×	×	×	×	×	×	×
Urine pregnancy test	×		If necessary											
Stool routine test + occult blood	×	×			×	×	×	×	×	×	×	×	×	×
Blood biochemistry test	×	×	×	×	×	×	×	×	×	×	×	×	×	×
Coagulation function test	×	×	×	×	×	×	×	×	×	×	×	×	×	×
Blood ammonia	×	×	×	×	×	×	×	×	×	×	×	×	×	×
AFP	×	×			×	×	×	×	×	×	×	×	×	×
Electrocardiogram	×	×		×	×	×	×	×	×	×		×		×
Chest X-ray or CT	×									×				×
Abdominal ultrasound examination	×	×				×	×	×	×	×	×	×	×	×
Gastroscopy	×							×		×		×		×
Elastography	×						×	×	×	×	×	×	×	×
Upper abdominal CT/MRI plain scan + enhancement	×							×		×		×		×

MELD score	×	×	×	×	×	×	×	×	×	×	×	×	×	×
Child–Pugh score	×	×	×	×	×	×	×	×	×	×	×	×	×	×
QoL scale	×	×		×	×	×	×	×	×	×	×	×	×	×
Collection of samples for omics research (blood sample + stool)		×	×	×	×	×	×	×	×	×	×	×	×	×
Infusion records		×												
Adverse events	×	×	×	×	×	×	×	×	×	×	×	×	×	×
Concomitant medication/treatment	×	×	×	×	×	×	×	×	×	×	×	×	×	×

3.6 Sample size

This is a proof-of-concept study. A total of 3–6 patients will be recruited for each dose cohort, comprising low, medium, high and super high-dose groups, denoted as cohorts 1, 2, 3 and 4, with three patients in each cohort initially. The additional patients will be added to the cohort if DLT is presented. A total of 12–24 patients will be recruited for the study. [19].

3.7 Recruitment

Patient will be recruited from The Fifth Medical Center of PLA General Hospital. No other recruitment strategy (e.g., advertisement) will be adopted in this study.

3.8 Allocation and product management

3.8.1 Allocation

This study is an open-label, dose-escalation, single-armed phase I trial. In this study, a total of four dose groups (5.0×10^7 cells, 1.0×10^8 cells, 1.5×10^8 cells, 2.0×10^8 cells) are set up, dose escalation will be performed according to the "3+3" rule, each subject will be only entered into one corresponding dose group.

3.8.2 Dose escalation and dose-limiting toxicity

The single dose-escalation scheme, denoted as S-1, will be as follows 3+3: cohort 1 (5.0×10^7 cells), cohort 2 (1.0×10^8 cells), cohort 3 (1.5×10^8 cells) and cohort 4 (2.0×10^8 cells). Each patient will be observed for 3days for signs of dose-limiting toxicity (DLT) before another patient in the same cohort can receive treatment. The last patient of each cohort will be observed for 7days before the next cohort with a higher dose can start to receive treatment. The first three patients in a cohort will be observed to determine the next action. If one of the three patients shows signs of DLT, the next three patients in the cohort will be treated with the same dose. If two or more patients in any cohort show signs of DLT, then the study will be concluded. The study will only proceed to the next cohort if none of the first three patients shows signs of DLT after the outlined observation periods have elapsed or if only one out of the six patients in a cohort shows signs of DLT. The specific rules for dose escalation can be found in figure1.

The first dose of UC-MSCs in cohort 1 (three patients initially) will be 5.0×10^7 cells. The maximum-tolerated dose (MTD) is determined as the highest dose that induces DLT in no more than one patient among the extended cohort of six patients. Patients will be recruited into four cohorts and will begin dosing at successively higher doses until the MTD is established or up to a dosage of 2.0×10^8 cells.

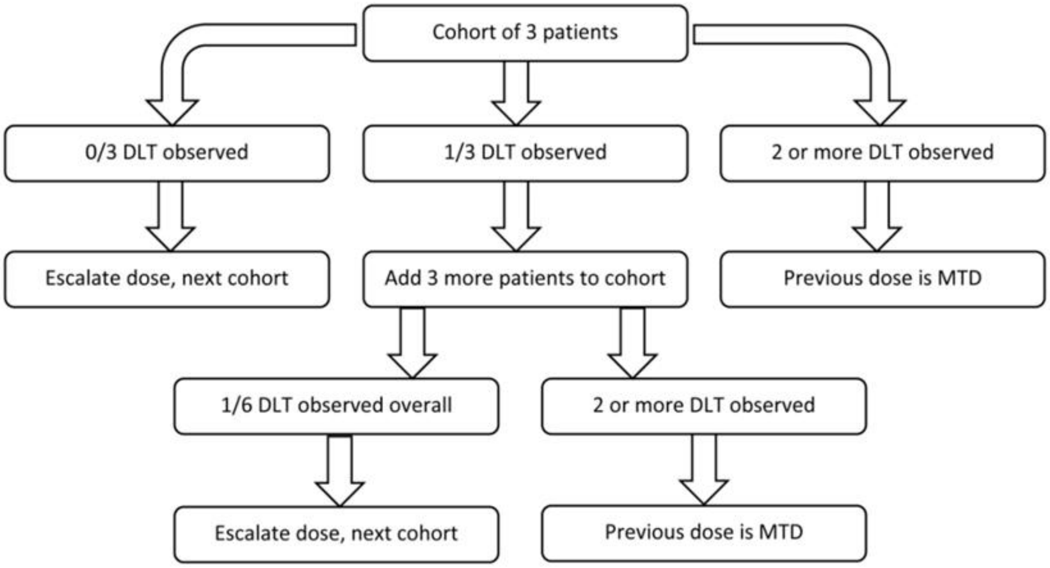

Figure 1 Dose escalation flowchart

3.8.3 Assessment of DLT and MTD

DLT will be evaluated according to the National Cancer Institute Common Toxicity Criteria (NCI-CTC) 5.0 (https://ctep.cancer.gov/protocoldevelopment/electronic_applications/ctc.htm). DLT is defined as any grade 4 hematological toxicity; grade 3 thrombocytopenia with hemorrhage; grade 3 or greater nausea, vomiting or, diarrhea or any grade 3 or greater treatment-related non-hematological toxicity (excluding alopecia and fatigue). Groups of three patients will be recruited at a time and allocated to their respective cohorts, starting with the lowest dose. For each cohort, one patient will be given the treatment at first and then observed for 3days for signs of DLT. The next patient's treatment will only begin after the first patient has undergone 3 days of observation. The treatment of another cohort start after the previous cohort has experienced at least 7 days of observation.

The trial will proceed as follows to ensure safe dose escalation and determine the MTD: (1) If there is no DLT observed in all three patients of a cohort, the trial will proceed to the next dose cohort; (2)

If one of the three patients of a cohort experiences DLT, then three more patients will be included and tested in this dose cohort. If still only one of the six patients experiences DLT, the trial will proceed to the next dose cohort. However, if two or more of the six patients experience DLT, the previous dose will be considered as the MTD; (3) If two or all three of the three initial patients experience DLT in a cohort, the MTD will remain undetermined.

3.8.4 Product management

The investigator will designate a dedicated person to be responsible for the storage, distribution, recovery, inventory and recording of investigational drugs. When the investigational product arrive at the research center, the researcher or his authorized person needs to check the storage conditions of the study drugs when they arrive, count the quantity, check the product number, and sign and confirm on the handover record form. The investigator or his authorized person will distribute the investigational drugs, and corresponding records will be kept for each distribution and use.

3.9 Data collection and data management

This study will use an electronic data collection system (EDC) to collect and manage research data to ensure the traceability of clinical trial data; the data management process must comply with GCP specifications and the "Stem Cell Clinical Research Management Measures" (2015 edition) to ensure the authenticity, completeness and accuracy of clinical trial data.

3.10 Statistical considerations

3.10.1 Statistical methods

Efficacy will be assessed according to the intent-to-treat (ITT) analysis principle, while safety will be assessed by analyzing adverse events within the cohorts and noting their category and severity. Continuous variables will be expressed as the mean with the standard deviation (SD). Categorical variables will be expressed as a number count together with the rate or composition ratio. The chi-square test or Fisher's exact test will be used to compare categorical variables between different doses used by the cohorts for evaluating efficacy. They will also be used to compare the rate of adverse events occurrence between cohorts to assess safety. The change in the patients' lab values in each cohort after treatment will also be described to explore any potential correlations

between these changes and the occurrence of adverse events. Continuous variables will be compared using the *t*-test. However, comparisons will be made using the Wilcoxon signed-rank test when analyzing changes in continuous quantitative variables before and after treatment. The 95% confidence interval (95% CI) will be calculated for the outcomes of the continuous variables. A more detailed plan for the statistical analysis will be formulated and finalized before the data gathering is finished for the study. This final plan will dictate the method and content of the study's statistical analysis. $P<0.05$ will be considered as showing statistical significance. All the statistical analyses will be performed using SAS 9.4 (Statistics Analysis System, Inc. Cary, NC, USA).

3.10.2 Analysis population

Full Analysis Set(FAS)： According to the principle of Intention to Treat (ITT) will constitute the Full Analysis Set for all subjects who were successfully enrolled and had at least one documented treatment.

Per Protocol Set(PPS)： It is a subset of FAS subjects who meet the inclusion criteria, not meet the exclusion criteria, complete the treatment regimen, fully adhere to the trial protocol, and not have any major protocol violations (subjects who will discontinue trial treatment due to disease progression will be included in the Per-Protocol Set, PPS).

Safety Set(SS)： Includes all subjects who have received an infusion. The safety population will primarily be used for the analysis of safety data.

3.11 Safety/harms

Adverse events (AEs) and severer adverse events (SAEs) will be monitored and recorded from the time the subject signed informed consent to the completion of the follow-ups. AEs/SAEs will be recorded in detail, including onset date, duration, severity, treatment, relation to the investigational medical product. All AEs/SAEs will be followed up until finalized (recover/relief, stable, deaths, or other explainable circumstances, e.g., lost to follow-up).

SAEs, once identified, must be taken into action and reported within 24 hours. SAEs will be reported to IRB as soon as possible.

3.12 Auditing

This study will be conducted following Good Clinical Practice (GCP). Investigators and coordinators will conduct source data verification when patients are hospitalized. Additional auditing and quality control by the site's GCP office and contract research organizations will be adopted.

4 ETHICS AND DISSEMINATION

4.1 Research ethics approval

The study has been approved by the institutional review boards of the Fifth Medical Center of PLA General Hospital (Approval#: 2018-107-D-4. The research process will be carried out in strict accordance with the requirements of the NMPA Quality Control Practice for Drug Clinical Trials and the Helsinki Declaration.

4.2 Protocol amendments

Major changes, such as study objectives, study design, patient population, sample sizes, study procedures, outcomes which may impact potential benefit or harm of the patients will require a formal amendment to the protocol and will be approved by IRB.

4.3 Informed consent process

The patient or their legal representative must read and understand and sign the informed consent form before enrolling in the study. Paper-based consent forms will be used in this study.

4.4 Confidentiality

The investigators are bound to keep all patient's records that contain names or other personal identifiers confidential. All research data will be identified by a study subject ID only. Data or records shall not be used for purposes other than this clinical study.

4.5 Declaration of interests

The principal investigators declare no financial and other competing interests.

4.6 Access to data

All investigators from the steering committee will be given full access to the final data sets.

4.7 Ancillary and post-trial care

The investigators will continue to follow up patients after 2 years for safety and research purposes. Long-period results of efficacy and safety will be collected.

4.8 Dissemination policy

The study team will communicate trial results with health authorities, professionals, and patients who participated in this study. The results of this trial will be published when available.

Further publications, authorship of this study results must be reviewed by the principal investigator and sponsor, and written consent must be obtained.

Data sharing policy will be described in detail in the data sharing statement when the study result is published.

5 STUDY ADMINISTRATION

5.1 Key contracts

Central contract

Principal Investigator: Fu-Sheng Wang, MD, Ph.D.86-10-66933332 fswang302@163.com

Investigator and coordinator: Lei Shi, MD, Ph.D. 86-10-66933333 shilei302@126.com

Study Sites: The Fifth Medical Center of PLA General Hospital, Beijng, China., 100039

5.2 Roles and responsibilities

5.2.1 Protocol contributors

Fu-Sheng Wang and Lei Shi conceived and designed the trial.

Lei Shi, Zerui Wang, Ziying Zhang, and Mengqi Yuan developed data management.

Yongji Wang developed statistical plan.

Lei Shi and Zerui Wang drafted the present protocol.

5.2.2 Sponsor and funding and collaborators

Sponsor

The Fifth Medical Center of PLA General Hospital

Funding

1. National Key Research and Development Program of China (2017YFA0105700, 2022YFA1105604)
2. The Innovation Platform for Academicians of Hainan Province (YSPTZX202216)
3. National Clinical Center for Infectious Diseases, PLA General Hospital (NCRC-ID202105,413FZT6)

Collaborators

Study site:

The Fifth Medical Center of Chinese PLA General Hospital

UC-MSC Provider:

VCANBIO Cell & Gene Engineering Corp., Ltd, China

Statistical analysis:

Beijing KeyTech Statistical Consulting Co., Ltd

6 REFERENCES

1. Mansour D, McPherson S. Management of decompensated cirrhosis. Clin Med (Lond) 2018;18:s60-s65. doi:10.7861/clinmedicine.18-2-s60.
2. Osborn C, Murrel D. Decompensated Cirrhosis. 2018.
3. Crismale JF, Friedman SL. Acute Liver Injury and Decompensated Cirrhosis. Med Clin North Am 2020;104:647-62. doi:10.1016/j.mcna.2020.02.010.

4. Potosek J, Curry M, Buss M, et al. Integration of palliative care in end-stage liver disease and liver transplantation. J Palliat Med 2014;17:1271-7. doi:10.1089/jpm.2013.0167.

5. Al-Dhamin Z, Liu LD, Li DD, et al. Therapeutic efficiency of bone marrow-derived mesenchymal stem cells for liver fibrosis: A systematic review of in vivo studies. World J Gastroenterol 2020;26:7444-69. doi:10.3748/wjg.v26.i47.7444.

6. Li TT, Wang ZR, Yao WQ, et al. Stem Cell Therapies for Chronic Liver Diseases: Progress and Challenges. Stem Cells Transl Med 2022;11:900-11. doi:10.1093/stcltm/szac053.

7. Jang YO, Jun BG, Baik SK, et al. Inhibition of hepatic stellate cells by bone marrow-derived mesenchymal stem cells in hepatic fibrosis. Clin Mol Hepatol 2015;21:141-9. doi:10.3350/cmh.2015.21.2.141.

8. Zhang LT, Peng XB, Fang XQ, et al. Human umbilical cord mesenchymal stem cells inhibit proliferation of hepatic stellate cells in vitro. Int J Mol Med 2018;41:2545-52. doi:10.3892/ijmm.2018.3500.

9. Ezquer F, Bruna F, Calligaris S, et al. Multipotent mesenchymal stromal cells: A promising strategy to manage alcoholic liver disease. World J Gastroenterol 2016;22:24-36. doi:10.3748/wjg.v22.i1.24.

10. Berardis S, Dwisthi Sattwika P, Najimi M, et al. Use of mesenchymal stem cells to treat liver fibrosis: current situation and future prospects. World J Gastroenterol 2015;21:742-58. doi:10.3748/wjg.v21.i3.742.

11. Suk KT, Yoon JH, Kim MY, et al. Transplantation with autologous bone marrow-derived mesenchymal stem cells for alcoholic cirrhosis: Phase 2 trial. Hepatology 2016;64:2185-97. doi:10.1002/hep.28693.

12. Lin BL, Chen JF, Qiu WH, et al. Allogeneic bone marrow-derived mesenchymal stromal cells for hepatitis B virus-related acute-on-chronic liver failure: A randomized controlled trial. Hepatology 2017;66:209-19. doi:10.1002/hep.29189.

13. Zhang Z, Lin H, Shi M, et al. Human umbilical cord mesenchymal stem cells improve liver function and ascites in decompensated liver cirrhosis patients. J Gastroenterol Hepatol 2012;27 Suppl 2:112-20. doi:10.1111/j.1440-1746.2011.07024.x.

14. Shi M, Zhang Z, Xu R, et al. Human mesenchymal stem cell transfusion is safe and improves liver function in acute-on-chronic liver failure patients. Stem Cells Transl Med 2012;1:725-31. doi:10.5966/sctm.2012-0034.

15. Wang L, Li J, Liu H, et al. Pilot study of umbilical cord-derived mesenchymal stem cell transfusion in patients with primary biliary cirrhosis. J Gastroenterol Hepatol 2013;28 Suppl 1:85-92. doi:10.1111/jgh.12029.

16. Shi M, Liu Z, Wang Y, et al. A Pilot Study of Mesenchymal Stem Cell Therapy for Acute Liver Allograft Rejection. Stem Cells Transl Med 2017;6:2053-61. doi:10.1002/sctm.17-0134.

17. Shi M, Li YY, Xu RN, et al. Mesenchymal stem cell therapy in decompensated liver cirrhosis: a long-term follow-up analysis of the randomized controlled clinical trial. Hepatol Int 2021;15:1431-41. doi:10.1007/s12072-021-10199-2.

18. Shi L, Huang H, Lu X, et al. Effect of human umbilical cord-derived mesenchymal stem cells on lung damage in severe COVID-19 patients: a randomized, double-blind, placebo-controlled phase 2 trial. Signal Transduct Target Ther 2021;6:58. doi:10.1038/s41392-021-00488-5.

19. Berry SM, Carlin BP, Lee JJ, et al. Bayesian adaptive methods for clinical trials: CRC press; 2010.

A clinical study on the safety and efficacy of human umbilical cord-derived mesenchymal stem cells for treating cirrhosis in the decompensated stage (MSC-DLC-1b)

Protocol

Version 1.2 – July 24, 2023

Sponsor	The Fifth Medical Center of PLA General Hospital
Principal Investigator	Fu-Sheng Wang
Ethics Reference Number	KY-2023-6-43-3
ClinicalTrials.gov	NCT05984303

Table of Content

1 TRIAL SUMMARY 27

2 INTRODUCTION 30

    2.1 Background and rationale 30

    2.2 Objectives 31

    2.3 Trial design 31

3 METHODS 31

    3.1 Study setting 31

    3.2 Eligibility criteria 31

        3.2.1 Inclusion criteria 31

        3.2.2 Exclusion criteria 32

    3.3 Interventions 33

        3.3.1 Investigational medicinal product 33

        3.3.2 Standard of care(SOC) 34

        3.3.3 Intervention assignment 34

        3.3.4 Modifications of intervention 34

    3.4 Outcomes 34

        3.4.1 Primary outcomes 34

        3.4.2 Secondary outcomes 34

    3.5 Participant timeline 35

    3.6 Sample size 38

    3.7 Recruitment 38

    3.8 Allocation and product management 38

        3.8.1 Allocation 38

        3.8.2 Dose escalation and dose-limiting toxicity 38

        3.8.3 Assessment of DLT and MTD 39

        3.8.4 Product management 40

    3.9 Data collection and data management 40

    3.10 Statistical considerations 40

        3.10.1 Statistical methods 40

        3.10.2 Analysis population 41

    3.11 Safety/harms 41

3.12 Auditing 41
4 ETHICS AND DISSEMINATION 42
4.1 Research ethics approval 42
4.2 Protocol amendments 42
4.3 Informed consent process 42
4.4 Confidentiality 42
4.5 Declaration of interests 42
4.6 Access to data 43
4.7 Ancillary and post-trial care 43
4.8 Dissemination policy 43
5 STUDY ADMINISTRATION 43
5.1 Key contracts 43
5.2 Roles and responsibilities 43
5.2.1 Protocol contributors 43
5.2.2 Sponsor and funding and collaborators 44
6 REFERENCES 44

1 TRIAL SUMMARY

Title	A clinical study on the safety and efficacy of human umbilical cord-derived mesenchymal stem cells for treating cirrhosis in the decompensated stage (MSC-DLC-1b)
Primary registry and trial identifying number	ClinicalTrials.gov NCT05984303
Secondary identifying numbers	KY-2023-6-43-3
Sources of monetary or material support	The Fifth Medical Center of PLA General Hospital
Primary sponsor	The Fifth Medical Center of PLA General Hospital
Central contract	Principal Investigator: Fu-Sheng Wang, MD, Ph.D.<br>86-10-66933332 fswang302@163.com<br>Investigator and coordinator: Lei Shi, MD, Ph.D.<br>86-10-66933333 shilei302@126.com<br>Study site:<br>The Fifth Medical Center of PLA General Hospital, Beijng, China., 100039
Study officials/investigators	Study Principal Investigator<br>Fu-Sheng Wang, MD, Ph.D.<br>The Fifth Medical Center of PLA General Hospital
Brief title	Human Umbilical Cord-derived Mesenchymal Stem Cells for Decompensated Cirrhosis (MSC-DLC-1b)
Countries of recruitment	P. R. China
Condition(s) or focus of study	Decompensated cirrhosis
Interventions	Human umbilical cord-derived mesenchymal stem cells(UC-MSCs)
Key eligibility criteria	Inclusion criteria<br>5. Willing to provide written informed consent<br>6. Aged 18 to 75 years (including 18 and 75 years), male or female

	7. Patients diagnosed with decompensated liver cirrhosis based on clinical findings, laboratory tests, imaging findings and/or representative pathological findings (decompensated liver cirrhosis is defined as the occurrence of at least one serious complication, including esophageal and gastric varices bleeding, hepatic encephalopathy, ascites, spontaneous bacterial peritonitis and other serious complications)<br>8. Child-Turcotte-Pugh (CTP) score 7 to 12 point<br>Exclusion criteria<br>1. Patients with hepatitis B cirrhosis who had HBV-DNA ≥ the lower limit of test, or who may have discontinued antiviral therapy during the study, or received anti-hepatitis B virus therapy for less than 12 months<br>2. Patients with hepatitis C cirrhosis who had HCV-RNA ≥ the lower limit of test, or who received anti-hepatitis C virus therapy for less than 12 months<br>3. Patients under treatment with corticosteroids for autoimmune hepatitis for less than 6 months<br>4. Trans-jugular intrahepatic portosystemic shunts (TIPS) insertion within 6 months prior to study inclusion<br>5. Active drinkers with alcohol-related decompensated cirrhosis are unwilling to stop alcohol abuse after inclusion<br>6. Patients were complicated with biliary obstruction or portal vein cavernous transformation<br>7. Patients who were known to have other malignant tumors within 5 years before signing informed consent, except for basal cell carcinoma, squamous cell carcinoma and/or carcinoma in situ which had received curative treatment<br>8. Patients with history of organ transplantation<br>9. Patients with severe heart, lung, kidney and blood system diseases<br>10. Patients with drug abuse, drug dependence and patients who receive methadone treatment or with psychosis<br>11. Patients with history of immunodeficiency disease, including a positive test result for human immunodeficiency virus (HIV)

	antibodies, or other acquired or congenital immunodeficiency diseases<br>12. Pregnant or lactating female. Fertile patients who were unable or unwilling to use effective non-pharmaceutical contraception during the trial and within 6 months after the end of the trial<br>13. Patients with cardiovascular and cerebrovascular events (such as unstable angina pectoris, cerebral hemorrhage, severe ischemic cerebral infarction) within 3 months before the first dose; Myocardial infarction or clinically significant cardiac rhythm or conduction abnormalities within 12 months before the first dose<br>14. Patients with allergic constitution (history of allergy to more than two foods or drugs) or history of severe allergy, or known severe allergy to the investigational drug or any of the excipients<br>15. Patients who have received previous stem cell therapy or are intolerant to cell therapy<br>16. Participants in other clinical trials within the past 3 months<br>17. Patients with any other condition considered by the investigator to be ineligible for participation in the study
Study design	Open-label, multiple-dose, dose-escalation, single-armed phase Ib trial
Date of enrollment	August 22, 2023 (The first subject signed the informed consent form)
Target sample size	6~12
Recruitment status	Participants in all dose groups have completed 28 days of observation after the first dose, and 2-year safety follow-up is ongoing. The trial finishing date is estimated as March 25, 2026
Primary outcomes	The primary outcomes are the incidence of DLTs, adverse events (AEs), MTD and the change in the MELD score from baseline to the 28th day after the first dose intervention
Secondary outcomes	The secondary outcomes include:<br>1. Change in the MELD score from baseline to 7 days, 14 days, 21 days, 2 months, 3 months, 6 months, 12 months, 18 months and 24 months after the intervention<br>2. Incidence of each complication associated with decompensated

	cirrhosis<br>3. Liver transplant-free survival<br>4. Incidence of liver failure<br>5. Plasma albumin (ALB) level<br>6. Plasma prealbumin (PALB) level<br>7. Total bilirubin (TBIL)<br>8. Serum cholinesterase (CHE)<br>9. Prothrombin time (PT)<br>10. Child-Turcotte-Pugh (CTP) score<br>11. EuroQol Group 5-Dimension Self-Report Questionnaire (EQ-5D) scale score<br>12. Incidence of liver cancer<br>13. Chronic Liver Disease Questionnaire (CLDQ) score

2 INTRODUCTION

2.1 Background and rationale

Decompensated liver cirrhosis (DLC) is characterized by severely impaired liver function and other associated complications, such as portal hypertension, ascites, spontaneous peritonitis, coagulation dysfunction, gastrointestinal bleeding, hepatic encephalopathy, and hepatorenal syndrome [1]. Current available treatment strategies can help improve the patients' quality of life and partially reduce the complications of DLC, and include taking diuretics and following a low-sodium diet, but there are no efficient therapeutic regimens [2, 3]. Furthermore, DLC indicates that the patient is nearing the end stage of liver failure, and liver transplantation then becomes the best option [4]. However, liver transplantation carries considerable risks, since liver failure is frequently associated with liver immune disorders that may contribute to rejection of the new organ and a decrease in the patient's quality of life [2]. In the absence of a liver transplant, an alternative therapy that can effectively relieve the symptoms and promote recovery would be very helpful for patients.

Mesenchymal stem cell (MSC) therapies have emerged as a novel alternative for the treatment of end-stage liver diseases [5, 6]. MSCs have a variety of useful properties, such as the secretion of cytokines, growth factors, and hepatocytes, which can help improve liver functionality and regress fibrosis. Studies have also shown that MSCs can suppress the proliferation of hepatic stellate cells and induce their apoptosis [7-10]. Multiple studies on MSCs have demonstrated their potential for treating end-stage liver disease [11-17], but the appropriate dose and related mechanisms are still unclear. In addition, no clinical research reports have yet explored escalating the dose of MSCs for treating DLC.

2.2 Objectives

We have designed an investigator-initiated, single-arm, dose-escalation clinical trial to evaluate the safety and identify the suitable dose of UC-MSCs as an infusion in patients with decompensated liver cirrhosis. This trial aims to provide evidence for evaluating the safety and efficacy of the UC-MSC infusion in future studies.

2.3 Trial design

This study was an open-label, multiple-dose, dose-escalation, single-armed phase Ib trial (MSC-DLC-1b) to evaluate the safety and efficacy of UC-MSCs for the treatment of patients with DLC. The study comprises three phases: screening + evaluation, 3 + 3 escalation, and follow up. Patients with DLC will be enrolled in the study. Each patient will receive UC-MSCs treatment on days 1, 7, and 14. After treatment, the patients will be followed up for 24 months.

3 METHODS

3.1 Study setting

The trial was conducted in The Fifth Medical Center of PLA General Hospital.

3.2 Eligibility criteria

3.2.1 Inclusion criteria

1. Willing to provide written informed consent

2. Aged 18 to 75 years (including 18 and 75 years), male or female
3. Patients diagnosed with decompensated liver cirrhosis based on clinical findings, laboratory tests, imaging findings and/or representative pathological findings (decompensated liver cirrhosis is defined as the occurrence of at least one serious complication, including esophageal and gastric varices bleeding, hepatic encephalopathy, ascites, spontaneous bacterial peritonitis and other serious complications)
4. Child-Turcotte-Pugh (CTP) score 7 to 12 points

3.2.2 Exclusion criteria

1. Patients with hepatitis B cirrhosis who had HBV-DNA≥ the lower limit of test, or who may have discontinued antiviral therapy during the study, or received anti-hepatitis B virus therapy for less than 12 months
2. Patients with hepatitis C cirrhosis who had HCV-RNA≥ the lower limit of test, or who received anti-hepatitis C virus therapy for less than 12 months
3. Patients under treatment with corticosteroids for autoimmune hepatitis for less than 6 months
4. Trans-jugular intrahepatic portosystemic shunts (TIPS) insertion within 6 months prior to study inclusion
5. Active drinkers with alcohol-related decompensated cirrhosis are unwilling to stop alcohol abuse after inclusion
6. Patients were complicated with biliary obstruction or portal vein cavernous transformation
7. Patients who were known to have other malignant tumors within 5 years before signing informed consent, except for basal cell carcinoma, squamous cell carcinoma and/or carcinoma in situ which had received curative treatment
8. Patients with history of organ transplantation
9. Patients with severe heart, lung, kidney and blood system diseases
10. Patients with drug abuse, drug dependence and patients who receive methadone treatment or with psychosis
11. Patients with history of immunodeficiency disease, including a positive test result for human immunodeficiency virus (HIV) antibodies, or other acquired or congenital immunodeficiency diseases

12. Pregnant or lactating female. Fertile patients who were unable or unwilling to use effective non-pharmaceutical contraception during the trial and within 6 months after the end of the trial
13. Patients with cardiovascular and cerebrovascular events (such as unstable angina pectoris, cerebral hemorrhage, severe ischemic cerebral infarction) within 3 months before the first dose; Myocardial infarction or clinically significant cardiac rhythm or conduction abnormalities within 12 months before the first dose
14. Patients with allergic constitution (history of allergy to more than two foods or drugs) or history of severe allergy, or known severe allergy to the investigational drug or any of the excipients
15. Patients who have received previous stem cell therapy or are intolerant to cell therapy
16. Participants in other clinical trials within the past 3 months
17. Patients with any other condition considered by the investigator to be ineligible for participation in the study

3.3 Interventions

3.3.1 Investigational medicinal product

The UC-MSCs (VUM02, 5×10^7 cells/10 mL/bag) will be prepared by Wuhan Optics Valley Vcanbiopharma Co., Ltd., in China. Briefly, mesenchymal stem cells (MSCs) will be obtained according to the method described in our previous study [18]. Umbilical cord tissue for sampling will be selected and collected after the mothers have signed the informed consent form. After the blood vessels are removed from the umbilical cord tissue, it will be cut into small pieces of about 1–2 mm^3, uniformly inoculated in cell culture flasks for isolation of the MSCs, and the primary cells will be digested with TrypLE digestive enzyme for 10–13 days. The primary cells will be inoculated in cell culture flasks at a density of about $2\times10^4/cm^2$ and cultured in an incubator at 37 °C with a volume fraction of 5% CO_2 and saturated humidity. After being passaged for P4 generation, the cells will be cryopreserved for use as the working cell bank. Subsequently, safety and biological performance tests will be conducted on the working cell bank. Following successful testing, the cells will undergo a resuscitation culture for 48 to 72 h to prepare P5 generation cell formulations. The cells must meet strict criteria to be used in our study. Specifically, the expression of cell immunophenotypes CD90, CD105, CD73 must be greater than 95%; the

expression of CD45, CD34, CD14, CD19, and HLA-DR must be lower than 2%; the cells must test negative for sterility and mycoplasma; and the intracellular toxins must be less than 1 EU /mL. Only UC-MSCs that pass these tests will be used for the clinical trials.

3.3.2 Standard of care(SOC)

Provide optimal support and medication in accordance with relevant latest clinical guidelines.

3.3.3 Intervention assignment

This study is an open-label, multiple-dose, dose-escalation, single-armed phase Ib trial. In this study, a total of two dose groups (1.0×10^8 cells, 2.0×10^8 cells) are set up, multiple dose escalation will be performed according to the "3+3" rule, each subject will be only entered into one corresponding dose group. See Section 3.8 for details.

3.3.4 Modifications of intervention

Subjects in each corresponding dose group who experience grade 3 or higher adverse events within one week after the first HUC-MSCs treatment may have their second HUC-MSCs treatment delayed by one week or terminated based on the specific clinical situation, and the assessment content and process must be recorded in detail.

3.4 Outcomes

3.4.1 Primary outcomes

The primary outcomes are the incidence of DLTs, adverse events(AEs), MTD and the change in the MELD score from baseline to the 28th day after the first dose intervention.

3.4.2 Secondary outcomes

The secondary outcomes include:

1. Change in the MELD score from baseline to 7 days, 14 days, 21 days, 2 months, 3 months, 6 months, 12 months, 18 months and 24 months after the intervention
2. Incidence of each complication associated with decompensated cirrhosis
3. Liver transplant-free survival
4. Incidence of liver failure

5. Plasma albumin (ALB) level
6. Plasma prealbumin (PALB) level
7. Total bilirubin (TBIL)
8. Serum cholinesterase (CHE)
9. Prothrombin time (PT)
10. Child-Turcotte-Pugh (CTP) score
11. EuroQol Group 5-Dimension Self-Report Questionnaire (EQ-5D) scale score
12. Incidence of liver cancer
13. Chronic Liver Disease Questionnaire (CLDQ) score

3.5 Participant timeline

Assessment schedule for the patients with decompensated cirrhosis

	Screening	Period of treatment			Observation period after treatment					Long-term safety follow-up		
**Timepoint**	Day -21 to 0	Day 1 ±0 day	Day 7 ± 0 day	Day 14 ± 0 day	Day 21 ± 1 day	Day 28 ± 1 days	Month 2 ±7 days	Month 3 ±7 days	Month 6 ±7 days	Month 12 ±7 days	Month 18 ±7 days	Month 24 ±7 days
Enrollment and informed consent	×											
Screening table	×											
Demographic data	×											
Allergies and family history	×											
Past medical history and treatment history	×											
Existing disease and concomitant treatment	×											
Vital signs	×	×	×	×	×	×	×	×	×	×	×	×
Physical examination	×	×	×	×	×	×	×	×	×	×	×	×
Hepatitis B blood test/ HCV antibody	×								×	×	×	×
HIV/TP antibody	×											
Blood routine test	×	×	×	×	×	×	×	×	×	×	×	×
Urine routine test	×	×	×	×		×	×	×	×	×	×	×
Urine pregnancy test	×											
Stool routine test + occult blood	×	×	×	×		×	×	×	×	×	×	×
Blood biochemistry test	×	×	×	×	×	×	×	×	×	×	×	×
Coagulation function test	×	×	×	×	×	×	×	×	×	×	×	×
Blood ammonia	×	×	×	×	×	×	×	×	×	×	×	×
AFP	×	×	×	×	×	×	×	×	×	×	×	×
Electrocardiogram	×	×	×	×	×	×	×	×	×	×	×	×
Chest X-ray or CT	×									×		×
Abdominal ultrasound examination	×	×			×	×	×	×	×	×	×	×
Elastography	×					×		×				
Gastroscopy	×								×			
Upper abdominal CT/MRI plain scan + enhancement	×								×	×	×	×
MELD score	×	×	×	×	×	×	×	×	×			

Child–Pugh score	×				×	×	×	×	×			
QoL scale	×	×			×	×	×	×	×	×	×	×
Collection of samples for omics research (blood sample + stool)		×	×	×	×	×	×	×	×			
Infusion records		×	×	×								
Adverse events		×	×	×	×	×	×	×	×	×	×	×
Concomitant medication/treatment	×	×	×	×	×	×	×	×	×	×	×	×

3.6 Sample size

This is a proof-of-concept study. A total of 3-6 patients will be recruited for each dose cohort, comprising low and high dose groups, with 3 patients in each cohort initially. The additional patients will be added to the cohort if dose-limiting toxicity is presented. A total of 6-12 patients will be recruited for the study [19].

3.7 Recruitment

Patient will be recruited from The Fifth Medical Center of PLA General Hospital. No other recruitment strategy (e.g., advertisement) will be adopted in this study.

3.8 Allocation and product management

3.8.1 Allocation

This study is an open-label, multiple-dose, dose-escalation, single-armed phase Ib trial. In this study, a total of two dose groups (1.0×10^8 cells, 2.0×10^8 cells) are set up, multiple dose escalation will be performed according to the "3+3" rule, each subject will be only entered into one corresponding dose group.

3.8.2 Dose escalation and dose-limiting toxicity

In this study, a total of two dose groups (1.0×10^8 cells, 2.0×10^8 cells) were set up, multiple dose escalation was performed according to the "3+3" rule, each subject was only entered into one corresponding dose group. Starting from the low-dose group (1.0×10^8 cells), each subject was closely observed for 7 days after the corresponding dose of UC-MSCs treatment, and the possible DLTs were evaluated. Subjects in the same dose group must complete the DLT assessment for 7 days before starting the next subject, and the first patient in the high-dose group (2.0×10^8 cells) could not be enrolled until the last patient in the low-dose group (1.0×10^8 cells) had completed a 28-day safety assessment. During dose escalation, safety data from the previous dose group were used by the investigator and the sponsor to determine whether to proceed with the next dose.

During the observation period of DLT, if none of the 3 subjects in a dose group observed DLT (0/3), they could be escalated to the next dose group. If there was one DLT (1/3) in a dose group, 3

additional subjects in this dose group were required, and if the 3 additional subjects had no DLT (total DLT1/6), they could escalate to the next dose group. If one or more DLTs (total DLTs ≥2/6) occurred in the supplementary 3 patients, the dose escalation was terminated, and the previous dosage was MTD. If no DLT occurred after the completion of dose escalation in either dose group, the decision to continue dose escalation was made in joint discussion between the investigator and the sponsor. The specific rules for dose escalation can be found in **Figure 1** [19].

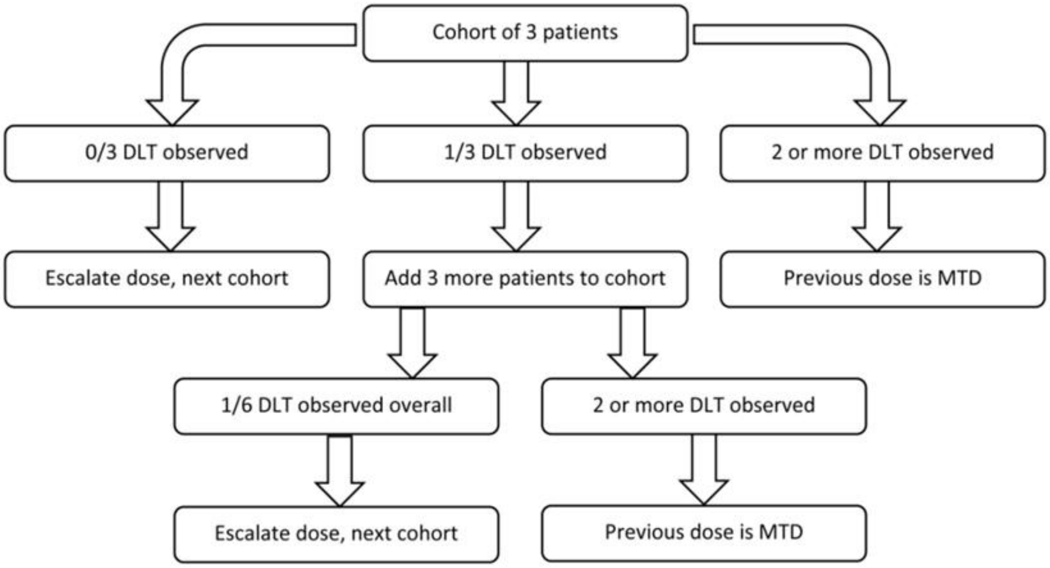

Figure 1 Dose escalation flowchart

3.8.3 Assessment of DLT and MTD

DLT will be evaluated according to the National Cancer Institute Common Toxicity Criteria (NCI-CTC) 5.0 (https://ctep.cancer.gov/protocoldevelopment/electronic_applications/ctc.htm). The following drug-related adverse events were considered DLTs: (1) Grade ≥3 allergic reactions related to UC-MSCs, such as dyspnea, chills and fever; (2) Grade ≥3 embolic adverse events related to UC-MSCs, such as acute pulmonary embolism, deep vein thrombosis, etc.; (3) Grade ≥3 hematological toxicity related to UC-MSCs; (4) Any unexpected toxicity requiring discontinuation of treatment at the discretion of the investigator and sponsor.

During the observation period of DLT, if none of the 3 subjects in a dose group observed DLT (0/3), they could be escalated to the next dose group. If there was one DLT (1/3) in a dose group, 3 additional subjects in this dose group were required, and if the 3 additional subjects had no DLT (total DLT1/6), they could escalate to the next dose group. If one or more DLTs (total DLTs ≥2/6)

occurred in the supplementary 3 patients, the dose escalation was terminated, and the previous dosage was MTD. If no DLT occurred after the completion of dose escalation in either dose group, the decision to continue dose escalation was made in joint discussion between the investigator and the sponsor.

3.8.4 Product management

The investigator will designate a dedicated person to be responsible for the storage, distribution, recovery, inventory and recording of investigational drugs. When the investigational product arrive at the research center, the researcher or his authorized person needs to check the storage conditions of the study drugs when they arrive, count the quantity, check the product number, and sign and confirm on the handover record form. The investigator or his authorized person will distribute the investigational drugs, and corresponding records will be kept for each distribution and use.

3.9 Data collection and data management

This study will use an electronic data collection system (EDC) to collect and manage research data to ensure the traceability of clinical trial data; the data management process must comply with GCP specifications and the "Stem Cell Clinical Research Management Measures" (2015 edition) to ensure the authenticity, completeness and accuracy of clinical trial data.

3.10 Statistical considerations

3.10.1 Statistical methods

Efficacy will be assessed according to the intent-to-treat (ITT) analysis principle, while safety will be assessed by analyzing adverse events within the cohorts and noting their category and severity. Continuous variables will be expressed as the mean with the standard deviation (SD). Categorical variables will be expressed as a number count together with the rate or composition ratio. The chi-square test or Fisher's exact test will be used to compare categorical variables between different doses used by the cohorts for evaluating efficacy. They will also be used to compare the rate of adverse events occurrence between cohorts to assess safety. The change in the patients' lab values in each cohort after treatment will also be described to explore any potential correlations between these changes and the occurrence of adverse events. Continuous variables will be

compared using the *t*-test. However, comparisons will be made using the Wilcoxon signed-rank test when analyzing changes in continuous quantitative variables before and after treatment. The 95% confidence interval (95% CI) will be calculated for the outcomes of the continuous variables. A more detailed plan for the statistical analysis will be formulated and finalized before the data gathering is finished for the study. This final plan will dictate the method and content of the study's statistical analysis. $P<0.05$ will be considered as showing statistical significance. All the statistical analyses will be performed using SAS 9.4 (Statistics Analysis System, Inc. Cary, NC, USA).

3.10.2 Analysis population

Full Analysis Set(FAS)： According to the principle of Intention to Treat (ITT) will constitute the Full Analysis Set for all subjects who were successfully enrolled and had at least one documented treatment.

Per Protocol Set(PPS)： It is a subset of FAS subjects who meet the inclusion criteria, not meet the exclusion criteria, complete the treatment regimen, fully adhere to the trial protocol, and not have any major protocol violations (subjects who will discontinue trial treatment due to disease progression will be included in the Per-Protocol Set, PPS).

Safety Set(SS)： Includes all subjects who have received an infusion. The safety population will primarily be used for the analysis of safety data.

3.11 Safety/harms

Adverse events (AEs) and severer adverse events (SAEs) will be monitored and recorded from the time the subject signed informed consent to the completion of the follow-ups. AEs/SAEs will be recorded in detail, including onset date, duration, severity, treatment, relation to the investigational medical product. All AEs/SAEs will be followed up until finalized (recover/relief, stable, deaths, or other explainable circumstances, e.g., lost to follow-up).

SAEs, once identified, must be taken into action and reported within 24 hours. SAEs will be reported to IRB as soon as possible.

3.12 Auditing

This study will be conducted following Good Clinical Practice (GCP). Investigators and coordinators will conduct source data verification when patients are hospitalized. Additional auditing and quality control by the site's GCP office and contract research organizations will be adopted.

4 ETHICS AND DISSEMINATION

4.1 Research ethics approval

The study has been approved by the institutional review boards of the Fifth Medical Center of PLA General Hospital (Approval#: KY-2023-6-43-3. The research process will be carried out in strict accordance with the requirements of the NMPA Quality Control Practice for Drug Clinical Trials and the Helsinki Declaration.

4.2 Protocol amendments

Major changes, such as study objectives, study design, patient population, sample sizes, study procedures, outcomes which may impact potential benefit or harm of the patients will require a formal amendment to the protocol and will be approved by IRB.

4.3 Informed consent process

The patient or their legal representative must read and understand and sign the informed consent form before enrolling in the study. Paper-based consent forms will be used in this study.

4.4 Confidentiality

The investigators are bound to keep all patient's records that contain names or other personal identifiers confidential. All research data will be identified by a study subject ID only. Data or records shall not be used for purposes other than this clinical study.

4.5 Declaration of interests

The principal investigators declare no financial and other competing interests.

4.6 Access to data

All investigators from the steering committee will be given full access to the final data sets.

4.7 Ancillary and post-trial care

The investigators will continue to follow up patients after 2 years for safety and research purposes. Long-period results of efficacy and safety will be collected.

4.8 Dissemination policy

The study team will communicate trial results with health authorities, professionals, and patients who participated in this study. The results of this trial will be published when available.

Further publications, authorship of this study results must be reviewed by the principal investigator and sponsor, and written consent must be obtained.

Data sharing policy will be described in detail in the data sharing statement when the study result is published.

5 STUDY ADMINISTRATION

5.1 Key contracts

Central contract

Principal Investigator: Fu-Sheng Wang, MD, Ph.D.86-10-66933332 fswang302@163.com

Investigator and coordinator: Lei Shi, MD, Ph.D. 86-10-66933333 shilei302@126.com

Study Sites: The Fifth Medical Center of PLA General Hospital, Beijng, China., 100039

5.2 Roles and responsibilities

5.2.1 Protocol contributors

Fu-Sheng Wang and Lei Shi conceived and designed the trial.

Lei Shi, Zerui Wang, Ziying Zhang, and Mengqi Yuan developed data management.

Yongji Wang developed statistical plan.

Lei Shi and Zerui Wang drafted the present protocol.

5.2.2 Sponsor and funding and collaborators

Sponsor

The Fifth Medical Center of PLA General Hospital

Funding

1. National Key Research and Development Program of China (2017YFA0105700, 2022YFA1105604)
2. The Innovation Platform for Academicians of Hainan Province (YSPTZX202216)
3. National Clinical Center for Infectious Diseases, PLA General Hospital (NCRC-ID202105,413FZT6)

Collaborators

Study site:

The Fifth Medical Center of Chinese PLA General Hospital

UC-MSC Provider:

VCANBIO Cell & Gene Engineering Corp., Ltd, China

Statistical analysis:

Beijing KeyTech Statistical Consulting Co., Ltd

6 REFERENCES

1. Mansour D, McPherson S. Management of decompensated cirrhosis. Clin Med (Lond) 2018;18:s60-s65. doi:10.7861/clinmedicine.18-2-s60.
2. Osborn C, Murrel D. Decompensated Cirrhosis. 2018.
3. Crismale JF, Friedman SL. Acute Liver Injury and Decompensated Cirrhosis. Med Clin North Am 2020;104:647-62. doi:10.1016/j.mcna.2020.02.010.

4. Potosek J, Curry M, Buss M, et al. Integration of palliative care in end-stage liver disease and liver transplantation. J Palliat Med 2014;17:1271-7. doi:10.1089/jpm.2013.0167.
5. Al-Dhamin Z, Liu LD, Li DD, et al. Therapeutic efficiency of bone marrow-derived mesenchymal stem cells for liver fibrosis: A systematic review of in vivo studies. World J Gastroenterol 2020;26:7444-69. doi:10.3748/wjg.v26.i47.7444.
6. Li TT, Wang ZR, Yao WQ, et al. Stem Cell Therapies for Chronic Liver Diseases: Progress and Challenges. Stem Cells Transl Med 2022;11:900-11. doi:10.1093/stcltm/szac053.
7. Jang YO, Jun BG, Baik SK, et al. Inhibition of hepatic stellate cells by bone marrow-derived mesenchymal stem cells in hepatic fibrosis. Clin Mol Hepatol 2015;21:141-9. doi:10.3350/cmh.2015.21.2.141.
8. Zhang LT, Peng XB, Fang XQ, et al. Human umbilical cord mesenchymal stem cells inhibit proliferation of hepatic stellate cells in vitro. Int J Mol Med 2018;41:2545-52. doi:10.3892/ijmm.2018.3500.
9. Ezquer F, Bruna F, Calligaris S, et al. Multipotent mesenchymal stromal cells: A promising strategy to manage alcoholic liver disease. World J Gastroenterol 2016;22:24-36. doi:10.3748/wjg.v22.i1.24.
10. Berardis S, Dwisthi Sattwika P, Najimi M, et al. Use of mesenchymal stem cells to treat liver fibrosis: current situation and future prospects. World J Gastroenterol 2015;21:742-58. doi:10.3748/wjg.v21.i3.742.
11. Suk KT, Yoon JH, Kim MY, et al. Transplantation with autologous bone marrow-derived mesenchymal stem cells for alcoholic cirrhosis: Phase 2 trial. Hepatology 2016;64:2185-97. doi:10.1002/hep.28693.
12. Lin BL, Chen JF, Qiu WH, et al. Allogeneic bone marrow-derived mesenchymal stromal cells for hepatitis B virus-related acute-on-chronic liver failure: A randomized controlled trial. Hepatology 2017;66:209-19. doi:10.1002/hep.29189.
13. Zhang Z, Lin H, Shi M, et al. Human umbilical cord mesenchymal stem cells improve liver function and ascites in decompensated liver cirrhosis patients. J Gastroenterol Hepatol 2012;27 Suppl 2:112-20. doi:10.1111/j.1440-1746.2011.07024.x.
14. Shi M, Zhang Z, Xu R, et al. Human mesenchymal stem cell transfusion is safe and improves liver function in acute-on-chronic liver failure patients. Stem Cells Transl Med 2012;1:725-31. doi:10.5966/sctm.2012-0034.

15. Wang L, Li J, Liu H, et al. Pilot study of umbilical cord-derived mesenchymal stem cell transfusion in patients with primary biliary cirrhosis. J Gastroenterol Hepatol 2013;28 Suppl 1:85-92. doi:10.1111/jgh.12029.

16. Shi M, Liu Z, Wang Y, et al. A Pilot Study of Mesenchymal Stem Cell Therapy for Acute Liver Allograft Rejection. Stem Cells Transl Med 2017;6:2053-61. doi:10.1002/sctm.17-0134.

17. Shi M, Li YY, Xu RN, et al. Mesenchymal stem cell therapy in decompensated liver cirrhosis: a long-term follow-up analysis of the randomized controlled clinical trial. Hepatol Int 2021;15:1431-41. doi:10.1007/s12072-021-10199-2.

18. Shi L, Huang H, Lu X, et al. Effect of human umbilical cord-derived mesenchymal stem cells on lung damage in severe COVID-19 patients: a randomized, double-blind, placebo-controlled phase 2 trial. Signal Transduct Target Ther 2021;6:58. doi:10.1038/s41392-021-00488-5.

19. Berry SM, Carlin BP, Lee JJ, et al. Bayesian adaptive methods for clinical trials: CRC press; 2010.
