# Supplementary material for: Dose-escalation studies of mesenchymal stromal cell therapy for decompensated liver cirrhosis: phase Ia/Ib results and immune modulation insights
Source: Signal Transduct Target Ther. 2025 Jul 29;10:238. doi: 10.1038/s41392-025-02318-4 (PMC12304480; doi:10.1038/s41392-025-02318-4)
Supplement: Supplementary file 2 — Statistical Analysis Plan (SAP) [file 41392_2025_2318_MOESM2_ESM.pdf]

Supplementary File 2

Statistical Analysis Plan (SAP)

This appendix contains the following two clinical study SAPs:

Clinical study	Page
An exploratory clinical study of the tolerability, safety and efficacy of human umbilical cord-derived mesenchymal stem cells for dose-escalation in patients with decompensated cirrhosis (MSC-DLC-1a)	2 ~ 26
A clinical study on the safety and efficacy of human umbilical cord-derived mesenchymal stem cells for treating cirrhosis in the decompensated stage (MSC-DLC-1b)	27 ~ 50

An exploratory clinical study of the tolerability, safety and efficacy of human umbilical cord-derived mesenchymal stem cells for dose-escalation in patients with decompensated cirrhosis

Statistical Analysis Plan

**Sponsor**	Chinese PLA General Hospital
**Collaboration**	Chinese PLA General Hospital
**Protocol No.**	MSC-DLC
**Author**	Beijing KeyTech Statistical Consulting Co., Ltd

Statistical Analysis Plan Approval Form

**Protocol Title:**	An exploratory clinical study of the tolerability, safety and efficacy of human umbilical cord-derived mesenchymal stem cells for dose-escalation in patients with decompensated cirrhosis
**SAP Version:**	V1.1
**SAP Date:**	21 July 2023

The statistical analysis plan has been reviewed and approved.

Principal Investigator: *Fusheng Wang*
Chinese PLA General Hospital

Signature ______________________ Date ______________

Project Statistician: *Yin Li*
Beijing KeyTech Statistical Consulting Co., Ltd

Signature ______________________ Date ______________

Approver: *Zhiwei Jiang, PhD*
General Manager
Beijing KeyTech Statistical Consulting Co., Ltd

Signature ______________________ Date ______________

Contents

1 LIST OF ABBREVIATIONS AND DEFINITION OF TERMS 5
2 INTRODUCTION 5
2.1 Preface 5
2.2 Changes to the Protocol-Planned Analysis 6
3 Purpose of Analysis 6
4 Study Design 6
4.1 Overall Study Design 6
4.2 Randomisation 8
4.3 Sample Size 8
5 STUDY ENDPOINTS 8
5.1 Endpoints of Safety 8
5.2 Endpoints of Efficacy 10
5.2.1 Primary Endpoints of Efficacy 10
5.2.2 Secondary Endpoints of Efficacy 10
6 ANALYSIS SETS 14
7 STATISTICAL METHOD 15
7.1 General Considerations 15
7.1.1 General Method 15
7.1.2 Related Definitions and Derivation Rules 15
7.1.3 Analysis Window 17
7.1.4 Analysis Software 17
7.1.5 Table and Listing 17
7.2 Disposition of Participants 18
7.3 Demographics and Clinical Characteristics 18
7.4 Concomitant Medication 19
7.5 Hypothesis 19
7.6 Safety Evaluation 19
7.6.1 DLT Occurrence 19
7.6.2 Adverse Events 19
7.6.3 Laboratory Tests 20
7.6.4 ECG 20
7.6.5 Physical Examinations 20
7.6.6 Vital Signs 20
7.6.7 HIV/TP Antibodies 21
7.6.8 Quantification of Nucleic Acids 21
7.6.9 AFP 21
7.6.10 Abdominal Ultrasoundv 21
7.6.11 Hepatitis B penta/Hepatitis C Virus (HCV) Antibodies 22
7.6.12 Other 22
7.7 Efficacy Evaluation 22
7.7.1 Primary Efficacy Evaluation 22
7.7.2 Secondary Efficacy Evaluation 22
7.8 Handling of Missing Data 24
7.9 Subgroup Analysis 24
7.10 Multiplicity 24
7.11 Interim Analysis 24
8 Notes on this Plan 25
VERSION HISTORY 26

1 LIST OF ABBREVIATIONS AND DEFINITION OF TERMS

ALT	Alanine Aminotransferase
AST	Aspartate Transaminase
BMI	Body Mass Index
CT	Computed Tomography
CT/MRI	Computed Tomography & Magnetic Resonance Imaging
DLT	Dose Limited Toxicity
EDC	Electronic Data Capture System
EQ-5D-5L	The 5-Level Euroqol Five Dimensions Questionnaire
FAS	Full Analysis Set
HCV	Hepatitis C Virus
HIV/TP	Human Immunodeficiency Virus & Treponema Pallidum
ITT	Intention To Treat
LEU	Leukocyte
LOCF	Last Observation Carried Forward
MedDRA	Medical Dictionary for Regulatory Activities
MELD	Model for End-Stage Liver Disease
MTD	Maximum Tolerated Dose
PPS	Per Protocol Set
PT	Preferred Term
SAP	Statistical Analysis Plan
SAS	Statistical Analysis System
SOC	System Organ Class
SS	Safety Set
TEAE	Treatment-Emergent Adverse Event
VAS	Visual Analogue Scale

2 INTRODUCTIONS

2.1 Preface

This document is the statistical analysis plan (SAP) for **"An exploratory clinical study of the tolerability, safety and efficacy of human umbilical cord-derived mesenchymal stem cells for dose-escalation in patients with decompensated"**, which will provide the details and methods to analyze and report the results of baseline characteristics, efficacy and safety.

This SAP will be finalized and approved before the database is locked, and the programming of the corresponding statistical analysis will be progressively completed as the study data is accumulating until the database is locked.

Mockup Shell will be provided as an attachment of this SAP.

2.2 Changes to the Protocol-Planned Analysis

The definition of the Dose-Limited Toxicity (DLT) analysis set, along with the corresponding statistical analyses, has been added.

3 Purpose of Analysis

Primary Purpose : To explore the tolerated dose and safety of human umbilical cord-derived mesenchymal stem cells in the treatment of decompensated cirrhosis, and to establish a basis for determining their safety, efficacy, recommended usage, and dosage.

Secondary Purpose : To explore the changes in endogenous substances and immune-related indices before and after the infusion of human umbilical cord-derived mesenchymal stem cells, to elucidate the mechanism of drug action through histological research (including metabolomics, proteomics, immunomics, or a combination of these approaches) and systems biology, and to search for the potential clinical biomarkers for exploratory subgroup analyses of the clinical trials.

4 Study Design

4.1 Overall Study Design

The study is designed as a single-center, dose-escalation, single-arm study. Subjects with decompensated cirrhosis will be screened as the target disease for the clinical investigation. The study follows the ‘3+3’ dose-escalation rule, with 12 to 24 participants expected to be enrolled, and 3 to 6 subjects in each of the low, medium, high, and ultra-high dose groups.

Dose-Escalation Rule:

In this study, dose escalation will be conducted according to the ‘3+3’ rule, where each subject will be assigned to a dose group, starting with the low dose group. Each subject received the corresponding dose of VUM02, followed by close monitoring and assessment for potential dose-limiting toxicities (DLTs). Within the low, medium, and high dose groups, subjects must complete a 3-day DLT evaluation before the next subject in the same group can begin dosing. Dosing in the subsequent dose group can only proceed after all subjects in the current group have completed a 7-day DLT evaluation. In the ultra-high-dose group, each subject will be treated with the appropriate dose of VUM02 and observed for 7 days, with the 7-day DLT assessment being completed before the next subject could be dosed. Throughout the dose escalation process, the investigator and sponsor will evaluate the safety data from the previous dose group to determine whether to proceed with dosing in the next dose level.

During the DLT observation period, if no dose-limiting toxicities (DLTs) occur in a dose group (0/3), the dose can be escalated to the next level. If 1 DLT (1/3) occurs in a dose group, the group must be supplemented with 3 additional subjects. If none of the 3 supplementary subjects experience DLTs (total DLT 1/6), the dose can be escalated to the next group. However, if 1 or more DLTs occur among the 3 supplemented subjects (total DLT ⩾ 2/6), dose escalation will be halted, and the previous dose level will be considered the maximum tolerated dose (MTD). If the highest dose is reached without any DLTs, this dose will be considered the MTD, and the decision to continue dose escalation will be made through a discussion between the investigator and the sponsor.

Efficacy Observations：

①The Model for End-Stage Liver Disease (MELD) scores, along with the Child-Pugh scores, will be collected from all subjects at screening, on day 1, day 3, day 7, day 14, day 28, and at month 3, month 6, month 9, month 12, month 15, month 18, month 21, and month 24.

②Quality of life assessments will be collected from all subjects at screening, day 14, day 28, and at month 3, month 6, month 9, month 12, month 15, month 18, month 21, and month 24. the assessments included three scales: the alcohol dependence scale, the chronic liver disease questionnaire, and the 5-Level EuroQoL Five Dimensions Questionnaire (EQ-5D-5L).

The occurrence of complications related to decompensated cirrhosis, liver transplantation, liver failure, and hepatocellular carcinoma will be collected in subjects throughout the trial.

Safety Observations：

①The results of vital signs, physical examination, hematology, serum chemistry, coagulation, blood ammonia, and electrocardiogram (ECG) will be collected from all subjects at the screening period, day 1, day 3, day 7, day 14, day 28, and at month 3, month 6, month 9, month 12, month 15, month 18, month 21, and month 24.

②The results of urine routine, stool routine (including occult blood), and alpha-fetoprotein (AFP) tests will be collected from all subjects at the screening phase, on day 1, day 14, day 28, and at month 3, month 6, month 9, month 12, month 15, month 18, month 21, and month 24.

③Hepatitis B penta/Hepatitis C Virus (HCV) antibodies, Human Immunodeficiency Virus (HIV) & Treponema Pallidum (TP) antibodies, upper abdominal Computed Tomography (CT) and Magnetic Resonance Imaging (MRI) with and without contrast, as well as gastroscopy results, will be collected from all subjects at the Screening period, and at month 6, month 12, month 18, and month 24.

④The results of abdominal ultrasound will be collected from all subjects during the screening phase, as well as on day 1, day 28, month 3, month 6, month 9, month 12, month 15, month 18, month 21 and month 24.

⑤ Chest radiographs/computed tomography (CT) scans of the chest will be collected from all subjects during the screening phase, at month 12,month 24.

⑥ FibroScan results will be collected from all subjects at screening, month 3, month 6, month 9, month 12, month 15, month 18, month 21, and month 24.

⑦ Adverse events, serious adverse events, and concomitant medications occurring during the trial will be collected.

This study will be conducted by Chinese PLA General Hospital。

4.2 Randomisation

Not applicable.

4.3 Sample Size

This trial is an exploratory study. Subjects with decompensated cirrhosis will be screened as the disease type for the clinical study. A total of 9 to 18 subjects are expected to be enrolled, with 3 to 6 subjects in each of the low, medium, high, and ultra-high dose groups.

5 STUDY ENDPOINTS

5.1 Endpoints of Safety

(1)DLT Events

DLT is defined as any grade 4 hematological toxicity; grade 3 thrombocytopenia with hemorrhage; grade 3 or greater nausea, vomiting or, diarrhea or any grade 3 or greater treatment-related non-hematological toxicity (excluding alopecia and fatigue).

(2)Adverse Events

(3)Laboratory Examinations

- **Hematology**： including leukocytes, percentage of neutrophils, percentage of lymphocytes, percentage of monocytes, percentage of eosinophils, percentage of basophils, absolute neutrophil count, absolute lymphocyte count, absolute monocyte count, absolute eosinophil count, absolute basophil count, erythrocytes, hemoglobin, hematocrit, mean corpuscular volume (MCV), mean corpuscular hemoglobin (MCH), red blood cell distribution width (RDW), platelets, mean platelet volume (MPV), plateletcrit (PCT), platelet distribution width (PDW), and large platelet ratio；
- **Serum Chemistry**： including alanine aminotransferase (ALT), aspartate aminotransferase (AST), total protein, albumin, globulin, total bilirubin, direct bilirubin, total bile acids, glucose, urea,

creatinine, uric acid, alkaline phosphatase, γ-glutamyltransferase (GGT), potassium, sodium, chloride, creatine kinase (CK), lactate dehydrogenase (LDH), prealbumin, direct/total bilirubin ratio, AST/ALT ratio, cholinesterase, total cholesterol, triglycerides, high-density lipoprotein (HDL) cholesterol, low-density lipoprotein (LDL) cholesterol；

direct/total bilirubin ratio = direct bilirubin ratio / total bilirubin ratio；

AST/ALT ratio = Aspartate aminotransferase / Alanine aminotransferase；

- **Urine Routine**：including pH, specific gravity, glucose, protein, ketone bodies, erythrocytes, leukocytes, leukocyte esterase (LEU), erythrocyte esterase (LEU)；
- **Coagulation**：including Prothrombin time, international normalized ratio, activity, activated partial thromboplastin time, fibrinogen, thrombin time, activated partial thromboplastin ratio；
- **Stool Routine (including Occult Blood)**：including fecal erythrocytes, fecal leukocytes, occult blood；
- **Blood Ammonia。**

(4)ECG

(5)Physical Examinations

Including general condition, skin, lymph nodes, head and neck region, chest, abdomen, musculoskeletal system, nervous system, others.

(6)Vital Signs

Including temperature, respiration rate, pulse rate, systolic blood pressure, diastolic blood pressure, body weight.

(7)HIV/TP Antibodies

Including human immunodeficiency virus antibodies, syphilis treponemal antibodies.

(8)Quantification of Nucleic Acids

Including hepatitis B virus nucleic acid, hepatitis C virus nucleic acid.

(9)AFP

(10)Abdominal Ultrasound

(11)Hepatitis B penta/Hepatitis C Virus (HCV) Antibodies

Including hepatitis B surface antigen, hepatitis B surface antibody, hepatitis B e antigen, hepatitis B e antibody, hepatitis B core antibody, hepatitis C antibody.

(12)Other

Including CT/MRI plain and contrast-enhanced imaging of the upper abdomen, chest x-ray/chest CT, gastroscopy, and fibroscan examination.

5.2 Endpoints of Efficacy

5.2.1 Primary Endpoints of Efficacy

28-day Model for End-Stage Liver Disease (MELD) Score.

5.2.2 Secondary Endpoints of Efficacy

➢ **Model for End-Stage Liver Disease (MELD) scores and their changes from baseline at each visit:** including MELD scores and the changes from baseline at visits on day 3, day 7, day 14, month 3, month 6, month 9, month 12, month 15, month 18, month 21, and month 24.

➢ **Child-Pugh score**

Child-Pugh total score and its change from baseline at day 3, day 7, day 14, day 28, month 3, month 6, month 9, month 12, month 15, month 18, month 21, and month 24.

Grading of the Child-Pugh total score and its change from baseline at day 3, day 7, day 14, day 28, month 3, month 6, month 9, month 12, month 15, month 18, month 21, and month 24.

The proportion of subjects with different scores in the individual Child-Pugh components and their change from baseline at day 3, day 7, day 14, day 28, month 3, month 6, month 9, month 12, month 15, month 18, month 21, and month 24.

The scoring criteria for individual items are as follows: The scoring criteria for individual items are as follows:

**Criteria**	**1 point**	**2 point**	**3 point**
Encephalopathy(Grade)	None	Grade I-II	Grade III-IV
Ascites	None	Mild	Moderate to Severe
Bilirubin (Total)(umol/L)	<34	34－51	>51
Albumin(g/L)	>35	28-35	<28
Prothrombin time(s)	<4	4-6	>6

➢ **Alcohol Dependence Scale**

Total scores on the alcohol dependence scale and their changes from baseline at visits on day 14, day 28, month 3, month 6, month 9, month 12, month 15, month 18, month 21, and month 24.

distribution of clinical grades and their changes from baseline at visits on day 14, day 28, month 3, month 6, month 9, month 12, month 15, month 18, month 21, and month 24.

the scores for all items of the alcohol dependence scale totalled 47 points, which will be categorized into five clinical grades based on the total score. The criteria for evaluating the individual scores as well as the clinical grades are outlined below:

**Standard Category**	**Entry**	**Criterion**
Individual scoring criteria	1. How much did you drink the last time you drank?	scored 0. Enough to get high or less, scored 1. Enough to get drunk, scored 2. Enough to pass out
	2. Do you often have hangovers on Sunday or Monday mornings?	scored 0. No, scored 1. Yes
	3. Have you had the "shakes" when sobering up (hands tremble, shake inside)?	scored 0. No, scored 1. Sometimes, scored 2. Often
	4. Do you get physically sick (e.g., vomit, stomach cramps) as a result of drinking?	scored 0. No, scored 1. Sometimes, scored 2. Almost every time I drink
	5. Have you had the "DTs" (delirium tremens) – that is, seen, felt or heard things not really there; felt very anxious, restless, and over excited?	scored 0. No, scored 1. Sometimes, scored 2. Several times
	6. When you drink, do you stumble about, stagger, and weave?	scored 0. No, scored 1. Sometimes, scored 2. Often
	7. As a result of drinking, have you felt overly hot and sweaty (feverish)?	scored 0. No, scored 1. Once, scored 2. Several times
	8. As a result of drinking, have you seen things that were not really there?	scored 0. No, scored 1. Once, scored 2. Several times
	9. Do you panic because you fear you may not have a drink when you need it?	scored 0. No, scored 1. Yes
	10. Have you had blackouts ("loss of memory" without passing out) as a result of drinking?	scored 0. No, never, scored 1. Sometimes, scored 2. Often, scored 3. Almost every time I drink
	11. Do you carry a bottle with you or keep one close at hand?	scored 0. No, scored 1. Some of the time, scored 2. Most of the time
	12. After a period of abstinence (not drinking), do you end up drinking heavily again?	scored 0. No, scored 1. Sometimes, scored 2. Almost every time I drink
	13. In the past 12 months, have you passed out as a result of drinking?	scored 0. No, scored 1. Once, scored 2. More than once
	14. Have you had a convulsion (fit) following a period of drinking?	scored 0. No, scored 1. Yes, scored 2. Several times
	15. Do you drink throughout the day?	scored 0. No, scored 1. Yes
	16. After drinking heavily, has your thinking been fuzzy or unclear?	scored 0. No, scored 1. Yes, but only for a few hours, scored 2. Yes, for one or two days, scored 3. Yes, for many days
	17. As a result of drinking, have you felt your heart beating rapidly?	scored 0. No, scored 1. Yes
	18. Do you almost constantly think about drinking and alcohol?	scored 0. No, scored 1. Yes
	19. As a result of drinking, have you heard "things" that were not really there?	scored 0. No, scored 1. Yes, scored 2. Several times

	20. Have you had weird and frightening sensations when drinking?	scored 0. No, scored 1. Once or twice, scored 2. Often
	21. As a result of drinking have you "felt things" crawling on you that were not really there (e.g., bugs, spiders)?	scored 0. No, scored 1. Yes, scored 2. Several times
	22. With respect to blackouts (loss; of memory)?	scored 0. Have never had a blackout<br>scored 1. Have had blackouts that last less than an hour<br>scored 2. Have had blackouts that last for several hours<br>scored 3. Have had blackouts that last a day or more
	23. Have you tried to cut down on your drinking and failed?	scored 0. No, scored 1. Once, scored 2. Several times
	24. Do you gulp drinks (drink quickly)？	scored 0. No, scored 1. Yes
	25. After taking one or two drinks, can you usually stop?	scored 0. Yes, scored 1. No
Clinical Level Criteria	Grade 1：No evidence of alcohol dependence was reported.	ADS Raw Score：0
	Grade 2：Low level of alcohol dependence.	ADS Raw Score：1-13
	Grade 3：Intermediate level of alcohol dependence.	ADS Raw Score：14-21
	Grade 4：Substantial level of alcohol dependence.	ADS Raw Score：22-30
	Grade 5：Severe level of alcohol dependence.	ADS Raw Score：31-47

- **Chronic Liver Disease Questionnaire**

Total scores on the chronic liver disease questionnaire and their changes from baseline at visits on day 14, day 28, month 3, month 6, month 9, month 12, month 15, month 18, month 21, and month 24.

Total scores for each domain of the chronic liver disease questionnaire and their changes from baseline at visits on day 14, day 28, month 3, month 6, month 9, month 12, month 15, month 18, month 21, and month 24.

The criteria for evaluating individual scores, as well as those for assessing each domain, are outlined below.

Scale or factor	Entry	Criteria
Individual scoring criteria	All of the time	Score 1
	Most of the time	Score 2
	A good bit of the time	Score 3
	Some of the time	Score 4
	A little of the time	Score 5
	Hardly any of the time	Score 6
	None of the time	Score 7
Domain scoring criteria	Items 1：Abdominal symptoms(AS)	question 1，5，17
	Items 2：Fatigue(FA)	question 2，4，8，11，13
	Items 3：Systemic symptoms(SS)	question 3，6，21，23，27
	Items 4：Activity(AC)	question 7，9，14
	Items 5：Emotional function(EF)	question 10，12，15，16，19，20，24，26
	Items 6：Worry(WO)	question 18，22，25，28，29

- **EQ-5D-5L Scale**

The index values of the EQ-5D-5L scales and their changes from baseline scores will be calculated on day 14, day 28, month 3, month 6, month 9, month 12, month 15, month 18, month 21, and month 24 visits according to Chinese standards.

index value = 1-(factor of mobility + factor of self-care + factor of usual activities + factor of pain/discomfort + factor of anxiety/depression).

	Level of value set				
	Level 1	Level 2	Level 3	Level 4	Level 5
MOBILITY	0	0.066	0.158	0.287	0.345
SELF-CARE	0	0.048	0.116	0.210	0.253
USUAL ACTIVITIES	0	0.045	0.107	0.194	0.233
PAIN / DISCOMFORT	0	0.058	0.138	0.252	0.302

	Level of value set				
	Level 1	Level 2	Level 3	Level 4	Level 5
ANXIETY / DEPRESSION	0	0.049	0.118	0.215	0.258

The Visual Analogue Scale (VAS) of the EQ-5D-5L scale and the change from baseline score will be calculated on day 14, day 28, month 3, month 6, month 9, month 12, month 15, month 18, month 21, and month 24.

➢ **Incidence of Complications associated with Decompensated Cirrhosis**

The proportion of subjects who will experience complications related to decompensated cirrhosis following the infusion of the investigational drug, as well as the proportion of subjects experiencing each specific complication.

➢ **Survival without Liver Transplantation**

The proportion of subjects who remain alive without undergoing liver transplantation after receiving the investigational drug infusion.

➢ **Liver Failure**

The proportion of subjects with liver failure after receiving the investigational drug infusion.

➢ **Liver Cancer**

The proportion of subjects with liver cancer after receiving the investigational drug infusion.

6 ANALYSIS SETS

(1)Full Analysis Set(FAS)： According to the principle of Intention to Treat (ITT) will constitute the Full Analysis Set for all subjects who were successfully enrolled and had at least one documented treatment. The following conditions may result in the exclusion of enrolled subjects from the FAS: for example, failure to receive at least one dose of the study medication, or absence of any post-enrollment data.

Demographic and baseline characteristics, as well as efficacy endpoints, will be statistically analyzed based on the FAS.

(2)Per Protocol Set(PPS)： Subjects who meet the inclusion criteria, not meet the exclusion criteria, complete the treatment regimen, fully adhere to the trial protocol, and not have any major protocol violations (subjects who will discontinue trial treatment due to disease progression will be included in the Per-Protocol Set, PPS).

Efficacy endpoints will be statistically analyzed based on the FAS or PPS.

(3)Dose Limited Toxicity Set(DLT)： Includes all subjects who have received an infusion. The DLT analysis set will be used primarily for DLT evaluation.

(4)Safety Set(SS)： Includes all subjects who have received an infusion. The safety population will primarily be used for the analysis of safety data.

The above analysis datasets will be reviewed and discussed by the principal investigator, sponsor, statistician, and data management team in a data review session prior to the database lock.

7 STATISTICAL METHOD

7.1 General Considerations

7.1.1 General Method

➢ **Descriptive Analysis**

Unless otherwise specified, the following descriptive statistics are given according to the variable types:

- ✓ Continuous variables will be summarized by mean, standard deviation, median, minimum and maximum.
- ✓ Categorical or ordinal variables will be summarized by frequency counts and percentages, where the denominator will be the number of participants in the corresponding analysis set.

➢ **Decimal places**

Unless otherwise specified, the number of decimal points in the statistical analysis report will be performed according to the following rules:

- ✓ The minimum and maximum values are consistent with the maximum decimal number of the original data.
- ✓ The number of decimal points of median, mean, standard deviation and 95% CI is 1 place more than the maximum decimal number of the original data.
- ✓ Percentages, Rates, rate differentials and rate ratios are retained to 2 decimal places.
- ✓ If the P-value is ⩾0.0001, it is retained to 4 decimal places; if the P-value is <0.0001, it is reported as “<0.0001”.
- ✓ Test statistics for all statistical tests are retained to 3 decimal places.
- ✓ Derived data is retained to 2 decimal places.

7.1.2 Related Definitions and Derivation Rules

➢ **Baseline**

Baseline is defined as the last non-missing value before the first dose.

➢ **Conversion among year, month and day**

Month = days /30.4375, year = days /365.25, rounded to one decimal.

- **Study Days**

The study start date is defined as the infusion date. The study day corresponding to a visit or event will be calculated using the following formula, with the study start date as the reference point:

✓ If the event date occurs before the study start date, study day = event date – study start date;

✓ if the event date occurs on or after the study start date, study day = event date – study start date +1.

- **Adverse Event**

Adverse Event Occurrence Time (Days) = Adverse Event Occurrence Date – Infusion Date.

Adverse Event Duration (Days) = Adverse Event End Date – Adverse Event Start Date + 1.

- **Treatment Emergent Adverse Event(TEAE)**

A TEAE (Treatment-Emergent Adverse Event) is defined as an adverse event that occurs on or after the infusion (including the same day), or one that occurs prior to the infusion but is exacerbated after the infusion. The following rules are applied during programming:

✧ If the adverse event occurs after (or on the same day as) the start of treatment with the investigational drug, it will be counted as a TEAE;

✧ If the adverse event occurs before the start of treatment with the investigational drug, but its severity worsens after treatment, it will be counted as a TEAE;

✧ If the adverse event or the start time of treatment with the investigational drug is missing, and it is not possible to clearly determine the relationship between the occurrence of the adverse event and the start of treatment, the event will be counted as a TEAE for all purposes;

✧ If the adverse event occurs prior to the start of treatment with the investigational drug, but the severity is missing and it cannot be determined whether the severity worsened after treatment, the event will be counted as a TEAE.

Due to varying severity, multiple observations of the same adverse event may be recorded in the Clinical Trial Electronic Data Capture (EDC) system. The following rules are applied to determine whether each observation of these adverse event qualifies as a TEAE:

(1)If the start time of all observations for the adverse event is after (or on the same day as) the first dose of the investigational drug, all observations for that adverse event will be classified as TEAEs;

(2)If any observation starts before the first dose of the investigational drug but ends after (or on the same day as) the first dose, the severity of this observation will serve as the baseline. For all subsequent observations with a start time after the first dose, the severity will be compared to this reference point. If the severity has increased, the observation will be classified as a TEAE; if the severity remains the same or decreases, the observation will be classified as a non-TEAE.

According to the above rules, multiple TEAEs may be recorded for the same adverse event. However, when calculating instances, only one TEAE will be counted per adverse event, with the severity being determined by the highest severity level recorded among the TEAEs.

- **Relevance of Adverse Events**

An adverse event is considered relevant to the study drug if its relationship is classified as “Definitely related”, “Highly Likely Relevant”, “Possibly related”, “Possibly Not Relevant”, or if the relevance is missing. An adverse event is considered unrelated to the study drug if its relationship is classified as “Definitely Irrelevant”.

- **Medical History, Treatment History, Current Illness, and Concomitant Treatment**

"Medical History" refers to the names of diseases recorded in the past medical history and treatment history form. "Treatment History" refers to the treatments recorded in the past medical history and treatment history form.

"Current Disease" refers to the diseases recorded in the current disease and concomitant treatment form, while "Concomitant Treatment" refers to the treatments documented in the same form.

- **Prior Medication and Concomitant Medication**

"Prior Medication" refers to medications that will be discontinued before the infusion. "Concomitant Medication" refers to medications that: (1) totalled initiated before the infusion and will be still being used at the time of the infusion, or (2) will be initiated on or after the infusion date.

- **Coding**

Medical history or newly emerging diseases, adverse events and serious adverse events will be coded according to the Medical Dictionary for Regulatory Activities (MedDRA), version 24.0 or later. Prior /concomitant medication will be coded according to the Anatomical Therapeutic Chemical Classification (ATC).

7.1.3 Analysis Window

For visits after baseline, all analyses will be conducted based on the scheduled visits in the protocol. Results of both scheduled and unscheduled visits will be listed.

7.1.4 Analysis Software

All statistical analysis will be conducted by SAS 9.4 or later version.

7.1.5 Table and Listing

- **Table**

All data will be summarized by treatment (i.e., low dose group, medium dose group, high dose group and ultra-high dose group). The treatments will be presented in columns.

- **Listing**

Unless otherwise specified, all listings will include group, subject ID. SDTM data will be preferred to presented in the listings.

7.2 Disposition of Participants

Summarize separately the number of subjects enrolled, completed the trial, and included in each analysis set, and analyze the reasons for subject withdrawals.

Provide separate lists of subjects who withdrew from the trial and those who will be will be not included in each analysis set.

Statistically describe the protocol deviations/violations for each group, both in total and by category. Provide a list of protocol deviations/violations.

7.3 Demographics and Clinical Characteristics

The following demographic and baseline characteristics will be statistically summarized for each group:

- Demographic characteristics(including age, sex, ethnicity, height, weight, and Body Mass Index (BMI));
- Clinical characteristics at screening (including disease duration, allergy history, family history, smoking history, alcohol use, substance abuse history, treatment history, and concomitant medications);
- Baseline physical examination (including general condition, skin, lymph nodes, head and neck region, chest, abdomen, musculoskeletal system, nervous system, and other);
- Baseline vital signs (temperature, respiratory rate, pulse rate, systolic blood pressure, diastolic blood pressure);

Disease duration (months) = (date of informed consent signing - date of first diagnosis + 1) / 30.4375.

Medical history and current diseases will be categorized according to System Organ Class (SOC) and Preferred Term (PT), and the frequency of occurrences, number of cases, and incidence rates will be calculated for each group.

Prior medication use will be coded according to the ATC classification. The number of occurrences, number of cases, and frequency of use after infusion will be calculated for each group, categorized by the ATC classification.

Separate lists will be generated for the following: demographic data, allergy history, family history, smoking history, alcohol consumption history, substance abuse history, clinical trial participation status, past medical history and treatments, current diseases and concomitant treatments, and other treatments.

Demographic data and baseline characteristics will be analyzed based on the FAS.

7.4 Concomitant Medication

Concomitant medication will be coded according to ATC. The number and percentage of subjects who have concomitant medication and usage frequency will be tabulated by ATC, and a list of concomitant medications will be generated.

The above analysis is based on SS.

7.5 Hypothesis

It is an exploratory clinical study and no formal statistical tests will be performed.

7.6 Safety Evaluation

7.6.1 DLT Occurrence

The incidence of DLT will be calculated for each group, and its 95% confidence interval will be calculated by the Clopper-Pearson method.

Descriptive statistics will be performed for the time from infusion to the occurrence of DLT for each dose group, including the calculation of the mean, standard deviation, median, Q1, Q3, minimum, and maximum values.

A list of subjects experiencing DLT will be compiled.

7.6.2 Adverse Events

Adverse events will be medically coded using MedDRA version 24.0 or the latest version. They will be categorized and counted according to the two levels of System Organ Class (SOC) and Preferred Term (PT). The number of occurrences, number of cases, and incidence rates for each type of adverse event will be calculated separately for each group in the following categories:

- All adverse events;
- Adverse events related to the study drug;
- Adverse events leading to discontinuation of the study drug;
- Adverse events related to the study drug leading to discontinuation of the study drug;
- Adverse events with severity ⩾ Grade 3;
- Adverse events with severity ⩾ Grade 3 related to the study drug;
- Adverse events leading to withdrawal from the trial;
- Adverse events related to the study drug leading to withdrawal from the trial;
- Serious adverse events;
- Serious adverse events related to the study drug;
- Adverse events leading to death.

Separate lists will be generated for post-treatment adverse events, adverse events leading to withdrawal from the trial, adverse events leading to death, adverse events occurring before treatment, and serious adverse events.

7.6.3 Laboratory Tests

The test values of various laboratory tests (including routine blood tests, blood biochemistry, and coagulation function indicators) and their changes from baseline to post-treatment time points (until the end of treatment or early withdrawal of subjects from the trial) will be statistically described for each group separately. The number of subjects, mean, standard deviation, median, minimum, and maximum values will be calculated.

For each group, the results of various laboratory tests (including routine blood tests, blood biochemistry, urinalysis, coagulation function, stool analysis with occult blood, and blood ammonia levels) will be presented in the form of shift tables comparing pre- and post-treatment values. This comparison will be based on normal reference ranges and the investigator's judgment of clinical significance, showing the change in the worst results from pre-treatment to post-treatment (up to the end of treatment or until withdrawal from the trial).

The lists of laboratory tests will be compiled.。

7.6.4 ECG

For each group, the results of ECG will be presented in the form of shift tables comparing pre- and post-treatment values. This comparison will be based on normal reference ranges and the investigator's judgment of clinical significance, showing the change in the worst results from pre-treatment to post-treatment (up to the end of treatment or until withdrawal from the trial).

The list of ECG will be compiled.。

7.6.5 Physical Examinations

For each group, the results of various physical examinations (Including General condition, skin, lymph nodes, head and neck region, chest, abdomen, musculoskeletal system, nervous system, others) will be presented in the form of shift tables comparing pre- and post-treatment values. This comparison will be based on normal reference ranges and the investigator's judgment of clinical significance, showing the change in the worst results from pre-treatment to post-treatment (up to the end of treatment or until the withdrawal from the trial).

The list of physical examinations will be compiled.。

7.6.6 Vital Signs

The test values of various vital signs (including temperature, respiration rate, pulse rate, systolic blood pressure, diastolic blood pressure, body weight) and their changes from baseline to post-treatment time points (until the end of treatment or early withdrawal of subjects from the trial) will be statistically described for each group separately. The number of subjects, mean, standard deviation, median, minimum, and maximum values will be calculated.

The list of vital signs will be compiled.。

7.6.7 HIV/TP Antibodies

For each group, the results of various HIV/TP antibodies (including human iimmunodeficiency virus antibodies, ssyphilis treponemal antibodies) will be presented in the form of shift tables comparing pre- and post-treatment values. This comparison will be based on normal reference ranges and the investigator's judgment of clinical significance, showing the change in the worst results from pre-treatment to post-treatment (up to the end of treatment or until the withdrawal from the trial).

The list of HIV/TP antibodies will be compiled.。

7.6.8 Quantification of Nucleic Acids

For each group, the results of various quantification of nucleic acids (including hepatitis B virus nucleic aacid, hepatitis C virus nucleic acidv) will be presented in the form of shift tables comparing pre- and post-treatment values. This comparison will be based on normal reference ranges and the investigator's judgment of clinical significance, showing the change in the worst results from pre-treatment to post-treatment (up to the end of treatment or until the withdrawal from the trial).

The list of quantification of nucleic acids will be compiled.。

7.6.9 AFP

For each group, the results of AFP will be presented in the form of shift tables comparing pre- and post-treatment values. This comparison will be based on normal reference ranges and the investigator's judgment of clinical significance, showing the change in the worst results from pre-treatment to post-treatment (up to the end of treatment or until the withdrawal from the trial).

The list of AFP will be compiled.。

7.6.10 Abdominal Ultrasound

For each group, the results of abdominal ultrasound will be presented in the form of shift tables comparing pre- and post-treatment values. This comparison will be based on normal reference ranges and the investigator's judgment of clinical significance, showing the change in the worst results from pre-treatment to post-treatment (up to the end of treatment or until the withdrawal from the trial).

The list of abdominal ultrasound will be compiled.。

7.6.11 Hepatitis B penta/Hepatitis C Virus (HCV) Antibodies

For each group, the results of various hepatitis B penta/HCV antibodies (including hepatitis B surface antigen, hepatitis B surface antibody, hepatitis B e antigen, hepatitis B e antibody, hepatitis B core antibody, hepatitis C antibody) will be presented in the form of shift tables comparing pre- and post-treatment values. This comparison will be based on normal reference ranges and the investigator's judgment of clinical significance, showing the change in the worst results from pre-treatment to post-treatment (up to the end of treatment or until the withdrawal from the trial).

The list of hepatitis B penta/HCV antibodies will be compiled.。

7.6.12 Other

For each group, the results of CT/MRI plain and contrast-enhanced imaging of the upper abdomen, chest X-ray/chest CT, gastroscopy, and fibroscan examination will be presented in the form of shift tables comparing pre- and post-treatment values. This comparison will be based on normal reference ranges and the investigator's judgment of clinical significance, showing the change in the worst results from pre-treatment to post-treatment (up to the end of treatment or until the withdrawal from the trial).

The lists of CT/MRI plain and contrast-enhanced imaging of the upper abdomen, chest X-ray/cchest CT, gastroscopy, and fibroscan examination will be compiled.。

7.7 Efficacy Evaluation

7.7.1 Primary Efficacy Evaluation

28-day Model for End-Stage Liver Disease (MELD) score

Statistical analysis of the 28-day Model for End-Stage Liver Disease (MELD) score will be conducted using an analysis of covariance (ANCOVA) model. In this model, the 28-day MELD score will be treated as the dependent variable, with groups as fixed effects and baseline values as a covariate. The least squares means (LS-means) for the 28-day MELD scores will be calculated for each group, along with the differences in adjusted means between groups and their corresponding 95% confidence intervals.

Additionally, statistical descriptive analysis of the 28-day MELD score and its change from baseline will be performed.

7.7.2 Secondary Efficacy Evaluation

(1) Model for End-Stage Liver Disease (MELD) scores and their changes from baseline at each visit

Descriptive statistics of the Model for End-Stage Liver Disease (MELD) scores and their changes from baseline will be provided for day 3, day 7, day 14, month 3, month 6, month 9, month 12, month 15, month 18, month 21, and month 24.

(2) Child-Pugh score

Descriptive statistics of the model for Child-Pugh total score and their changes from baseline will be provided for day 3, day 7, day 14, day 28, month 3, month 6, month 9, month 12, month 15, month 18, month 21, and month 24.

Descriptive statistics of the Child-Pugh total score grading and its changes from baseline, including the number of subjects and percentages, will be provided for each group of subjects at day 3, day 7, day 14, day 28, month 3, month 6, month 9, month 12, month 15, month 18, month 21, and month 24.

Descriptive statistics of the individual components of the Child-Pugh score grading and its changes from baseline, including the number of subjects and percentages, will be provided for each group of subjects at day 3, day 7, day 14, day 28, month 3, month 6, month 9, month 12, month 15, month 18, month 21, and month 24.

(3) Alcohol Dependence Scale

Descriptive statistics of the model for total scores of alcohol dependence scale and their changes from baseline will be provided for day 14, day 28, month 3, month 6, month 9, month 12, month 15, month 18, month 21, and month 24.

Descriptive statistics of the clinical grade distribution of the alcohol dependence scale and its changes from baseline, including the number of subjects and percentages, will be provided for each group of subjects at day 14, day 28, month 3, month 6, month 9, month 12, month 15, month 18, month 21, and month 24.

(4) Chronic Liver Disease Questionnaire

Descriptive statistics of the model for total scores of chronic liver disease questionnaire and their changes from baseline will be provided for day 14, day 28, month 3, month 6, month 9, month 12, month 15, month 18, month 21, and month 24.

Descriptive statistics of the model for domains scores of chronic liver disease questionnaire and their changes from baseline will be provided for day 14, day 28, month 3, month 6, month 9, month 12, month 15, month 18, month 21, and month 24.

(5) EQ-5D-5L Scale

Descriptive statistics of the model for index value scores of EQ-5D-5L Scale and their changes from baseline will be provided for day 14, day 28, month 3, month 6, month 9, month 12, month 15, month 18, month 21, and month 24.

Descriptive statistics of the model for VAS scores of EQ-5D-5L Scale and their changes from

baseline will be provided for day 14, day 28, month 3, month 6, month 9, month 12, month 15, month 18, month 21, and month 24.

(6) Incidence of complications associated with decompensated cirrhosis

The total incidence of complications associated with decompensated cirrhosis and the incidence of each individual complication in each group will be calculated, with 95% confidence intervals by using the Clopper-Pearson method.

(7) Survival without liver transplantation

The incidence of survival without liver transplantation in each group will be calculated, with 95% confidence intervals by using the Clopper-Pearson method.

(8) Liver Failure

The incidence of liver failure in each group will be calculated, with 95% confidence intervals by using the Clopper-Pearson method.

(9) Liver Cancer

The incidence of liver cancer in each group will be calculated, with 95% confidence intervals by using the Clopper-Pearson method.

7.8 Handling of Missing Data

In this trial, if any individual score required to calculate the total score for each domain of the chronic liver disease questionnaire or the EQ-5D-5L index value was missing, the corresponding derived endpoint will be treated as missing.

For the statistical analysis of the Full Analysis Set (FAS), the trial will employ the Last Observation Carried Forward (LOCF) method to handle missing data for the MELD score, Child-Pugh total score, the grading of the Child-Pugh score, the total score of the alcohol dependence scale and the distribution of clinical grades from the alcohol dependence scale alcohol dependence scale, the total score of the chronic liver disease questionnaire, the domain scores of the chronic liver disease questionnaire, and the EQ-5D-5L utility index values, as well as the EQ-5D-5L VAS score.

Missing data for other efficacy and safety outcomes will be not addressed in this trial.

7.9 Subgroup Analysis

Not Applicable.

7.10 Multiplicity

The p-values calculated in this trial are nominal p-values, primarily used to characterize the strength of the association between the evaluation endpoints and the treatment groups, and should not be interpreted as a basis for formal statistical inference.

7.11 Interim Analysis

Not Applicable.

8 Notes on this Plan

This analysis plan has been drafted based on the relevant descriptions outlined in the study protocol, which specifies the efficacy and safety evaluation endpoints. Specific statistical methods for the evaluation endpoints have been proposed, considering the basic characteristics of the indicators in the study protocol, as well as the specific requirements of this study. Given the possibility of unforeseen changes in the final data distribution of the clinical trial, the statistical methods may be subject to minor adjustments, and the presentation of the corresponding statistical analysis results may also undergo certain changes.

VERSION HISTORY

Version Number	Version Date	Author	Description of Change
V1.0	16Feb2023	Yin Li	Initial version
V1.1	21July2023	Yin Li	Added ultra-high dose groups, as well as gastroscopy and liver transient imaging elastography (FibroScan).

A Clinical Study on the Safety and Efficacy of Human Umbilical Cord-Derived Mesenchymal Stem Cells for Treating Cirrhosis in the Decompensated Stage (Climbing Trial)

Statistical Analysis Plan

**Sponsor**	Chinese PLA General Hospital
**Collaboration**	Chinese PLA General Hospital
**Protocol No.**	MSC-DLC
**Author**	Beijing KeyTech Statistical Consulting Co., Ltd

Statistical Analysis Plan Approval Form

**Protocol Title:**	A Clinical Study on the Safety and Efficacy of Human Umbilical Cord-Derived Mesenchymal Stem Cells for Treating Cirrhosis in the Decompensated Stage (Climbing Trial)
**SAP Version:**	V1.0
**SAP Date:**	20Nov2023

The statistical analysis plan has been reviewed and approved.

Principal Investigator: *Fusheng Wang*
Chinese PLA General Hospital

Signature ______________________ Date ______________________

Project Statistician: *Yin Li*
Beijing KeyTech Statistical Consulting Co., Ltd

Signature ______________________ Date ______________________

Approver: *Zhiwei Jiang, PhD*
General Manager
Beijing KeyTech Statistical Consulting Co., Ltd

Signature ______________________ Date ______________________

Contents

1 LIST OF ABBREVIATIONS AND DEFINITION OF TERMS 30
2 INTRODUCTIONS 30
2.1 Preface 30
2.2 Changes to the Protocol-Planned Analysis 31
3 Purpose of Analysis 31
4 Study Design 31
4.1 Overall Study Design 31
4.2 Randomisation 33
4.3 Sample Size 33
5 STUDY ENDPOINTS 33
5.1 Endpoints of Safety 33
5.2 Endpoints of Efficacy 34
6 ANALYSIS SETS 39
7 STATISTICAL METHOD 40
7.1 General Considerations 40
7.1.1 General Method 40
7.1.2 Related Definitions and Derivation Rules 40
7.1.3 Analysis Window 42
7.1.4 Analysis Software 42
7.1.5 Table and Listing 42
7.2 Disposition of Participants 42
7.3 Demographics and Clinical Characteristics 43
7.4 Concomitant Medication 43
7.5 Hypothesis 44
7.6 Safety Evaluation 44
7.6.1 DLT Occurrence 44
7.6.2 Adverse Events 44
7.6.3 Laboratory Tests 44
7.6.4 ECG 45
7.6.5 Physical Examinations 45
7.6.6 Vital Signs 45
7.6.7 Fibroscan Examination 45
7.6.8 Quantification of Nucleic Acids 46
7.6.9 AFP 46
7.6.10 Abdominal Ultrasound 46
7.6.11 Hepatitis B penta/Hepatitis C Virus (HCV) Antibodies 46
7.6.12 Gastroscopy 47
7.6.12 Other 47
7.7 Efficacy Evaluation 47
7.8 Handling of Missing Data 48
7.9 Subgroup Analysis 49
7.10 Multiplicity 49
7.11 Interim Analysis 49
8 Notes on this Plan 49
VERSION HISTORY 50

1 LIST OF ABBREVIATIONS AND DEFINITION OF TERMS

AFP	Alpha-Fetoprotein
ATC	Anatomical Therapeutic Chemical classification
CT	Computed Tomography
CTCAE	Common Terminology Criteria for Adverse Events
DLT	Dose Limited Toxicity
EDC	Electronic Data Capture System
EQ-5D-5L	The 5-Level Euroqol Five Dimensions Questionnaire
FAS	Full Analysis Set
HCV	Hepatitis C Virus
HIV	Human Immunodeficiency Virus
ITT	Intention to Treat
LOCF	Last Observation Carried Forward
MedDRA	Medical Dictionary for Regulatory Activities
MELD	Model for End-Stage Liver Disease
MRI	Magnetic Resonance Imaging
MTD	Maximum Tolerated Dose
NCI	National Cancer Institute
PPS	Per Protocol Set
PT	Preferred Term
SAP	Statistical Analysis Plan
SAS	Statistical Analysis System
SOC	System Organ Class
SS	Safety Set
TEAE	Treatment-Emergent Adverse Event
TP	Treponema Pallidum
VAS	Visual Analogue Scale
WHO	World Health Orgnization

2 INTRODUCTIONS

2.1 Preface

This document is the statistical analysis plan (SAP) for **“A Clinical Study on the Safety and Efficacy of Human Umbilical Cord-Derived Mesenchymal Stem Cells for Treating Cirrhosis in the Decompensated Stage (Climbing Trial)”**, which will provide the details and methods to analyze and report the results of baseline characteristics, efficacy and safety.

This SAP will be finalized and approved before the database is locked, and the programming of the corresponding statistical analysis will be progressively completed as the study data is accumulating

until the database is locked.

Mockup Shell will be provided as an attachment of this SAP.

2.2 Changes to the Protocol-Planned Analysis

The statistical analysis planned in this SAP is consistent with the requirements in the protocol.

3 Purpose of Analysis

Primary Purpose : To assess the safety and tolerability of multiple administrations of VUM02 Injection in patients with decompensated cirrhosis and to determine the safe dose for clinical use.

Secondary Purpose : To evaluate the preliminary efficacy of VUM02 Injection in patients receiving multiple doses, and to provide a basis for designing subsequent clinical trial protocols and determining appropriate dosing for cell therapy.

4 Study Design

4.1 Overall Study Design

The study is designed as a single-center, dose-escalation, single-arm study. Subjects with decompensated cirrhosis will be screened as the target disease for the clinical investigation. The study follows the ‘3+3’ dose-escalation rule, with 12 to 24 participants expected to be enrolled, and 3 to 6 subjects in each of the low, medium, high, and ultra-high dose groups.

In this study, dose escalation will be conducted according to the ‘3+3’ rule, where each subject will be assigned to a dose group, starting with the low dose group. Each subject received the corresponding dose, followed by close monitoring and assessment for potential dose-limiting toxicities (DLTs) in 7 days. Subjects must complete a 7-day DLT evaluation before the next subject in the same group can begin dosing. Dosing in the subsequent dose group can only proceed after all subjects in the current group have completed a 28 days assessment of safety after first dose. Throughout the dose escalation process, the investigator and sponsor will evaluate the safety data from the previous dose group to determine whether to proceed with dosing in the next dose level.

During the DLT observation period, if no dose-limiting toxicities (DLTs) occur in a dose group (0/3), the dose can be escalated to the next level. If 1 DLT (1/3) occurs in a dose group, the group must be supplemented with 3 additional subjects. If none of the 3 supplementary subjects experience DLTs (total DLT 1/6), the dose can be escalated to the next group. However, if 1 or more DLTs occur among the 3 supplemented subjects (total DLT ⩾ 2/6), dose escalation will be halted, and the previous dose level will be considered the maximum tolerated dose (MTD). If the highest dose is reached without any DLTs, this dose will be considered the MTD, and the decision to continue dose escalation will be made through a discussion between the investigator and the sponsor.

Safety Observations：

①The results of vital signs, physical examination, hematology, serum chemistry, coagulation, blood ammonia, and electrocardiogram (ECG) will be collected from all subjects at the screening period, day 1, day 7, day 14, day 28, and at month 2, month 3, month 6, month 12, month 18, and month 24..

② The results of urine routine, stool routine (including occult blood will be collected from all subjects at the screening phase, on day 1, day 14, day 28, and at month 2, month 3, month 6, month 12 month 18 and month 24.

③The results of alpha-fetoprotein (AFP) tests will be collected from all subjects at the screening phase, on day 1, day 14, day 21, day 28, and at month 2, month 3, month 6, month 12 month 18 and month 24.

④The results of abdominal ultrasound will be collected from all subjects during the screening phase, as well as on day 1, day 21, day 28, and at month 2, month 3, month 6, month 12 month 18 and month 24.

⑤Hepatitis B penta/Hepatitis C Virus (HCV) antibodies, upper abdominal Computed Tomography (CT) and Magnetic Resonance Imaging (MRI) with and without contrast, and quantification of nucleic acids will be collected from all subjects at the Screening period, and at month 6, month 12, month 18, and month 24.

⑥ Chest radiographs/computed tomography (CT) scans of the chest will be collected from all subjects during the screening phase, at month 12,month 24.

⑦FibroScan results will be collected from all subjects at screening, on day 28 and at month 3.

⑧Gastroscopy results will be collected from all subjects at the Screening period, and at month 6.

⑨ Adverse events, serious adverse events, concomitant medications, and non-pharmacological treatment occurring during the trial will be collected.

Efficacy Observations：

①The Model for End-Stage Liver Disease (MELD) scores, along with the Child-Pugh scores, will be collected from all subjects at screening, on day 1, day 7, day 14, day 21, day 28, and at month 2,month 3, month 6.

②Quality of life assessments will be collected from all subjects at screening, day 14, day 21, day 28, and at month 2,month 3, month 6, month 1, month 18, and month 24. the assessments included three scales: the alcohol dependence scale, the chronic liver disease questionnaire, and the 5-Level EuroQoL Five Dimensions Questionnaire (EQ-5D-5L).

③ The occurrence of complications related to decompensated cirrhosis, liver transplantation, and hepatocellular carcinoma will be collected in subjects throughout the trial.

This study will be conducted by Chinese PLA General Hospital。

4.2 Randomisation

Not applicable.

4.3 Sample Size

This trial is an exploratory study. Subjects with decompensated cirrhosis will be screened as the disease type for the clinical study. A total of 6 to 12 subjects are expected to be enrolled, with 3 to 6 subjects in each of the low and high dose groups.

5 STUDY ENDPOINTS

5.1 Endpoints of Safety

(1)DLT Events

Adverse events in the trial will be assessed using the National Cancer Institute (NCI) Common Terminology Criteria for Adverse Events (CTCAE) version 5.0 grading scale. The following drug-related adverse events will be considered as DLT:

(1) Grade ≥3 allergic reactions related to UC-MSCs, such as dyspnea, chills and fever; (2) Grade ≥3 embolic adverse events related to UC-MSCs, such as acute pulmonary embolism, deep vein thrombosis and other embolic events, etc.; (3) Grade ≥3 hematological toxicity related to UC-MSCs; (4) Any unexpected toxicity requiring discontinuation of treatment at the discretion of the investigator and sponsor.

(2)Adverse Events

(3)Laboratory Examinations

- **Hematology**： including leukocytes, absolute neutrophil count, absolute lymphocyte count, absolute monocyte count, absolute eosinophil count, absolute basophil count, erythrocytes, hemoglobin, hematocrit, mean corpuscular volume (MCV), mean corpuscular hemoglobin (MCH), platelets, erythrocyte hemoglobin concentration；
- **Serum Chemistry**： including alanine aminotransferase (ALT), aspartate aminotransferase (AST), albumin, globulin, total bilirubin, direct bilirubin, total bile acids, glucose, urea, creatinine, alkaline phosphatase, γ-glutamyltransferase (GGT), potassium, sodium, chloride, creatine kinase (CK), lactate dehydrogenase (LDH), prealbumin, cholinesterase, total cholesterol, triglycerides, high-density lipoprotein (HDL) cholesterol, low-density lipoprotein (LDL) cholesterol；
- **Urine Routine**：including pH, specific gravity, glucose, protein, ketone bodies, erythrocytes,

leukocytes, leukocyte esterase (LEU), erythrocyte esterase (LEU)；

- **Coagulation**： including Prothrombin time, international normalized ratio, activity, activated partial thromboplastin time, fibrinogen, thrombin time, activated partial thromboplastin ratio；
- **Stool Routine (including Occult Blood)**： including fecal erythrocytes, fecal leukocytes, occult blood；
- **Blood Ammonia**。

(4)ECG

(5)Physical Examinations

Including general condition, skin, lymph nodes, head and neck region, chest, abdomen, musculoskeletal system, nervous system, others.

(6)Vital Signs

Including temperature, respiration rate, pulse rate, systolic blood pressure, diastolic blood pressure, body height, body weight.

(7)Fibroscan Examination

(8)Quantification of Nucleic Acids

Including hepatitis B virus nucleic acid, hepatitis C virus nucleic acid.

(9)AFP

(10)Abdominal Ultrasound

(11)Hepatitis B penta/Hepatitis C Virus (HCV) Antibodies

Including hepatitis B surface antigen, hepatitis B surface antibody, hepatitis B e antigen, hepatitis B e antibody, hepatitis B core antibody, hepatitis C antibody.

(12)Gastroscopy

(13)Other

Including CT/MRI plain and contrast-enhanced imaging of the upper abdomen, chest x-ray/chest CT.

5.2 Endpoints of Efficacy

- **Model for End-Stage Liver Disease (MELD) scores and their changes from baseline at each visit:** including MELD scores and the changes from baseline at visits on day 7, day 14, day 21, day 28, month 2, month 3, month 6.
- **Child-Pugh score**

Child-Pugh total score and its change from baseline at day 7, day 14, day 21, day 28, month 2, month 3, month 6.

Grading of the Child-Pugh total score and its change from baseline at day 7, day 14, day 21, day 28, month 2, month 3, month 6.

The proportion of subjects with different scores in the individual Child-Pugh components and their change from baseline at day 7, day 14, day 21, day 28, month 2, month 3, month 6.

The scoring criteria for individual items are as follows: The scoring criteria for individual items are as follows:

Criteria	1 point	2 point	3 point
Encephalopathy(Grade)	None	Grade I-II	Grade III-IV
Ascites	None	Mild	Moderate to Severe
Bilirubin (Total)(umol/L)	<34	34－51	>51
Albumin(g/L)	>35	28-35	<28
Prothrombin time(s)	<4	4-6	>6

➢ **Alcohol Dependence Scale**

Total scores on the alcohol dependence scale and their changes from baseline at visits on day 14, day 21, day 28, month 2,month 3, month 6, month 12, month 18, and month 24.

distribution of clinical grades and their changes from baseline at visits on day 14, day 21, day 28, month 2,month 3, month 6, month 12, month 18, and month 24.

the scores for all items of the alcohol dependence scale totalled 47 points, which will be categorized into five clinical grades based on the total score. The criteria for evaluating the individual scores as well as the clinical grades are outlined below:

Standard Category	Entry	Criterion
Individual scoring criteria	1. How much did you drink the last time you drank?	scored 0. Enough to get high or less, scored 1. Enough to get drunk, scored 2. Enough to pass out
	2. Do you often have hangovers on Sunday or Monday mornings?	scored 0. No, scored 1. Yes
	3. Have you had the "shakes" when sobering up (hands tremble, shake inside)?	scored 0. No, scored 1. Sometimes, scored 2. Often
	4. Do you get physically sick (e.g., vomit, stomach cramps) as a result of drinking?	scored 0. No, scored 1. Sometimes, scored 2. Almost every time I drink
	5. Have you had the "DTs" (delirium tremens) – that is, seen, felt or heard things not really there; felt very anxious, restless, and over excited?	scored 0. No, scored 1. Sometimes, scored 2. Several times
	6. When you drink, do you stumble about, stagger, and weave?	scored 0. No, scored 1. Sometimes, scored 2. Often
	7. As a result of drinking, have you felt overly hot and sweaty (feverish)?	scored 0. No, scored 1. Once, scored 2. Several times
	8. As a result of drinking, have you seen things that were not really there?	scored 0. No, scored 1. Once, scored 2. Several times
	9. Do you panic because you fear you may not have a drink when you need it?	scored 0. No, scored 1. Yes
	10. Have you had blackouts ("loss of memory" without passing out) as a result of drinking?	scored 0. No, never, scored 1. Sometimes, scored 2. Often, scored 3. Almost every time I drink
	11. Do you carry a bottle with you or keep one close at hand?	scored 0. No, scored 1. Some of the time, scored 2. Most of the time
	12. After a period of abstinence (not drinking), do you end up drinking heavily again?	scored 0. No, scored 1. Sometimes, scored 2. Almost every time I drink
	13. In the past 12 months, have you passed out as a result of drinking?	scored 0. No, scored 1. Once, scored 2. More than once
	14. Have you had a convulsion (fit) following a period of drinking?	scored 0. No, scored 1. Yes, scored 2. Several times
	15. Do you drink throughout the day?	scored 0. No, scored 1. Yes
	16. After drinking heavily, has your thinking been fuzzy or unclear?	scored 0. No, scored 1. Yes, but only for a few hours, scored 2. Yes, for one or two days, scored 3. Yes, for many days
	17. As a result of drinking, have you felt your heart beating rapidly?	scored 0. No, scored 1. Yes
	18. Do you almost constantly think about drinking and alcohol?	scored 0. No, scored 1. Yes
	19. As a result of drinking, have you heard "things" that were not really there?	scored 0. No, scored 1. Yes, scored 2. Several times

	20. Have you had weird and frightening sensations when drinking?	scored 0. No, scored 1. Once or twice, scored 2. Often
	21. As a result of drinking have you "felt things" crawling on you that were not really there (e.g., bugs, spiders)?	scored 0. No, scored 1. Yes, scored 2. Several times
	22. With respect to blackouts (loss; of memory)?	scored 0. Have never had a blackout<br>scored 1. Have had blackouts that last less than an hour<br>scored 2. Have had blackouts that last for several hours<br>scored 3. Have had blackouts that last a day or more
	23. Have you tried to cut down on your drinking and failed?	scored 0. No, scored 1. Once, scored 2. Several times
	24. Do you gulp drinks (drink quickly)？	scored 0. No, scored 1. Yes
	25. After taking one or two drinks, can you usually stop?	scored 0. Yes, scored 1. No
Clinical Level Criteria	Grade 1：No evidence of alcohol dependence was reported.	ADS Raw Score：0
	Grade 2：Low level of alcohol dependence.	ADS Raw Score：1-13
	Grade 3：Intermediate level of alcohol dependence.	ADS Raw Score：14-21
	Grade 4：Substantial level of alcohol dependence.	ADS Raw Score：22-30
	Grade 5：Severe level of alcohol dependence.	ADS Raw Score：31-47

➢ **Chronic Liver Disease Questionnaire**

Total scores on the chronic liver disease questionnaire and their changes from baseline at visits on day 14, day 21, day 28, month 2,month 3, month 6, month 12, month 18, and month 24.

Total scores for each domain of the chronic liver disease questionnaire and their changes from baseline at visits on day 14, day 21, day 28, month 2,month 3, month 6, month 12, month 18, and month 24.

The criteria for evaluating individual scores, as well as those for assessing each domain, are outlined below.

Scale or factor	Entry	Criteria
Individual scoring criteria	All of the time	Score 1
	Most of the time	Score 2
	A good bit of the time	Score 3
	Some of the time	Score 4
	A little of the time	Score 5
	Hardly any of the time	Score 6
	None of the time	Score 7
Domain scoring criteria	Items 1：Abdominal symptoms(AS)	question 1，5，17
	Items 2：Fatigue(FA)	question 2，4，8，11，13
	Items 3：Systemic symptoms(SS)	question 3，6，21，23，27
	Items 4：Activity(AC)	question 7，9，14
	Items 5：Emotional function(EF)	question 10，12，15，16，19，20，24，26
	Items 6：Worry(WO)	question 18，22，25，28，29

➢ **EQ-5D-5L Scale**

The index values of the EQ-5D-5L scales and their changes from baseline scores will be calculated on day 14, day 21, day 28, month 2,month 3, month 6, month 12, month 18, and month 24 visits according to Chinese standards.

index value = 1-(factor of mobility + factor of self-care + factor of usual activities + factor of pain/discomfort + factor of anxiety/depression).

	Level of value set				
	Level 1	Level 2	Level 3	Level 4	Level 5
MOBILITY	0	0.066	0.158	0.287	0.345
SELF-CARE	0	0.048	0.116	0.210	0.253
USUAL ACTIVITIES	0	0.045	0.107	0.194	0.233
PAIN / DISCOMFORT	0	0.058	0.138	0.252	0.302

	Level of value set				
	Level 1	Level 2	Level 3	Level 4	Level 5
ANXIETY / DEPRESSION	0	0.049	0.118	0.215	0.258

The Visual Analogue Scale (VAS) of the EQ-5D-5L scale and the change from baseline score will be calculated at the day 14, day 21, day 28, month 2,month 3, month 6, month 12, month 18, and month 24 visits.

➢ **Incidence of Complications associated with Decompensated Cirrhosis**

The proportion of subjects who will experience complications related to decompensated cirrhosis following the infusion of the investigational drug, as well as the proportion of subjects experiencing each specific complication.

➢ **Survival without Liver Transplantation**

The proportion of subjects who remain alive without undergoing liver transplantation after receiving the investigational drug infusion.

➢ **Liver Failure**

The proportion of subjects with liver failure after receiving the investigational drug infusion.

➢ **Liver Cancer**

The proportion of subjects with liver cancer after receiving the investigational drug infusion.

6 ANALYSIS SETS

(1)Full Analysis Set(FAS)： According to the principle of Intention to Treat (ITT) will constitute the Full Analysis Set for all subjects who were successfully enrolled and had at least one documented treatment.

Demographic and baseline characteristics, as well as efficacy endpoints, will be statistically analyzed based on the FAS.

(2)Per Protocol Set(PPS)： It is a subset of FAS. subjects who meet the inclusion criteria, not meet the exclusion criteria, complete the treatment regimen, fully adhere to the trial protocol, and not have any major protocol violations (subjects who will discontinue trial treatment due to disease progression will be included in the Per-Protocol Set, PPS).

Efficacy endpoints will be statistically analyzed based on the PPS.

(3)Dose Limited Toxicity Set(DLTS)： Includes all subjects who have received an infusion and completed the DLT observation period(or had a DLT event). The DLT analysis set will be used primarily for DLTS evaluation.

(4)Safety Set(SS)： Includes all subjects who have received an infusion. The safety population will primarily be used for the analysis of safety data.

Safety endpoints will be statistically analyzed based on the SS.

The above analysis datasets will be reviewed and discussed by the principal investigator, sponsor, statistician, and data management team in a data review session prior to the database lock.

7 STATISTICAL METHOD

7.1 General Considerations

7.1.1 General Method

➢ **Descriptive Analysis**

Unless otherwise specified, the following descriptive statistics are given according to the variable types:

- ✓ Continuous variables will be summarized by mean, standard deviation, median, minimum and maximum.
- ✓ Categorical or ordinal variables will be summarized by frequency counts and percentages, where the denominator will be the number of participants in the corresponding analysis set.

➢ **Decimal places**

Unless otherwise specified, the number of decimal points in the statistical analysis report will be performed according to the following rules:

- ✓ The minimum and maximum values are consistent with the maximum decimal number of the original data.
- ✓ The number of decimal points of median, mean, standard deviation and 95% CI is 1 place more than the maximum decimal number of the original data.
- ✓ Percentages, Rates, rate differentials are retained to 2 decimal places.
- ✓ If the P-value is ≥0.0001, it is retained to 4 decimal places; if the P-value is <0.0001, it is reported as “<0.0001”.
- ✓ Test statistics for all statistical tests are retained to 3 decimal places.
- ✓ Derived data is retained to 2 decimal places.

7.1.2 Related Definitions and Derivation Rules

➢ **Baseline**

Baseline is defined as the last non-missing value before the first dose.

➢ **Conversion among year, month and day**

Month = days /30.4375, year = days /365.25, rounded to one decimal.

- **Study Days**

The study start date is defined as the first infusion date. The study day corresponding to a visit or event will be calculated using the following formula, with the study start date as the reference point:

✓ If the event date occurs before the study start date, study day = event date – study start date;

✓ if the event date occurs on or after the study start date, study day = event date – study start date +1.

- **Adverse Event**

Adverse Event Occurrence Time (Days) = Adverse Event Occurrence Date – Infusion Date.
Adverse Event Duration (Days) = Adverse Event End Date – Adverse Event Start Date + 1.

- **Treatment Emergent Adverse Event(TEAE)**

A TEAE (Treatment-Emergent Adverse Event) is defined as an adverse event that occurs on or after the first infusion (including the same day), or one that occurs prior to the infusion but is exacerbated after the infusion. The following rules are applied during programming:

✧ If the adverse event occurs after (or on the same day as) the start of treatment with the investigational drug, it will be counted as a TEAE;

✧ If the adverse event occurs before the start of treatment with the investigational drug, but its severity worsens after treatment, it will be counted as a TEAE;

✧ If the adverse event or the start time of treatment with the investigational drug is missing, and it is not possible to clearly determine the relationship between the occurrence of the adverse event and the start of treatment, the event will be counted as a TEAE for all purposes;

✧ If the adverse event occurs prior to the start of treatment with the investigational drug, but the severity is missing and it cannot be determined whether the severity worsened after treatment, the event will be counted as a TEAE.

Due to varying severity, multiple observations of the same adverse event may be recorded in the Clinical Trial Electronic Data Capture (EDC) system. The following rules are applied to determine whether each observation of these adverse event qualifies as a TEAE:

(1)If the start time of all observations for the adverse event is after (or on the same day as) the first dose of the investigational drug, all observations for that adverse event will be classified as TEAEs;

(2)If any observation starts before the first dose of the investigational drug but ends after (or on the same day as) the first dose, the severity of this observation will serve as the baseline. For all subsequent observations with a start time after the first dose, the severity will be compared to this reference point. If the severity has increased, the observation will be classified as a TEAE; if the severity remains the same or decreases, the observation will be classified as a non-TEAE.

According to the above rules, multiple TEAEs may be recorded for the same adverse event. However, when calculating instances, only one TEAE will be counted per adverse event, with the severity being determined by the highest severity level recorded among the TEAEs.

- **Prior Medication and Concomitant Medication**

"Prior Medication" refers to medications that will be discontinued before the first infusion. "Concomitant Medication" refers to medications that: (1) totalled initiated before the first infusion and will be still being used at the time of the first infusion, or (2) will be initiated on or after the first infusion date.

- **Relevance of Adverse Events**

An adverse event is considered relevant to the study drug if its relationship is classified as “Definitely related”, “Highly Likely Relevant”, “Possibly related”, or if the relevance is missing. An adverse event is considered unrelated to the study drug if its relationship is classified as “Possibly Not Relevant”, “Definitely Irrelevant”.

- **Coding**

Medical history or newly emerging diseases, adverse events and serious adverse events will be coded according to the Medical Dictionary for Regulatory Activities (MedDRA), version 26.0 or later. Prior /concomitant medication will be coded according to the Anatomical Therapeutic Chemical Classification (ATC).

7.1.3 Analysis Window

For visits after baseline, all analyses will be conducted based on the scheduled visits in the protocol. Results of both scheduled and unscheduled visits will be listed.

7.1.4 Analysis Software

All statistical analysis will be conducted by SAS 9.4 or later version.

7.1.5 Table and Listing

- **Table**

All data will be summarized by treatment (i.e., low dose group, high dose group). The treatments will be presented in columns.

- **Listing**

Unless otherwise specified, all listings will include group, subject ID. SDTM data will be preferred to presented in the listings.

7.2 Disposition of Participants

Summarize separately the number of subjects screened, percentage and number of subjects enrolled and completed the trial, and included in each analysis set, and analyze the reasons for subject withdrawals.

Provide separate lists of subjects who screened failed and who withdrew from the trial and those who will be not included in each analysis set.

Statistically describe the protocol deviations for each group, both in total and by category. Provide a list of protocol deviations/violations. Listing of programme deviations.

7.3 Demographics and Clinical Characteristics

The following demographic and baseline characteristics will be statistically summarized for each group:

- Demographic characteristics(including age, sex, ethnicity);
- Clinical characteristics at screening (including disease duration, allergy history, family history, smoking history, alcohol use, substance abuse history);
- Baseline physical examination (including general condition, skin, lymph nodes, head and neck region, chest, abdomen, musculoskeletal system, nervous system, and other);
- Baseline vital signs (temperature, respiratory rate, pulse rate, systolic blood pressure, diastolic blood pressure, body height, body weight);

Disease duration (months) = (date of informed consent signing - date of first diagnosis + 1) / 30.4375.

Prior medication use will be coded according to the ATC classification. The number of occurrences, number of cases, and frequency of use after infusion will be calculated for each group, categorized by the ATC classification.

Medical history and current diseases will be categorized according to System Organ Class (SOC) and Preferred Term (PT), and the frequency of occurrences, number of cases, and incidence rates will be calculated for each group.

Separate lists will be generated for the following: demographic data, allergy history, family history, smoking history, alcohol consumption history, substance abuse history, HIV/TP antibody, past medical history and treatments, current diseases and concomitant treatments, and other treatments,.

Demographic data and baseline characteristics will be analyzed based on the FAS.

7.4 Concomitant Medication

Concomitant medication will be coded according to ATC. The number and percentage of subjects who have concomitant medication and usage frequency will be tabulated by ATC1 and ATC2, and a list of concomitant medications will be generated.

The above analysis is based on SS.

7.5 Hypothesis

It is an exploratory clinical study and no formal statistical tests will be performed.

7.6 Safety Evaluation

7.6.1 DLT Occurrence

The incidence of DLT will be calculated for each group, and its 95% confidence interval will be calculated by the Clopper-Pearson method.

Descriptive statistics will be performed for the time from infusion to the occurrence of DLT for each dose group, including the calculation of the mean, standard deviation, median, Q1, Q3, minimum, and maximum values.

A list of subjects experiencing DLT will be compiled.

7.6.2 Adverse Events

Adverse events will be medically coded using MedDRA version 26.0 or the latest version. They will be categorized and counted according to the two levels of System Organ Class (SOC) and Preferred Term (PT). The number of occurrences, number of cases, and incidence rates for each type of adverse event will be calculated separately for each group in the following categories:

- All adverse events;
- Adverse events related to the study drug;
- Adverse events leading to discontinuation of the study drug;
- Adverse events related to the study drug leading to discontinuation of the study drug;
- Adverse events with severity ≥ Grade 3;
- Adverse events with severity ≥ Grade 3 related to the study drug;
- Adverse events leading to withdrawal from the trial;
- Adverse events related to the study drug leading to withdrawal from the trial;
- Serious adverse events;
- Serious adverse events related to the study drug;
- Adverse events leading to death;
- Adverse events leading to death related to the study drug.

Separate lists will be generated for adverse events, adverse events leading to withdrawal from the trial, adverse events leading to death, and serious adverse events.

7.6.3 Laboratory Tests

The test values of various laboratory tests (including routine blood tests, blood biochemistry, and coagulation function indicators) and their changes from baseline to post-treatment time points (until the end of treatment or early withdrawal of subjects from the trial) will be statistically described for

each group separately. The number of subjects, mean, standard deviation, median, minimum, and maximum values will be calculated.

For each group, the results of various laboratory tests (including routine blood tests, blood biochemistry, urinalysis, coagulation function, stool analysis with occult blood, and blood ammonia levels) will be presented in the form of shift tables comparing pre- and post-treatment values. This comparison will be based on normal reference ranges and the investigator's judgment of clinical significance, showing the change in the worst results from pre-treatment to post-treatment (up to the end of treatment or until withdrawal from the trial).

The lists of laboratory tests will be compiled.。

7.6.4 ECG

For each group, the results of ECG will be presented in the form of shift tables comparing pre- and post-treatment values. This comparison will be based on normal reference ranges and the investigator's judgment of clinical significance, showing the change in the worst results from pre-treatment to post-treatment (up to the end of treatment or until withdrawal from the trial).

The list of ECG will be compiled.。

7.6.5 Physical Examinations

For each group, the results of various physical examinations (Including General condition, skin, lymph nodes, head and neck region, chest, abdomen, musculoskeletal system, nervous system, others) will be presented in the form of shift tables comparing pre- and post-treatment values. This comparison will be based on normal reference ranges and the investigator's judgment of clinical significance, showing the change in the worst results from pre-treatment to post-treatment (up to the end of treatment or until the withdrawal from the trial).

The list of physical examinations will be compiled.。

7.6.6 Vital Signs

The test values of various vital signs (including temperature, respiration rate, pulse rate, systolic blood pressure, diastolic blood pressure, body weight) and their changes from baseline to post-treatment time points (until the end of treatment or early withdrawal of subjects from the trial) will be statistically described for each group separately. The number of subjects, mean, standard deviation, median, minimum, and maximum values will be calculated.

The list of vital signs will be compiled.。

7.6.7 Fibroscan Examination

For each group, the results of fibroscan examination will be presented in the form of shift tables comparing pre- and post-treatment values. This comparison will be based on normal reference ranges and the investigator's judgment of clinical significance, showing the change in the worst results from pre-treatment to post-treatment (up to the end of treatment or until the withdrawal from the trial).

The list of fibroscan examination will be compiled.。

7.6.8 Quantification of Nucleic Acids

For each group, the results of various quantification of nucleic acids (including hepatitis B virus nucleic aacid, hepatitis C virus nucleic acidv) will be presented in the form of shift tables comparing pre- and post-treatment values. This comparison will be based on normal reference ranges and the investigator's judgment of clinical significance, showing the change in the worst results from pre-treatment to post-treatment (up to the end of treatment or until the withdrawal from the trial).

The list of quantification of nucleic acids will be compiled.。

7.6.9 AFP

For each group, the results of AFP will be presented in the form of shift tables comparing pre- and post-treatment values. This comparison will be based on normal reference ranges and the investigator's judgment of clinical significance, showing the change in the worst results from pre-treatment to post-treatment (up to the end of treatment or until the withdrawal from the trial).

The list of AFP will be compiled.。

7.6.10 Abdominal Ultrasound

For each group, the results of abdominal ultrasound will be presented in the form of shift tables comparing pre- and post-treatment values. This comparison will be based on normal reference ranges and the investigator's judgment of clinical significance, showing the change in the worst results from pre-treatment to post-treatment (up to the end of treatment or until the withdrawal from the trial).

The list of abdominal ultrasound will be compiled.。

7.6.11 Hepatitis B penta/Hepatitis C Virus (HCV) Antibodies

For each group, the results of various hepatitis B penta/HCV antibodies (including hepatitis B surface antigen, hepatitis B surface antibody, hepatitis B e antigen, hepatitis B e antibody, hepatitis B core antibody, hepatitis C antibody) will be presented in the form of shift tables comparing pre- and post-treatment values. This comparison will be based on normal reference ranges and the investigator's judgment of clinical significance, showing the change in the worst results from pre-treatment to post-treatment (up to the end of treatment or until the withdrawal from the trial).

The list of hepatitis B penta/HCV antibodies will be compiled.。

7.6.12 Gastroscopy

For each group, the results of gastroscopy will be presented in the form of shift tables comparing pre- and post-treatment values. This comparison will be based on normal reference ranges and the investigator's judgment of clinical significance, showing the change in the worst results from pre-treatment to post-treatment (up to the end of treatment or until the withdrawal from the trial).

The lists of gastroscopy will be compiled.。

7.6.12 Other

For each group, the results of CT/MRI plain and contrast-enhanced imaging of the upper abdomen, chest X-ray/chest CT will be presented in the form of shift tables comparing pre- and post-treatment values. This comparison will be based on normal reference ranges and the investigator's judgment of clinical significance, showing the change in the worst results from pre-treatment to post-treatment (up to the end of treatment or until the withdrawal from the trial).

The lists of CT/MRI plain and contrast-enhanced imaging of the upper abdomen, chest X-ray/cchest CT will be compiled.。

7.7 Efficacy Evaluation

(1) Model for End-Stage Liver Disease (MELD) scores and their changes from baseline at each visit

Descriptive statistics of the Model for End-Stage Liver Disease (MELD) scores and their changes from baseline will be provided for day 7, day 14, day 21, day 28, month 2, month 3, month 6.

(2) Child-Pugh score

Descriptive statistics of the model for Child-Pugh total score and their changes from baseline will be provided for day 7, day 14, day 21, day 28, month 2, month 3, month 6.

Descriptive statistics of the Child-Pugh total score grading and its changes from baseline, including the number of subjects and percentages, will be provided for each group of subjects at day 7, day 14, day 21, day 28, month 2, month 3, month 6.

Descriptive statistics of the individual components of the Child-Pugh score grading and its changes from baseline, including the number of subjects and percentages, will be provided for each group of subjects at day 7, day 14, day 21, day 28, month 2, month 3, month 6.

(3) Alcohol Dependence Scale

Descriptive statistics of the model for total scores of alcohol dependence scale and their changes from baseline will be provided for day 14, day 21, day 28, month 2,month 3, month 6, month 12, month 18, and month 24.

Descriptive statistics of the clinical grade distribution of the alcohol dependence scale and its

changes from baseline, including the number of subjects and percentages, will be provided for each group of subjects at day 14, day 21, day 28, month 2,month 3, month 6, month 12, month 18, and month 24.

(4) Chronic Liver Disease Questionnaire

Descriptive statistics of the model for total scores of chronic liver disease questionnaire and their changes from baseline will be provided for day 14, day 21, day 28, month 2,month 3, month 6, month 12, month 18, and month 24.

Descriptive statistics of the model for domains scores of chronic liver disease questionnaire and their changes from baseline will be provided for day 14, day 21, day 28, month 2,month 3, month 6, month 12, month 18, and month 24.

(5) EQ-5D-5L Scale

Descriptive statistics of the model for index value scores of EQ-5D-5L Scale and their changes from baseline will be provided for day 14, day 21, day 28, month 2,month 3, month 6, month 12, month 18, and month 24.

Descriptive statistics of the model for VAS scores of EQ-5D-5L Scale and their changes from baseline will be provided for day 14, day 21, day 28, month 2,month 3, month 6, month 12, month 18, and month 24.

(6) Incidence of complications associated with decompensated cirrhosis

The total incidence of complications associated with decompensated cirrhosis and the incidence of each individual complication in each group will be calculated, with 95% confidence intervals by using the Clopper-Pearson method.

(7) Survival without liver transplantation

The incidence of survival without liver transplantation in each group will be calculated, with 95% confidence intervals by using the Clopper-Pearson method.

(8) Liver Cancer

The incidence of liver cancer in each group will be calculated, with 95% confidence intervals by using the Clopper-Pearson method.

7.8 Handling of Missing Data

In this trial, if any individual score required to calculate the total score for each domain of the chronic liver disease questionnaire or the EQ-5D-5L index value was missing, the corresponding derived endpoint will be treated as missing.

For the statistical analysis of the Full Analysis Set (FAS), the trial will employ the Last Observation Carried Forward (LOCF) method to handle missing data for the MELD score, Child-Pugh total score,

the grading of the Child-Pugh score, the total score of the alcohol dependence scale and the distribution of clinical grades from the alcohol dependence scale alcohol dependence scale, the total score of the chronic liver disease questionnaire, the domain scores of the chronic liver disease questionnaire, and the EQ-5D-5L utility index values, as well as the EQ-5D-5L VAS score.
Missing data for other efficacy and safety outcomes will be not addressed in this trial.

7.9 Subgroup Analysis

Not Applicable.

7.10 Multiplicity

The p-values calculated in this trial are nominal p-values, primarily used to characterize the strength of the association between the evaluation endpoints and the treatment groups, and should not be interpreted as a basis for formal statistical inference.

7.11 Interim Analysis

Not Applicable.

8 Notes on this Plan

This analysis plan has been drafted based on the relevant descriptions outlined in the study protocol, which specifies the efficacy and safety evaluation endpoints. Specific statistical methods for the evaluation endpoints have been proposed, considering the basic characteristics of the indicators in the study protocol, as well as the specific requirements of this study. Given the possibility of unforeseen changes in the final data distribution of the clinical trial, the statistical methods may be subject to minor adjustments, and the presentation of the corresponding statistical analysis results may also undergo certain changes.

VERSION HISTORY

Version Number	Version Date	Author	Description of Change
V1.0	20Nov2023	Yin Li	Initial version
