# Supplementary material for: Dose-escalation studies of mesenchymal stromal cell therapy for decompensated liver cirrhosis: phase Ia/Ib results and immune modulation insights
Source: Signal Transduct Target Ther. 2025 Jul 29;10:238. doi: 10.1038/s41392-025-02318-4 (PMC12304480; doi:10.1038/s41392-025-02318-4)
Supplement: Supplementary file 3 — Supplementary Materials [file 41392_2025_2318_MOESM3_ESM.docx]

Supplementary Materials for

**Dose-Escalation Studies of Mesenchymal Stromal Cell Therapy for Decompensated Liver Cirrhosis: Phase Ia/Ib Results and Immune Modulation Insights**

Lei Shi1^*#^, Ziying Zhang^*^, Song Mei^*^, Zerui Wang^*^, Zhe Xu, Weiqi Yao, Limin Liu, Mengqi Yuan, Yuefei Pan, Kaidi Zhu, Kai Liu, Fanglin Meng, Jiao Sun, Wenying Liu, Xiaohui Xie, Tengyun Dong, Lei Huang, Fanping Meng, Jun-Liang Fu, Yuanyuan Li, Chao Zhang, Xing Fan, Ming Shi, Yu Zhang, Yonggang Li, Wei-Fen Xie, Peng Zhang^#^, Fu-Sheng Wang^#^

**Correspondence to:**

Prof. Fu-Sheng Wang, fswang302@163.com

Prof. Lei Shi, shilei302@126.com

Prof. Peng Zhang, peng.zhang@mail.ccmu.edu.cn

**This file includes:**

Supplementary Figures 1 to 4.

Supplementary Tables 1 to 10.

**Other Supplementary Materials for this manuscript include the following:**

| **Supplementary File** | **File title** |
| --- | --- |
| Supplementary File 1 | Clinical Study Protocol |
| Supplementary File 2 | Statistical Analysis Plan (SAP) |

Supplementary Figure 1.

**Supplementary Figure 1. The dynamic single-cell landscape of DLC patients under MSC treatment, related to Fig. 3. a,** t-distributed stochastic neighbor embedding (tSNE) plot of the annotated immune cell lineages in CyTOF. **b,** Boxplot showing the dynamics of four major immune cell lineages in CyTOF. **c,** t-SNE plot of the annotated monocyte subpopulations in CyTOF. **d,** Monocle3 (left) and Slingshot (right) pseudotime trajectories of monocyte differentiation. **e,** Heatmap showing that the expression of genes varied over pseudotime. **f,** Density plots showing the distribution of monocyte subpopulations across pseudoperiods in the healthy cohort and at baseline and posttreatment time points in Cohorts I, II, and IV. **g,** Heatmap of cytokines expressed by monocyte subsets. # indicates a significant difference between baseline and healthy individuals. * indicates a significant difference between timepoints after MSC treatment and baseline. **h,** Boxplot showing the dynamics of MX1^+^ monocytes in groups divided by variation in the Child‒Pugh score. The Wilcoxon rank-sum test was used to test the difference in cell proportion between groups or between posttreatment and baseline in each cohort.

Supplementary Figure 2.

**Supplementary Figure 2. Variation in other immune cells in DLC patients under MSC treatment, related to Fig. 4. a,** UMAP plot of the annotated NK cell subpopulations and the expression of *NCAM1* and *FCGR3A* (left). Boxplots showing the dynamics of major NK cell subpopulations (right). The cell proportions were calculated as the percentage of total NK cells. **b,** tSNE plot of the annotated NK cell subpopulations in CyTOF (left) and boxplot showing the dynamics of the three NK cell subpopulations in CyTOF (right). **c,** UMAP plot of the annotated B-cell subpopulations (left) and boxplots showing the dynamics of major B-cell subpopulations (right). The percentages of total B cells were calculated as percentages of total B cells. **d,** tSNE plot of the annotated B-cell subpopulations in CyTOF (left) and boxplot showing the dynamics of three B-cell subpopulations in CyTOF (right). The Wilcoxon rank-sum test was used to test the difference in cell proportion between groups (*p* values highlighted in red) and between posttreatment and baseline in each cohort (*p* values highlighted in the corresponding cohort color). ***, *p*<0.001. **, *p*<0.01. *, *p*<0.05.

Supplementary Figure 3.

**Supplementary Figure 3. Interaction between various monocytes and other immune cell subpopulations, related to Fig. 4. a,** Dot plots showing the outgoing and incoming interaction strengths of cell subpopulations that differed between DLC patients at baseline and healthy individuals. **b-c,** The strength of the galectin (b) and resistin (c) pathways at the healthy, baseline, and posttreatment time points in each cohort. **d,** Heatmaps showing the strength of ligand‒receptor pairs between c38_Mono_MX1 and four other B-cell subpopulations: healthy, baseline, D3, and D7.

Supplementary Figure 4

**Supplementary Figure 4. Representative data of viable cell analysis of MSCs prior to intravenous infusion.** a, Representative flow cytometric plots showing the vitality assessment of MSCs. b, Representative flow cytometric plots illustrating the cell phenotype identification of MSCs. c, Detailed results of cell viability for each batch of MSCs prior to infusion.

Supplementary Table 1. Clinical outcome indicators of Phase Ia within 28 days.

|  | **Cohort I (n=3)** | **Cohort II (n=3)** | **Cohort III (n=6)** | **Cohort IV (n=3)** |
| --- | --- | --- | --- | --- |
| **MELD score** |  |  |  |  |
| D3 | 12.67 (3.21) | 17.00 (0.00) | 11.50 (3.94) | 11.67 (3.06) |
| D7 | 13.33 (4.62) | 16.00 (0.00) | 12.17 (4.75) | 11.33 (2.31) |
| D14 | 13.00 (2.65) | 17.33 (2.52) | 12.50 (5.01) | 12.33 (5.13) |
| D28 | 13.67 (3.51) | 17.67 (2.08) | 12.50 (5.01) | 11.33 (3.21) |
| **Child-Pugh score** |  |  |  |  |
| D3 | 6.7 (1.2) | 9.0 (1.7) | 7.7 (1.8) | 7.0 (1.0) |
| D7 | 7.7 (0.6) | 8.3 (1.5) | 7.8 (1.5) | 6.3 (1.5) |
| D14 | 7.0 (0.0) | 7.7 (1.2) | 7.8 (1.2) | 6.7 (1.2) |
| D28 | 7.0 (1.0) | 9.3 (1.2) | 7.8 (1.3) | 6.7 (1.5) |
| **EQ-5D-5L scale utility index** |  |  |  |  |
| D14 | 0.9643 (0.0618) | 1.0000 (0.0000) | 0.5790 (0.4299) | 0.7897 (0.1870) |
| D28 | 1.0000 (0.0000) | 0.9643 (0.0618) | 0.5693 (0.4736) | 0.8563 (0.0635) |
| **EQ-5D-5L scale VAS Score** |  |  |  |  |
| D14 | 73.3 (20.8) | 90.0 (0.0) | 66.3 (30.4) | 61.7 (7.6) |
| D28 | 76.7 (15.3) | 85.0 (5.0) | 61.2 (32.0) | 63.3 (5.8) |
| **Chronic Liver Disease Questionnaire Score** |  |  |  |  |
| D14 | 175.0 (28.8) | 194.3 (5.1) | 156.2 (37.9) | 121.3 (23.5) |
| D28 | 175.7 (35.0) | 177.7 (21.5) | 143.3 (43.7) | 133.7 (12.3) |
| **Albumin (g/L)** |  |  |  |  |
| D3 | 37.33 (1.53) | 33.33 (6.11) | 34.17 (3.06) | 33.00 (1.00) |
| D7 | 35.00 (1.00) | 33.67 (5.69) | 34.83 (3.25) | 34.00 (4.00) |
| D14 | 35.67 (3.06) | 33.67 (3.21) | 36.67 (3.50) | 34.00 (1.00) |
| D28 | 35.60 (1.22) | 32.00 (4.00) | 33.83 (4.17) | 33.67 (4.16) |
| **Prealbumin (g/L)** |  |  |  |  |
| D3 | 95.0 (33.8) | 36.0 (8.7) | 68.7 (27.9) | 112.0 (64.7) |
| D7 | 94.0 (41.2) | 42.7 (21.4) | 71.7 (34.3) | 117.7 (69.6) |
| D14 | 107.3 (27.5) | 41.0 (3.0) | 73.3 (29.1) | 112.3 (50.8) |
| D28 | 125.7 (17.6) | 38.5 (4.9) | 74.0 (31.5) | 116.3 (72.5) |
| **Total bilirubin (μmol/L)** |  |  |  |  |
| D3 | 37.67 (18.03) | 52.77 (8.40) | 35.75 (18.17) | 34.23 (22.19) |
| D7 | 43.03 (19.85) | 49.67 (12.12) | 36.95 (15.93) | 34.60 (17.90) |
| D14 | 38.40 (12.90) | 54.70 (17.68) | 47.50 (30.11) | 41.57 (35.71) |
| D28 | 45.77 (16.20) | 62.87 (9.27) | 46.15 (26.65) | 32.57 (20.26) |
| **Lactic dehydrogenase (U/L)** |  |  |  |  |
| D3 | 189.7 (25.6) | 211.0 (42.9) | 199.2 (61.6) | 222.3 (49.9) |
| D7 | 189.7 (42.0) | 207.0 (75.9) | 184.7 (48.9) | 228.3 (59.8) |
| D14 | 207.0 (52.5) | 208.0 (55.7) | 202.7 (49.3) | 216.7 (54.7) |
| D28 | 174.5 (24.7) | 321.0 (7.1) | 199.6 (36.6) | 230.0 (61.2) |
| **Cholinesterase (U/L)** |  |  |  |  |
| D3 | 3737.0 (532.0) | 1506.0 (285.7) | 2989.2 (1321.6) | 4741.7 (3739.3) |
| D7 | 3613.3 (635.3) | 1590.7 (404.5) | 2979.7 (1371.4) | 4969.7 (4234.9) |
| D14 | 3563.0 (621.5) | 1514.3 (428.1) | 3166.5 (1375.6) | 4793.7 (3453.8) |
| D28 | 3750.3 (548.7) | 1681.5 (878.9) | 2842.2 (1377.6) | 5192.3 (4081.5) |
| **Total bile acid (μmol/L)** |  |  |  |  |
| D3 | 61.67 (43.98) | 201.67 (96.18) | 191.00 (239.45) | 111.33 (133.67) |
| D7 | 75.00 (50.09) | 294.67 (240.90) | 106.50 (91.35) | 180.67 (233.28) |
| D14 | 59.67 (31.90) | 167.67 (63.32) | 84.83 (61.31) | 82.33 (82.85) |
| D28 | 67.47 (27.25) | 147.00 (117.38) | 96.60 (63.82) | 160.33 (218.31) |
| **Prothrombin activity (%)** |  |  |  |  |
| D3 | 61.03 (17.50) | 47.40 (9.79) | 74.05 (24.06) | 64.93 (11.24) |
| D7 | 65.77 (33.74) | 44.17 (4.88) | 71.52 (31.73) | 68.57 (17.30) |
| D14 | 57.87 (17.19) | 41.40 (6.99) | 64.77 (24.61) | 65.73 (9.88) |
| D28 | 58.97 (15.35) | 44.37 (7.09) | 66.90 (26.74) | 68.50 (15.27) |

Clinical outcome indicators of Phase Ia within 28 days.

Supplementary Table 2. Clinical outcome indicators of Phase Ib within 28 days.

|  | **Cohort A (n=3)** | **Cohort B (n=6)** |
| --- | --- | --- |
| **MELD score** |  |  |
| D7 | 12.0 (4.4) | 11.2 (1.9) |
| D14 | 12.7 (3.8) | 10.8 (1.7) |
| D21 | 13.0 (4.6) | 10.2 (2.1) |
| D28 | 13.0 (4.6) | 10.7 (2.2) |
| **Child-Pugh score** |  |  |
| D7 | 8.3 (2.5) | 7.3 (1.4) |
| D14 | 7.7 (1.5) | 7.2 (1.2) |
| D21 | 7.0 (1.7) | 7.0 (0.9) |
| D28 | 7.3 (1.2) | 6.8 (1.0) |
| **EQ-5D-5L scale utility index** |  |  |
| D14 | 0.9493 (0.0067) | 0.8485 (0.0941) |
| D21 | 0.9453 (0.0577) | 0.8637 (0.0857) |
| D28 | 0.9643 (0.0312) | 0.8718 (0.1257) |
| **EQ-5D-5L scale VAS Score** |  |  |
| D14 | 71.3 (7.1) | 70.0 (13.8) |
| D21 | 71.7 (5.7) | 73.0 (8.9) |
| D28 | 75.0 (5.0) | 74.7 (12.0) |
| **Chronic Liver Disease Questionnaire Score** |  |  |
| D14 | 153.3 (19.9) | 141.5 (20.6) |
| D21 | 160.7 (15.8) | 142.0 (25.0) |
| D28 | 163.7 (23.1) | 150.0 (21.5) |
| **Albumin (g/L)** |  |  |
| D7 | 33.67 (5.13) | 31.00 (3.16) |
| D14 | 36.00 (3.46) | 31.00 (3.16) |
| D21 | 41.00 (3.00) | 32.33 (3.14) |
| D28 | 40.00 (3.61) | 34.67 (4.59) |
| **Prealbumin (g/L)** |  |  |
| D7 | 103.00 (59.51) | 68.50 (28.49) |
| D14 | 101.00 (49.49) | 71.00 (20.98) |
| D21 | 120.33 (54.45) | 77.00 (29.63) |
| D28 | 117.33 (62.74) | 87.67 (31.28) |
| **Total bilirubin (μmol/L)** |  |  |
| D7 | 33.10 (17.27) | 31.02 (30.08) |
| D14 | 38.27 (22.23) | 28.72 (25.09) |
| D21 | 45.00 (23.43) | 31.08 (31.03) |
| D28 | 44.50 (24.60) | 37.22 (36.78) |
| **Lactic dehydrogenase (U/L)** |  |  |
| D7 | 135.7 (10.2) | 181.8 (44.8) |
| D14 | 149.7 (18.1) | 176.0 (31.7) |
| D21 | 155.3 (7.8) | 197.7 (27.7) |
| D28 | 162.3 (21.5) | 194.7 (25.3) |
| **Cholinesterase (U/L)** |  |  |
| D7 | 3387.7 (1840.6) | 2627.2 (832.0) |
| D14 | 3467.7 (1853.6) | 2814.7 (692.7) |
| D21 | 3829.0 (1924.6) | 2825.5 (779.7) |
| D28 | 3854.0 (2029.0) | 3136.3 (947.6) |
| **Total bile acid (μmol/L)** |  |  |
| D7 | 131.67 (140.23) | 94.67 (80.33) |
| D14 | 173.00 (213.10) | 68.83 (42.54) |
| D21 | 201.33 (242.90) | 81.50 (27.82) |
| D28 | 245.67 (273.03) | 84.67 (40.22) |
| **Prothrombin activity (%)** |  |  |
| D7 | 58.33 (16.86) | 64.30 (8.62) |
| D14 | 59.77 (13.85) | 66.33 (12.04) |
| D21 | 62.90 (20.52) | 69.38 (7.37) |
| D28 | 61.73 (18.15) | 68.50 (7.31) |

Clinical outcome indicators of Phase Ib within 28 days.

Supplementary Table 3. Total markers for T cells, B cells, NK cells, monocytes and other myeloid immune cells.

| **cluster** | **gene** | **p_val** | **p_val_adj** | **avg_log2FC** | **pct.1** | **pct.2** |
| --- | --- | --- | --- | --- | --- | --- |
| T | IL7R | 0 | 0 | 4.873382595 | 0.715 | 0.031 |
| T | CD3D | 0 | 0 | 4.835160825 | 0.896 | 0.026 |
| T | CD3G | 0 | 0 | 4.376614002 | 0.75 | 0.019 |
| T | TCF7 | 0 | 0 | 3.546103423 | 0.581 | 0.059 |
| T | CD6 | 0 | 0 | 2.894186971 | 0.547 | 0.066 |
| T | CD3E | 0 | 0 | 2.888582371 | 0.971 | 0.151 |
| T | FLT3LG | 0 | 0 | 2.514941463 | 0.585 | 0.146 |
| T | LDHB | 0 | 0 | 2.316276787 | 0.855 | 0.453 |
| T | TC2N | 0 | 0 | 2.199079336 | 0.52 | 0.092 |
| T | SPOCK2 | 0 | 0 | 2.177689086 | 0.647 | 0.143 |
| T | IL32 | 0 | 0 | 2.168089985 | 0.964 | 0.215 |
| T | LAT | 0 | 0 | 2.072297943 | 0.665 | 0.161 |
| T | LIME1 | 0 | 0 | 2.018821751 | 0.658 | 0.169 |
| T | PIK3IP1 | 0 | 0 | 2.015738926 | 0.512 | 0.153 |
| T | NOSIP | 0 | 0 | 1.916964597 | 0.627 | 0.333 |
| T | CD2 | 0 | 0 | 1.909090415 | 0.645 | 0.148 |
| T | PCED1B-AS1 | 0 | 0 | 1.81125239 | 0.511 | 0.212 |
| T | LEPROTL1 | 0 | 0 | 1.791059721 | 0.645 | 0.292 |
| T | LCK | 0 | 0 | 1.676074986 | 0.787 | 0.229 |
| T | DNAJB1 | 0 | 0 | 1.664650681 | 0.548 | 0.238 |
| T | C12orf57 | 0 | 0 | 1.65140941 | 0.833 | 0.41 |
| T | LINC00861 | 0 | 0 | 1.637803318 | 0.592 | 0.16 |
| T | CLEC2D | 0 | 0 | 1.300047365 | 0.531 | 0.215 |
| T | LTB | 0 | 0 | 1.29392132 | 0.732 | 0.391 |
| T | GIMAP7 | 0 | 0 | 1.290677074 | 0.793 | 0.47 |
| T | ETS1 | 0 | 0 | 1.274362449 | 0.671 | 0.303 |
| T | TRBC2 | 0 | 0 | 1.195590269 | 0.62 | 0.287 |
| T | RHOH | 0 | 0 | 1.124105669 | 0.557 | 0.23 |
| T | PEBP1 | 0 | 0 | 1.037887662 | 0.584 | 0.329 |
| T | GYPC | 0 | 0 | 1.005576091 | 0.643 | 0.39 |
| B | CD79A | 0 | 0 | 7.310104692 | 0.99 | 0.018 |
| B | MS4A1 | 0 | 0 | 6.995221558 | 0.962 | 0.012 |
| B | TCL1A | 0 | 0 | 6.396202619 | 0.676 | 0.003 |
| B | IGHM | 0 | 0 | 6.291079968 | 0.809 | 0.008 |
| B | BANK1 | 0 | 0 | 6.073637161 | 0.88 | 0.011 |
| B | LINC00926 | 0 | 0 | 5.784303779 | 0.811 | 0.003 |
| B | FCER2 | 0 | 0 | 5.425462407 | 0.683 | 0.007 |
| B | IGKC | 0 | 0 | 5.367080036 | 0.5 | 0.017 |
| B | FCRL1 | 0 | 0 | 5.326693186 | 0.675 | 0.005 |
| B | IGHD | 0 | 0 | 5.077261789 | 0.59 | 0.002 |
| B | RALGPS2 | 0 | 0 | 4.976354428 | 0.787 | 0.035 |
| B | NIBAN3 | 0 | 0 | 4.957043808 | 0.518 | 0.002 |
| B | AFF3 | 0 | 0 | 4.914262777 | 0.722 | 0.035 |
| B | VPREB3 | 0 | 0 | 4.808905049 | 0.552 | 0 |
| B | CD79B | 0 | 0 | 4.769827089 | 0.891 | 0.09 |
| B | FCRLA | 0 | 0 | 4.628177247 | 0.56 | 0.001 |
| B | CD22 | 0 | 0 | 4.609458507 | 0.584 | 0.005 |
| B | CD19 | 0 | 0 | 4.480553426 | 0.557 | 0.001 |
| B | SPIB | 0 | 0 | 4.285281275 | 0.505 | 0.007 |
| B | BLK | 0 | 0 | 4.266889154 | 0.531 | 0.003 |
| B | BLNK | 0 | 0 | 4.132512308 | 0.534 | 0.007 |
| B | ADAM28 | 0 | 0 | 4.059314225 | 0.506 | 0.018 |
| B | PLPP5 | 0 | 0 | 3.929672273 | 0.614 | 0.069 |
| B | GNG7 | 0 | 0 | 3.871008824 | 0.53 | 0.023 |
| B | RUBCNL | 0 | 0 | 3.769762159 | 0.509 | 0.022 |
| B | HVCN1 | 0 | 0 | 3.701518359 | 0.788 | 0.145 |
| B | CD40 | 0 | 0 | 3.642638455 | 0.542 | 0.043 |
| B | SWAP70 | 0 | 0 | 3.619645993 | 0.67 | 0.08 |
| B | EAF2 | 0 | 0 | 3.474629107 | 0.549 | 0.062 |
| B | TCF4 | 0 | 0 | 3.427946336 | 0.521 | 0.042 |
| NK | SH2D1B | 0 | 0 | 4.283872669 | 0.54 | 0.016 |
| NK | KLRF1 | 0 | 0 | 3.989996957 | 0.766 | 0.052 |
| NK | SPON2 | 0 | 0 | 3.842032533 | 0.739 | 0.117 |
| NK | CLIC3 | 0 | 0 | 3.744345405 | 0.697 | 0.087 |
| NK | GNLY | 0 | 0 | 3.623359677 | 0.941 | 0.182 |
| NK | GZMB | 0 | 0 | 3.432874232 | 0.927 | 0.162 |
| NK | TRDC | 0 | 0 | 3.279037583 | 0.558 | 0.041 |
| NK | PRF1 | 0 | 0 | 3.276881589 | 0.966 | 0.233 |
| NK | HOPX | 0 | 0 | 3.058146017 | 0.844 | 0.164 |
| NK | IL2RB | 0 | 0 | 3.019678652 | 0.669 | 0.135 |
| NK | KLRD1 | 0 | 0 | 2.972177229 | 0.91 | 0.158 |
| NK | FGFBP2 | 0 | 0 | 2.94966215 | 0.801 | 0.158 |
| NK | CTSW | 0 | 0 | 2.92254977 | 0.979 | 0.287 |
| NK | TTC38 | 0 | 0 | 2.828979087 | 0.559 | 0.094 |
| NK | S1PR5 | 0 | 0 | 2.78574181 | 0.701 | 0.124 |
| NK | NKG7 | 0 | 0 | 2.784369113 | 0.994 | 0.306 |
| NK | PRSS23 | 0 | 0 | 2.778879473 | 0.536 | 0.083 |
| NK | CST7 | 0 | 0 | 2.643847835 | 0.982 | 0.279 |
| NK | ADGRG1 | 0 | 0 | 2.637020587 | 0.622 | 0.108 |
| NK | FCGR3A | 0 | 0 | 2.588067755 | 0.838 | 0.179 |
| NK | GZMA | 0 | 0 | 2.552715159 | 0.949 | 0.237 |
| NK | CD247 | 0 | 0 | 2.525828917 | 0.938 | 0.387 |
| NK | TBX21 | 0 | 0 | 2.514104127 | 0.568 | 0.111 |
| NK | CCL4 | 0 | 0 | 2.506304116 | 0.664 | 0.155 |
| NK | MATK | 0 | 0 | 2.465293024 | 0.636 | 0.138 |
| NK | NCR3 | 0 | 0 | 2.368458228 | 0.509 | 0.112 |
| NK | CMC1 | 0 | 0 | 2.326964339 | 0.616 | 0.22 |
| NK | FCRL6 | 0 | 0 | 2.309415252 | 0.534 | 0.106 |
| NK | TRBC1 | 0 | 0 | 2.283516018 | 0.748 | 0.265 |
| NK | CD7 | 0 | 0 | 2.174989063 | 0.884 | 0.412 |
| Monocyte | FCN1 | 0 | 0 | 6.166106903 | 0.981 | 0.029 |
| Monocyte | VCAN | 0 | 0 | 5.956489348 | 0.865 | 0.026 |
| Monocyte | LYZ | 0 | 0 | 5.764075218 | 0.992 | 0.076 |
| Monocyte | CD14 | 0 | 0 | 5.375379733 | 0.824 | 0.014 |
| Monocyte | S100A9 | 0 | 0 | 5.324175784 | 0.988 | 0.1 |
| Monocyte | IFI30 | 0 | 0 | 5.052653966 | 0.868 | 0.107 |
| Monocyte | S100A12 | 0 | 0 | 4.954070885 | 0.78 | 0.019 |
| Monocyte | CD68 | 0 | 0 | 4.948411933 | 0.896 | 0.033 |
| Monocyte | SERPINA1 | 0 | 0 | 4.914487254 | 0.949 | 0.032 |
| Monocyte | CFD | 0 | 0 | 4.895639181 | 0.838 | 0.026 |
| Monocyte | S100A8 | 0 | 0 | 4.762036606 | 0.952 | 0.072 |
| Monocyte | CSTA | 0 | 0 | 4.759013687 | 0.846 | 0.017 |
| Monocyte | TMEM176B | 0 | 0 | 4.755009925 | 0.56 | 0.005 |
| Monocyte | CD36 | 0 | 0 | 4.754592125 | 0.722 | 0.015 |
| Monocyte | AIF1 | 0 | 0 | 4.697395166 | 0.991 | 0.122 |
| Monocyte | LGALS2 | 0 | 0 | 4.612563935 | 0.635 | 0.016 |
| Monocyte | LRP1 | 0 | 0 | 4.594572462 | 0.749 | 0.014 |
| Monocyte | MS4A6A | 0 | 0 | 4.590275212 | 0.833 | 0.028 |
| Monocyte | CST3 | 0 | 0 | 4.575253069 | 0.997 | 0.082 |
| Monocyte | MAFB | 0 | 0 | 4.52577758 | 0.606 | 0.008 |
| Monocyte | TYMP | 0 | 0 | 4.510509723 | 0.963 | 0.129 |
| Monocyte | TNFAIP2 | 0 | 0 | 4.490303086 | 0.858 | 0.023 |
| Monocyte | DUSP6 | 0 | 0 | 4.45638638 | 0.744 | 0.026 |
| Monocyte | LILRB2 | 0 | 0 | 4.450544898 | 0.791 | 0.022 |
| Monocyte | LILRA5 | 0 | 0 | 4.446342972 | 0.721 | 0.011 |
| Monocyte | MS4A7 | 0 | 0 | 4.383999055 | 0.604 | 0.023 |
| Monocyte | SLC7A7 | 0 | 0 | 4.244945343 | 0.72 | 0.018 |
| Monocyte | NCF2 | 0 | 0 | 4.218392412 | 0.886 | 0.035 |
| Monocyte | SLC11A1 | 0 | 0 | 4.150550904 | 0.754 | 0.024 |
| Monocyte | CD300E | 0 | 0 | 4.137869402 | 0.595 | 0.008 |
| DC | FCER1A | 0 | 0 | 5.620880596 | 0.661 | 0.002 |
| DC | CLEC10A | 0 | 0 | 5.097519947 | 0.744 | 0.018 |
| DC | CD1C | 0 | 0 | 4.367547525 | 0.654 | 0.027 |
| DC | HLA-DQA1 | 0 | 0 | 3.270094933 | 0.965 | 0.283 |
| DC | CST3 | 0 | 0 | 3.22070807 | 1 | 0.289 |
| DC | FABP5 | 0 | 0 | 3.174213846 | 0.595 | 0.088 |
| DC | HLA-DQA2 | 0 | 0 | 3.149508751 | 0.709 | 0.116 |
| DC | HLA-DQB1 | 0 | 0 | 3.119467807 | 0.991 | 0.339 |
| DC | HLA-DRA | 0 | 0 | 2.947088124 | 1 | 0.473 |
| DC | HLA-DRB5 | 0 | 0 | 2.932915415 | 0.991 | 0.497 |
| DC | CPVL | 0 | 0 | 2.907775044 | 0.941 | 0.177 |
| DC | HLA-DPA1 | 0 | 0 | 2.847607812 | 0.999 | 0.627 |
| DC | HLA-DRB1 | 0 | 0 | 2.817166721 | 0.999 | 0.538 |
| DC | HLA-DPB1 | 0 | 0 | 2.733209108 | 0.999 | 0.634 |
| DC | PLD4 | 0 | 0 | 2.62535657 | 0.688 | 0.06 |
| DC | GPAT3 | 0 | 0 | 2.56544725 | 0.644 | 0.052 |
| DC | GSN | 0 | 0 | 2.488376497 | 0.835 | 0.12 |
| DC | F13A1 | 0 | 0 | 2.418261486 | 0.545 | 0.067 |
| DC | RTN1 | 0 | 0 | 2.367612337 | 0.585 | 0.062 |
| DC | IL18 | 0 | 0 | 2.320093495 | 0.552 | 0.046 |
| DC | CCDC88A | 0 | 0 | 2.290054369 | 0.877 | 0.152 |
| DC | PID1 | 0 | 0 | 2.287985706 | 0.55 | 0.064 |
| DC | HLA-DMA | 0 | 0 | 2.28559813 | 0.982 | 0.346 |
| DC | LILRB4 | 0 | 0 | 2.253568913 | 0.675 | 0.091 |
| DC | CD86 | 0 | 0 | 2.237818599 | 0.806 | 0.128 |
| DC | LGALS2 | 0 | 0 | 2.214421548 | 0.868 | 0.153 |
| DC | CD33 | 0 | 0 | 2.195559613 | 0.766 | 0.113 |
| DC | CD1D | 0 | 0 | 2.157152931 | 0.654 | 0.088 |
| DC | IL13RA1 | 0 | 0 | 2.097551428 | 0.623 | 0.067 |
| DC | FCGR2B | 0 | 0 | 2.095009419 | 0.555 | 0.089 |
| pDC | LILRA4 | 0 | 0 | 6.862732491 | 0.937 | 0.005 |
| pDC | SERPINF1 | 0 | 0 | 6.190161848 | 0.947 | 0.013 |
| pDC | PLD4 | 0 | 0 | 6.002516772 | 0.965 | 0.065 |
| pDC | SCT | 0 | 0 | 5.627193206 | 0.797 | 0 |
| pDC | TPM2 | 0 | 0 | 5.578345497 | 0.806 | 0.007 |
| pDC | ITM2C | 0 | 0 | 5.573131872 | 0.952 | 0.08 |
| pDC | CLEC4C | 0 | 0 | 5.537414243 | 0.869 | 0.003 |
| pDC | IL3RA | 0 | 0 | 5.442021821 | 0.927 | 0.027 |
| pDC | LRRC26 | 0 | 0 | 5.37034043 | 0.794 | 0 |
| pDC | PTCRA | 0 | 0 | 5.131219969 | 0.782 | 0.001 |
| pDC | MAP1A | 0 | 0 | 4.956517027 | 0.792 | 0.003 |
| pDC | UGCG | 0 | 0 | 4.905660234 | 0.945 | 0.098 |
| pDC | PTPRS | 0 | 0 | 4.896756321 | 0.77 | 0.003 |
| pDC | CCDC50 | 0 | 0 | 4.881061229 | 0.956 | 0.116 |
| pDC | TCF4 | 0 | 0 | 4.83169438 | 0.961 | 0.097 |
| pDC | APP | 0 | 0 | 4.808865175 | 0.968 | 0.157 |
| pDC | DNASE1L3 | 0 | 0 | 4.799009046 | 0.778 | 0.006 |
| pDC | IRF8 | 0 | 0 | 4.759914458 | 0.969 | 0.169 |
| pDC | MZB1 | 0 | 0 | 4.704702026 | 0.913 | 0.036 |
| pDC | LINC00996 | 0 | 0 | 4.566615895 | 0.669 | 0.009 |
| pDC | SMPD3 | 0 | 0 | 4.545706793 | 0.768 | 0.011 |
| pDC | PPP1R14B | 0 | 0 | 4.509029598 | 0.883 | 0.177 |
| pDC | IRF7 | 0 | 0 | 4.508819467 | 0.977 | 0.294 |
| pDC | TNFRSF21 | 0 | 0 | 4.457745895 | 0.721 | 0.003 |
| pDC | DERL3 | 0 | 0 | 4.402349442 | 0.805 | 0.017 |
| pDC | ZFAT | 0 | 0 | 4.370991471 | 0.794 | 0.032 |
| pDC | LAMP5 | 0 | 0 | 4.296734655 | 0.629 | 0.005 |
| pDC | JCHAIN | 0 | 0 | 4.175793297 | 0.947 | 0.052 |
| pDC | GAS6 | 0 | 0 | 4.1477684 | 0.712 | 0.024 |
| pDC | LGMN | 0 | 0 | 3.97710865 | 0.732 | 0.022 |
| Neutrophil | FCGR3B | 0 | 0 | 8.179700311 | 0.965 | 0.018 |
| Neutrophil | G0S2 | 0 | 0 | 6.597485194 | 0.766 | 0.034 |
| Neutrophil | CMTM2 | 0 | 0 | 6.588182944 | 0.692 | 0.009 |
| Neutrophil | TNFRSF10C | 0 | 0 | 6.128581682 | 0.707 | 0.021 |
| Neutrophil | CXCR2 | 0 | 0 | 5.999391455 | 0.8 | 0.042 |
| Neutrophil | IFIT2 | 0 | 0 | 5.86782732 | 0.726 | 0.104 |
| Neutrophil | FFAR2 | 0 | 0 | 5.587099211 | 0.572 | 0.026 |
| Neutrophil | MMP25 | 0 | 0 | 5.462376469 | 0.639 | 0.031 |
| Neutrophil | IFIT3 | 0 | 0 | 5.452360859 | 0.732 | 0.112 |
| Neutrophil | SLC25A37 | 0 | 0 | 5.342760988 | 0.88 | 0.158 |
| Neutrophil | IFIT1 | 0 | 0 | 5.269965851 | 0.555 | 0.055 |
| Neutrophil | RSAD2 | 0 | 0 | 5.218354567 | 0.515 | 0.056 |
| Neutrophil | BASP1 | 0 | 0 | 5.171308783 | 0.776 | 0.103 |
| Neutrophil | NAMPT | 0 | 0 | 5.134791824 | 0.964 | 0.219 |
| Neutrophil | AQP9 | 0 | 0 | 4.923360784 | 0.589 | 0.049 |
| Neutrophil | CSF3R | 0 | 0 | 4.764736113 | 0.962 | 0.203 |
| Neutrophil | MXD1 | 0 | 0 | 4.599827366 | 0.738 | 0.138 |
| Neutrophil | MNDA | 0 | 0 | 4.483604259 | 0.986 | 0.247 |
| Neutrophil | SOD2 | 0 | 0 | 4.404405088 | 0.93 | 0.357 |
| Neutrophil | FCGR2A | 0 | 0 | 4.351558696 | 0.786 | 0.162 |
| Neutrophil | HERC5 | 0 | 0 | 4.313162605 | 0.517 | 0.117 |
| Neutrophil | CDA | 0 | 0 | 4.251220121 | 0.723 | 0.153 |
| Neutrophil | IFITM3 | 0 | 0 | 4.228007755 | 0.948 | 0.412 |
| Neutrophil | ISG15 | 0 | 0 | 4.221262155 | 0.706 | 0.376 |
| Neutrophil | FPR1 | 0 | 0 | 4.177142273 | 0.884 | 0.196 |
| Neutrophil | GCA | 0 | 0 | 4.157595702 | 0.834 | 0.219 |
| Neutrophil | ZDHHC18 | 0 | 0 | 4.109930869 | 0.504 | 0.093 |
| Neutrophil | MBOAT7 | 0 | 0 | 4.077815783 | 0.631 | 0.142 |
| Neutrophil | NIBAN1 | 0 | 0 | 4.054078699 | 0.598 | 0.138 |
| Neutrophil | HIST1H2AC | 0 | 0 | 4.027978729 | 0.507 | 0.119 |

Total markers for T cells, B cells, NK cells, monocytes and other myeloid immune cells.

Supplementary Table 4. Monocyte markers.

| **cluster** | **gene** | **p_val** | **p_val_adj** | **avg_log2FC** | **pct.1** | **pct.2** |
| --- | --- | --- | --- | --- | --- | --- |
| c35_Mono_VCAN | PADI4 | 0 | 0 | 2.238968727 | 0.447 | 0.14 |
| c35_Mono_VCAN | CYP1B1 | 0 | 0 | 1.841386313 | 0.464 | 0.205 |
| c35_Mono_VCAN | S100A12 | 0 | 0 | 1.74300866 | 0.941 | 0.702 |
| c35_Mono_VCAN | SLC2A3 | 0 | 0 | 1.395651758 | 0.541 | 0.32 |
| c35_Mono_VCAN | RBP7 | 0 | 0 | 1.295874091 | 0.619 | 0.458 |
| c35_Mono_VCAN | EMB | 0 | 0 | 1.249064451 | 0.433 | 0.275 |
| c35_Mono_VCAN | PLBD1 | 0 | 0 | 1.24368739 | 0.675 | 0.572 |
| c35_Mono_VCAN | DYSF | 0 | 0 | 1.206886114 | 0.481 | 0.318 |
| c35_Mono_VCAN | NFE2 | 0 | 0 | 1.116568483 | 0.501 | 0.355 |
| c35_Mono_VCAN | VCAN | 0 | 0 | 1.11368321 | 0.955 | 0.821 |
| c35_Mono_VCAN | ITGAM | 0 | 0 | 1.109015026 | 0.562 | 0.424 |
| c35_Mono_VCAN | HMGB2 | 0 | 0 | 1.050037006 | 0.538 | 0.437 |
| c35_Mono_VCAN | LINC00937 | 0 | 0 | 0.973327979 | 0.41 | 0.308 |
| c35_Mono_VCAN | SELL | 0 | 0 | 0.954123409 | 0.676 | 0.558 |
| c35_Mono_VCAN | MGST1 | 0 | 0 | 0.938608737 | 0.431 | 0.32 |
| c35_Mono_VCAN | ARGLU1 | 2.66E-05 | 0.802061844 | 0.280728639 | 0.51 | 0.626 |
| c35_Mono_VCAN | GRK2 | 9.78E-17 | 2.95E-12 | 0.274490579 | 0.662 | 0.771 |
| c35_Mono_VCAN | RCSD1 | 7.66E-21 | 2.31E-16 | 0.269891936 | 0.637 | 0.743 |
| c35_Mono_VCAN | SCAND1 | 2.26E-34 | 6.81E-30 | 0.263718361 | 0.663 | 0.774 |
| c35_Mono_VCAN | PLAUR | 6.35E-18 | 1.91E-13 | 0.261206684 | 0.485 | 0.595 |
| c35_Mono_VCAN | TCF25 | 5.87E-05 | 1 | 0.254387451 | 0.634 | 0.748 |
| c36_Mono_LGALS2 | LGALS2 | 0 | 0 | 0.987505266 | 0.911 | 0.551 |
| c36_Mono_LGALS2 | F13A1 | 0 | 0 | 0.966254753 | 0.437 | 0.21 |
| c36_Mono_LGALS2 | CD99 | 0 | 0 | 0.87054813 | 0.859 | 0.574 |
| c36_Mono_LGALS2 | CRIP1 | 0 | 0 | 0.803171066 | 0.811 | 0.645 |
| c36_Mono_LGALS2 | NRG1 | 0 | 0 | 0.689626912 | 0.426 | 0.231 |
| c36_Mono_LGALS2 | TAGLN2 | 0 | 0 | 0.64208734 | 0.937 | 0.806 |
| c36_Mono_LGALS2 | LDHB | 0 | 0 | 0.602327768 | 0.55 | 0.362 |
| c36_Mono_LGALS2 | APEX1 | 0 | 0 | 0.488173197 | 0.483 | 0.336 |
| c36_Mono_LGALS2 | PPA1 | 0 | 0 | 0.485065821 | 0.47 | 0.325 |
| c36_Mono_LGALS2 | PLD3 | 0 | 0 | 0.473788488 | 0.583 | 0.413 |
| c36_Mono_LGALS2 | CRTAP | 0 | 0 | 0.458296106 | 0.835 | 0.651 |
| c36_Mono_LGALS2 | LY86 | 0 | 0 | 0.456888759 | 0.736 | 0.547 |
| c36_Mono_LGALS2 | TMEM176A | 2.28E-251 | 6.88E-247 | 0.450580206 | 0.488 | 0.378 |
| c36_Mono_LGALS2 | ANXA1 | 0 | 0 | 0.419392532 | 0.918 | 0.791 |
| c36_Mono_LGALS2 | MGST1 | 0 | 0 | 0.416890859 | 0.501 | 0.312 |
| c36_Mono_LGALS2 | CNIH1 | 0 | 0 | 0.411418225 | 0.463 | 0.33 |
| c36_Mono_LGALS2 | RNASE6 | 0 | 0 | 0.406824797 | 0.443 | 0.285 |
| c36_Mono_LGALS2 | PCBP2 | 0 | 0 | 0.394963571 | 0.837 | 0.722 |
| c36_Mono_LGALS2 | HINT1 | 0 | 0 | 0.385207192 | 0.881 | 0.748 |
| c36_Mono_LGALS2 | NPM1 | 0 | 0 | 0.383717312 | 0.835 | 0.683 |
| c36_Mono_LGALS2 | OSTC | 4.31E-263 | 1.30E-258 | 0.368272671 | 0.467 | 0.35 |
| c36_Mono_LGALS2 | TMEM173 | 6.81E-266 | 2.05E-261 | 0.36777848 | 0.41 | 0.288 |
| c36_Mono_LGALS2 | TGFBI | 0 | 0 | 0.350598701 | 0.8 | 0.659 |
| c36_Mono_LGALS2 | ATP6V1F | 0 | 0 | 0.344702329 | 0.878 | 0.745 |
| c36_Mono_LGALS2 | EIF3L | 0 | 0 | 0.343314894 | 0.82 | 0.687 |
| c36_Mono_LGALS2 | EIF3E | 0 | 0 | 0.338184721 | 0.841 | 0.711 |
| c36_Mono_LGALS2 | ALDH2 | 0 | 0 | 0.336080315 | 0.744 | 0.575 |
| c36_Mono_LGALS2 | AP1S2 | 0 | 0 | 0.33398966 | 0.927 | 0.822 |
| c36_Mono_LGALS2 | MYADM | 0 | 0 | 0.321161911 | 0.719 | 0.582 |
| c36_Mono_LGALS2 | CAPG | 0 | 0 | 0.31714458 | 0.857 | 0.68 |
| c37_Mono_HLA-DPB1 | HLA-DQA1 | 0 | 0 | 1.706779529 | 0.716 | 0.311 |
| c37_Mono_HLA-DPB1 | HLA-DPB1 | 0 | 0 | 1.607936667 | 0.993 | 0.745 |
| c37_Mono_HLA-DPB1 | HLA-DPA1 | 0 | 0 | 1.332665191 | 0.995 | 0.809 |
| c37_Mono_HLA-DPB1 | PID1 | 0 | 0 | 1.296707112 | 0.529 | 0.209 |
| c37_Mono_HLA-DPB1 | SASH1 | 0 | 0 | 1.275522809 | 0.4 | 0.148 |
| c37_Mono_HLA-DPB1 | APOBEC3A | 0 | 0 | 1.237060873 | 0.71 | 0.456 |
| c37_Mono_HLA-DPB1 | HLA-DQB1 | 0 | 0 | 1.193544144 | 0.89 | 0.554 |
| c37_Mono_HLA-DPB1 | HLA-DRB1 | 0 | 0 | 1.140924738 | 0.996 | 0.895 |
| c37_Mono_HLA-DPB1 | ATF5 | 0 | 0 | 1.109499821 | 0.483 | 0.217 |
| c37_Mono_HLA-DPB1 | HLA-DMA | 0 | 0 | 1.10252344 | 0.936 | 0.634 |
| c37_Mono_HLA-DPB1 | PEA15 | 0 | 0 | 0.994933476 | 0.564 | 0.271 |
| c37_Mono_HLA-DPB1 | MARCKS | 0 | 0 | 0.968464305 | 0.87 | 0.578 |
| c37_Mono_HLA-DPB1 | LIPA | 0 | 0 | 0.962481895 | 0.566 | 0.308 |
| c37_Mono_HLA-DPB1 | GBP1 | 0 | 0 | 0.957446509 | 0.609 | 0.341 |
| c37_Mono_HLA-DPB1 | ABI3 | 0 | 0 | 0.955765187 | 0.712 | 0.34 |
| c37_Mono_HLA-DPB1 | HLA-DRB5 | 0 | 0 | 0.918440638 | 0.925 | 0.767 |
| c37_Mono_HLA-DPB1 | LGALS2 | 0 | 0 | 0.888838368 | 0.898 | 0.579 |
| c37_Mono_HLA-DPB1 | HLA-DMB | 0 | 0 | 0.879315194 | 0.812 | 0.502 |
| c37_Mono_HLA-DPB1 | EGR1 | 0 | 0 | 0.87400975 | 0.443 | 0.225 |
| c37_Mono_HLA-DPB1 | PSME2 | 0 | 0 | 0.851300462 | 0.976 | 0.807 |
| c37_Mono_HLA-DPB1 | EPB41L3 | 0 | 0 | 0.840824457 | 0.515 | 0.276 |
| c37_Mono_HLA-DPB1 | CALHM6 | 0 | 0 | 0.813419326 | 0.834 | 0.543 |
| c37_Mono_HLA-DPB1 | PLAC8 | 0 | 0 | 0.809524217 | 0.771 | 0.542 |
| c37_Mono_HLA-DPB1 | KLF2 | 0 | 0 | 0.781065188 | 0.861 | 0.651 |
| c37_Mono_HLA-DPB1 | GBP4 | 0 | 0 | 0.77841891 | 0.521 | 0.282 |
| c37_Mono_HLA-DPB1 | CAMK1 | 0 | 0 | 0.777769566 | 0.606 | 0.338 |
| c37_Mono_HLA-DPB1 | FUOM | 0 | 0 | 0.752555159 | 0.498 | 0.261 |
| c37_Mono_HLA-DPB1 | LAP3 | 0 | 0 | 0.708194438 | 0.749 | 0.504 |
| c37_Mono_HLA-DPB1 | NENF | 0 | 0 | 0.705649729 | 0.421 | 0.224 |
| c37_Mono_HLA-DPB1 | HSPA8 | 0 | 0 | 0.704876703 | 0.91 | 0.69 |
| c38_Mono_MX1 | MX1 | 0 | 0 | 1.284885864 | 0.655 | 0.435 |
| c38_Mono_MX1 | IFI44L | 0 | 0 | 1.141409737 | 0.532 | 0.317 |
| c38_Mono_MX1 | OAS3 | 0 | 0 | 1.124289399 | 0.45 | 0.265 |
| c38_Mono_MX1 | PARP14 | 0 | 0 | 1.10606249 | 0.695 | 0.477 |
| c38_Mono_MX1 | ISG15 | 0 | 0 | 1.083070596 | 0.699 | 0.541 |
| c38_Mono_MX1 | IFIT3 | 0 | 0 | 1.069899135 | 0.401 | 0.253 |
| c38_Mono_MX1 | IFI44 | 0 | 0 | 1.007912448 | 0.466 | 0.285 |
| c38_Mono_MX1 | MX2 | 0 | 0 | 0.989167296 | 0.601 | 0.415 |
| c38_Mono_MX1 | RNF213 | 0 | 0 | 0.900671615 | 0.919 | 0.798 |
| c38_Mono_MX1 | XAF1 | 0 | 0 | 0.886752673 | 0.7 | 0.517 |
| c38_Mono_MX1 | AC020916.1 | 0 | 0 | 0.881039668 | 0.605 | 0.405 |
| c38_Mono_MX1 | CYP27A1 | 0 | 0 | 0.878908333 | 0.421 | 0.234 |
| c38_Mono_MX1 | OAS2 | 0 | 0 | 0.867903826 | 0.481 | 0.334 |
| c38_Mono_MX1 | SAMD9L | 0 | 0 | 0.853807309 | 0.41 | 0.277 |
| c38_Mono_MX1 | EIF2AK2 | 0 | 0 | 0.84422111 | 0.427 | 0.289 |
| c38_Mono_MX1 | TNFAIP3 | 0 | 0 | 0.819812685 | 0.454 | 0.303 |
| c38_Mono_MX1 | EPSTI1 | 0 | 0 | 0.819223937 | 0.606 | 0.441 |
| c38_Mono_MX1 | ACSL1 | 0 | 0 | 0.816329285 | 0.521 | 0.367 |
| c38_Mono_MX1 | ARHGAP26 | 0 | 0 | 0.79189126 | 0.655 | 0.504 |
| c38_Mono_MX1 | STAT1 | 0 | 0 | 0.766656995 | 0.797 | 0.64 |
| c38_Mono_MX1 | TRIM22 | 0 | 0 | 0.763730935 | 0.655 | 0.496 |
| c38_Mono_MX1 | STAT2 | 0 | 0 | 0.762253558 | 0.676 | 0.528 |
| c38_Mono_MX1 | IFI6 | 0 | 0 | 0.758530118 | 0.76 | 0.617 |
| c38_Mono_MX1 | FOSB | 0 | 0 | 0.752730276 | 0.451 | 0.295 |
| c38_Mono_MX1 | SMCHD1 | 0 | 0 | 0.75196837 | 0.771 | 0.625 |
| c38_Mono_MX1 | GBP1 | 1.12E-258 | 3.38E-254 | 0.750324797 | 0.482 | 0.371 |
| c38_Mono_MX1 | ALDH1A1 | 0 | 0 | 0.746505772 | 0.404 | 0.249 |
| c38_Mono_MX1 | PELI1 | 0 | 0 | 0.727028885 | 0.435 | 0.31 |
| c38_Mono_MX1 | LAP3 | 0 | 0 | 0.723906333 | 0.661 | 0.526 |
| c38_Mono_MX1 | TNFSF10 | 0 | 0 | 0.718121266 | 0.796 | 0.659 |
| c39_Mono_FCGR3A | CDKN1C | 0 | 0 | 5.457253979 | 0.789 | 0.033 |
| c39_Mono_FCGR3A | FCGR3A | 0 | 0 | 4.983485229 | 0.987 | 0.142 |
| c39_Mono_FCGR3A | HES4 | 0 | 0 | 3.459406561 | 0.705 | 0.058 |
| c39_Mono_FCGR3A | RHOC | 0 | 0 | 3.440789603 | 0.886 | 0.177 |
| c39_Mono_FCGR3A | MTSS1 | 0 | 0 | 2.836381129 | 0.897 | 0.213 |
| c39_Mono_FCGR3A | CD79B | 0 | 0 | 2.801625797 | 0.539 | 0.047 |
| c39_Mono_FCGR3A | TCF7L2 | 0 | 0 | 2.466863626 | 0.868 | 0.259 |
| c39_Mono_FCGR3A | ADA | 0 | 0 | 2.463362732 | 0.485 | 0.05 |
| c39_Mono_FCGR3A | NEURL1 | 0 | 0 | 2.423751946 | 0.48 | 0.055 |
| c39_Mono_FCGR3A | MS4A4A | 0 | 0 | 2.377431538 | 0.473 | 0.071 |
| c39_Mono_FCGR3A | CTSL | 0 | 0 | 2.362016414 | 0.649 | 0.109 |
| c39_Mono_FCGR3A | PLD4 | 0 | 0 | 2.327324397 | 0.406 | 0.049 |
| c39_Mono_FCGR3A | SIGLEC10 | 0 | 0 | 2.319615262 | 0.793 | 0.206 |
| c39_Mono_FCGR3A | IFITM1 | 0 | 0 | 2.283118542 | 0.824 | 0.284 |
| c39_Mono_FCGR3A | EVL | 0 | 0 | 2.226875242 | 0.779 | 0.194 |
| c39_Mono_FCGR3A | NR4A1 | 0 | 0 | 2.201066955 | 0.662 | 0.155 |
| c39_Mono_FCGR3A | MS4A7 | 0 | 0 | 2.17325528 | 0.957 | 0.553 |
| c39_Mono_FCGR3A | SPN | 0 | 0 | 2.171523135 | 0.839 | 0.277 |
| c39_Mono_FCGR3A | BATF3 | 0 | 0 | 2.150367299 | 0.485 | 0.071 |
| c39_Mono_FCGR3A | LTB | 0 | 0 | 2.069800899 | 0.667 | 0.166 |
| c39_Mono_FCGR3A | INSIG1 | 0 | 0 | 2.066000257 | 0.615 | 0.14 |
| c39_Mono_FCGR3A | SMIM25 | 0 | 0 | 2.036055075 | 0.949 | 0.489 |
| c39_Mono_FCGR3A | CSF1R | 0 | 0 | 1.99460189 | 0.931 | 0.453 |
| c39_Mono_FCGR3A | ABI3 | 0 | 0 | 1.971456279 | 0.885 | 0.334 |
| c39_Mono_FCGR3A | CX3CR1 | 0 | 0 | 1.964177818 | 0.746 | 0.296 |
| c39_Mono_FCGR3A | CTSC | 0 | 0 | 1.953204813 | 0.917 | 0.5 |
| c39_Mono_FCGR3A | HMOX1 | 0 | 0 | 1.938309248 | 0.9 | 0.468 |
| c39_Mono_FCGR3A | CYFIP2 | 0 | 0 | 1.90294592 | 0.563 | 0.116 |
| c39_Mono_FCGR3A | PHTF2 | 0 | 0 | 1.834433064 | 0.516 | 0.117 |
| c39_Mono_FCGR3A | FAM110A | 0 | 0 | 1.825559021 | 0.82 | 0.344 |

Monocyte markers.

Supplementary Table 5. T cell markers.

| **cluster** | **gene** | **p_val** | **p_val_adj** | **avg_log2FC** | **pct.1** | **pct.2** |
| --- | --- | --- | --- | --- | --- | --- |
| c1_CD4Tn_CCR7 | CCR7 | 0 | 0 | 1.902624506 | 0.804 | 0.305 |
| c1_CD4Tn_CCR7 | LEF1 | 0 | 0 | 1.530444346 | 0.813 | 0.371 |
| c1_CD4Tn_CCR7 | IL6ST | 0 | 0 | 1.526835149 | 0.524 | 0.234 |
| c1_CD4Tn_CCR7 | MAL | 0 | 0 | 1.362802328 | 0.667 | 0.288 |
| c1_CD4Tn_CCR7 | TRABD2A | 0 | 0 | 1.301436348 | 0.423 | 0.194 |
| c1_CD4Tn_CCR7 | BACH2 | 0 | 0 | 1.246847814 | 0.418 | 0.189 |
| c1_CD4Tn_CCR7 | TCF7 | 0 | 0 | 1.20061874 | 0.856 | 0.509 |
| c1_CD4Tn_CCR7 | SELL | 0 | 0 | 1.126602592 | 0.856 | 0.471 |
| c1_CD4Tn_CCR7 | ABLIM1 | 0 | 0 | 1.090388958 | 0.571 | 0.336 |
| c1_CD4Tn_CCR7 | PIK3IP1 | 0 | 0 | 1.087271212 | 0.719 | 0.458 |
| c1_CD4Tn_CCR7 | SATB1 | 0 | 0 | 1.076135902 | 0.489 | 0.267 |
| c1_CD4Tn_CCR7 | MAML2 | 0 | 0 | 1.07544775 | 0.442 | 0.256 |
| c1_CD4Tn_CCR7 | CAMK4 | 0 | 0 | 1.066505985 | 0.703 | 0.428 |
| c1_CD4Tn_CCR7 | RCAN3 | 0 | 0 | 1.04603057 | 0.579 | 0.325 |
| c1_CD4Tn_CCR7 | LDLRAP1 | 0 | 0 | 0.989217247 | 0.456 | 0.265 |
| c1_CD4Tn_CCR7 | OXNAD1 | 0 | 0 | 0.947427254 | 0.483 | 0.289 |
| c1_CD4Tn_CCR7 | FOXP1 | 0 | 0 | 0.904588433 | 0.774 | 0.543 |
| c1_CD4Tn_CCR7 | NOSIP | 0 | 0 | 0.881557622 | 0.807 | 0.58 |
| c1_CD4Tn_CCR7 | TESPA1 | 0 | 0 | 0.825535521 | 0.457 | 0.309 |
| c1_CD4Tn_CCR7 | RGS10 | 0 | 0 | 0.824409947 | 0.708 | 0.504 |
| c1_CD4Tn_CCR7 | CD55 | 0 | 0 | 0.769490476 | 0.468 | 0.32 |
| c1_CD4Tn_CCR7 | NDFIP1 | 0 | 0 | 0.766370656 | 0.705 | 0.55 |
| c1_CD4Tn_CCR7 | TRAT1 | 0 | 0 | 0.727524218 | 0.449 | 0.294 |
| c1_CD4Tn_CCR7 | DGKA | 0 | 0 | 0.724253293 | 0.475 | 0.325 |
| c1_CD4Tn_CCR7 | LDHB | 0 | 0 | 0.717698123 | 0.966 | 0.825 |
| c1_CD4Tn_CCR7 | TMEM123 | 0 | 0 | 0.709730812 | 0.726 | 0.531 |
| c1_CD4Tn_CCR7 | LTB | 0 | 0 | 0.706095099 | 0.978 | 0.667 |
| c1_CD4Tn_CCR7 | AC243960.1 | 0 | 0 | 0.704007676 | 0.496 | 0.365 |
| c1_CD4Tn_CCR7 | INPP4B | 0 | 0 | 0.702214871 | 0.497 | 0.348 |
| c1_CD4Tn_CCR7 | EIF3E | 0 | 0 | 0.684169409 | 0.865 | 0.728 |
| c2_CD4Tcm_GPR183 | AQP3 | 0 | 0 | 1.571983022 | 0.692 | 0.287 |
| c2_CD4Tcm_GPR183 | CD82 | 0 | 0 | 1.420704783 | 0.481 | 0.159 |
| c2_CD4Tcm_GPR183 | CD40LG | 0 | 0 | 1.381662376 | 0.401 | 0.123 |
| c2_CD4Tcm_GPR183 | LTB | 0 | 0 | 1.354284432 | 0.991 | 0.684 |
| c2_CD4Tcm_GPR183 | LIMS1 | 0 | 0 | 1.315931445 | 0.46 | 0.193 |
| c2_CD4Tcm_GPR183 | TRADD | 0 | 0 | 1.31450414 | 0.609 | 0.269 |
| c2_CD4Tcm_GPR183 | GPR183 | 0 | 0 | 1.255708161 | 0.529 | 0.208 |
| c2_CD4Tcm_GPR183 | PLP2 | 0 | 0 | 1.159868881 | 0.758 | 0.408 |
| c2_CD4Tcm_GPR183 | CD4 | 0 | 0 | 1.109284188 | 0.454 | 0.173 |
| c2_CD4Tcm_GPR183 | MAL | 0 | 0 | 1.079770775 | 0.671 | 0.31 |
| c2_CD4Tcm_GPR183 | TIMP1 | 0 | 0 | 1.079525548 | 0.509 | 0.245 |
| c2_CD4Tcm_GPR183 | INPP4B | 0 | 0 | 1.02613795 | 0.635 | 0.331 |
| c2_CD4Tcm_GPR183 | IL7R | 0 | 0 | 1.017128661 | 0.957 | 0.67 |
| c2_CD4Tcm_GPR183 | TRAT1 | 0 | 0 | 0.954020918 | 0.574 | 0.28 |
| c2_CD4Tcm_GPR183 | CORO1B | 0 | 0 | 0.938003836 | 0.543 | 0.301 |
| c2_CD4Tcm_GPR183 | RNASET2 | 0 | 0 | 0.931150708 | 0.781 | 0.482 |
| c2_CD4Tcm_GPR183 | CD28 | 0 | 0 | 0.908328363 | 0.431 | 0.197 |
| c2_CD4Tcm_GPR183 | S100A11 | 0 | 0 | 0.892546336 | 0.86 | 0.575 |
| c2_CD4Tcm_GPR183 | TTC39C | 0 | 0 | 0.848847616 | 0.588 | 0.321 |
| c2_CD4Tcm_GPR183 | COTL1 | 0 | 0 | 0.835529368 | 0.795 | 0.452 |
| c2_CD4Tcm_GPR183 | ITGB1 | 0 | 0 | 0.822617848 | 0.699 | 0.393 |
| c2_CD4Tcm_GPR183 | BIRC3 | 0 | 0 | 0.770401367 | 0.442 | 0.222 |
| c2_CD4Tcm_GPR183 | PLSCR3 | 0 | 0 | 0.755641706 | 0.526 | 0.312 |
| c2_CD4Tcm_GPR183 | GSTK1 | 0 | 0 | 0.746356813 | 0.933 | 0.757 |
| c2_CD4Tcm_GPR183 | GATA3 | 0 | 0 | 0.746044808 | 0.451 | 0.275 |
| c2_CD4Tcm_GPR183 | C1orf162 | 0 | 0 | 0.724771955 | 0.411 | 0.24 |
| c2_CD4Tcm_GPR183 | ARHGAP15 | 0 | 0 | 0.690829345 | 0.777 | 0.569 |
| c2_CD4Tcm_GPR183 | CRIP1 | 0 | 0 | 0.679870594 | 0.859 | 0.656 |
| c2_CD4Tcm_GPR183 | TESPA1 | 0 | 0 | 0.67644322 | 0.528 | 0.304 |
| c2_CD4Tcm_GPR183 | RCAN3 | 0 | 0 | 0.658971478 | 0.602 | 0.336 |
| c3_CD4Tem_GZMK | GZMK | 0 | 0 | 2.004623723 | 0.657 | 0.152 |
| c3_CD4Tem_GZMK | CXCR3 | 0 | 0 | 1.599358589 | 0.486 | 0.173 |
| c3_CD4Tem_GZMK | IL7R | 0 | 0 | 1.322873213 | 0.971 | 0.701 |
| c3_CD4Tem_GZMK | CD82 | 0 | 0 | 1.083118006 | 0.408 | 0.199 |
| c3_CD4Tem_GZMK | TIMP1 | 0 | 0 | 1.081478284 | 0.502 | 0.275 |
| c3_CD4Tem_GZMK | SAMSN1 | 0 | 0 | 0.992825069 | 0.402 | 0.224 |
| c3_CD4Tem_GZMK | GPR183 | 0 | 0 | 0.983648097 | 0.457 | 0.248 |
| c3_CD4Tem_GZMK | LYAR | 0 | 0 | 0.942103474 | 0.612 | 0.329 |
| c3_CD4Tem_GZMK | S100A11 | 0 | 0 | 0.93051775 | 0.843 | 0.608 |
| c3_CD4Tem_GZMK | TRADD | 0 | 0 | 0.906799169 | 0.517 | 0.312 |
| c3_CD4Tem_GZMK | TNFAIP3 | 0 | 0 | 0.849226719 | 0.615 | 0.439 |
| c3_CD4Tem_GZMK | ANXA1 | 0 | 0 | 0.750593169 | 0.893 | 0.639 |
| c3_CD4Tem_GZMK | CXCR4 | 0 | 0 | 0.747718175 | 0.732 | 0.569 |
| c3_CD4Tem_GZMK | TRAT1 | 0 | 0 | 0.673478799 | 0.484 | 0.318 |
| c3_CD4Tem_GZMK | KLF6 | 0 | 0 | 0.621085758 | 0.807 | 0.644 |
| c3_CD4Tem_GZMK | LTB | 0 | 0 | 0.61477134 | 0.961 | 0.72 |
| c3_CD4Tem_GZMK | AQP3 | 0 | 0 | 0.60417037 | 0.533 | 0.341 |
| c3_CD4Tem_GZMK | PAG1 | 1.30E-182 | 3.91E-178 | 0.570247902 | 0.407 | 0.289 |
| c3_CD4Tem_GZMK | PLP2 | 8.02E-302 | 2.42E-297 | 0.566814757 | 0.615 | 0.455 |
| c3_CD4Tem_GZMK | PBXIP1 | 1.23E-259 | 3.71E-255 | 0.549744331 | 0.577 | 0.425 |
| c3_CD4Tem_GZMK | CD69 | 1.53E-254 | 4.60E-250 | 0.548021467 | 0.679 | 0.55 |
| c3_CD4Tem_GZMK | GATA3 | 3.84E-166 | 1.16E-161 | 0.54050297 | 0.411 | 0.297 |
| c3_CD4Tem_GZMK | ARHGAP15 | 4.78E-260 | 1.44E-255 | 0.52095484 | 0.723 | 0.596 |
| c3_CD4Tem_GZMK | TAGAP | 1.45E-244 | 4.37E-240 | 0.500541288 | 0.694 | 0.573 |
| c3_CD4Tem_GZMK | S100A10 | 0 | 0 | 0.494268367 | 0.933 | 0.827 |
| c3_CD4Tem_GZMK | EML4 | 9.61E-181 | 2.90E-176 | 0.488046752 | 0.601 | 0.488 |
| c3_CD4Tem_GZMK | AHNAK | 0 | 0 | 0.472158827 | 0.839 | 0.652 |
| c3_CD4Tem_GZMK | TXN | 5.65E-146 | 1.70E-141 | 0.468424867 | 0.5 | 0.386 |
| c3_CD4Tem_GZMK | CD2 | 1.58E-290 | 4.77E-286 | 0.460364835 | 0.769 | 0.638 |
| c3_CD4Tem_GZMK | DUSP2 | 7.48E-256 | 2.25E-251 | 0.458246083 | 0.501 | 0.344 |
| c4_CD4Teff_GZMH | GZMH | 0 | 0 | 2.100690762 | 0.993 | 0.305 |
| c4_CD4Teff_GZMH | FGFBP2 | 0 | 0 | 2.001075117 | 0.921 | 0.248 |
| c4_CD4Teff_GZMH | CX3CR1 | 0 | 0 | 1.954353734 | 0.696 | 0.189 |
| c4_CD4Teff_GZMH | GNLY | 0 | 0 | 1.76363972 | 0.857 | 0.276 |
| c4_CD4Teff_GZMH | LAIR2 | 0 | 0 | 1.749985776 | 0.417 | 0.129 |
| c4_CD4Teff_GZMH | CD320 | 0 | 0 | 1.663828403 | 0.485 | 0.193 |
| c4_CD4Teff_GZMH | PRSS23 | 0 | 0 | 1.581695394 | 0.432 | 0.129 |
| c4_CD4Teff_GZMH | GZMA | 0 | 0 | 1.505891487 | 0.953 | 0.392 |
| c4_CD4Teff_GZMH | EFHD2 | 0 | 0 | 1.487366587 | 0.767 | 0.353 |
| c4_CD4Teff_GZMH | LGALS1 | 0 | 0 | 1.467859642 | 0.785 | 0.364 |
| c4_CD4Teff_GZMH | HOPX | 0 | 0 | 1.450039419 | 0.652 | 0.251 |
| c4_CD4Teff_GZMH | NKG7 | 0 | 0 | 1.398624727 | 0.999 | 0.432 |
| c4_CD4Teff_GZMH | TBX21 | 0 | 0 | 1.396835467 | 0.487 | 0.179 |
| c4_CD4Teff_GZMH | PLEK | 0 | 0 | 1.380743338 | 0.735 | 0.281 |
| c4_CD4Teff_GZMH | ADGRG1 | 0 | 0 | 1.345891218 | 0.515 | 0.175 |
| c4_CD4Teff_GZMH | ZEB2 | 0 | 0 | 1.340128577 | 0.651 | 0.241 |
| c4_CD4Teff_GZMH | CTSC | 0 | 0 | 1.333538885 | 0.803 | 0.443 |
| c4_CD4Teff_GZMH | FCRL6 | 0 | 0 | 1.276456857 | 0.477 | 0.173 |
| c4_CD4Teff_GZMH | CD4 | 0 | 0 | 1.267193161 | 0.452 | 0.206 |
| c4_CD4Teff_GZMH | TGFBR3 | 0 | 0 | 1.264422684 | 0.517 | 0.225 |
| c4_CD4Teff_GZMH | SPON2 | 0 | 0 | 1.24729966 | 0.48 | 0.181 |
| c4_CD4Teff_GZMH | S1PR5 | 0 | 0 | 1.237926993 | 0.547 | 0.204 |
| c4_CD4Teff_GZMH | C12orf75 | 0 | 0 | 1.229105924 | 0.694 | 0.338 |
| c4_CD4Teff_GZMH | S100A4 | 0 | 0 | 1.225996445 | 0.997 | 0.839 |
| c4_CD4Teff_GZMH | HLA-DRB1 | 0 | 0 | 1.200398731 | 0.596 | 0.27 |
| c4_CD4Teff_GZMH | PRF1 | 0 | 0 | 1.186195993 | 0.88 | 0.386 |
| c4_CD4Teff_GZMH | ITGB1 | 0 | 0 | 1.1788739 | 0.766 | 0.426 |
| c4_CD4Teff_GZMH | HLA-DRB5 | 0 | 0 | 1.154027824 | 0.571 | 0.269 |
| c4_CD4Teff_GZMH | MYO1F | 0 | 0 | 1.124919024 | 0.583 | 0.298 |
| c4_CD4Teff_GZMH | GZMB | 0 | 0 | 1.118041032 | 0.693 | 0.26 |
| c5_CD4Treg_FOXP3 | FOXP3 | 0 | 0 | 4.171358098 | 0.483 | 0.018 |
| c5_CD4Treg_FOXP3 | CTLA4 | 0 | 0 | 2.845025811 | 0.432 | 0.056 |
| c5_CD4Treg_FOXP3 | STAM | 0 | 0 | 2.387848365 | 0.456 | 0.089 |
| c5_CD4Treg_FOXP3 | IKZF2 | 0 | 0 | 2.265648701 | 0.402 | 0.077 |
| c5_CD4Treg_FOXP3 | RNF214 | 0 | 0 | 1.772340282 | 0.408 | 0.116 |
| c5_CD4Treg_FOXP3 | TIGIT | 0 | 0 | 1.767771896 | 0.493 | 0.157 |
| c5_CD4Treg_FOXP3 | SHMT2 | 0 | 0 | 1.714270925 | 0.442 | 0.151 |
| c5_CD4Treg_FOXP3 | FAS | 0 | 0 | 1.431514367 | 0.407 | 0.151 |
| c5_CD4Treg_FOXP3 | LIMS1 | 0 | 0 | 1.423530222 | 0.471 | 0.223 |
| c5_CD4Treg_FOXP3 | GBP5 | 0 | 0 | 1.409601896 | 0.7 | 0.399 |
| c5_CD4Treg_FOXP3 | MAP3K1 | 0 | 0 | 1.39565375 | 0.429 | 0.169 |
| c5_CD4Treg_FOXP3 | HLA-DRA | 0 | 0 | 1.365495238 | 0.401 | 0.167 |
| c5_CD4Treg_FOXP3 | CLDND1 | 0 | 0 | 1.3262954 | 0.568 | 0.273 |
| c5_CD4Treg_FOXP3 | CORO1B | 0 | 0 | 1.300921945 | 0.604 | 0.326 |
| c5_CD4Treg_FOXP3 | EPSTI1 | 0 | 0 | 1.250723887 | 0.436 | 0.204 |
| c5_CD4Treg_FOXP3 | CYTOR | 0 | 0 | 1.246089512 | 0.507 | 0.233 |
| c5_CD4Treg_FOXP3 | CD28 | 0 | 0 | 1.216687878 | 0.492 | 0.222 |
| c5_CD4Treg_FOXP3 | ATP2B1 | 0 | 0 | 1.212983889 | 0.445 | 0.204 |
| c5_CD4Treg_FOXP3 | PHACTR2 | 0 | 0 | 1.212206373 | 0.403 | 0.176 |
| c5_CD4Treg_FOXP3 | COTL1 | 0 | 0 | 1.179020235 | 0.769 | 0.493 |
| c5_CD4Treg_FOXP3 | AQP3 | 0 | 0 | 1.168237096 | 0.615 | 0.338 |
| c5_CD4Treg_FOXP3 | OGDH | 0 | 0 | 1.163841932 | 0.42 | 0.193 |
| c5_CD4Treg_FOXP3 | USP15 | 0 | 0 | 1.15388073 | 0.736 | 0.478 |
| c5_CD4Treg_FOXP3 | TTC39C | 0 | 0 | 1.136542104 | 0.622 | 0.351 |
| c5_CD4Treg_FOXP3 | IL10RA | 0 | 0 | 1.135091975 | 0.756 | 0.451 |
| c5_CD4Treg_FOXP3 | GBP1 | 0 | 0 | 1.134524714 | 0.465 | 0.264 |
| c5_CD4Treg_FOXP3 | ISG20 | 0 | 0 | 1.13169739 | 0.848 | 0.591 |
| c5_CD4Treg_FOXP3 | HLA-DRB1 | 0 | 0 | 1.117789605 | 0.503 | 0.274 |
| c5_CD4Treg_FOXP3 | GBP2 | 0 | 0 | 1.111714153 | 0.626 | 0.383 |
| c5_CD4Treg_FOXP3 | S1PR4 | 0 | 0 | 1.104166792 | 0.632 | 0.399 |
| c6_CD8Tn_CCR7 | LINC02446 | 0 | 0 | 2.86509876 | 0.707 | 0.114 |
| c6_CD8Tn_CCR7 | NELL2 | 0 | 0 | 2.40882431 | 0.748 | 0.176 |
| c6_CD8Tn_CCR7 | AIF1 | 0 | 0 | 2.230939153 | 0.553 | 0.109 |
| c6_CD8Tn_CCR7 | CD8B | 0 | 0 | 2.019267187 | 0.95 | 0.259 |
| c6_CD8Tn_CCR7 | ACTN1 | 0 | 0 | 1.893470852 | 0.581 | 0.153 |
| c6_CD8Tn_CCR7 | BACH2 | 0 | 0 | 1.53098501 | 0.565 | 0.205 |
| c6_CD8Tn_CCR7 | PDE3B | 0 | 0 | 1.470544783 | 0.681 | 0.32 |
| c6_CD8Tn_CCR7 | CCR7 | 0 | 0 | 1.282746116 | 0.829 | 0.368 |
| c6_CD8Tn_CCR7 | NUCB2 | 0 | 0 | 1.2635541 | 0.658 | 0.303 |
| c6_CD8Tn_CCR7 | CD55 | 0 | 0 | 1.251815191 | 0.654 | 0.322 |
| c6_CD8Tn_CCR7 | LEF1 | 0 | 0 | 1.229489118 | 0.85 | 0.426 |
| c6_CD8Tn_CCR7 | TXK | 0 | 0 | 1.219642426 | 0.466 | 0.195 |
| c6_CD8Tn_CCR7 | OXNAD1 | 0 | 0 | 1.218769711 | 0.625 | 0.301 |
| c6_CD8Tn_CCR7 | TRABD2A | 0 | 0 | 1.17568198 | 0.509 | 0.216 |
| c6_CD8Tn_CCR7 | LDLRAP1 | 0 | 0 | 1.017799765 | 0.555 | 0.28 |
| c6_CD8Tn_CCR7 | ABLIM1 | 0 | 0 | 0.988126147 | 0.662 | 0.358 |
| c6_CD8Tn_CCR7 | MAML2 | 0 | 0 | 0.984655048 | 0.512 | 0.274 |
| c6_CD8Tn_CCR7 | SNHG8 | 0 | 0 | 0.96144249 | 0.763 | 0.485 |
| c6_CD8Tn_CCR7 | SERINC5 | 0 | 0 | 0.912731526 | 0.456 | 0.232 |
| c6_CD8Tn_CCR7 | RGS10 | 0 | 0 | 0.904144012 | 0.813 | 0.521 |
| c6_CD8Tn_CCR7 | THEMIS | 0 | 0 | 0.855116092 | 0.556 | 0.327 |
| c6_CD8Tn_CCR7 | SATB1 | 0 | 0 | 0.851795878 | 0.55 | 0.291 |
| c6_CD8Tn_CCR7 | TGFBR2 | 0 | 0 | 0.851320576 | 0.559 | 0.342 |
| c6_CD8Tn_CCR7 | PIK3IP1 | 0 | 0 | 0.84673186 | 0.781 | 0.487 |
| c6_CD8Tn_CCR7 | TCF7 | 0 | 0 | 0.820810818 | 0.876 | 0.553 |
| c6_CD8Tn_CCR7 | NDFIP1 | 0 | 0 | 0.766945785 | 0.788 | 0.562 |
| c6_CD8Tn_CCR7 | RCAN3 | 0 | 0 | 0.754945086 | 0.62 | 0.354 |
| c6_CD8Tn_CCR7 | CAMK4 | 0 | 0 | 0.750228217 | 0.736 | 0.461 |
| c6_CD8Tn_CCR7 | FOXP1 | 0 | 0 | 0.737970711 | 0.822 | 0.568 |
| c6_CD8Tn_CCR7 | NOSIP | 0 | 0 | 0.727895685 | 0.865 | 0.605 |
| c7_CD8Tcm_IL7R | CD8B | 0 | 0 | 1.393429978 | 0.873 | 0.298 |
| c7_CD8Tcm_IL7R | NELL2 | 0 | 0 | 1.220112432 | 0.512 | 0.215 |
| c7_CD8Tcm_IL7R | JUNB | 0 | 0 | 0.971204472 | 0.899 | 0.77 |
| c7_CD8Tcm_IL7R | GATA3 | 0 | 0 | 0.958633514 | 0.497 | 0.295 |
| c7_CD8Tcm_IL7R | KLRK1 | 0 | 0 | 0.915744011 | 0.534 | 0.229 |
| c7_CD8Tcm_IL7R | IL7R | 0 | 0 | 0.719853691 | 0.961 | 0.705 |
| c7_CD8Tcm_IL7R | COTL1 | 0 | 0 | 0.719278851 | 0.78 | 0.495 |
| c7_CD8Tcm_IL7R | PTGER2 | 8.24E-226 | 2.48E-221 | 0.715023094 | 0.41 | 0.249 |
| c7_CD8Tcm_IL7R | ARHGAP15 | 0 | 0 | 0.705081251 | 0.806 | 0.594 |
| c7_CD8Tcm_IL7R | CD8A | 0 | 0 | 0.663652223 | 0.821 | 0.335 |
| c7_CD8Tcm_IL7R | TRADD | 1.05E-268 | 3.17E-264 | 0.631092645 | 0.509 | 0.315 |
| c7_CD8Tcm_IL7R | PLP2 | 0 | 0 | 0.61048802 | 0.662 | 0.455 |
| c7_CD8Tcm_IL7R | STT3B | 7.73E-178 | 2.33E-173 | 0.569074213 | 0.416 | 0.267 |
| c7_CD8Tcm_IL7R | NOSIP | 0 | 0 | 0.567506564 | 0.81 | 0.62 |
| c7_CD8Tcm_IL7R | CLDND1 | 3.36E-189 | 1.01E-184 | 0.5654704 | 0.439 | 0.28 |
| c7_CD8Tcm_IL7R | DUSP1 | 6.35E-199 | 1.91E-194 | 0.545387857 | 0.633 | 0.466 |
| c7_CD8Tcm_IL7R | LTB | 0 | 0 | 0.530054312 | 0.964 | 0.723 |
| c7_CD8Tcm_IL7R | C1orf162 | 6.10E-160 | 1.84E-155 | 0.525798238 | 0.405 | 0.262 |
| c7_CD8Tcm_IL7R | CD69 | 2.52E-209 | 7.60E-205 | 0.494025826 | 0.715 | 0.551 |
| c7_CD8Tcm_IL7R | GAS5 | 2.78E-195 | 8.37E-191 | 0.488102407 | 0.601 | 0.444 |
| c7_CD8Tcm_IL7R | SELL | 2.18E-230 | 6.57E-226 | 0.479535375 | 0.728 | 0.544 |
| c7_CD8Tcm_IL7R | GPR183 | 3.84E-185 | 1.16E-180 | 0.475948634 | 0.41 | 0.252 |
| c7_CD8Tcm_IL7R | SNHG6 | 2.96E-144 | 8.90E-140 | 0.475043892 | 0.568 | 0.437 |
| c7_CD8Tcm_IL7R | CORO7 | 5.13E-133 | 1.55E-128 | 0.472849347 | 0.416 | 0.28 |
| c7_CD8Tcm_IL7R | TESPA1 | 1.32E-187 | 3.99E-183 | 0.470305508 | 0.502 | 0.333 |
| c7_CD8Tcm_IL7R | PAG1 | 3.76E-142 | 1.13E-137 | 0.468855568 | 0.432 | 0.289 |
| c7_CD8Tcm_IL7R | LDHB | 0 | 0 | 0.462332286 | 0.967 | 0.85 |
| c7_CD8Tcm_IL7R | TNFAIP3 | 1.70E-118 | 5.11E-114 | 0.455510957 | 0.574 | 0.443 |
| c7_CD8Tcm_IL7R | SLC2A3 | 7.05E-118 | 2.12E-113 | 0.453156 | 0.536 | 0.398 |
| c7_CD8Tcm_IL7R | RCAN3 | 4.66E-254 | 1.40E-249 | 0.449432486 | 0.585 | 0.37 |
| c8_CD8Tem_GZMK | GZMK | 0 | 0 | 3.271169271 | 0.879 | 0.146 |
| c8_CD8Tem_GZMK | DUSP2 | 0 | 0 | 1.92495636 | 0.758 | 0.333 |
| c8_CD8Tem_GZMK | CMC1 | 0 | 0 | 1.730396237 | 0.571 | 0.244 |
| c8_CD8Tem_GZMK | CD74 | 0 | 0 | 1.526308736 | 0.976 | 0.852 |
| c8_CD8Tem_GZMK | LYAR | 0 | 0 | 1.519500711 | 0.72 | 0.327 |
| c8_CD8Tem_GZMK | CXCR3 | 0 | 0 | 1.472727475 | 0.449 | 0.177 |
| c8_CD8Tem_GZMK | CD8A | 0 | 0 | 1.455893264 | 0.839 | 0.331 |
| c8_CD8Tem_GZMK | CCL5 | 0 | 0 | 1.369664123 | 0.996 | 0.473 |
| c8_CD8Tem_GZMK | CD8B | 0 | 0 | 1.313020381 | 0.768 | 0.299 |
| c8_CD8Tem_GZMK | HLA-DRB5 | 0 | 0 | 1.223018436 | 0.549 | 0.27 |
| c8_CD8Tem_GZMK | SH2D1A | 0 | 0 | 1.147871355 | 0.448 | 0.247 |
| c8_CD8Tem_GZMK | GZMA | 0 | 0 | 1.080497225 | 0.879 | 0.397 |
| c8_CD8Tem_GZMK | HLA-DPA1 | 0 | 0 | 0.978509837 | 0.707 | 0.419 |
| c8_CD8Tem_GZMK | CLDND1 | 7.86E-267 | 2.37E-262 | 0.962229328 | 0.415 | 0.28 |
| c8_CD8Tem_GZMK | HLA-DRB1 | 0 | 0 | 0.955334596 | 0.492 | 0.275 |
| c8_CD8Tem_GZMK | CBLB | 0 | 0 | 0.915150335 | 0.52 | 0.335 |
| c8_CD8Tem_GZMK | COTL1 | 0 | 0 | 0.888994799 | 0.668 | 0.498 |
| c8_CD8Tem_GZMK | CST7 | 0 | 0 | 0.887494335 | 0.95 | 0.454 |
| c8_CD8Tem_GZMK | CYTOR | 0 | 0 | 0.882591508 | 0.416 | 0.238 |
| c8_CD8Tem_GZMK | SAMD3 | 0 | 0 | 0.846086005 | 0.587 | 0.352 |
| c8_CD8Tem_GZMK | CXCR4 | 0 | 0 | 0.826349578 | 0.734 | 0.57 |
| c8_CD8Tem_GZMK | HLA-DPB1 | 0 | 0 | 0.786634948 | 0.705 | 0.458 |
| c8_CD8Tem_GZMK | PIK3R1 | 5.04E-293 | 1.52E-288 | 0.764440187 | 0.598 | 0.464 |
| c8_CD8Tem_GZMK | CCL4 | 0 | 0 | 0.746154101 | 0.493 | 0.241 |
| c8_CD8Tem_GZMK | GZMM | 0 | 0 | 0.742567039 | 0.843 | 0.606 |
| c8_CD8Tem_GZMK | FYN | 0 | 0 | 0.685808907 | 0.75 | 0.589 |
| c8_CD8Tem_GZMK | HERPUD1 | 4.39E-193 | 1.32E-188 | 0.679925123 | 0.419 | 0.296 |
| c8_CD8Tem_GZMK | TPST2 | 1.69E-288 | 5.10E-284 | 0.664889829 | 0.457 | 0.292 |
| c8_CD8Tem_GZMK | IL10RA | 0 | 0 | 0.641358978 | 0.614 | 0.458 |
| c8_CD8Tem_GZMK | ZBTB38 | 1.21E-189 | 3.66E-185 | 0.636449428 | 0.456 | 0.333 |
| c9_CD8Teff_ZNF683 | ZNF683 | 0 | 0 | 3.430676013 | 0.506 | 0.047 |
| c9_CD8Teff_ZNF683 | FGFBP2 | 0 | 0 | 2.530915487 | 0.936 | 0.212 |
| c9_CD8Teff_ZNF683 | PRSS23 | 0 | 0 | 2.422550611 | 0.549 | 0.102 |
| c9_CD8Teff_ZNF683 | GZMH | 0 | 0 | 2.386215679 | 0.994 | 0.27 |
| c9_CD8Teff_ZNF683 | GZMB | 0 | 0 | 2.380681015 | 0.919 | 0.215 |
| c9_CD8Teff_ZNF683 | GNLY | 0 | 0 | 2.326436975 | 0.917 | 0.241 |
| c9_CD8Teff_ZNF683 | CD8A | 0 | 0 | 2.315450205 | 0.955 | 0.293 |
| c9_CD8Teff_ZNF683 | ADGRG1 | 0 | 0 | 2.122034802 | 0.642 | 0.145 |
| c9_CD8Teff_ZNF683 | FCRL6 | 0 | 0 | 2.060582792 | 0.614 | 0.144 |
| c9_CD8Teff_ZNF683 | CX3CR1 | 0 | 0 | 2.006457709 | 0.659 | 0.167 |
| c9_CD8Teff_ZNF683 | NKG7 | 0 | 0 | 1.980030163 | 1 | 0.403 |
| c9_CD8Teff_ZNF683 | KLRD1 | 0 | 0 | 1.943993856 | 0.818 | 0.213 |
| c9_CD8Teff_ZNF683 | LGALS1 | 0 | 0 | 1.910726489 | 0.839 | 0.337 |
| c9_CD8Teff_ZNF683 | PRF1 | 0 | 0 | 1.807522108 | 0.92 | 0.357 |
| c9_CD8Teff_ZNF683 | ZEB2 | 0 | 0 | 1.781135497 | 0.7 | 0.215 |
| c9_CD8Teff_ZNF683 | SPON2 | 0 | 0 | 1.720244454 | 0.525 | 0.161 |
| c9_CD8Teff_ZNF683 | CD8B | 0 | 0 | 1.703318656 | 0.843 | 0.267 |
| c9_CD8Teff_ZNF683 | PLEK | 0 | 0 | 1.688454059 | 0.759 | 0.255 |
| c9_CD8Teff_ZNF683 | FCGR3A | 0 | 0 | 1.674242435 | 0.559 | 0.143 |
| c9_CD8Teff_ZNF683 | TBX21 | 0 | 0 | 1.642922489 | 0.504 | 0.162 |
| c9_CD8Teff_ZNF683 | EFHD2 | 0 | 0 | 1.612568816 | 0.764 | 0.332 |
| c9_CD8Teff_ZNF683 | CCL5 | 0 | 0 | 1.602512368 | 0.999 | 0.445 |
| c9_CD8Teff_ZNF683 | PATL2 | 0 | 0 | 1.59710564 | 0.407 | 0.156 |
| c9_CD8Teff_ZNF683 | C1orf21 | 0 | 0 | 1.595484051 | 0.414 | 0.122 |
| c9_CD8Teff_ZNF683 | CST7 | 0 | 0 | 1.587496951 | 0.988 | 0.424 |
| c9_CD8Teff_ZNF683 | GZMA | 0 | 0 | 1.576128066 | 0.935 | 0.365 |
| c9_CD8Teff_ZNF683 | FGR | 0 | 0 | 1.559868066 | 0.434 | 0.128 |
| c9_CD8Teff_ZNF683 | CTSW | 0 | 0 | 1.549092823 | 0.934 | 0.456 |
| c9_CD8Teff_ZNF683 | S1PR5 | 0 | 0 | 1.545587522 | 0.58 | 0.183 |
| c9_CD8Teff_ZNF683 | C12orf75 | 0 | 0 | 1.51046741 | 0.718 | 0.318 |
| c10_CD8Teff_NKG7 | KLRF1 | 0 | 0 | 2.816164503 | 0.471 | 0.054 |
| c10_CD8Teff_NKG7 | CMC1 | 0 | 0 | 2.786444132 | 0.812 | 0.205 |
| c10_CD8Teff_NKG7 | AOAH | 0 | 0 | 2.560667108 | 0.622 | 0.127 |
| c10_CD8Teff_NKG7 | FCRL6 | 0 | 0 | 2.243148298 | 0.668 | 0.14 |
| c10_CD8Teff_NKG7 | EOMES | 0 | 0 | 2.222461529 | 0.464 | 0.096 |
| c10_CD8Teff_NKG7 | FCGR3A | 0 | 0 | 2.221338168 | 0.647 | 0.136 |
| c10_CD8Teff_NKG7 | PTGDR | 0 | 0 | 2.17666587 | 0.431 | 0.085 |
| c10_CD8Teff_NKG7 | FGR | 0 | 0 | 2.171299263 | 0.556 | 0.118 |
| c10_CD8Teff_NKG7 | CCL4L2 | 0 | 0 | 2.132694366 | 0.451 | 0.081 |
| c10_CD8Teff_NKG7 | S1PR5 | 0 | 0 | 2.019154115 | 0.692 | 0.174 |
| c10_CD8Teff_NKG7 | PLEK | 0 | 0 | 2.013630406 | 0.831 | 0.25 |
| c10_CD8Teff_NKG7 | KLRD1 | 0 | 0 | 2.013500094 | 0.828 | 0.215 |
| c10_CD8Teff_NKG7 | NKG7 | 0 | 0 | 2.012767951 | 1 | 0.405 |
| c10_CD8Teff_NKG7 | TTC38 | 0 | 0 | 1.979840043 | 0.456 | 0.104 |
| c10_CD8Teff_NKG7 | GZMH | 0 | 0 | 1.941892084 | 0.953 | 0.277 |
| c10_CD8Teff_NKG7 | CST7 | 0 | 0 | 1.863771406 | 0.995 | 0.426 |
| c10_CD8Teff_NKG7 | KLRK1 | 0 | 0 | 1.859931348 | 0.647 | 0.201 |
| c10_CD8Teff_NKG7 | RHOC | 0 | 0 | 1.844080502 | 0.51 | 0.154 |
| c10_CD8Teff_NKG7 | CCL5 | 0 | 0 | 1.839269031 | 0.999 | 0.447 |
| c10_CD8Teff_NKG7 | CX3CR1 | 0 | 0 | 1.836147281 | 0.65 | 0.17 |
| c10_CD8Teff_NKG7 | CCL4 | 0 | 0 | 1.819239006 | 0.731 | 0.206 |
| c10_CD8Teff_NKG7 | TIGIT | 0 | 0 | 1.800584685 | 0.496 | 0.142 |
| c10_CD8Teff_NKG7 | ZEB2 | 0 | 0 | 1.78494101 | 0.722 | 0.215 |
| c10_CD8Teff_NKG7 | TBX21 | 0 | 0 | 1.7658562 | 0.551 | 0.159 |
| c10_CD8Teff_NKG7 | ADGRG1 | 0 | 0 | 1.758889769 | 0.584 | 0.152 |
| c10_CD8Teff_NKG7 | SLAMF7 | 0 | 0 | 1.735270228 | 0.411 | 0.109 |
| c10_CD8Teff_NKG7 | HLA-DRB1 | 0 | 0 | 1.728918257 | 0.7 | 0.245 |
| c10_CD8Teff_NKG7 | EFHD2 | 0 | 0 | 1.727423527 | 0.807 | 0.33 |
| c10_CD8Teff_NKG7 | ABI3 | 0 | 0 | 1.706501311 | 0.559 | 0.183 |
| c10_CD8Teff_NKG7 | GZMA | 0 | 0 | 1.70054529 | 0.961 | 0.365 |
| c11_CD8Teff_TNFSF13B | CREM | 0 | 0 | 2.747591995 | 0.482 | 0.168 |
| c11_CD8Teff_TNFSF13B | AD000090.1 | 0 | 0 | 2.631341282 | 0.84 | 0.336 |
| c11_CD8Teff_TNFSF13B | TNFSF13B | 0 | 0 | 2.543843951 | 0.846 | 0.373 |
| c11_CD8Teff_TNFSF13B | SAMSN1 | 0 | 0 | 2.444639518 | 0.448 | 0.228 |
| c11_CD8Teff_TNFSF13B | MTRNR2L1 | 0 | 0 | 2.426435984 | 0.818 | 0.33 |
| c11_CD8Teff_TNFSF13B | MTRNR2L12 | 0 | 0 | 2.402312214 | 0.933 | 0.413 |
| c11_CD8Teff_TNFSF13B | AMFR | 0 | 0 | 2.38652474 | 0.768 | 0.369 |
| c11_CD8Teff_TNFSF13B | MTRNR2L8 | 0 | 0 | 2.38474398 | 0.841 | 0.347 |
| c11_CD8Teff_TNFSF13B | CLDN11 | 0 | 0 | 2.324493072 | 0.717 | 0.278 |
| c11_CD8Teff_TNFSF13B | AXL | 0 | 0 | 2.30591436 | 0.413 | 0.16 |
| c11_CD8Teff_TNFSF13B | ATP1B3 | 0 | 0 | 2.282396283 | 0.43 | 0.23 |
| c11_CD8Teff_TNFSF13B | DAAM1 | 0 | 0 | 2.16616054 | 0.543 | 0.254 |
| c11_CD8Teff_TNFSF13B | ZNF688 | 0 | 0 | 2.137505576 | 0.714 | 0.367 |
| c11_CD8Teff_TNFSF13B | HSPA5 | 0 | 0 | 2.081238881 | 0.679 | 0.539 |
| c11_CD8Teff_TNFSF13B | REL | 0 | 0 | 2.057136966 | 0.482 | 0.319 |
| c11_CD8Teff_TNFSF13B | PDGFA | 0 | 0 | 2.048403251 | 0.482 | 0.21 |
| c11_CD8Teff_TNFSF13B | ARID5B | 0 | 0 | 2.034375713 | 0.837 | 0.539 |
| c11_CD8Teff_TNFSF13B | SCYL3 | 0 | 0 | 1.770647002 | 0.459 | 0.258 |
| c11_CD8Teff_TNFSF13B | KAT7 | 3.25E-236 | 9.78E-232 | 1.539777254 | 0.426 | 0.301 |
| c11_CD8Teff_TNFSF13B | FGF23 | 0 | 0 | 1.513795537 | 0.534 | 0.291 |
| c11_CD8Teff_TNFSF13B | CD8A | 6.69E-140 | 2.01E-135 | 1.030012501 | 0.463 | 0.35 |
| c11_CD8Teff_TNFSF13B | CYTIP | 4.79E-55 | 1.44E-50 | 0.866930607 | 0.528 | 0.631 |
| c11_CD8Teff_TNFSF13B | CD44 | 8.43E-67 | 2.54E-62 | 0.83132558 | 0.589 | 0.697 |
| c11_CD8Teff_TNFSF13B | H2AFZ | 4.93E-07 | 0.014860626 | 0.779716054 | 0.449 | 0.584 |
| c11_CD8Teff_TNFSF13B | SP100 | 1.79E-19 | 5.38E-15 | 0.777493876 | 0.458 | 0.561 |
| c11_CD8Teff_TNFSF13B | PGK1 | 3.06E-17 | 9.23E-13 | 0.71786745 | 0.511 | 0.673 |
| c11_CD8Teff_TNFSF13B | ELF1 | 8.62E-06 | 0.259636824 | 0.701102128 | 0.415 | 0.527 |
| c11_CD8Teff_TNFSF13B | AKAP13 | 8.48E-06 | 0.255613149 | 0.674021679 | 0.423 | 0.531 |
| c11_CD8Teff_TNFSF13B | HSP90AB1 | 3.15E-140 | 9.49E-136 | 0.672987776 | 0.746 | 0.865 |
| c11_CD8Teff_TNFSF13B | NCL | 9.28E-96 | 2.80E-91 | 0.672284849 | 0.664 | 0.801 |
| c12_CD8Tex_TIGIT | FXYD2 | 0 | 0 | 4.543151591 | 0.625 | 0.032 |
| c12_CD8Tex_TIGIT | AC004585.1 | 0 | 0 | 3.412762116 | 0.482 | 0.027 |
| c12_CD8Tex_TIGIT | SESTD1 | 0 | 0 | 3.408074092 | 0.45 | 0.013 |
| c12_CD8Tex_TIGIT | GZMK | 0 | 0 | 3.392995954 | 0.941 | 0.174 |
| c12_CD8Tex_TIGIT | HAVCR2 | 0 | 0 | 3.386892939 | 0.46 | 0.022 |
| c12_CD8Tex_TIGIT | LGALS3 | 0 | 0 | 3.076978344 | 0.581 | 0.092 |
| c12_CD8Tex_TIGIT | CD38 | 0 | 0 | 2.900361568 | 0.522 | 0.054 |
| c12_CD8Tex_TIGIT | AFF3 | 0 | 0 | 2.876192132 | 0.409 | 0.033 |
| c12_CD8Tex_TIGIT | PGM2L1 | 0 | 0 | 2.601588867 | 0.48 | 0.072 |
| c12_CD8Tex_TIGIT | NUCB2 | 0 | 0 | 2.460363005 | 0.883 | 0.331 |
| c12_CD8Tex_TIGIT | LYST | 0 | 0 | 2.438445258 | 0.691 | 0.173 |
| c12_CD8Tex_TIGIT | CD27 | 0 | 0 | 2.389858962 | 0.935 | 0.424 |
| c12_CD8Tex_TIGIT | CLDND1 | 0 | 0 | 2.347230551 | 0.707 | 0.284 |
| c12_CD8Tex_TIGIT | CTLA4 | 0 | 0 | 2.28713873 | 0.466 | 0.071 |
| c12_CD8Tex_TIGIT | DTHD1 | 0 | 0 | 2.284476798 | 0.455 | 0.065 |
| c12_CD8Tex_TIGIT | FABP5 | 0 | 0 | 2.278317369 | 0.499 | 0.077 |
| c12_CD8Tex_TIGIT | PECAM1 | 0 | 0 | 2.19774354 | 0.633 | 0.134 |
| c12_CD8Tex_TIGIT | FCMR | 0 | 0 | 2.104977145 | 0.96 | 0.589 |
| c12_CD8Tex_TIGIT | NCOA1 | 2.64E-289 | 7.96E-285 | 2.022271363 | 0.512 | 0.141 |
| c12_CD8Tex_TIGIT | TIGIT | 0 | 0 | 2.020735054 | 0.635 | 0.17 |
| c12_CD8Tex_TIGIT | GPR183 | 0 | 0 | 2.003540353 | 0.75 | 0.256 |
| c12_CD8Tex_TIGIT | SPTBN1 | 0 | 0 | 1.977581469 | 0.7 | 0.259 |
| c12_CD8Tex_TIGIT | SH2D1A | 0 | 0 | 1.966467029 | 0.741 | 0.253 |
| c12_CD8Tex_TIGIT | TTN | 4.27E-236 | 1.29E-231 | 1.962371855 | 0.428 | 0.115 |
| c12_CD8Tex_TIGIT | PHACTR2 | 3.64E-275 | 1.10E-270 | 1.954937567 | 0.578 | 0.184 |
| c12_CD8Tex_TIGIT | SESN3 | 1.16E-242 | 3.50E-238 | 1.922488202 | 0.543 | 0.18 |
| c12_CD8Tex_TIGIT | BRD7 | 1.04E-284 | 3.12E-280 | 1.907655568 | 0.659 | 0.239 |
| c12_CD8Tex_TIGIT | COTL1 | 7.96E-287 | 2.40E-282 | 1.831611992 | 0.858 | 0.504 |
| c12_CD8Tex_TIGIT | ISG20 | 0 | 0 | 1.818975758 | 0.921 | 0.601 |
| c12_CD8Tex_TIGIT | SEPTIN6 | 1.02E-197 | 3.08E-193 | 1.818448035 | 0.685 | 0.401 |
| c13_gdT_KLRC1 | TRDV2 | 0 | 0 | 5.542475761 | 0.956 | 0.031 |
| c13_gdT_KLRC1 | TRGV9 | 0 | 0 | 4.648714887 | 0.956 | 0.073 |
| c13_gdT_KLRC1 | KLRC1 | 0 | 0 | 4.048772718 | 0.665 | 0.035 |
| c13_gdT_KLRC1 | TRDC | 0 | 0 | 3.355456071 | 0.544 | 0.048 |
| c13_gdT_KLRC1 | TYROBP | 0 | 0 | 3.256442182 | 0.595 | 0.095 |
| c13_gdT_KLRC1 | FCGR3A | 0 | 0 | 2.812767903 | 0.796 | 0.15 |
| c13_gdT_KLRC1 | SPON2 | 0 | 0 | 2.690288316 | 0.72 | 0.168 |
| c13_gdT_KLRC1 | GNLY | 0 | 0 | 2.49777574 | 0.98 | 0.269 |
| c13_gdT_KLRC1 | FGFBP2 | 0 | 0 | 2.320808018 | 0.952 | 0.244 |
| c13_gdT_KLRC1 | KLRD1 | 0 | 0 | 2.192038835 | 0.89 | 0.238 |
| c13_gdT_KLRC1 | ADGRG1 | 0 | 0 | 2.183395395 | 0.674 | 0.166 |
| c13_gdT_KLRC1 | PRF1 | 0 | 0 | 2.167950121 | 0.951 | 0.381 |
| c13_gdT_KLRC1 | HOPX | 0 | 0 | 2.070584105 | 0.779 | 0.243 |
| c13_gdT_KLRC1 | GZMB | 0 | 0 | 2.064890025 | 0.924 | 0.247 |
| c13_gdT_KLRC1 | CX3CR1 | 0 | 0 | 2.026218935 | 0.695 | 0.188 |
| c13_gdT_KLRC1 | TTC38 | 0 | 0 | 2.020017662 | 0.465 | 0.118 |
| c13_gdT_KLRC1 | CD300A | 0 | 0 | 1.968207096 | 0.493 | 0.148 |
| c13_gdT_KLRC1 | TBX21 | 0 | 0 | 1.905571361 | 0.56 | 0.175 |
| c13_gdT_KLRC1 | EFHD2 | 0 | 0 | 1.893989192 | 0.839 | 0.348 |
| c13_gdT_KLRC1 | S1PR5 | 0 | 0 | 1.874617051 | 0.66 | 0.198 |
| c13_gdT_KLRC1 | PRSS23 | 0 | 0 | 1.856917321 | 0.479 | 0.126 |
| c13_gdT_KLRC1 | FGR | 0 | 0 | 1.852287009 | 0.497 | 0.139 |
| c13_gdT_KLRC1 | CD247 | 0 | 0 | 1.807212311 | 0.943 | 0.666 |
| c13_gdT_KLRC1 | NKG7 | 0 | 0 | 1.796889981 | 1 | 0.43 |
| c13_gdT_KLRC1 | CST7 | 0 | 0 | 1.795160775 | 0.992 | 0.45 |
| c13_gdT_KLRC1 | CTSW | 0 | 0 | 1.772490709 | 0.959 | 0.477 |
| c13_gdT_KLRC1 | MATK | 0 | 0 | 1.728916888 | 0.612 | 0.22 |
| c13_gdT_KLRC1 | C1orf21 | 0 | 0 | 1.712250099 | 0.439 | 0.134 |
| c13_gdT_KLRC1 | ZEB2 | 0 | 0 | 1.711839732 | 0.705 | 0.238 |
| c13_gdT_KLRC1 | PLEK | 0 | 0 | 1.709828747 | 0.774 | 0.278 |
| c14_gdT_GZMK | TRDV2 | 0 | 0 | 3.259338448 | 0.898 | 0.055 |
| c14_gdT_GZMK | TRGV9 | 0 | 0 | 2.720187523 | 0.878 | 0.097 |
| c14_gdT_GZMK | TRDC | 0 | 0 | 2.689063778 | 0.502 | 0.061 |
| c14_gdT_GZMK | KLRC1 | 0 | 0 | 2.560144229 | 0.445 | 0.056 |
| c14_gdT_GZMK | DUSP2 | 0 | 0 | 2.545049088 | 0.853 | 0.34 |
| c14_gdT_GZMK | GZMK | 0 | 0 | 2.115202121 | 0.715 | 0.165 |
| c14_gdT_GZMK | GABARAPL1 | 0 | 0 | 1.99851828 | 0.452 | 0.145 |
| c14_gdT_GZMK | NR4A2 | 0 | 0 | 1.910468385 | 0.468 | 0.179 |
| c14_gdT_GZMK | NFKBIA | 0 | 0 | 1.838468596 | 0.794 | 0.617 |
| c14_gdT_GZMK | MAP3K8 | 0 | 0 | 1.824789434 | 0.444 | 0.142 |
| c14_gdT_GZMK | DUSP1 | 0 | 0 | 1.812407444 | 0.711 | 0.466 |
| c14_gdT_GZMK | KLRB1 | 0 | 0 | 1.76712977 | 0.835 | 0.294 |
| c14_gdT_GZMK | FOS | 1.49E-297 | 4.48E-293 | 1.761926741 | 0.444 | 0.244 |
| c14_gdT_GZMK | PPP1R15A | 0 | 0 | 1.702103318 | 0.56 | 0.301 |
| c14_gdT_GZMK | ZFP36 | 0 | 0 | 1.689892668 | 0.813 | 0.652 |
| c14_gdT_GZMK | TNFAIP3 | 0 | 0 | 1.6315285 | 0.749 | 0.441 |
| c14_gdT_GZMK | JUN | 3.13E-273 | 9.42E-269 | 1.621276981 | 0.659 | 0.482 |
| c14_gdT_GZMK | BHLHE40 | 0 | 0 | 1.571141473 | 0.434 | 0.177 |
| c14_gdT_GZMK | CXCR4 | 0 | 0 | 1.502966912 | 0.847 | 0.571 |
| c14_gdT_GZMK | LYAR | 0 | 0 | 1.501171933 | 0.729 | 0.334 |
| c14_gdT_GZMK | GADD45B | 0 | 0 | 1.462994627 | 0.535 | 0.325 |
| c14_gdT_GZMK | SH2D2A | 0 | 0 | 1.380697031 | 0.561 | 0.31 |
| c14_gdT_GZMK | REL | 2.63E-304 | 7.91E-300 | 1.375273934 | 0.511 | 0.318 |
| c14_gdT_GZMK | CD69 | 0 | 0 | 1.311609258 | 0.745 | 0.552 |
| c14_gdT_GZMK | CCL4 | 0 | 0 | 1.298597082 | 0.528 | 0.245 |
| c14_gdT_GZMK | KLRD1 | 0 | 0 | 1.286222373 | 0.727 | 0.257 |
| c14_gdT_GZMK | KLRG1 | 0 | 0 | 1.27055912 | 0.717 | 0.336 |
| c14_gdT_GZMK | NINJ1 | 0 | 0 | 1.262783963 | 0.406 | 0.212 |
| c14_gdT_GZMK | SRGN | 0 | 0 | 1.254236632 | 0.948 | 0.74 |
| c14_gdT_GZMK | SQSTM1 | 6.33E-183 | 1.91E-178 | 1.22292309 | 0.625 | 0.507 |
| c15_NKT_FCGR3A | TYROBP | 0 | 0 | 1.878575838 | 0.569 | 0.112 |
| c15_NKT_FCGR3A | KLRF1 | 0 | 0 | 1.516003367 | 0.447 | 0.085 |
| c15_NKT_FCGR3A | AOAH | 0 | 0 | 1.291404169 | 0.573 | 0.164 |
| c15_NKT_FCGR3A | CLIC3 | 0 | 0 | 1.203804872 | 0.496 | 0.142 |
| c15_NKT_FCGR3A | FCGR3A | 0 | 0 | 1.11907 | 0.629 | 0.174 |
| c15_NKT_FCGR3A | GNLY | 0 | 0 | 1.076986853 | 0.878 | 0.293 |
| c15_NKT_FCGR3A | SPON2 | 0 | 0 | 1.053952245 | 0.572 | 0.188 |
| c15_NKT_FCGR3A | MCTP2 | 0 | 0 | 0.95111171 | 0.427 | 0.126 |
| c15_NKT_FCGR3A | FP671120.4 | 1.18E-230 | 3.57E-226 | 0.880212431 | 0.457 | 0.22 |
| c15_NKT_FCGR3A | RAP1GAP2 | 0 | 0 | 0.878362926 | 0.4 | 0.137 |
| c15_NKT_FCGR3A | RHOC | 0 | 0 | 0.868950743 | 0.53 | 0.18 |
| c15_NKT_FCGR3A | IFITM3 | 8.39E-199 | 2.53E-194 | 0.864242451 | 0.445 | 0.208 |
| c15_NKT_FCGR3A | GZMB | 0 | 0 | 0.857534235 | 0.84 | 0.27 |
| c15_NKT_FCGR3A | APOO | 1.21E-222 | 3.63E-218 | 0.840052378 | 0.492 | 0.246 |
| c15_NKT_FCGR3A | FP236383.3 | 3.14E-198 | 9.47E-194 | 0.83617797 | 0.459 | 0.231 |
| c15_NKT_FCGR3A | PRF1 | 0 | 0 | 0.798673976 | 0.912 | 0.4 |
| c15_NKT_FCGR3A | HOPX | 0 | 0 | 0.795574084 | 0.711 | 0.262 |
| c15_NKT_FCGR3A | CD300A | 0 | 0 | 0.793552883 | 0.465 | 0.16 |
| c15_NKT_FCGR3A | FGR | 0 | 0 | 0.778884976 | 0.51 | 0.15 |
| c15_NKT_FCGR3A | IL2RB | 1.72E-291 | 5.18E-287 | 0.763658648 | 0.548 | 0.232 |
| c15_NKT_FCGR3A | RBFOX2 | 4.70E-233 | 1.41E-228 | 0.761304984 | 0.439 | 0.196 |
| c15_NKT_FCGR3A | KLRD1 | 0 | 0 | 0.749811233 | 0.793 | 0.26 |
| c15_NKT_FCGR3A | CTSW | 0 | 0 | 0.73242854 | 0.937 | 0.493 |
| c15_NKT_FCGR3A | EFHD2 | 0 | 0 | 0.724870991 | 0.835 | 0.364 |
| c15_NKT_FCGR3A | TXK | 1.52E-237 | 4.57E-233 | 0.702792562 | 0.484 | 0.214 |
| c15_NKT_FCGR3A | IGF2R | 3.57E-303 | 1.07E-298 | 0.694255478 | 0.562 | 0.236 |
| c15_NKT_FCGR3A | RUNX3 | 0 | 0 | 0.692196337 | 0.679 | 0.316 |
| c15_NKT_FCGR3A | TTC38 | 0 | 0 | 0.686969466 | 0.423 | 0.13 |
| c15_NKT_FCGR3A | CTSD | 1.82E-268 | 5.47E-264 | 0.673207408 | 0.619 | 0.292 |
| c15_NKT_FCGR3A | LYST | 1.44E-273 | 4.35E-269 | 0.671079309 | 0.434 | 0.171 |
| c16_MAIT_SLC4A10 | SLC4A10 | 0 | 0 | 4.111049549 | 0.524 | 0.011 |
| c16_MAIT_SLC4A10 | TRAV1-2 | 0 | 0 | 3.252139456 | 0.606 | 0.045 |
| c16_MAIT_SLC4A10 | KLRB1 | 0 | 0 | 3.06332189 | 0.987 | 0.289 |
| c16_MAIT_SLC4A10 | NCR3 | 0 | 0 | 2.78567601 | 0.676 | 0.138 |
| c16_MAIT_SLC4A10 | FOS | 0 | 0 | 2.488287258 | 0.481 | 0.243 |
| c16_MAIT_SLC4A10 | DUSP1 | 0 | 0 | 2.473163261 | 0.761 | 0.465 |
| c16_MAIT_SLC4A10 | GZMK | 0 | 0 | 2.320267419 | 0.899 | 0.159 |
| c16_MAIT_SLC4A10 | DUSP2 | 0 | 0 | 2.149148097 | 0.7 | 0.343 |
| c16_MAIT_SLC4A10 | ZFP36 | 0 | 0 | 2.144625684 | 0.812 | 0.652 |
| c16_MAIT_SLC4A10 | TNFAIP3 | 0 | 0 | 1.908922783 | 0.719 | 0.441 |
| c16_MAIT_SLC4A10 | BHLHE40 | 0 | 0 | 1.906655172 | 0.488 | 0.175 |
| c16_MAIT_SLC4A10 | NR4A2 | 0 | 0 | 1.900820294 | 0.444 | 0.179 |
| c16_MAIT_SLC4A10 | NFKBIA | 0 | 0 | 1.768425099 | 0.771 | 0.617 |
| c16_MAIT_SLC4A10 | PPP1R15A | 0 | 0 | 1.731080605 | 0.512 | 0.302 |
| c16_MAIT_SLC4A10 | GPR65 | 0 | 0 | 1.712378272 | 0.583 | 0.232 |
| c16_MAIT_SLC4A10 | CXCR4 | 0 | 0 | 1.66830772 | 0.8 | 0.572 |
| c16_MAIT_SLC4A10 | GYG1 | 0 | 0 | 1.608502119 | 0.452 | 0.177 |
| c16_MAIT_SLC4A10 | CD69 | 0 | 0 | 1.594855128 | 0.779 | 0.551 |
| c16_MAIT_SLC4A10 | DDIT4 | 0 | 0 | 1.559093232 | 0.571 | 0.308 |
| c16_MAIT_SLC4A10 | IFNGR1 | 0 | 0 | 1.52021471 | 0.432 | 0.166 |
| c16_MAIT_SLC4A10 | ERN1 | 0 | 0 | 1.471234787 | 0.468 | 0.211 |
| c16_MAIT_SLC4A10 | SYTL2 | 0 | 0 | 1.451535254 | 0.411 | 0.161 |
| c16_MAIT_SLC4A10 | JUN | 1.07E-182 | 3.22E-178 | 1.409154625 | 0.62 | 0.483 |
| c16_MAIT_SLC4A10 | LYAR | 0 | 0 | 1.256325916 | 0.691 | 0.335 |
| c16_MAIT_SLC4A10 | AQP3 | 0 | 0 | 1.193459962 | 0.634 | 0.344 |
| c16_MAIT_SLC4A10 | GADD45B | 3.24E-196 | 9.78E-192 | 1.159794461 | 0.481 | 0.326 |
| c16_MAIT_SLC4A10 | KLRG1 | 0 | 0 | 1.148967344 | 0.698 | 0.336 |
| c16_MAIT_SLC4A10 | ID2 | 0 | 0 | 1.146522359 | 0.575 | 0.314 |
| c16_MAIT_SLC4A10 | IL7R | 0 | 0 | 1.110842449 | 0.97 | 0.708 |
| c16_MAIT_SLC4A10 | SPOCK2 | 0 | 0 | 1.106321171 | 0.863 | 0.641 |
| c17_Tprol_MKI67 | TYMS | 0 | 0 | 5.770015945 | 0.819 | 0.005 |
| c17_Tprol_MKI67 | MKI67 | 0 | 0 | 5.22858105 | 0.711 | 0.007 |
| c17_Tprol_MKI67 | RRM2 | 0 | 0 | 5.103410516 | 0.605 | 0.005 |
| c17_Tprol_MKI67 | STMN1 | 0 | 0 | 4.834465667 | 0.972 | 0.17 |
| c17_Tprol_MKI67 | HIST1H1B | 0 | 0 | 4.651064944 | 0.407 | 0.016 |
| c17_Tprol_MKI67 | PCLAF | 0 | 0 | 4.466188374 | 0.702 | 0.013 |
| c17_Tprol_MKI67 | TK1 | 0 | 0 | 4.397055436 | 0.638 | 0.005 |
| c17_Tprol_MKI67 | HIST1H2AI | 0 | 0 | 4.2069133 | 0.415 | 0.039 |
| c17_Tprol_MKI67 | NUSAP1 | 0 | 0 | 4.008163274 | 0.552 | 0.011 |
| c17_Tprol_MKI67 | CLSPN | 0 | 0 | 3.986525026 | 0.664 | 0.007 |
| c17_Tprol_MKI67 | UBE2C | 0 | 0 | 3.928153471 | 0.413 | 0.002 |
| c17_Tprol_MKI67 | TOP2A | 0 | 0 | 3.901773702 | 0.497 | 0.008 |
| c17_Tprol_MKI67 | CDT1 | 0 | 0 | 3.89308689 | 0.681 | 0.008 |
| c17_Tprol_MKI67 | UHRF1 | 0 | 0 | 3.887947669 | 0.599 | 0.005 |
| c17_Tprol_MKI67 | TPX2 | 0 | 0 | 3.748931359 | 0.513 | 0.007 |
| c17_Tprol_MKI67 | TUBA1B | 0 | 0 | 3.67078736 | 0.99 | 0.584 |
| c17_Tprol_MKI67 | GINS2 | 0 | 0 | 3.666314833 | 0.61 | 0.008 |
| c17_Tprol_MKI67 | CENPF | 0 | 0 | 3.66004794 | 0.468 | 0.013 |
| c17_Tprol_MKI67 | ASF1B | 0 | 0 | 3.596387782 | 0.575 | 0.007 |
| c17_Tprol_MKI67 | MCM2 | 0 | 0 | 3.564078649 | 0.649 | 0.016 |
| c17_Tprol_MKI67 | MCM4 | 0 | 0 | 3.532497894 | 0.667 | 0.023 |
| c17_Tprol_MKI67 | MCM7 | 0 | 0 | 3.525260845 | 0.826 | 0.099 |
| c17_Tprol_MKI67 | ASPM | 0 | 0 | 3.522465745 | 0.402 | 0.004 |
| c17_Tprol_MKI67 | CENPM | 0 | 0 | 3.47735746 | 0.645 | 0.028 |
| c17_Tprol_MKI67 | ZWINT | 0 | 0 | 3.450900828 | 0.6 | 0.011 |
| c17_Tprol_MKI67 | DHFR | 0 | 0 | 3.448254332 | 0.704 | 0.04 |
| c17_Tprol_MKI67 | PCNA | 0 | 0 | 3.436839082 | 0.784 | 0.092 |
| c17_Tprol_MKI67 | HMGB2 | 0 | 0 | 3.427510626 | 0.954 | 0.36 |
| c17_Tprol_MKI67 | BIRC5 | 0 | 0 | 3.421910835 | 0.453 | 0.002 |
| c17_Tprol_MKI67 | PKMYT1 | 0 | 0 | 3.410494856 | 0.444 | 0.001 |

T cell markers.

Supplementary Table 6. NK cell markers.

| **cluster** | **gene** | **p_val** | **p_val_adj** | **avg_log2FC** | **pct.1** | **pct.2** |
| --- | --- | --- | --- | --- | --- | --- |
| c18_NK_FCER1G | IGFBP7 | 0 | 0 | 1.791196852 | 0.627 | 0.236 |
| c18_NK_FCER1G | FCER1G | 0 | 0 | 1.766969123 | 0.93 | 0.392 |
| c18_NK_FCER1G | MYOM2 | 0 | 0 | 1.640362725 | 0.595 | 0.249 |
| c18_NK_FCER1G | AKR1C3 | 0 | 0 | 1.610934953 | 0.476 | 0.169 |
| c18_NK_FCER1G | SPON2 | 0 | 0 | 1.536830963 | 0.949 | 0.623 |
| c18_NK_FCER1G | CCL4 | 0 | 0 | 1.328697682 | 0.81 | 0.584 |
| c18_NK_FCER1G | CD38 | 0 | 0 | 1.272609326 | 0.457 | 0.234 |
| c18_NK_FCER1G | KLRB1 | 0 | 0 | 1.226195012 | 0.906 | 0.543 |
| c18_NK_FCER1G | LAIR2 | 0 | 0 | 1.036445322 | 0.549 | 0.343 |
| c18_NK_FCER1G | C1orf162 | 0 | 0 | 1.026777499 | 0.524 | 0.286 |
| c18_NK_FCER1G | CLIC3 | 0 | 0 | 1.012238254 | 0.83 | 0.623 |
| c18_NK_FCER1G | CEBPD | 0 | 0 | 0.914667414 | 0.439 | 0.276 |
| c18_NK_FCER1G | CD160 | 0 | 0 | 0.866976779 | 0.426 | 0.274 |
| c18_NK_FCER1G | SH2D1B | 0 | 0 | 0.813368259 | 0.677 | 0.465 |
| c18_NK_FCER1G | METRNL | 0 | 0 | 0.811665994 | 0.409 | 0.266 |
| c18_NK_FCER1G | LAT2 | 0 | 0 | 0.766863843 | 0.415 | 0.278 |
| c18_NK_FCER1G | CXXC5 | 0 | 0 | 0.744040134 | 0.428 | 0.291 |
| c18_NK_FCER1G | ALOX5AP | 0 | 0 | 0.724958339 | 0.712 | 0.529 |
| c18_NK_FCER1G | CX3CR1 | 0 | 0 | 0.684041403 | 0.73 | 0.589 |
| c18_NK_FCER1G | DIP2A | 5.71E-283 | 1.72E-278 | 0.661418769 | 0.426 | 0.312 |
| c18_NK_FCER1G | GLIPR2 | 0 | 0 | 0.660746344 | 0.471 | 0.344 |
| c18_NK_FCER1G | ICAM2 | 0 | 0 | 0.645852027 | 0.456 | 0.333 |
| c18_NK_FCER1G | MAP3K8 | 5.91E-269 | 1.78E-264 | 0.638515998 | 0.509 | 0.401 |
| c18_NK_FCER1G | CD7 | 0 | 0 | 0.63546134 | 0.967 | 0.839 |
| c18_NK_FCER1G | DDIT4 | 0 | 0 | 0.634227399 | 0.574 | 0.421 |
| c18_NK_FCER1G | RHOC | 0 | 0 | 0.630552491 | 0.677 | 0.568 |
| c18_NK_FCER1G | TTC38 | 0 | 0 | 0.629720147 | 0.663 | 0.502 |
| c18_NK_FCER1G | AGTRAP | 0 | 0 | 0.621179678 | 0.476 | 0.348 |
| c18_NK_FCER1G | GK5 | 2.31E-229 | 6.95E-225 | 0.618878863 | 0.425 | 0.323 |
| c18_NK_FCER1G | CARD16 | 0 | 0 | 0.599053583 | 0.602 | 0.477 |
| c19_NK_KLRC2 | KLRC2 | 0 | 0 | 1.97849342 | 0.798 | 0.379 |
| c19_NK_KLRC2 | CD3E | 0 | 0 | 1.635551544 | 0.914 | 0.382 |
| c19_NK_KLRC2 | CD52 | 0 | 0 | 1.294685091 | 0.96 | 0.621 |
| c19_NK_KLRC2 | TRG-AS1 | 0 | 0 | 1.105753391 | 0.558 | 0.282 |
| c19_NK_KLRC2 | HLA-DRB5 | 0 | 0 | 1.018739968 | 0.637 | 0.373 |
| c19_NK_KLRC2 | KLRC3 | 0 | 0 | 1.000822482 | 0.411 | 0.216 |
| c19_NK_KLRC2 | GZMH | 0 | 0 | 0.961115257 | 0.993 | 0.786 |
| c19_NK_KLRC2 | IL32 | 0 | 0 | 0.931218207 | 0.972 | 0.619 |
| c19_NK_KLRC2 | LGALS1 | 0 | 0 | 0.879664917 | 0.843 | 0.629 |
| c19_NK_KLRC2 | LIME1 | 0 | 0 | 0.862417769 | 0.645 | 0.421 |
| c19_NK_KLRC2 | HLA-DRB1 | 0 | 0 | 0.852920956 | 0.704 | 0.439 |
| c19_NK_KLRC2 | ITGB1 | 0 | 0 | 0.819644209 | 0.628 | 0.438 |
| c19_NK_KLRC2 | S100A6 | 0 | 0 | 0.818731617 | 0.979 | 0.848 |
| c19_NK_KLRC2 | SYNGR1 | 0 | 0 | 0.818715994 | 0.457 | 0.279 |
| c19_NK_KLRC2 | HLA-DRA | 0 | 0 | 0.70439914 | 0.411 | 0.249 |
| c19_NK_KLRC2 | CRIP1 | 0 | 0 | 0.662186867 | 0.856 | 0.724 |
| c19_NK_KLRC2 | HLA-DPA1 | 0 | 0 | 0.659271899 | 0.814 | 0.629 |
| c19_NK_KLRC2 | FGFBP2 | 0 | 0 | 0.626727783 | 0.973 | 0.757 |
| c19_NK_KLRC2 | VIM | 0 | 0 | 0.604913106 | 0.862 | 0.677 |
| c19_NK_KLRC2 | HLA-DPB1 | 0 | 0 | 0.59705695 | 0.813 | 0.648 |
| c19_NK_KLRC2 | CCL5 | 0 | 0 | 0.58375165 | 1 | 0.876 |
| c19_NK_KLRC2 | DSTN | 1.18E-281 | 3.54E-277 | 0.57300487 | 0.551 | 0.406 |
| c19_NK_KLRC2 | CD2 | 0 | 0 | 0.567371614 | 0.648 | 0.445 |
| c19_NK_KLRC2 | TRBC1 | 0 | 0 | 0.545400731 | 0.909 | 0.706 |
| c19_NK_KLRC2 | SSBP4 | 3.34E-178 | 1.01E-173 | 0.524796099 | 0.515 | 0.412 |
| c19_NK_KLRC2 | ANXA2 | 7.23E-285 | 2.18E-280 | 0.513794609 | 0.66 | 0.521 |
| c19_NK_KLRC2 | PRSS23 | 6.98E-266 | 2.10E-261 | 0.452430039 | 0.662 | 0.503 |
| c19_NK_KLRC2 | FCRL6 | 3.31E-233 | 9.98E-229 | 0.43121713 | 0.652 | 0.504 |
| c19_NK_KLRC2 | ZBTB38 | 2.22E-137 | 6.69E-133 | 0.427340053 | 0.475 | 0.37 |
| c19_NK_KLRC2 | ANXA1 | 0 | 0 | 0.412600839 | 0.878 | 0.771 |
| c20_NK_IL32 | KLRC1 | 7.67E-284 | 2.31E-279 | 0.747662242 | 0.507 | 0.362 |
| c20_NK_IL32 | IL32 | 0 | 0 | 0.7194379 | 0.912 | 0.643 |
| c20_NK_IL32 | FP236383.3 | 0 | 0 | 0.678183829 | 0.45 | 0.272 |
| c20_NK_IL32 | ZEB2 | 0 | 0 | 0.655046534 | 0.796 | 0.607 |
| c20_NK_IL32 | FP671120.4 | 8.18E-290 | 2.47E-285 | 0.614972585 | 0.417 | 0.251 |
| c20_NK_IL32 | GZMH | 0 | 0 | 0.577111549 | 0.982 | 0.794 |
| c20_NK_IL32 | RAP1GAP2 | 1.17E-161 | 3.52E-157 | 0.574373994 | 0.415 | 0.302 |
| c20_NK_IL32 | SPOCK2 | 5.92E-181 | 1.78E-176 | 0.532170625 | 0.455 | 0.327 |
| c20_NK_IL32 | PRKCB | 4.64E-170 | 1.40E-165 | 0.505983586 | 0.547 | 0.44 |
| c20_NK_IL32 | JAZF1 | 3.26E-136 | 9.83E-132 | 0.498997377 | 0.413 | 0.307 |
| c20_NK_IL32 | IKZF3 | 1.83E-179 | 5.52E-175 | 0.4981116 | 0.489 | 0.363 |
| c20_NK_IL32 | TRBC1 | 0 | 0 | 0.495766993 | 0.887 | 0.717 |
| c20_NK_IL32 | SYNGR1 | 1.58E-165 | 4.75E-161 | 0.479404575 | 0.419 | 0.292 |
| c20_NK_IL32 | FCGR3A | 0 | 0 | 0.475664988 | 0.954 | 0.813 |
| c20_NK_IL32 | TNFRSF1B | 1.57E-154 | 4.73E-150 | 0.47072013 | 0.481 | 0.363 |
| c20_NK_IL32 | TGFBR3 | 3.75E-177 | 1.13E-172 | 0.458289354 | 0.602 | 0.489 |
| c20_NK_IL32 | ADGRG1 | 1.19E-297 | 3.60E-293 | 0.457566531 | 0.756 | 0.593 |
| c20_NK_IL32 | FCRL6 | 1.10E-227 | 3.31E-223 | 0.451975109 | 0.657 | 0.507 |
| c20_NK_IL32 | SYNE2 | 9.04E-252 | 2.72E-247 | 0.427953169 | 0.759 | 0.623 |
| c20_NK_IL32 | ZBTB38 | 9.90E-131 | 2.98E-126 | 0.42170597 | 0.479 | 0.372 |
| c20_NK_IL32 | FGFBP2 | 0 | 0 | 0.40788811 | 0.964 | 0.765 |
| c20_NK_IL32 | PPP2R5C | 1.08E-177 | 3.26E-173 | 0.385911665 | 0.648 | 0.522 |
| c20_NK_IL32 | HLA-DRB5 | 4.75E-156 | 1.43E-151 | 0.357012695 | 0.533 | 0.404 |
| c20_NK_IL32 | CCL5 | 0 | 0 | 0.348406484 | 0.999 | 0.88 |
| c20_NK_IL32 | S100A6 | 0 | 0 | 0.347064531 | 0.962 | 0.856 |
| c20_NK_IL32 | CX3CR1 | 3.81E-200 | 1.15E-195 | 0.333733584 | 0.76 | 0.612 |
| c20_NK_IL32 | ANXA2 | 2.67E-120 | 8.05E-116 | 0.315170097 | 0.636 | 0.531 |
| c20_NK_IL32 | PRSS23 | 3.27E-135 | 9.85E-131 | 0.305586432 | 0.642 | 0.512 |
| c20_NK_IL32 | VIM | 9.73E-194 | 2.93E-189 | 0.280875214 | 0.821 | 0.691 |
| c21_NK_HLA-DRA | HLA-DQB1 | 0 | 0 | 1.970998183 | 0.426 | 0.117 |
| c21_NK_HLA-DRA | HLA-DRA | 0 | 0 | 1.947258787 | 0.614 | 0.235 |
| c21_NK_HLA-DRA | HLA-DQA1 | 0 | 0 | 1.605881336 | 0.449 | 0.169 |
| c21_NK_HLA-DRA | HLA-DRB1 | 0 | 0 | 1.585910064 | 0.748 | 0.457 |
| c21_NK_HLA-DRA | RBFOX2 | 0 | 0 | 1.436840765 | 0.445 | 0.17 |
| c21_NK_HLA-DRA | APOO | 0 | 0 | 1.236525042 | 0.522 | 0.243 |
| c21_NK_HLA-DRA | FP236383.3 | 0 | 0 | 1.21686632 | 0.575 | 0.265 |
| c21_NK_HLA-DRA | KLRC1 | 0 | 0 | 1.188115297 | 0.61 | 0.356 |
| c21_NK_HLA-DRA | PTMS | 0 | 0 | 1.187068175 | 0.433 | 0.18 |
| c21_NK_HLA-DRA | FP671120.4 | 0 | 0 | 1.037729171 | 0.535 | 0.244 |
| c21_NK_HLA-DRA | SH2D1A | 0 | 0 | 0.99486358 | 0.51 | 0.295 |
| c21_NK_HLA-DRA | VIM | 0 | 0 | 0.980487432 | 0.887 | 0.69 |
| c21_NK_HLA-DRA | KLRG1 | 0 | 0 | 0.96806342 | 0.502 | 0.29 |
| c21_NK_HLA-DRA | TRG-AS1 | 0 | 0 | 0.96624648 | 0.534 | 0.311 |
| c21_NK_HLA-DRA | TC2N | 0 | 0 | 0.86955453 | 0.483 | 0.271 |
| c21_NK_HLA-DRA | HLA-DPA1 | 0 | 0 | 0.753391766 | 0.819 | 0.645 |
| c21_NK_HLA-DRA | GAS5 | 4.15E-295 | 1.25E-290 | 0.745845411 | 0.469 | 0.279 |
| c21_NK_HLA-DRA | AC092821.3 | 2.05E-235 | 6.17E-231 | 0.738563114 | 0.407 | 0.241 |
| c21_NK_HLA-DRA | HLA-DPB1 | 0 | 0 | 0.733662213 | 0.829 | 0.66 |
| c21_NK_HLA-DRA | RPL36A | 0 | 0 | 0.706288195 | 0.91 | 0.769 |
| c21_NK_HLA-DRA | TIGIT | 1.24E-263 | 3.75E-259 | 0.703913665 | 0.493 | 0.306 |
| c21_NK_HLA-DRA | CD2 | 0 | 0 | 0.696730052 | 0.704 | 0.456 |
| c21_NK_HLA-DRA | CMC1 | 0 | 0 | 0.657197849 | 0.806 | 0.589 |
| c21_NK_HLA-DRA | SNHG6 | 2.99E-207 | 9.00E-203 | 0.634651103 | 0.519 | 0.354 |
| c21_NK_HLA-DRA | PDCD4 | 2.69E-190 | 8.10E-186 | 0.603890566 | 0.518 | 0.363 |
| c21_NK_HLA-DRA | LYST | 5.68E-177 | 1.71E-172 | 0.586198608 | 0.486 | 0.336 |
| c21_NK_HLA-DRA | EOMES | 3.42E-150 | 1.03E-145 | 0.564256834 | 0.453 | 0.315 |
| c21_NK_HLA-DRA | LYAR | 1.91E-130 | 5.77E-126 | 0.562154501 | 0.497 | 0.371 |
| c21_NK_HLA-DRA | CNN2 | 4.64E-206 | 1.40E-201 | 0.544002262 | 0.627 | 0.468 |
| c21_NK_HLA-DRA | CD81 | 3.94E-267 | 1.19E-262 | 0.539765574 | 0.755 | 0.597 |
| c22_NK_GZMK | IGFBP4 | 0 | 0 | 4.159929651 | 0.517 | 0.007 |
| c22_NK_GZMK | IL7R | 0 | 0 | 4.040671151 | 0.629 | 0.031 |
| c22_NK_GZMK | CAPG | 0 | 0 | 3.997331738 | 0.633 | 0.025 |
| c22_NK_GZMK | SPTSSB | 0 | 0 | 3.908215931 | 0.494 | 0.007 |
| c22_NK_GZMK | GZMK | 0 | 0 | 3.759852339 | 0.904 | 0.09 |
| c22_NK_GZMK | COTL1 | 0 | 0 | 3.687683205 | 0.815 | 0.096 |
| c22_NK_GZMK | GPR183 | 0 | 0 | 3.304461392 | 0.438 | 0.016 |
| c22_NK_GZMK | XCL1 | 0 | 0 | 3.228126037 | 0.895 | 0.186 |
| c22_NK_GZMK | SELL | 0 | 0 | 2.988315486 | 0.985 | 0.333 |
| c22_NK_GZMK | TCF7 | 0 | 0 | 2.841822767 | 0.744 | 0.116 |
| c22_NK_GZMK | ZMAT4 | 0 | 0 | 2.322401517 | 0.421 | 0.052 |
| c22_NK_GZMK | HVCN1 | 0 | 0 | 2.293447644 | 0.446 | 0.065 |
| c22_NK_GZMK | XCL2 | 0 | 0 | 2.169595581 | 0.801 | 0.277 |
| c22_NK_GZMK | LTB | 0 | 0 | 2.098620927 | 0.576 | 0.166 |
| c22_NK_GZMK | CXCR3 | 0 | 0 | 1.980480213 | 0.498 | 0.105 |
| c22_NK_GZMK | GAS5 | 0 | 0 | 1.860708225 | 0.715 | 0.274 |
| c22_NK_GZMK | IL18R1 | 0 | 0 | 1.817761899 | 0.492 | 0.125 |
| c22_NK_GZMK | CD44 | 0 | 0 | 1.728348637 | 0.854 | 0.392 |
| c22_NK_GZMK | IFITM3 | 0 | 0 | 1.715711281 | 0.938 | 0.508 |
| c22_NK_GZMK | TMEM123 | 0 | 0 | 1.661504374 | 0.641 | 0.208 |
| c22_NK_GZMK | ATP8B4 | 0 | 0 | 1.610218488 | 0.431 | 0.119 |
| c22_NK_GZMK | GSN | 0 | 0 | 1.603217867 | 0.591 | 0.169 |
| c22_NK_GZMK | CAPN12 | 0 | 0 | 1.512915045 | 0.419 | 0.12 |
| c22_NK_GZMK | MCUB | 0 | 0 | 1.476749257 | 0.553 | 0.176 |
| c22_NK_GZMK | NCAM1 | 0 | 0 | 1.474812413 | 0.679 | 0.294 |
| c22_NK_GZMK | CLDND1 | 0 | 0 | 1.423180408 | 0.552 | 0.205 |
| c22_NK_GZMK | SNHG29 | 0 | 0 | 1.394774251 | 0.734 | 0.366 |
| c22_NK_GZMK | ITGAX | 0 | 0 | 1.337373615 | 0.554 | 0.205 |
| c22_NK_GZMK | SMAP2 | 0 | 0 | 1.268869641 | 0.635 | 0.282 |
| c22_NK_GZMK | MMP25-AS1 | 0 | 0 | 1.255222653 | 0.441 | 0.169 |
| c23_NK_XCL1 | GZMK | 0 | 0 | 1.887206704 | 0.499 | 0.125 |
| c23_NK_XCL1 | CEBPD | 0 | 0 | 1.650493891 | 0.665 | 0.318 |
| c23_NK_XCL1 | FOS | 2.43E-131 | 7.32E-127 | 1.374844691 | 0.419 | 0.238 |
| c23_NK_XCL1 | XCL1 | 0 | 0 | 1.324625206 | 0.538 | 0.216 |
| c23_NK_XCL1 | CLDND1 | 8.61E-301 | 2.59E-296 | 1.276534068 | 0.48 | 0.214 |
| c23_NK_XCL1 | XCL2 | 0 | 0 | 1.178823005 | 0.605 | 0.296 |
| c23_NK_XCL1 | MCUB | 8.18E-231 | 2.46E-226 | 1.098316114 | 0.419 | 0.189 |
| c23_NK_XCL1 | IL2RB | 0 | 0 | 1.041836881 | 0.895 | 0.657 |
| c23_NK_XCL1 | IL18RAP | 4.05E-221 | 1.22E-216 | 0.995892677 | 0.503 | 0.254 |
| c23_NK_XCL1 | CD160 | 1.12E-185 | 3.38E-181 | 0.945807987 | 0.552 | 0.317 |
| c23_NK_XCL1 | IFNGR1 | 1.32E-166 | 3.97E-162 | 0.933106031 | 0.431 | 0.222 |
| c23_NK_XCL1 | DUSP1 | 3.16E-117 | 9.53E-113 | 0.90707594 | 0.67 | 0.488 |
| c23_NK_XCL1 | AGK | 9.28E-141 | 2.80E-136 | 0.894573766 | 0.403 | 0.216 |
| c23_NK_XCL1 | FCER1G | 0 | 0 | 0.876243344 | 0.946 | 0.565 |
| c23_NK_XCL1 | DUSP2 | 1.45E-44 | 4.36E-40 | 0.87458437 | 0.562 | 0.458 |
| c23_NK_XCL1 | CD69 | 8.41E-156 | 2.54E-151 | 0.868878184 | 0.714 | 0.512 |
| c23_NK_XCL1 | PIK3R1 | 3.24E-160 | 9.75E-156 | 0.852959475 | 0.673 | 0.465 |
| c23_NK_XCL1 | CXCR4 | 9.14E-106 | 2.75E-101 | 0.82622657 | 0.498 | 0.321 |
| c23_NK_XCL1 | MAP3K8 | 2.72E-157 | 8.20E-153 | 0.813331785 | 0.651 | 0.429 |
| c23_NK_XCL1 | EIF3G | 0 | 0 | 0.786721157 | 0.917 | 0.77 |
| c23_NK_XCL1 | CXXC5 | 3.76E-144 | 1.13E-139 | 0.757238267 | 0.54 | 0.329 |
| c23_NK_XCL1 | KLRB1 | 7.58E-302 | 2.28E-297 | 0.752561644 | 0.925 | 0.659 |
| c23_NK_XCL1 | SELL | 6.88E-209 | 2.07E-204 | 0.750367698 | 0.621 | 0.362 |
| c23_NK_XCL1 | MATK | 2.23E-235 | 6.73E-231 | 0.738322877 | 0.829 | 0.626 |
| c23_NK_XCL1 | SH2D1A | 6.59E-120 | 1.99E-115 | 0.734963173 | 0.506 | 0.313 |
| c23_NK_XCL1 | CNN2 | 6.93E-138 | 2.09E-133 | 0.711547993 | 0.671 | 0.479 |
| c23_NK_XCL1 | HSH2D | 9.21E-121 | 2.78E-116 | 0.666129011 | 0.62 | 0.424 |
| c23_NK_XCL1 | IFITM3 | 5.71E-181 | 1.72E-176 | 0.660944622 | 0.758 | 0.524 |
| c23_NK_XCL1 | C1orf162 | 2.81E-112 | 8.47E-108 | 0.636715797 | 0.556 | 0.361 |
| c23_NK_XCL1 | STK17A | 5.93E-127 | 1.79E-122 | 0.628990939 | 0.709 | 0.522 |
| c24_NK_RORA | CLDN11 | 4.57E-100 | 1.38E-95 | 2.223771652 | 0.412 | 0.2 |
| c24_NK_RORA | MTRNR2L1 | 7.13E-89 | 2.15E-84 | 2.204766601 | 0.463 | 0.251 |
| c24_NK_RORA | AMFR | 7.37E-85 | 2.22E-80 | 2.055229892 | 0.469 | 0.281 |
| c24_NK_RORA | ZNF688 | 8.52E-47 | 2.57E-42 | 1.961438785 | 0.405 | 0.285 |
| c24_NK_RORA | FGF23 | 2.03E-96 | 6.12E-92 | 1.949407456 | 0.462 | 0.243 |
| c24_NK_RORA | TNFSF13B | 4.64E-92 | 1.40E-87 | 1.948207137 | 0.477 | 0.269 |
| c24_NK_RORA | AD000090.1 | 6.62E-46 | 2.00E-41 | 1.845292144 | 0.41 | 0.254 |
| c24_NK_RORA | ARID5B | 3.02E-83 | 9.11E-79 | 1.826337957 | 0.581 | 0.424 |
| c24_NK_RORA | MTRNR2L12 | 6.99E-122 | 2.10E-117 | 1.78168037 | 0.611 | 0.339 |
| c24_NK_RORA | MTRNR2L8 | 1.08E-69 | 3.26E-65 | 1.753217559 | 0.46 | 0.266 |
| c24_NK_RORA | HSPA5 | 0.00833496 | 1 | 0.994689789 | 0.476 | 0.604 |
| c24_NK_RORA | SF3B1 | 9.26E-05 | 1 | 0.714162888 | 0.472 | 0.583 |
| c24_NK_RORA | SF1 | 4.53E-08 | 0.001364373 | 0.68487859 | 0.521 | 0.637 |
| c24_NK_RORA | MT-ND6 | 3.04E-15 | 9.16E-11 | 0.664283849 | 0.654 | 0.76 |
| c24_NK_RORA | TAPBP | 1.29E-05 | 0.390037292 | 0.6593574 | 0.519 | 0.655 |
| c24_NK_RORA | CFLAR | 3.29E-05 | 0.991426522 | 0.645635024 | 0.471 | 0.574 |
| c24_NK_RORA | IRF1 | 6.34E-05 | 1 | 0.634876393 | 0.605 | 0.736 |
| c24_NK_RORA | ADGRE5 | 0.009280702 | 1 | 0.627055639 | 0.484 | 0.616 |
| c24_NK_RORA | CAST | 3.98E-09 | 0.000119806 | 0.616031236 | 0.563 | 0.672 |
| c24_NK_RORA | SON | 2.47E-15 | 7.45E-11 | 0.612813374 | 0.631 | 0.745 |
| c24_NK_RORA | MT-ATP8 | 2.16E-17 | 6.50E-13 | 0.60405475 | 0.798 | 0.899 |
| c24_NK_RORA | GNG2 | 1.69E-07 | 0.005105848 | 0.600843641 | 0.58 | 0.701 |
| c24_NK_RORA | ARGLU1 | 0.000370869 | 1 | 0.585029697 | 0.467 | 0.583 |
| c24_NK_RORA | MYH9 | 3.79E-09 | 0.000114198 | 0.569518908 | 0.606 | 0.714 |
| c24_NK_RORA | LRRFIP1 | 0.002907121 | 1 | 0.568738913 | 0.494 | 0.629 |
| c24_NK_RORA | STK10 | 0.000749282 | 1 | 0.563569135 | 0.47 | 0.575 |
| c24_NK_RORA | RIPOR2 | 0.00286521 | 1 | 0.522119461 | 0.503 | 0.641 |
| c24_NK_RORA | TLN1 | 0.006165628 | 1 | 0.521645389 | 0.503 | 0.634 |
| c24_NK_RORA | STK4 | 0.000592376 | 1 | 0.520264105 | 0.524 | 0.675 |
| c24_NK_RORA | IQGAP1 | 0.000356813 | 1 | 0.505543412 | 0.571 | 0.716 |
| c25_NK_GATA3 | LTB | 0 | 0 | 5.010881323 | 0.972 | 0.188 |
| c25_NK_GATA3 | IL7R | 0 | 0 | 4.93677158 | 0.972 | 0.064 |
| c25_NK_GATA3 | KIT | 0 | 0 | 4.679597975 | 0.481 | 0.008 |
| c25_NK_GATA3 | TNFRSF4 | 0 | 0 | 4.25607477 | 0.586 | 0.036 |
| c25_NK_GATA3 | LST1 | 0 | 0 | 3.790677438 | 0.411 | 0.035 |
| c25_NK_GATA3 | INPP4B | 0 | 0 | 3.492300198 | 0.589 | 0.042 |
| c25_NK_GATA3 | FXYD7 | 0 | 0 | 3.451068043 | 0.488 | 0.035 |
| c25_NK_GATA3 | TNFRSF18 | 0 | 0 | 3.397792922 | 0.664 | 0.103 |
| c25_NK_GATA3 | AHR | 0 | 0 | 3.217813681 | 0.47 | 0.041 |
| c25_NK_GATA3 | TNFRSF25 | 0 | 0 | 3.144852662 | 0.463 | 0.03 |
| c25_NK_GATA3 | GPR183 | 0 | 0 | 3.054453247 | 0.486 | 0.04 |
| c25_NK_GATA3 | BIRC3 | 0 | 0 | 2.834063488 | 0.474 | 0.055 |
| c25_NK_GATA3 | CAPG | 0 | 0 | 2.773095041 | 0.54 | 0.061 |
| c25_NK_GATA3 | TCF7 | 3.53E-199 | 1.06E-194 | 2.678574398 | 0.659 | 0.153 |
| c25_NK_GATA3 | SSBP2 | 1.83E-273 | 5.52E-269 | 2.6301123 | 0.512 | 0.07 |
| c25_NK_GATA3 | LTA4H | 2.27E-134 | 6.84E-130 | 2.534771851 | 0.619 | 0.182 |
| c25_NK_GATA3 | GATA3 | 2.12E-154 | 6.40E-150 | 2.388225245 | 0.741 | 0.253 |
| c25_NK_GATA3 | TNFSF10 | 2.24E-127 | 6.74E-123 | 2.36274742 | 0.521 | 0.137 |
| c25_NK_GATA3 | MDFIC | 4.34E-159 | 1.31E-154 | 2.329903223 | 0.535 | 0.12 |
| c25_NK_GATA3 | TESPA1 | 5.33E-179 | 1.61E-174 | 2.322449773 | 0.558 | 0.12 |
| c25_NK_GATA3 | PLP2 | 1.78E-151 | 5.37E-147 | 2.176374462 | 0.629 | 0.169 |
| c25_NK_GATA3 | TMEM123 | 2.40E-187 | 7.22E-183 | 2.16118746 | 0.787 | 0.232 |
| c25_NK_GATA3 | SYPL1 | 1.38E-169 | 4.17E-165 | 2.143614004 | 0.435 | 0.075 |
| c25_NK_GATA3 | TIMP1 | 1.36E-160 | 4.09E-156 | 2.136732487 | 0.736 | 0.221 |
| c25_NK_GATA3 | NFKB1 | 4.08E-118 | 1.23E-113 | 2.056021741 | 0.57 | 0.166 |
| c25_NK_GATA3 | PLEKHO1 | 6.26E-122 | 1.89E-117 | 1.949413394 | 0.404 | 0.083 |
| c25_NK_GATA3 | FXYD5 | 1.61E-218 | 4.85E-214 | 1.924592832 | 0.995 | 0.834 |
| c25_NK_GATA3 | DDIT4 | 2.43E-80 | 7.33E-76 | 1.816032189 | 0.822 | 0.474 |
| c25_NK_GATA3 | FLT3LG | 2.46E-93 | 7.41E-89 | 1.67789406 | 0.612 | 0.218 |
| c25_NK_GATA3 | LDHB | 3.24E-112 | 9.77E-108 | 1.651612564 | 0.874 | 0.516 |

NK cell markers.

Supplementary Table 7. B cell markers.

| **cluster** | **gene** | **p_val** | **p_val_adj** | **avg_log2FC** | **pct.1** | **pct.2** |
| --- | --- | --- | --- | --- | --- | --- |
| c26_NaiveB_IL4R | IL4R | 0 | 0 | 2.014467694 | 0.811 | 0.351 |
| c26_NaiveB_IL4R | YBX3 | 0 | 0 | 1.226757977 | 0.485 | 0.287 |
| c26_NaiveB_IL4R | BACH2 | 0 | 0 | 1.210709177 | 0.809 | 0.556 |
| c26_NaiveB_IL4R | PLPP5 | 0 | 0 | 1.110891434 | 0.788 | 0.519 |
| c26_NaiveB_IL4R | ADK | 0 | 0 | 1.075907282 | 0.75 | 0.558 |
| c26_NaiveB_IL4R | CCR7 | 0 | 0 | 1.004197958 | 0.601 | 0.34 |
| c26_NaiveB_IL4R | SELL | 0 | 0 | 0.988946717 | 0.886 | 0.685 |
| c26_NaiveB_IL4R | FCRL3 | 0 | 0 | 0.984243193 | 0.447 | 0.288 |
| c26_NaiveB_IL4R | H1FX | 5.83E-303 | 1.74E-298 | 0.949347825 | 0.429 | 0.302 |
| c26_NaiveB_IL4R | COL19A1 | 0 | 0 | 0.90309612 | 0.46 | 0.329 |
| c26_NaiveB_IL4R | FCRL1 | 0 | 0 | 0.880851441 | 0.777 | 0.619 |
| c26_NaiveB_IL4R | CCND3 | 0 | 0 | 0.851168364 | 0.546 | 0.427 |
| c26_NaiveB_IL4R | APLP2 | 0 | 0 | 0.844050968 | 0.436 | 0.304 |
| c26_NaiveB_IL4R | LINC00926 | 0 | 0 | 0.835348773 | 0.891 | 0.767 |
| c26_NaiveB_IL4R | TLE5 | 0 | 0 | 0.823147046 | 0.695 | 0.519 |
| c26_NaiveB_IL4R | TSPAN13 | 0 | 0 | 0.756716952 | 0.483 | 0.352 |
| c26_NaiveB_IL4R | KLF2 | 0 | 0 | 0.723491521 | 0.852 | 0.742 |
| c26_NaiveB_IL4R | HVCN1 | 0 | 0 | 0.705424229 | 0.868 | 0.743 |
| c26_NaiveB_IL4R | CALHM6 | 0 | 0 | 0.696895972 | 0.6 | 0.492 |
| c26_NaiveB_IL4R | CXCR4 | 0 | 0 | 0.685539796 | 0.865 | 0.714 |
| c26_NaiveB_IL4R | FCER2 | 0 | 0 | 0.64895657 | 0.814 | 0.61 |
| c26_NaiveB_IL4R | IGHD | 0 | 0 | 0.599535614 | 0.713 | 0.522 |
| c26_NaiveB_IL4R | TCL1A | 0 | 0 | 0.56363652 | 0.913 | 0.545 |
| c26_NaiveB_IL4R | IGHM | 0 | 0 | 0.532866282 | 0.941 | 0.736 |
| c26_NaiveB_IL4R | NIBAN3 | 5.45E-297 | 1.63E-292 | 0.496244337 | 0.621 | 0.461 |
| c27_NaiveB_TCL1A | CD72 | 0 | 0 | 0.952847692 | 0.62 | 0.348 |
| c27_NaiveB_TCL1A | TCL1A | 0 | 0 | 0.770403309 | 0.948 | 0.567 |
| c27_NaiveB_TCL1A | FCER2 | 0 | 0 | 0.722899349 | 0.865 | 0.61 |
| c27_NaiveB_TCL1A | VPREB3 | 0 | 0 | 0.703456393 | 0.713 | 0.487 |
| c27_NaiveB_TCL1A | C1orf162 | 0 | 0 | 0.630921291 | 0.522 | 0.341 |
| c27_NaiveB_TCL1A | NCF1 | 0 | 0 | 0.595824638 | 0.946 | 0.84 |
| c27_NaiveB_TCL1A | MARCKSL1 | 7.48E-153 | 2.24E-148 | 0.53281041 | 0.424 | 0.315 |
| c27_NaiveB_TCL1A | SMIM14 | 7.73E-219 | 2.31E-214 | 0.478867593 | 0.55 | 0.407 |
| c27_NaiveB_TCL1A | CYB561A3 | 1.43E-243 | 4.27E-239 | 0.447263288 | 0.686 | 0.555 |
| c27_NaiveB_TCL1A | CD22 | 2.10E-248 | 6.28E-244 | 0.445106641 | 0.679 | 0.546 |
| c27_NaiveB_TCL1A | NIBAN3 | 6.78E-223 | 2.03E-218 | 0.420415611 | 0.626 | 0.475 |
| c27_NaiveB_TCL1A | COL19A1 | 9.75E-200 | 2.91E-195 | 0.419449808 | 0.478 | 0.335 |
| c27_NaiveB_TCL1A | CAMK2D | 5.51E-182 | 1.65E-177 | 0.414886961 | 0.521 | 0.388 |
| c27_NaiveB_TCL1A | CD69 | 1.54E-212 | 4.60E-208 | 0.411379008 | 0.637 | 0.49 |
| c27_NaiveB_TCL1A | IFITM2 | 4.26E-218 | 1.27E-213 | 0.410601309 | 0.714 | 0.602 |
| c27_NaiveB_TCL1A | IGHD | 0 | 0 | 0.403897323 | 0.748 | 0.527 |
| c27_NaiveB_TCL1A | APLP2 | 2.15E-159 | 6.42E-155 | 0.387652614 | 0.445 | 0.314 |
| c27_NaiveB_TCL1A | ITGB2 | 1.53E-129 | 4.58E-125 | 0.361475671 | 0.501 | 0.398 |
| c27_NaiveB_TCL1A | HVCN1 | 0 | 0 | 0.349544178 | 0.883 | 0.749 |
| c27_NaiveB_TCL1A | TSPAN13 | 2.51E-123 | 7.51E-119 | 0.330858964 | 0.483 | 0.365 |
| c27_NaiveB_TCL1A | SNX29 | 1.25E-146 | 3.75E-142 | 0.329945588 | 0.588 | 0.478 |
| c27_NaiveB_TCL1A | RHOH | 2.93E-154 | 8.75E-150 | 0.327241859 | 0.683 | 0.583 |
| c27_NaiveB_TCL1A | SNHG29 | 2.99E-112 | 8.93E-108 | 0.31984706 | 0.586 | 0.477 |
| c27_NaiveB_TCL1A | GAS5 | 5.84E-109 | 1.75E-104 | 0.307782208 | 0.578 | 0.47 |
| c27_NaiveB_TCL1A | PLPP5 | 2.20E-208 | 6.58E-204 | 0.303187336 | 0.725 | 0.57 |
| c27_NaiveB_TCL1A | FCRLA | 2.09E-125 | 6.25E-121 | 0.298529733 | 0.634 | 0.531 |
| c27_NaiveB_TCL1A | AFF3 | 5.09E-188 | 1.52E-183 | 0.250683834 | 0.805 | 0.688 |
| c28_NaiveB_TTN | CD1C | 6.38E-242 | 1.91E-237 | 1.730266274 | 0.523 | 0.14 |
| c28_NaiveB_TTN | TTN | 1.80E-219 | 5.39E-215 | 1.55547767 | 0.657 | 0.225 |
| c28_NaiveB_TTN | LRRK2 | 1.82E-151 | 5.45E-147 | 1.244608583 | 0.446 | 0.135 |
| c28_NaiveB_TTN | CBLB | 6.25E-127 | 1.87E-122 | 1.13643933 | 0.588 | 0.233 |
| c28_NaiveB_TTN | SMIM14 | 2.91E-157 | 8.70E-153 | 1.114950221 | 0.842 | 0.441 |
| c28_NaiveB_TTN | MYO1E | 5.75E-113 | 1.72E-108 | 1.111187494 | 0.447 | 0.159 |
| c28_NaiveB_TTN | S100A4 | 8.53E-132 | 2.55E-127 | 1.109446839 | 0.642 | 0.275 |
| c28_NaiveB_TTN | ANXA4 | 1.20E-105 | 3.59E-101 | 1.04482075 | 0.562 | 0.237 |
| c28_NaiveB_TTN | HDAC9 | 1.16E-84 | 3.46E-80 | 0.976372728 | 0.624 | 0.312 |
| c28_NaiveB_TTN | AUTS2 | 7.71E-88 | 2.31E-83 | 0.967767064 | 0.481 | 0.197 |
| c28_NaiveB_TTN | CR2 | 2.38E-84 | 7.10E-80 | 0.963211977 | 0.411 | 0.16 |
| c28_NaiveB_TTN | DENND5B | 1.53E-80 | 4.58E-76 | 0.951070852 | 0.473 | 0.205 |
| c28_NaiveB_TTN | CHI3L2 | 2.32E-81 | 6.94E-77 | 0.948814578 | 0.422 | 0.168 |
| c28_NaiveB_TTN | FCRL2 | 3.46E-98 | 1.03E-93 | 0.944748611 | 0.558 | 0.238 |
| c28_NaiveB_TTN | PLAC8 | 7.35E-95 | 2.20E-90 | 0.910466967 | 0.858 | 0.556 |
| c28_NaiveB_TTN | SMC6 | 1.08E-94 | 3.21E-90 | 0.909392465 | 0.719 | 0.365 |
| c28_NaiveB_TTN | LINC01857 | 5.07E-87 | 1.52E-82 | 0.908072085 | 0.539 | 0.241 |
| c28_NaiveB_TTN | SPTBN1 | 1.55E-91 | 4.64E-87 | 0.896597513 | 0.6 | 0.277 |
| c28_NaiveB_TTN | GRK3 | 1.23E-90 | 3.68E-86 | 0.887857446 | 0.471 | 0.188 |
| c28_NaiveB_TTN | KLHL14 | 1.12E-78 | 3.36E-74 | 0.885218643 | 0.414 | 0.164 |
| c28_NaiveB_TTN | LRMP | 5.00E-92 | 1.49E-87 | 0.879314658 | 0.632 | 0.301 |
| c28_NaiveB_TTN | CDK14 | 7.08E-62 | 2.12E-57 | 0.864704524 | 0.518 | 0.266 |
| c28_NaiveB_TTN | CAMK2D | 1.23E-81 | 3.67E-77 | 0.853534109 | 0.751 | 0.42 |
| c28_NaiveB_TTN | VAV3 | 1.30E-75 | 3.88E-71 | 0.851867364 | 0.552 | 0.255 |
| c28_NaiveB_TTN | SYNE2 | 7.97E-77 | 2.38E-72 | 0.843692024 | 0.499 | 0.224 |
| c28_NaiveB_TTN | PARP15 | 2.08E-95 | 6.20E-91 | 0.842494956 | 0.696 | 0.341 |
| c28_NaiveB_TTN | ETV6 | 6.45E-75 | 1.93E-70 | 0.84143714 | 0.452 | 0.194 |
| c28_NaiveB_TTN | MCTP2 | 3.02E-89 | 9.02E-85 | 0.832227513 | 0.563 | 0.244 |
| c28_NaiveB_TTN | NOTCH2 | 2.02E-72 | 6.05E-68 | 0.823415505 | 0.574 | 0.284 |
| c28_NaiveB_TTN | DPYD | 1.07E-78 | 3.21E-74 | 0.810049096 | 0.444 | 0.179 |
| c29_MemB_AIM2 | COCH | 0 | 0 | 3.361587231 | 0.401 | 0.02 |
| c29_MemB_AIM2 | AIM2 | 0 | 0 | 2.6297792 | 0.579 | 0.072 |
| c29_MemB_AIM2 | ITGB1 | 0 | 0 | 2.522090901 | 0.512 | 0.121 |
| c29_MemB_AIM2 | LINC01781 | 0 | 0 | 2.487324792 | 0.447 | 0.08 |
| c29_MemB_AIM2 | S100A10 | 0 | 0 | 2.394760689 | 0.743 | 0.219 |
| c29_MemB_AIM2 | CRIP1 | 0 | 0 | 2.248717562 | 0.807 | 0.31 |
| c29_MemB_AIM2 | S100A4 | 0 | 0 | 2.06501625 | 0.645 | 0.234 |
| c29_MemB_AIM2 | CD27 | 0 | 0 | 1.948208518 | 0.525 | 0.092 |
| c29_MemB_AIM2 | TNFRSF13B | 0 | 0 | 1.80829056 | 0.571 | 0.144 |
| c29_MemB_AIM2 | LGALS1 | 0 | 0 | 1.778454881 | 0.476 | 0.11 |
| c29_MemB_AIM2 | PDE4D | 0 | 0 | 1.643670816 | 0.51 | 0.178 |
| c29_MemB_AIM2 | ANXA2 | 0 | 0 | 1.638478824 | 0.628 | 0.212 |
| c29_MemB_AIM2 | CAPN2 | 0 | 0 | 1.634046212 | 0.43 | 0.112 |
| c29_MemB_AIM2 | ZBTB38 | 0 | 0 | 1.496686935 | 0.406 | 0.113 |
| c29_MemB_AIM2 | AHNAK | 0 | 0 | 1.447036965 | 0.779 | 0.38 |
| c29_MemB_AIM2 | SCIMP | 0 | 0 | 1.385322385 | 0.602 | 0.219 |
| c29_MemB_AIM2 | LYPLAL1 | 0 | 0 | 1.32147602 | 0.45 | 0.163 |
| c29_MemB_AIM2 | GAPDH | 0 | 0 | 1.213594493 | 0.981 | 0.745 |
| c29_MemB_AIM2 | CPNE5 | 0 | 0 | 1.206133901 | 0.604 | 0.268 |
| c29_MemB_AIM2 | S100A6 | 0 | 0 | 1.189466859 | 0.792 | 0.448 |
| c29_MemB_AIM2 | CD82 | 0 | 0 | 1.18908131 | 0.8 | 0.386 |
| c29_MemB_AIM2 | CD99 | 0 | 0 | 1.166726701 | 0.759 | 0.413 |
| c29_MemB_AIM2 | ACP5 | 0 | 0 | 1.153377257 | 0.566 | 0.227 |
| c29_MemB_AIM2 | MARCKS | 0 | 0 | 1.128524935 | 0.449 | 0.193 |
| c29_MemB_AIM2 | TKT | 0 | 0 | 1.124455647 | 0.617 | 0.308 |
| c29_MemB_AIM2 | UBE2N | 0 | 0 | 1.105320042 | 0.628 | 0.317 |
| c29_MemB_AIM2 | GSTK1 | 0 | 0 | 1.066167735 | 0.757 | 0.449 |
| c29_MemB_AIM2 | NAPSA | 0 | 0 | 1.054404215 | 0.403 | 0.161 |
| c29_MemB_AIM2 | CAPG | 0 | 0 | 1.051253499 | 0.666 | 0.334 |
| c29_MemB_AIM2 | ANXA5 | 0 | 0 | 1.016263668 | 0.473 | 0.19 |
| c30_MemB_TNFRSF13B | MTRNR2L1 | 0 | 0 | 1.533434352 | 0.579 | 0.258 |
| c30_MemB_TNFRSF13B | AD000090.1 | 0 | 0 | 1.527342375 | 0.538 | 0.241 |
| c30_MemB_TNFRSF13B | TNFRSF13B | 0 | 0 | 1.519677076 | 0.431 | 0.161 |
| c30_MemB_TNFRSF13B | MTRNR2L12 | 0 | 0 | 1.496169135 | 0.676 | 0.317 |
| c30_MemB_TNFRSF13B | MTRNR2L8 | 0 | 0 | 1.390129694 | 0.596 | 0.271 |
| c30_MemB_TNFRSF13B | LINC01857 | 0 | 0 | 1.379107709 | 0.487 | 0.214 |
| c30_MemB_TNFRSF13B | GPR183 | 0 | 0 | 1.32511417 | 0.409 | 0.184 |
| c30_MemB_TNFRSF13B | SEPT6 | 0 | 0 | 1.320709999 | 0.404 | 0.181 |
| c30_MemB_TNFRSF13B | TNFSF13B | 0 | 0 | 1.285555016 | 0.567 | 0.271 |
| c30_MemB_TNFRSF13B | CLDN11 | 0 | 0 | 1.279488536 | 0.478 | 0.217 |
| c30_MemB_TNFRSF13B | ARHGAP24 | 0 | 0 | 1.190564918 | 0.454 | 0.197 |
| c30_MemB_TNFRSF13B | FGF23 | 0 | 0 | 1.136371364 | 0.483 | 0.229 |
| c30_MemB_TNFRSF13B | ZNF688 | 0 | 0 | 1.117113974 | 0.54 | 0.312 |
| c30_MemB_TNFRSF13B | AHNAK | 0 | 0 | 1.028378618 | 0.618 | 0.401 |
| c30_MemB_TNFRSF13B | AMFR | 1.61E-305 | 4.82E-301 | 1.027663087 | 0.646 | 0.43 |
| c30_MemB_TNFRSF13B | SEPT7 | 6.61E-301 | 1.98E-296 | 1.026748561 | 0.407 | 0.2 |
| c30_MemB_TNFRSF13B | DAAM1 | 1.14E-233 | 3.42E-229 | 1.010144322 | 0.44 | 0.255 |
| c30_MemB_TNFRSF13B | COTL1 | 0 | 0 | 0.965673805 | 0.755 | 0.541 |
| c30_MemB_TNFRSF13B | FAM129C | 0 | 0 | 0.962349551 | 0.436 | 0.204 |
| c30_MemB_TNFRSF13B | KLF6 | 2.55E-242 | 7.62E-238 | 0.902239503 | 0.698 | 0.524 |
| c30_MemB_TNFRSF13B | PPP1R15A | 2.46E-216 | 7.36E-212 | 0.885648842 | 0.583 | 0.403 |
| c30_MemB_TNFRSF13B | KAT7 | 1.14E-155 | 3.41E-151 | 0.870041845 | 0.4 | 0.25 |
| c30_MemB_TNFRSF13B | CCDC50 | 6.74E-178 | 2.01E-173 | 0.837941755 | 0.473 | 0.308 |
| c30_MemB_TNFRSF13B | CD82 | 4.34E-276 | 1.30E-271 | 0.835899629 | 0.62 | 0.408 |
| c30_MemB_TNFRSF13B | SLC2A3 | 3.58E-148 | 1.07E-143 | 0.832605437 | 0.403 | 0.254 |
| c30_MemB_TNFRSF13B | VOPP1 | 3.09E-180 | 9.24E-176 | 0.823192158 | 0.435 | 0.265 |
| c30_MemB_TNFRSF13B | PCBP2 | 0 | 0 | 0.806366402 | 0.777 | 0.619 |
| c30_MemB_TNFRSF13B | ATP5F1A | 1.82E-292 | 5.42E-288 | 0.802941238 | 0.788 | 0.624 |
| c30_MemB_TNFRSF13B | ZFP36 | 1.83E-227 | 5.47E-223 | 0.778404433 | 0.809 | 0.702 |
| c30_MemB_TNFRSF13B | CD83 | 1.65E-103 | 4.93E-99 | 0.757705958 | 0.542 | 0.422 |
| c31_MemB_FCRL5 | SOX5 | 0 | 0 | 2.86870325 | 0.428 | 0.05 |
| c31_MemB_FCRL5 | RGS2 | 0 | 0 | 2.686993948 | 0.419 | 0.058 |
| c31_MemB_FCRL5 | FGR | 0 | 0 | 2.544567017 | 0.812 | 0.23 |
| c31_MemB_FCRL5 | FCRL5 | 0 | 0 | 2.464398485 | 0.639 | 0.181 |
| c31_MemB_FCRL5 | TNFRSF1B | 0 | 0 | 2.448854546 | 0.623 | 0.139 |
| c31_MemB_FCRL5 | RHOB | 0 | 0 | 2.438953585 | 0.512 | 0.111 |
| c31_MemB_FCRL5 | CIB1 | 0 | 0 | 2.32125521 | 0.895 | 0.455 |
| c31_MemB_FCRL5 | HCK | 0 | 0 | 2.302694071 | 0.647 | 0.137 |
| c31_MemB_FCRL5 | ADGRE5 | 0 | 0 | 2.189609636 | 0.526 | 0.128 |
| c31_MemB_FCRL5 | S100A11 | 0 | 0 | 2.134818086 | 0.496 | 0.129 |
| c31_MemB_FCRL5 | CLECL1 | 0 | 0 | 2.132967433 | 0.512 | 0.115 |
| c31_MemB_FCRL5 | GRN | 0 | 0 | 2.046718069 | 0.675 | 0.236 |
| c31_MemB_FCRL5 | ZEB2 | 0 | 0 | 1.945266042 | 0.816 | 0.337 |
| c31_MemB_FCRL5 | LGALS1 | 2.66E-204 | 7.94E-200 | 1.857051915 | 0.439 | 0.145 |
| c31_MemB_FCRL5 | NEAT1 | 0 | 0 | 1.846703794 | 0.752 | 0.317 |
| c31_MemB_FCRL5 | PLEK | 3.30E-298 | 9.86E-294 | 1.783561307 | 0.711 | 0.295 |
| c31_MemB_FCRL5 | PPP1R14A | 1.13E-183 | 3.36E-179 | 1.757954739 | 0.488 | 0.187 |
| c31_MemB_FCRL5 | NAPSA | 2.03E-150 | 6.06E-146 | 1.750517343 | 0.444 | 0.183 |
| c31_MemB_FCRL5 | IFI30 | 3.54E-46 | 1.06E-41 | 1.731635471 | 0.445 | 0.327 |
| c31_MemB_FCRL5 | IL10RA | 5.43E-254 | 1.62E-249 | 1.659006513 | 0.556 | 0.188 |
| c31_MemB_FCRL5 | ITGB2 | 7.64E-275 | 2.28E-270 | 1.644317843 | 0.793 | 0.418 |
| c31_MemB_FCRL5 | NR4A2 | 2.91E-224 | 8.69E-220 | 1.631581648 | 0.433 | 0.129 |
| c31_MemB_FCRL5 | PSAP | 0 | 0 | 1.630681279 | 0.912 | 0.555 |
| c31_MemB_FCRL5 | SRGN | 3.03E-220 | 9.05E-216 | 1.609346455 | 0.488 | 0.159 |
| c31_MemB_FCRL5 | HSPB1 | 6.68E-125 | 2.00E-120 | 1.591782988 | 0.457 | 0.197 |
| c31_MemB_FCRL5 | DAPP1 | 1.89E-221 | 5.65E-217 | 1.588929537 | 0.706 | 0.351 |
| c31_MemB_FCRL5 | LRMP | 5.72E-196 | 1.71E-191 | 1.572584095 | 0.639 | 0.299 |
| c31_MemB_FCRL5 | ITGB2-AS1 | 1.11E-203 | 3.32E-199 | 1.566998785 | 0.482 | 0.164 |
| c31_MemB_FCRL5 | CYTH4 | 7.21E-219 | 2.16E-214 | 1.554425836 | 0.529 | 0.186 |
| c31_MemB_FCRL5 | FCRL2 | 2.10E-223 | 6.27E-219 | 1.542661122 | 0.602 | 0.235 |
| c32_MemB_TNF | CDKN1A | 0 | 0 | 4.187334549 | 0.873 | 0.094 |
| c32_MemB_TNF | LMNA | 0 | 0 | 4.013740201 | 0.548 | 0.031 |
| c32_MemB_TNF | MIR155HG | 0 | 0 | 3.928879155 | 0.428 | 0.053 |
| c32_MemB_TNF | RGS16 | 0 | 0 | 3.815998224 | 0.444 | 0.023 |
| c32_MemB_TNF | SAMSN1 | 0 | 0 | 3.797354242 | 0.757 | 0.074 |
| c32_MemB_TNF | NINJ1 | 0 | 0 | 3.763042694 | 0.493 | 0.024 |
| c32_MemB_TNF | SEMA7A | 0 | 0 | 3.700970067 | 0.614 | 0.032 |
| c32_MemB_TNF | SQSTM1 | 0 | 0 | 3.697248457 | 0.974 | 0.458 |
| c32_MemB_TNF | HMOX1 | 0 | 0 | 3.67907098 | 0.408 | 0.036 |
| c32_MemB_TNF | CD58 | 0 | 0 | 3.654740352 | 0.689 | 0.069 |
| c32_MemB_TNF | BHLHE40 | 0 | 0 | 3.575462717 | 0.594 | 0.036 |
| c32_MemB_TNF | TNF | 0 | 0 | 3.549857106 | 0.51 | 0.034 |
| c32_MemB_TNF | KDM6B | 0 | 0 | 3.49903372 | 0.769 | 0.113 |
| c32_MemB_TNF | CREM | 0 | 0 | 3.458535196 | 0.588 | 0.077 |
| c32_MemB_TNF | ICAM1 | 0 | 0 | 3.39849663 | 0.455 | 0.03 |
| c32_MemB_TNF | ARHGAP31 | 0 | 0 | 3.352329096 | 0.499 | 0.037 |
| c32_MemB_TNF | SLC3A2 | 0 | 0 | 3.330646661 | 0.822 | 0.169 |
| c32_MemB_TNF | CYSTM1 | 0 | 0 | 3.292620919 | 0.627 | 0.063 |
| c32_MemB_TNF | CD83 | 0 | 0 | 3.264805309 | 0.98 | 0.426 |
| c32_MemB_TNF | ATP1B3 | 0 | 0 | 3.185234913 | 0.665 | 0.109 |
| c32_MemB_TNF | PLK3 | 0 | 0 | 3.067892455 | 0.446 | 0.026 |
| c32_MemB_TNF | SLC7A5 | 0 | 0 | 3.060253976 | 0.593 | 0.07 |
| c32_MemB_TNF | FABP5 | 0 | 0 | 3.048189648 | 0.426 | 0.073 |
| c32_MemB_TNF | ID3 | 0 | 0 | 3.04104989 | 0.746 | 0.206 |
| c32_MemB_TNF | LY9 | 0 | 0 | 2.978031418 | 0.775 | 0.238 |
| c32_MemB_TNF | NR4A2 | 6.60E-164 | 1.97E-159 | 2.966000821 | 0.424 | 0.131 |
| c32_MemB_TNF | TRAF4 | 0 | 0 | 2.926892886 | 0.739 | 0.15 |
| c32_MemB_TNF | RARA | 0 | 0 | 2.904375384 | 0.68 | 0.104 |
| c32_MemB_TNF | SKIL | 0 | 0 | 2.85969511 | 0.765 | 0.147 |
| c32_MemB_TNF | MTHFD2 | 0 | 0 | 2.777975287 | 0.527 | 0.073 |
| c33_gcB_CD38 | NEIL1 | 0 | 0 | 2.368464739 | 0.509 | 0.101 |
| c33_gcB_CD38 | PLD4 | 0 | 0 | 2.136845148 | 0.812 | 0.273 |
| c33_gcB_CD38 | LIMS2 | 0 | 0 | 2.072845865 | 0.417 | 0.091 |
| c33_gcB_CD38 | AL139020.1 | 0 | 0 | 2.026260098 | 0.475 | 0.111 |
| c33_gcB_CD38 | PPP1R14A | 0 | 0 | 2.01233433 | 0.615 | 0.175 |
| c33_gcB_CD38 | ITM2C | 0 | 0 | 1.973265739 | 0.692 | 0.181 |
| c33_gcB_CD38 | CCDC191 | 0 | 0 | 1.75969654 | 0.443 | 0.135 |
| c33_gcB_CD38 | JUP | 0 | 0 | 1.75375408 | 0.588 | 0.182 |
| c33_gcB_CD38 | CD38 | 0 | 0 | 1.685410782 | 0.51 | 0.149 |
| c33_gcB_CD38 | PCDH9 | 0 | 0 | 1.650399677 | 0.575 | 0.198 |
| c33_gcB_CD38 | HCK | 0 | 0 | 1.624708101 | 0.421 | 0.136 |
| c33_gcB_CD38 | TCL1A | 0 | 0 | 1.416272092 | 0.994 | 0.662 |
| c33_gcB_CD38 | PMEPA1 | 2.44E-257 | 7.30E-253 | 1.350105976 | 0.415 | 0.151 |
| c33_gcB_CD38 | PPP1R14B | 2.46E-233 | 7.35E-229 | 1.332790281 | 0.412 | 0.161 |
| c33_gcB_CD38 | HMCES | 5.84E-229 | 1.75E-224 | 1.259891809 | 0.411 | 0.156 |
| c33_gcB_CD38 | CMTM7 | 6.21E-223 | 1.85E-218 | 1.247931572 | 0.549 | 0.268 |
| c33_gcB_CD38 | SPIB | 1.18E-301 | 3.54E-297 | 1.206608778 | 0.806 | 0.491 |
| c33_gcB_CD38 | FCRLA | 0 | 0 | 1.197630819 | 0.883 | 0.545 |
| c33_gcB_CD38 | LBR | 4.50E-181 | 1.35E-176 | 1.091314994 | 0.559 | 0.298 |
| c33_gcB_CD38 | VPREB3 | 3.00E-270 | 8.97E-266 | 1.055283822 | 0.841 | 0.538 |
| c33_gcB_CD38 | MZB1 | 0 | 0 | 1.029846223 | 0.675 | 0.244 |
| c33_gcB_CD38 | YBX3 | 1.02E-199 | 3.05E-195 | 0.992689523 | 0.654 | 0.344 |
| c33_gcB_CD38 | NIBAN3 | 3.86E-240 | 1.15E-235 | 0.981598163 | 0.784 | 0.506 |
| c33_gcB_CD38 | CD72 | 1.13E-239 | 3.37E-235 | 0.979389101 | 0.743 | 0.411 |
| c33_gcB_CD38 | MBD2 | 9.33E-134 | 2.79E-129 | 0.936407076 | 0.516 | 0.288 |
| c33_gcB_CD38 | SMIM14 | 8.46E-191 | 2.53E-186 | 0.913479074 | 0.722 | 0.435 |
| c33_gcB_CD38 | CD81 | 1.81E-197 | 5.41E-193 | 0.893565142 | 0.786 | 0.532 |
| c33_gcB_CD38 | CD79B | 0 | 0 | 0.87266004 | 0.989 | 0.887 |
| c33_gcB_CD38 | CD24 | 2.15E-167 | 6.44E-163 | 0.864291239 | 0.653 | 0.373 |
| c33_gcB_CD38 | LGALS9 | 8.60E-141 | 2.57E-136 | 0.858715258 | 0.532 | 0.286 |
| c34_Plasma_IGHA1 | IGHA2 | 0 | 0 | 6.965736309 | 0.748 | 0.057 |
| c34_Plasma_IGHA1 | IGHA1 | 0 | 0 | 6.744413356 | 0.859 | 0.142 |
| c34_Plasma_IGHA1 | JCHAIN | 0 | 0 | 6.451620536 | 0.994 | 0.36 |
| c34_Plasma_IGHA1 | TXNDC5 | 0 | 0 | 5.520406067 | 0.923 | 0.142 |
| c34_Plasma_IGHA1 | AQP3 | 0 | 0 | 5.333824879 | 0.913 | 0.005 |
| c34_Plasma_IGHA1 | XBP1 | 0 | 0 | 4.85343623 | 0.973 | 0.111 |
| c34_Plasma_IGHA1 | IGKC | 9.05E-199 | 2.70E-194 | 4.853184894 | 0.675 | 0.496 |
| c34_Plasma_IGHA1 | MZB1 | 0 | 0 | 4.761688525 | 0.995 | 0.245 |
| c34_Plasma_IGHA1 | FKBP11 | 0 | 0 | 4.616769182 | 0.949 | 0.062 |
| c34_Plasma_IGHA1 | IGLC2 | 1.39E-105 | 4.15E-101 | 4.4522011 | 0.449 | 0.245 |
| c34_Plasma_IGHA1 | PRDM1 | 0 | 0 | 4.296406491 | 0.883 | 0.013 |
| c34_Plasma_IGHA1 | TNFRSF17 | 0 | 0 | 4.286343517 | 0.947 | 0.068 |
| c34_Plasma_IGHA1 | DERL3 | 0 | 0 | 4.224480702 | 0.948 | 0.108 |
| c34_Plasma_IGHA1 | FNDC3B | 0 | 0 | 4.027005438 | 0.796 | 0.013 |
| c34_Plasma_IGHA1 | HSP90B1 | 0 | 0 | 4.002387584 | 0.998 | 0.515 |
| c34_Plasma_IGHA1 | SEC11C | 0 | 0 | 3.955117694 | 0.975 | 0.208 |
| c34_Plasma_IGHA1 | ITM2C | 0 | 0 | 3.914006106 | 0.962 | 0.185 |
| c34_Plasma_IGHA1 | IGLC1 | 0 | 0 | 3.856766037 | 0.466 | 0.075 |
| c34_Plasma_IGHA1 | PDIA4 | 0 | 0 | 3.847949995 | 0.965 | 0.136 |
| c34_Plasma_IGHA1 | SDF2L1 | 0 | 0 | 3.781329804 | 0.947 | 0.095 |
| c34_Plasma_IGHA1 | PPIB | 0 | 0 | 3.759972813 | 0.996 | 0.619 |
| c34_Plasma_IGHA1 | LIME1 | 0 | 0 | 3.655836666 | 0.93 | 0.071 |
| c34_Plasma_IGHA1 | WARS | 0 | 0 | 3.509214028 | 0.882 | 0.084 |
| c34_Plasma_IGHA1 | MYDGF | 0 | 0 | 3.486767006 | 0.96 | 0.192 |
| c34_Plasma_IGHA1 | SLAMF7 | 0 | 0 | 3.474981281 | 0.84 | 0.017 |
| c34_Plasma_IGHA1 | SRGN | 0 | 0 | 3.434314681 | 0.964 | 0.148 |
| c34_Plasma_IGHA1 | SSR3 | 0 | 0 | 3.374442657 | 0.967 | 0.201 |
| c34_Plasma_IGHA1 | TXNDC11 | 0 | 0 | 3.357295207 | 0.915 | 0.076 |
| c34_Plasma_IGHA1 | CD27 | 0 | 0 | 3.318122049 | 0.911 | 0.123 |
| c34_Plasma_IGHA1 | MAN1A1 | 0 | 0 | 3.317636856 | 0.854 | 0.07 |

B cell markers.

Supplementary Table 8. The antibody panel for CyTOF.

| **List** | **Label** | **Marker** | **Clone** | **Brand** | **Catalog** |
| --- | --- | --- | --- | --- | --- |
| 1 | 89Y | CD45 | HI30 | Biolegend | 304002 |
| 2 | 115In | CD3 | UCHT1 | Bioxcell | BE0231 |
| 3 | 141Pr | CD56 | NCAM16.2 | BD | 559043 |
| 4 | 142Nd | CD19 | HIB19 | Biolegend | 302268 |
| 5 | 142Nd | TCR γ/δ | 5A6.E9 | PLT | 100P001A |
| 6 | 143Nd | CD196(CCR6) | G034E3 | Biolegend | 353402 |
| 7 | 144Nd | CD14 | M5E2 | Biolegend | 301862 |
| 8 | 145Nd | CD69 | FN50 | Biolegend | 310902 |
| 9 | 146Nd | CD123(IL-3Rα) | 6H6 | Biolegend | 306002 |
| 10 | 147Sm | CD197(CCR7) | G043H7 | Biolegend | 353256 |
| 11 | 148Nd | CD28 | CD28.2 | Bioxcell | BE0291 |
| 12 | 149Sm | CD62L | DREG-56 | Biolegend | 304854 |
| 13 | 150Nd | CD1c | L161 | Biolegend | 331502 |
| 14 | 151Eu | CD223(LAG-3) | 874501 | R&D | MAB23193 |
| 15 | 152Sm | CD39 | A1 | Biolegend | 328202 |
| 16 | 153Eu | CD27 | O323 | Biolegend | 302802 |
| 17 | 154Sm | TIGIT(VSTM3) | A15153G | Biolegend | 372702 |
| 18 | 155Gd | CD45RA | HI100 | Biolegend | 304102 |
| 19 | 156Gd | CD194(CCR4) | L291H4 | Biolegend | 359402 |
| 20 | 157Gd | CD183(CXCR3) | G025H7 | Biolegend | 353750 |
| 21 | 158Gd | CD127(IL-7Rα) | A019D5 | Biolegend | 351302 |
| 22 | 159Tb | CD11c | BU15 | Biolegend | 337202 |
| 23 | 160Gd | CD25(IL-2Rα) | 24212 | R&D | MAB1020 |
| 24 | 161Dy | CD152 (CTLA-4) | 14D3 | eBioscience | 14-1529-82 |
| 25 | 162Dy | FOXP3 | PCH101 | eBioscience | 14-4776-82 |
| 26 | 163Dy | CD33 | WM53 | Biolegend | 303419 |
| 27 | 164Dy | CD141(Thrombomodulin) | M80 | Biolegend | 344102 |
| 28 | 165Ho | CD303(BDCA-2) | 201A | Biolegend | 354215 |
| 29 | 166Er | CD16 | 3G8 | Biolegend | 302057 |
| 30 | 167Er | Granzyme B Recombinant | QA16A02 | Biolegend | 372202 |
| 31 | 168Er | T-bet | 4B10 | Biolegend | 644802 |
| 32 | 169Tm | CD185(CXCR5) | RF8B2 | BD | 552032 |
| 33 | 170Er | CD366(Tim-3) | F38-2E2 | Biolegend | 345010 |
| 34 | 171Yb | CD66b | G10F5 | Biolegend | 305102 |
| 35 | 172Yb | CD38 | HIT2 | Biolegend | 303502 |
| 36 | 173Yb | CD278(ICOS) | C398.4A | Biolegend | 313502 |
| 37 | 174Yb | CD279(PD-1) | EH12.2H7 | Biolegend | 329926 |
| 38 | 175Lu | Ki-67 | SolA15 | eBioscience | 14-5698-82 |
| 39 | 176Yb | HLA-DR | L243 | Biolegend | 307648 |
| 40 | 197Au | CD4 | RPA-T4 | Biolegend | 300570 |
| 41 | 198Pt | CD8a | RPA-T8 | Biolegend | 301074 |
| 42 | 209Bi | CD11b | M1/70 | Biolegend | 101202 |

The antibody panel for CyTOF.

Supplementary Table 9. Comparisons of differences in immune cells across different time points.

| **Cell types** | | **All groups** | | | | | **Cohort I** | | | **Cohort II** | | | **Cohort IV** | | |
| --- | --- | --- | --- | --- | --- | --- | --- | --- | --- | --- | --- | --- | --- | --- | --- |
|  |  | **Baseline-Healthy** | **D3-Baseline** | **D7-Baseline** | **D14-Baseline** | **D28-Baseline** | **D3-Baseline** | **D7-Baseline** | **D14-Baseline** | **D3-Baseline** | **D7-Baseline** | **D14-Baseline** | **D3-Baseline** | **D7-Baseline** | **D14-Baseline** |
| **Immune cells** | **T** | 0.256427158970776 | 0.1827469759706 | 0.871161695230835 | 0.402331205717249 | 0.0892618955644265 | 0.0738399419839733 | 0.122763901943368 | 0.962727714396425 | 0.180066414121576 | 0.372533581280726 | 0.984856398001706 | 0.307697111753333 | 0.521967573547335 | 0.139281052800029 |
|  | **B** | 0.331355132790143 | 0.338019704289435 | 0.934454984253233 | 0.110063876458213 | 0.0198749631475366 | 0.799808096292727 | 0.539018288971566 | 0.370840622622664 | 0.106981717781153 | 0.253317909922709 | 0.526365375027492 | 0.534388396641254 | 0.282813018604661 | 0.0354689752767396 |
|  | **NK** | 0.687896671741836 | 0.497788170388123 | 0.0753160319325576 | 0.762708277414604 | 0.580859071191952 | 0.290733469334254 | 0.235885394224057 | 0.601460355695961 | 0.247982106493201 | 0.477640532498162 | 0.420402884972354 | 0.857309086912106 | 0.415246803382319 | 0.612413188981199 |
|  | **Monocyte** | 0.0291923119828763 | 0.997118136640525 | 0.631844542048425 | 0.744754731608256 | 0.236675250662297 | 0.168006596309026 | 0.0876905205827685 | 0.128160840841308 | 0.0490088410690333 | 0.447962847896997 | 0.735169425 | 0.62501002825102 | 0.469858508116028 | 0.0505610367859557 |
| **Monocytes** | **c35_Mono_VCAN** | 0.974791928255852 | 0.452806021318928 | 0.721175440317728 | 0.489807017789301 | 0.36696335430686 | 0.213361897138628 | 0.180103974417725 | 0.713197476635249 | 0.995166305842801 | 0.860893487814689 | 0.735981612961286 | 0.507106431457666 | 0.557361841093783 | 0.509126334681141 |
|  | **c36_Mono_LGALS2** | 0.0415550267266748 | 0.0191474128463496 | 0.3699820583492 | 0.701645649241754 | 0.875213403821621 | 0.424434640735044 | 0.678883117302704 | 0.570528175779647 | 0.383229486767249 | 0.874296020878845 | 0.100914518261919 | 0.0685167618257062 | 0.210880029367782 | 0.530237328249596 |
|  | **c37_Mono_HLA-DPB1** | 0.230035401531157 | 0.491356187043921 | 0.751849796965055 | 0.113962925542695 | 0.563170995462988 | 0.235713606103504 | 0.421446564736939 | 0.478884156006223 | 0.979788233203049 | 0.910664523610558 | 0.769336754481097 | 0.572937994257394 | 0.943479353406494 | 0.196387928744134 |
|  | **c38_Mono_MX1** | 0.00929550155661888 | 0.0547722339816085 | 0.857602324863127 | 0.453366231268352 | 0.945628644772449 | 0.786884232570476 | 0.442302438939046 | 0.451199346879067 | 0.0965488761907067 | 0.709787967330758 | 0.212093546632403 | 0.207498057405645 | 0.253472881701267 | 0.446016690177174 |
|  | **c39_Mono_FCGR3A** | 0.0919470964993035 | 0.393480897835829 | 0.319572210483591 | 0.408453688934863 | 0.515039048237639 | 0.446366799438928 | 0.769843372964371 | 0.459134211600283 | 0.396210314202853 | 0.944806736522013 | 0.492860946265016 | 0.80207638394719 | 0.210049231370784 | 0.267356109268222 |
| **T cells** | **c1_CD4Tn_CCR7** | 0.702366229436132 | 0.35018393923411 | 0.417143263126825 | 0.190614040876414 | 0.617948961756733 | 0.0937003714935182 | 0.638378207925953 | 0.30020322483875 | 0.979323191615861 | 0.573394424656834 | 0.588663046020004 | 0.887918908321049 | 0.467390385761774 | 0.944099543445427 |
|  | **c2_CD4Tcm_GPR183** | 0.0746212411856041 | 0.596454552366446 | 0.0119120314369113 | 0.791466197989369 | 0.622501782362536 | 0.718732449323138 | 0.192589386677969 | 0.536522348675312 | 0.719335967945126 | 0.186474522954017 | 0.236343295888091 | 0.66819051718413 | 0.0684655762933172 | 0.241083877525553 |
|  | **c5_CD4Treg_FOXP3** | 0.00136348568349582 | 0.0753784792790269 | 0.573247624486258 | 0.325852354365798 | 0.587493939297179 | 0.338586928163975 | 0.680391469912569 | 0.690990459598848 | 0.0334709352103556 | 0.960653979316241 | 0.418493945102946 | 0.89502585181189 | 0.699786152047184 | 0.707386486595391 |
|  | **c6_CD8Tn_CCR7** | 0.625494107034049 | 0.482825014344057 | 0.297127192568661 | 0.0507564762662952 | 0.848179460461428 | 0.275235452840615 | 0.844568014839998 | 0.0899324964646785 | 0.76143932680855 | 0.839794640093764 | 0.565926430086959 | 0.953385290201485 | 0.111613796317524 | 0.400381100652761 |
|  | **c8_CD8Tem_GZMK** | 0.0195460997568246 | 0.728396772114377 | 0.708118068617254 | 0.271066752775181 | 0.334471427244976 | 0.526518657236071 | 0.106526983755054 | 0.409288154539624 | 0.217822005287996 | 0.97420922230718 | 0.549451608037465 | 0.631630264549592 | 0.739382420445434 | 0.770194631381827 |
|  | **c9_CD8Teff_ZNF683** | 0.149857200214323 | 0.0364191174998155 | 0.0193530352779847 | 0.58542682695064 | 0.728855806548661 | 0.0225976707371769 | 0.0177683017897578 | 0.649441297827615 | 0.338327599618351 | 0.647991597066195 | 0.519628040150473 | 0.46219339839955 | 0.284071791483657 | 0.328975620296125 |
|  | **c10_CD8Teff_NKG7** | 0.0291308455611967 | 0.262752709872542 | 0.558524244313336 | 0.954784479747047 | 0.551322828115202 | 0.892772407520443 | 0.796918988924607 | 0.597482488594711 | 0.26953848309949 | 0.22031401057964 | 0.348657938671211 | 0.38738368257126 | 0.30940070905767 | 0.523730158329579 |
| **NK cells** | **c18_NK_FCER1G** | 0.141556308545965 | 0.367625478343262 | 0.337179580217104 | 0.69852455500801 | 0.475106053309184 | 0.238758755664448 | 0.412461230328016 | 0.546749212376809 | 0.742590525690779 | 0.772288337158146 | 0.694644954875798 | 0.53387249432885 | 0.454440234284847 | 0.0252863476122705 |
|  | **c19_NK_KLRC2** | 0.623849126312418 | 0.903838613507562 | 0.847289031657654 | 0.336242562663299 | 0.158399447389733 | 0.557127318843441 | 0.646500791095944 | 0.364756751127523 | 0.303717545543651 | 0.5 | 0.825908706056565 | 0.146624320763816 | 0.152991441266755 | 0.769559854854691 |
|  | **c20_NK_IL32** | 0.505083596753768 | 0.117097754614293 | 0.0387998686554459 | 0.428586055402228 | 0.423006019520465 | 0.0678022412230724 | 0.010179828967957 | 0.750488752687403 | 0.928126727852711 | 0.191464528730225 | 0.142720937957126 | 0.502267636083264 | 0.338253300840547 | 0.672392273931118 |
|  | **c21_NK_HLA-DRA** | 0.440469255823744 | 0.793442424465229 | 0.00269006180369557 | 0.962198735131561 | 0.317811504437779 | 0.764481328207261 | 0.136221619293113 | 0.919413352418837 | 0.872847526842378 | 0.460799856871232 | 0.495374338648671 | 0.603041973401142 | 0.0585254829120883 | 0.315051660125796 |
|  | **c22_NK_GZMK** | 0.0230275767310323 | 0.853401270569473 | 0.666879455833295 | 0.696130487095172 | 0.621051564669156 | 0.225770065214764 | 0.274658994212979 | 0.426305319306114 | 0.266866316714259 | 0.102821513536387 | 0.82606920672662 | 0.618498584512844 | 0.699135692742759 | 0.438224619559217 |
|  | **c23_NK_XCL1** | 0.124757393253006 | 0.0453167603835103 | 0.125615741123493 | 0.459148419377244 | 0.787474512424757 | 0.0271230676510833 | 0.0960729268830597 | 0.518062817606995 | 0.501755574825193 | 0.500701573053445 | 0.998378238713628 | 0.102238851925952 | 0.775626422582501 | 0.996669449780366 |
| **B cells** | **c26-c28_NaiveB** | 1.4509801757815e-06 | 0.382662251221278 | 0.279852201344591 | 0.132602348542955 | 0.0639975965077412 | 0.123057017834551 | 0.348451752116795 | 0.352040029888805 | 0.626112098724768 | 0.224559675658132 | 0.347602470719381 | 0.262546182381963 | 0.272597849259215 | 0.0947444944193343 |
|  | **c29-c32_MemB** | 3.98732863049709e-08 | 0.992827872819463 | 0.466948025005666 | 0.230967571345002 | 0.653512313232612 | 0.124753136943102 | 0.389499219793574 | 0.408762383295559 | 0.790662719863751 | 0.515641356920152 | 0.674491235676318 | 0.871572418096392 | 0.0588698818363863 | 0.290790724751386 |
|  | **c33_gcB_CD38** | 0.0430435102140995 | 0.854975900294036 | 0.704686816992485 | 0.343047062504405 | 0.434733293176022 | 0.930450435273194 | 0.292019006771328 | 0.818749994309966 | 0.685408990917265 | 0.495401071118792 | 0.171994324889012 | 0.59849206935599 | 0.705286183468876 | 0.700416197250476 |

Comparisons of differences in immune cells across different time points, Wilcoxon signed-rank test.

Supplementary Table 10. Comparison of differences in immune cells among each dose groups.

| **Cell** | **contrast** | **estimate** | **SE** | **df** | **t.ratio** | **p.value** |
| --- | --- | --- | --- | --- | --- | --- |
| T | L - M | -0.022010053 | 0.110248074747348 | 6.0143200293587 | -0.199641159 | 0.978346119550295 |
| T | L - V | -0.08254954 | 0.110040497320089 | 5.97039592425935 | -0.750174182 | 0.744695746136323 |
| T | M - V | -0.060539487 | 0.110248074747348 | 6.01432002935871 | -0.54912058 | 0.850708732027768 |
| B | L - M | 0.0921016953029976 | 0.0432385637898757 | 6.0214729836258 | 2.13008220510236 | 0.163364382788172 |
| B | L - V | 0.0185761278987877 | 0.0431130941043927 | 5.95478867986532 | 0.430869745831928 | 0.904309535109454 |
| B | M - V | -0.073525567 | 0.0432385637898758 | 6.02147298362578 | -1.700462757 | 0.279695373828034 |
| NK | L - M | 0.0381047568458033 | 0.0774496387831558 | 6.01116537206247 | 0.491993990475402 | 0.877662912236491 |
| NK | L - V | -0.054033939 | 0.077337250890547 | 5.97709396324335 | -0.69867934 | 0.773068902514191 |
| NK | M - V | -0.092138696 | 0.0774496387831559 | 6.01116537206237 | -1.189659471 | 0.500680717952216 |
| Monocyte | L - M | -0.081968988 | 0.0683052763940702 | 6.0504656848086 | -1.200038885 | 0.495065230941121 |
| Monocyte | L - V | 0.0804057092934708 | 0.0677730836451751 | 5.88348974619035 | 1.18639591071927 | 0.503172150557012 |
| Monocyte | M - V | 0.162374697011926 | 0.0683052763940702 | 6.0504656848086 | 2.37719112759526 | 0.118792333115531 |
|  |  |  |  |  |  |  |
| c35_Mono_VCAN | L - M | -0.007981077 | 0.0536844580996903 | 6.06926022667819 | -0.148666434 | 0.987913474920868 |
| c35_Mono_VCAN | L - V | 0.0708071225601811 | 0.0530371675943393 | 5.82549294849018 | 1.33504720881321 | 0.429972196721263 |
| c35_Mono_VCAN | M - V | 0.0787881995301053 | 0.0536844580996903 | 6.06926022667818 | 1.46761655643051 | 0.368398216543562 |
| c36_Mono_LGALS2 | L - M | -0.004690634 | 0.0256631237125491 | 6.08882916098889 | -0.182777193 | 0.981806218230054 |
| c36_Mono_LGALS2 | L - V | -0.01372418 | 0.0247838110977032 | 5.56804547406042 | -0.553755841 | 0.848744961565295 |
| c36_Mono_LGALS2 | M - V | -0.009033546 | 0.0256631237125491 | 6.08882916098889 | -0.352004945 | 0.934694140078816 |
| c37_Mono_HLA-DPB1 | L - M | 0.0355036921580573 | 0.0345519593626179 | 6.08452429237364 | 1.02754497322282 | 0.58775091253685 |
| c37_Mono_HLA-DPB1 | L - V | 0.0275338468329434 | 0.0339617822780088 | 5.76138338517959 | 0.810730326446157 | 0.711197227423908 |
| c37_Mono_HLA-DPB1 | M - V | -0.007969845 | 0.0345519593626179 | 6.08452429237365 | -0.230662616 | 0.971233099922291 |
| c38_Mono_MX1 | L - M | 0.0125194090270581 | 0.0225338940238757 | 6.08750312213805 | 0.55558125079461 | 0.847503539751112 |
| c38_Mono_MX1 | L - V | -0.018611543 | 0.0217451135250108 | 5.56043956043954 | -0.85589541 | 0.686280919834943 |
| c38_Mono_MX1 | M - V | -0.031130952 | 0.0225338940238757 | 6.08750312213804 | -1.381516743 | 0.406272239025505 |
| c39_Mono_FCGR3A | L - M | -0.037124292 | 0.0467932931280077 | 6.04681989521236 | -0.793367797 | 0.720345889181071 |
| c39_Mono_FCGR3A | L - V | -0.066005246 | 0.0464615224683464 | 5.89337715393599 | -1.420643209 | 0.390344813421698 |
| c39_Mono_FCGR3A | M - V | -0.028880955 | 0.0467932931280077 | 6.04681989521236 | -0.617202863 | 0.816397387928459 |
|  |  |  |  |  |  |  |
| c1_CD4Tn_CCR7 | L - M | 0.101690268031256 | 0.0605358771455889 | 6.02203718611843 | 1.67983471663738 | 0.286791147674922 |
| c1_CD4Tn_CCR7 | L - V | -0.01817034 | 0.060355195293699 | 5.95353103874341 | -0.301056769 | 0.951664548703181 |
| c1_CD4Tn_CCR7 | M - V | -0.119860608 | 0.0605358771455889 | 6.02203718611847 | -1.979992919 | 0.197617496534843 |
| c2_CD4Tcm_GPR183 | L - M | -0.000616796 | 0.059807472575785 | 6.00974913555282 | -0.010313024 | 0.999941363819663 |
| c2_CD4Tcm_GPR183 | L - V | 0.0452732227552374 | 0.0597320804464192 | 5.98006668718577 | 0.75793815344919 | 0.7403515161409 |
| c2_CD4Tcm_GPR183 | M - V | 0.0458900186275515 | 0.059807472575785 | 6.00974913555281 | 0.767295734983651 | 0.735066641835818 |
| c5_CD4Treg_FOXP3 | L - M | 0.00473406340309608 | 0.0098693374544554 | 6.02681587997376 | 0.479673881346406 | 0.883220030125953 |
| c5_CD4Treg_FOXP3 | L - V | 0.0174573464073305 | 0.00983279111523545 | 5.94270938119357 | 1.77542126164779 | 0.256047864123319 |
| c5_CD4Treg_FOXP3 | M - V | 0.0127232830042344 | 0.0098693374544554 | 6.02681587997377 | 1.28917296251641 | 0.450419735945105 |
| c6_CD8Tn_CCR7 | L - M | 0.00504886549198903 | 0.0516039808344793 | 6.01091002505872 | 0.0978386824106333 | 0.994741101669479 |
| c6_CD8Tn_CCR7 | L - V | -0.081322331 | 0.0515308781358243 | 5.9776314813157 | -1.578128181 | 0.324496372227471 |
| c6_CD8Tn_CCR7 | M - V | -0.086371196 | 0.0516039808344792 | 6.01091002505883 | -1.673731271 | 0.289041140996121 |
| c8_CD8Tem_GZMK | L - M | 0.0110330867626941 | 0.0177990001799425 | 6.09315888241436 | 0.619871152938533 | 0.814970134603317 |
| c8_CD8Tem_GZMK | L - V | 0.0125610401351749 | 0.0172456642663422 | 5.60126349970412 | 0.728359310559573 | 0.757420657910983 |
| c8_CD8Tem_GZMK | M - V | 0.00152795337248078 | 0.0177990001799425 | 6.09315888241436 | 0.0858448989849787 | 0.99594808804879 |
| c9_CD8Teff_ZNF683 | L - M | -0.040672933 | 0.0258242133606665 | 6.03844682302236 | -1.574992131 | 0.325092441774342 |
| c9_CD8Teff_ZNF683 | L - V | -0.003536064 | 0.0256799178234038 | 5.9149286990973 | -0.137697628 | 0.989619691950945 |
| c9_CD8Teff_ZNF683 | M - V | 0.0371368690610121 | 0.0258242133606665 | 6.03844682302233 | 1.43806390314201 | 0.38141409919661 |
| c10_CD8Teff_NKG7 | L - M | -0.034255356 | 0.0318667137341679 | 6.05558828655158 | -1.07495727 | 0.561693107755092 |
| c10_CD8Teff_NKG7 | L - V | 0.0245079916535026 | 0.031585197346915 | 5.86897917234728 | 0.775932832849511 | 0.730504998296226 |
| c10_CD8Teff_NKG7 | M - V | 0.0587633472512003 | 0.0318667137341679 | 6.05558828655157 | 1.84403536999152 | 0.23395945741103 |
|  |  |  |  |  |  |  |
| c18_NK_FCER1G | L - M | -0.100389577 | 0.083137735978849 | 6.06744531938554 | -1.207509149 | 0.491109609660652 |
| c18_NK_FCER1G | L - V | 0.115779210622696 | 0.0821747797431804 | 5.83177371029134 | 1.40893849651363 | 0.39608445009897 |
| c18_NK_FCER1G | M - V | 0.216168787480234 | 0.083137735978849 | 6.06744531938554 | 2.60012838857229 | 0.0891228502343615 |
| c19_NK_KLRC2 | L - M | -0.03392626 | 0.123408509454105 | 6.00333395483116 | -0.274910219 | 0.959472271583372 |
| c19_NK_KLRC2 | L - V | -0.00419977 | 0.123356494669315 | 5.9932830008935 | -0.034045792 | 0.999361220206821 |
| c19_NK_KLRC2 | M - V | 0.0297264907648535 | 0.123408509454105 | 6.00333395483116 | 0.24087877648266 | 0.96868562196567 |
| c20_NK_IL32 | L - M | 0.029786983893946 | 0.0776106428106905 | 6.00885153743009 | 0.383800247172325 | 0.923022087993663 |
| c20_NK_IL32 | L - V | -0.069139742 | 0.0775221010731464 | 5.98194013531681 | -0.891871358 | 0.664652480415826 |
| c20_NK_IL32 | M - V | -0.098926725 | 0.0776106428106905 | 6.00885153743012 | -1.274654118 | 0.457672537449868 |
| c21_NK_HLA-DRA | L - M | 0.0811745418413879 | 0.149391816876001 | 6.0017080287717 | 0.54336672207933 | 0.853511870334519 |
| c21_NK_HLA-DRA | L - V | -0.138568359 | 0.149359736866526 | 5.9965711706841 | -0.927749084 | 0.644238905693475 |
| c21_NK_HLA-DRA | M - V | -0.219742901 | 0.149391817 | 6.00170802877188 | -1.470916584 | 0.367607662546851 |
| c22_NK_GZMK | L - M | 0.0100104632665655 | 0.0628201295354851 | 6.01152349788995 | 0.159351203835244 | 0.986130782077596 |
| c22_NK_GZMK | L - V | 0.0346400395112398 | 0.0627259241172878 | 5.97633893952025 | 0.552244386969386 | 0.849207454165341 |
| c22_NK_GZMK | M - V | 0.0246295762446743 | 0.0628201295354851 | 6.01152349788996 | 0.392065034357527 | 0.919854125201978 |
| c23_NK_XCL1 | L - M | -0.00686717 | 0.0258948337664403 | 6.09065417027464 | -0.265194609 | 0.962210786299962 |
| c23_NK_XCL1 | L - V | 0.0208259560683672 | 0.0253705110557532 | 5.72297672642774 | 0.820872548550596 | 0.705578150552565 |
| c23_NK_XCL1 | M - V | 0.0276931263813267 | 0.0258948337664403 | 6.09065417027464 | 1.0694459995807 | 0.56454937469575 |
|  |  |  |  |  |  |  |
| c26-c28_NaiveB | L - M | 0.0786831066326369 | 0.125082326528922 | 6.03969472979052 | 0.629050552672952 | 0.81023300360635 |
| c26-c28_NaiveB | L - V | -0.031599263 | 0.12435648164822 | 5.91180855129251 | -0.254102261 | 0.96523933507874 |
| c26-c28_NaiveB | M - V | -0.11028237 | 0.125082326528922 | 6.03969472979053 | -0.881678274 | 0.67027962029852 |
| c29-c32_MemB | L - M | -0.021778171 | 0.112313034313524 | 6.04743203313948 | -0.193905995 | 0.979555076381737 |
| c29-c32_MemB | L - V | 0.0525095425118333 | 0.111503740022766 | 5.89174034191142 | 0.47092180496468 | 0.887162527209378 |
| c29-c32_MemB | M - V | 0.074287713125929 | 0.112313034313524 | 6.04743203313947 | 0.661434477128927 | 0.793093751595871 |
| c33_gcB_CD38 | L - M | 0.0152859182395666 | 0.02315876374542 | 6.02824035521134 | 0.660048973580882 | 0.793855930415143 |
| c33_gcB_CD38 | L - V | 0.00641284663744424 | 0.0230679123925562 | 5.93942195067911 | 0.277998569108213 | 0.958585361379642 |
| c33_gcB_CD38 | M - V | -0.008873072 | 0.02315876374542 | 6.02824035521131 | -0.383140987 | 0.923269323734206 |
| c34_Plasma_IGHA1 | L - M | -0.070355028 | 0.029337094266575 | 6.07697396140085 | -2.398159397 | 0.115332818834316 |
| c34_Plasma_IGHA1 | L - V | -0.027323126 | 0.0289173260175348 | 5.79624732685528 | -0.94487042 | 0.635260755657177 |
| c34_Plasma_IGHA1 | M - V | 0.043031902322937 | 0.029337094266575 | 6.07697396140086 | 1.46680860523958 | 0.368673521447364 |

Comparison of differences in immune cells among each dose groups.

Supplementary File 1. (separate file)

Clinical Study Protocol —separate file

Supplementary File 2. (separate file)

Statistical Analysis Plan (SAP) —separate file
